# A tool box for operational mosquito larval control: preliminary results and early lessons from the Urban Malaria Control Programme in Dar es Salaam, Tanzania

**DOI:** 10.1186/1475-2875-7-20

**Published:** 2008-01-25

**Authors:** Ulrike Fillinger, Khadija Kannady, George William, Michael J Vanek, Stefan Dongus, Dickson Nyika, Yvonne Geissbühler, Prosper P Chaki, Nico J Govella, Evan M Mathenge, Burton H Singer, Hassan Mshinda, Steven W Lindsay, Marcel Tanner, Deo Mtasiwa, Marcia C de Castro, Gerry F Killeen

**Affiliations:** 1 Durham University, School of Biological and Biomedical Sciences, South Road, Durham DH13LE, UK; 2 Dar es Salaam City Council, Ministry of Regional Administration and Local Government, United Republic of Tanzania; 3 Swiss Tropical Institute, Department of Public Health and Epidemiology, PO Box, 4002 Basel, Switzerland; 4 University of Freiburg, Department of Physical Geography, Freiburg, Germany; 5 Ifakara Health Research and Development Centre, Coordination Office, PO Box 78373, Kiko Avenue, Mikocheni, Dar es Salaam, United Republic of Tanzania; 6 Ministry of Agriculture and Food Security, Dar es Salaam, United Republic of Tanzania; 7 Kenya Medical Research Institute, PO Box 54840, Nairobi, Kenya, Africa; 8 Office of Population Research, Princeton University, Princeton, NJ08544, USA; 9 Harvard School of Public Health, Department of Population and International Health, 665 Huntington Avenue, Boston, MA 02115, USA

## Abstract

**Background:**

As the population of Africa rapidly urbanizes, large populations could be protected from malaria by controlling aquatic stages of mosquitoes if cost-effective and scalable implementation systems can be designed.

**Methods:**

A recently initiated Urban Malaria Control Programme in Dar es Salaam delegates responsibility for routine mosquito control and surveillance to modestly-paid community members, known as Community-Owned Resource Persons (CORPs). New vector surveillance, larviciding and management systems were designed and evaluated in 15 city wards to allow timely collection, interpretation and reaction to entomologic monitoring data using practical procedures that rely on minimal technology. After one year of baseline data collection, operational larviciding with *Bacillus thuringiensis var. israelensis *commenced in March 2006 in three selected wards.

**Results:**

The procedures and staff management systems described greatly improved standards of larval surveillance relative to that reported at the outset of this programme. In the first year of the programme, over 65,000 potential *Anopheles *habitats were surveyed by 90 CORPs on a weekly basis. Reaction times to vector surveillance at observations were one day, week and month at ward, municipal and city levels, respectively. One year of community-based larviciding reduced transmission by the primary malaria vector, *Anopheles gambiae s.l*., by 31% (95% C.I. = 21.6–37.6%; p = 0.04).

**Conclusion:**

This novel management, monitoring and evaluation system for implementing routine larviciding of malaria vectors in African cities has shown considerable potential for sustained, rapidly responsive, data-driven and affordable application. Nevertheless, the true programmatic value of larviciding in urban Africa can only be established through longer-term programmes which are stably financed and allow the operational teams and management infrastructures to mature by learning from experience.

## Background

With the prospect of more than half of the African population living in urban areas by the year 2030, it is anticipated that the challenge and opportunity for tackling malaria burden in urban areas will also grow [1-3]. Compared to rural settings, malaria in urban Africa is generally characterized by lower intensities and more focal distribution of transmission, resulting in weaker immunity in the afflicted population and distribution of disease burden across older age groups [2,3]. Compared to rural settings, urban areas usually offer more malaria control options because relatively good transport, communication, educational and health infrastructure is available to large populations in small geographic areas. Since there is relatively easy access to most urban area breeding sites, control interventions such as environmental control and larvicide application may be cost-effective [2,3], but remain to be rigorously evaluated in the modern African context [4-6]. Although locally targeted approaches [7-9] are desirable, and this may be realizable in the future [10-13], all documented successes of larval control against African malaria vectors have depended on rigorous and comprehensive surveillance for aquatic stage mosquitoes [14] to enable wholesale suppression [15] and even elimination [16,17]. To be sustainable in the context of African cities today, integrated vector management needs to be implemented through community-based systems using simple tools that are appropriately tailored to the enormous reservoir of affordable labour that is available *in situ *[18-20].

Although most malaria research has generally focused on rural settings [1-3,21], Dar es Salaam in Tanzania is one of the few African cities in which the distinctive characteristics of urban malaria ecology and epidemiology have been examined in depth with useful records dating back almost a century [22-25]. The main vectors of malaria in the area of Dar es Salaam are *Anopheles gambiae sensu stricto*, *Anopheles arabiensis*, *Anopheles funestus *and *Anopheles merus *[26]. *Plasmodium falciparum *is the most common malaria parasite, accounting for 90% of all cases [22]. Interestingly, malaria vectors in the city appear to have adapted to high coverage with bed nets and improved housing by predominantly feeding outdoors [26]. Thus, insecticide-treated nets confer slightly less protection than in rural areas so additional measures directed at aquatic stages of vector mosquitoes may have a useful role in this and similar urban settings [26].

This publication describes the principles and practices of a novel management system for implementing, monitoring and optimizing routine larviciding in African cities that was developed at the City Council of Dar es Salaam in Tanzania. It aims to provide an array of tools which can be adapted to different ecological settings for programmes aiming to integrate anti-larval interventions in ongoing malaria control programmes. Furthermore, preliminary results obtained in the first year of operation are described and the potential of these systems are discussed.

## Methods

### Study site

The study was conducted in Dar es Salaam, Tanzania's biggest and economically most important city with 2.7 million inhabitants and a total area of 1400 km^2 ^[22,27]. The city is divided into three municipalities, namely Ilala, Kinondoni and Temeke. Each of these municipalities is further divided into wards and then neighbourhoods known as m*itaa *(singular *mtaa*) in Kiswahili, literally meaning street [28].

A recently-initiated Urban Malaria Control Programme (UMCP) in Dar es Salaam delegates responsibility for routine mosquito control and surveillance to modestly paid community members, known as Community-Owned Resource Persons (CORPs) in a decentralized manner [29]. However, baseline evaluation revealed that at the early stage of the UMCP the levels of coverage achieved by the CORPs were insufficient to enable effective suppression of malaria transmission through larval control, and that training, support and supervision of the CORPs was poor [24]. The authors concluded that novel surveillance systems were required to enable community-based integrated vector management [24].

Early experience also indicated that control of culicine species, responsible for the bulk of biting nuisance [30-32], would be essential to achieve community acceptance and support for the programme. It was therefore decided to prioritize intensive control of malaria vector species in habitats which are open to sunlight (referred to as "open habitats") but to also implement less intensive control of sanitation structures, such as pit latrines, soakage pits, and container type habitats which are closed to the sun (referred to as "closed habitats") and produce huge numbers of *Culex *and *Aedes*, but no *Anopheles *[33,34]. Thus, the bulk of the programme description below prioritizes and focuses on the system for controlling open habitats suitable for *Anopheles*, with a brief section describing mosquito control in closed habitats, for which no detailed routine larval surveillance was undertaken.

### A strategic overview of the Dar es Salaam Urban Malaria Control Programme (UMCP)

**Figure 1:**
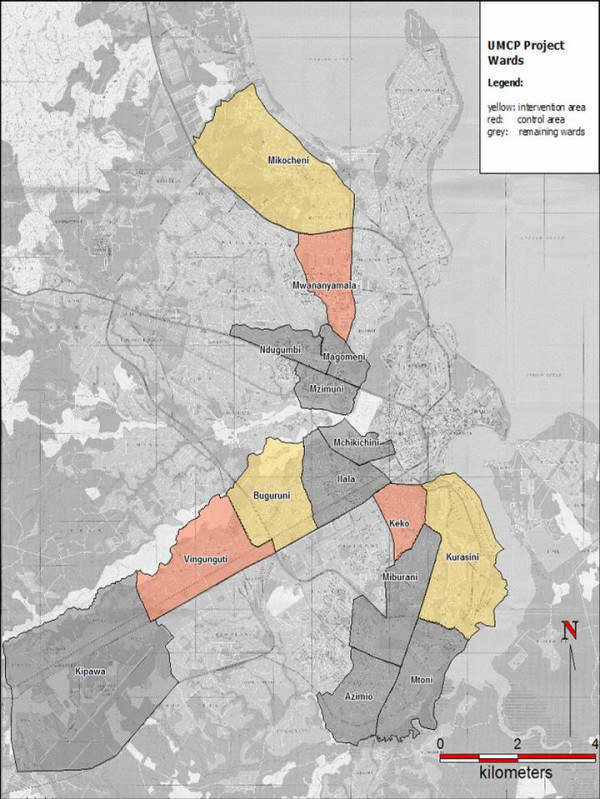
Wards included in the study area of the Dar es Salaam Urban Malaria Control Programme (UMCP), specifying those targeted for larviciding from March 2006 onwards (intervention), those considered to be the most comparable control (non-intervention wards) and those remaining.

Fifteen wards were included in the Dar es Salaam UMCP (Figure 1) encompassing as wide a variety of malariological situations as possible. In total an area of 55 km^2 ^is covered with wards ranging from 0.96 to 15 km^2 ^in size. In 2002, 611,871 people, representing 23% of the urban population, lived within this area [27] which covers 4% of the surface area of urban Dar es Salaam. By April 2007 all 15 wards had been mapped in detail as a precursor to systematic larviciding [28]. Acronyms and other specific terminology are defined and explained in Table 1. The Dar es Salaam UMCP was conceptualized and developed according to the key principles listed in Table 2 which were formulated on the basis of direct practical experience [23,24,29,35-38] and an extensive literature review [5,6,12,29].

**Table 1:** Definitions and abbreviations

**Closed habitat**	Any stagnant or slow-flowing water body which is not exposed to the sun and therefore unlikely to produce Anopheles malaria vectors but may produce culicines, notably abundant *Culex quinquefasciatus *[33, 34].
**CORP**	Community-Owned Resource Person. The responsibility for routine mosquito surveillance and application of larvicide is delegated to CORPs, who are individual community members appointed and managed through neighbourhood health committees [29].
**GIS**	Geographical Information System. GIS is a set of tools for capturing, storing, retrieving, transforming and displaying spatial data.
**GPS**	Global Positioning System. An operational system that allow receiving and converting signals from satellites to a specific position on Earth.
**Municipality**	The Dar es Salaam City Region is subdivided into three municipalities (the equivalent term for districts in urban Tanzania), namely Ilala, Temeke and Kinondoni.
**Neighbourhood**	The 73 wards of the Dar es Salaam City Region are administratively subdivided into 368 neighbourhoods. The 15 wards covered by UMCP comprise 67 neighbourhoods. The local Kiswahili term for neighbourhood is *mtaa *(plural *mitaa*) which literally means "street".
**Open habitat**	Any stagnant or slow-flowing water body which is openly exposed to sunlight, even if only partially and for a portion of the day. These constitute potential habitats for malaria vector *Anopheles *mosquitoes [61, 70], as well as a variety of culicines.
**Plot**	All TCUs within the wards covered by the UMCP are subdivided into plots. A plot is defined here as a specific physical area with an identifiable owner, occupant or user and with clearly defined boundaries within one specific TCU. The plot boundaries are defined by UMCP staff. Therefore, the plots do not always correspond to actual cadastral information such as land ownership.
**Region**	The United Republic of Tanzania is divided into 26 administrative regions, of which Dar es Salaam city and its associated hinterland is one.
**TCU**	Ten-Cell-Unit. The 368 neighbourhoods (mitaa) of the Dar es Salaam City Region are subdivided into several thousand ten-cell-units (TCUs). These are the smallest units of local government, headed by a locally elected chairperson. In principle, TCUs should comprise ten houses each but are typically larger in practice and sometimes exceed one hundred houses.
**UMCP**	Urban Malaria Control Programme of the Dar es Salaam City Medical Office of Health, developed in co-operation with national and international research and funding organizations.
**Ward**	The three municipalities of the Dar es Salaam City Region are subdivided into 73 administrative sub-units known as wards. Currently, 15 of these wards are covered by the UMCP.

**Table 2:** Conceptual principles underlying development of the Dar es Salaam Urban Malaria Control Programme on the basis of direct practical experience [23, 24, 29, 35-38] and an extensive literature review [5, 6, 12, 29]

**Rapid response**	*An. gambiae *sibling species readily develop from egg to adult within a week in habitats that often occur transiently and unpredictably [61, 70] so surveillance and larvicide application must be implemented in cycles of a week or less, with consequent responses to observed failures executed within 24 hours [14, 17, 36].
**Community-based implementation**	Sustainable programmes in Africa will be predominantly staffed by community-based personnel with minimal educational qualifications [29, 71-73] so simple protocols and readily-verifiable targets that can be managed with minimal technology are essential to achieve effectiveness [12].
**Decentralization**	Given these resource limitations and the sheer abundance of mosquito aquatic habitats in tropical Africa, responsibility for surveillance and response to operational monitoring observations must therefore be devolved to staff assigned to geographic sub-units small enough to be traversed daily on foot.
**Comprehensive coverage**	Until reliable, generalizable and practical procedures are developed which allow targeting of the most productive malaria vector habitats [10, 11] under such programmatic circumstances, high coverage of all potential sources [4, 5, 14-17, 74] is necessary to achieve satisfactory reductions of malaria transmission and burden in African settings [12, 75].
**Rigorous vertical management**	To achieve sufficient coverage, such decentralized, community-based approaches will require new tools for hierarchical, centralized management that individualize responsibility for all program activities [5, 17] and allow rigorous monitoring, evaluation and adaptive tuning [24]. Each level of management from the CORPs up to the City Mosquito Control Coordinator is responsible for identifying and addressing all programmatic shortcomings under their purview before they are detected by the next highest level within the program or external evaluators such as donors or research partners.
**Adult mosquito densities as a priority performance indicator**	Larval surveillance alone is inadequate to monitor or evaluate larviciding programs because it only reflects observations in habitats successfully covered by surveillance activities. Weekly monitoring of adult mosquitoes is necessary to allow rigorous monitoring, evaluation and management. While clinical or parasitological indicators are essential for rigorous evaluation of program impact, these are usually collected and reported on timescales too slow to enable day-to-day management for optimal performance.
**Separation of surveillance and treatment responsibilities**	Larvicidal treatment, monitoring and evaluation activities should each be implemented by distinct groups of personnel so that competing interests in data collection and interpretation are minimized [5, 14, 17]
**Integration with existing infrastructure and governance mechanisms**	Larval control programs must be integrated with pre-existing local government structures and public health systems to minimize costs, maximize effectiveness and ensure sustained acceptance by communities, public services and governments [29, 71-73].
**Full time staff**	Larval control program staff must be allocated to the program full time. New responsibilities can not be taken over by established and often overburdened public health staff. Larval control staff will be recruited and managed through existing infrastructure and governance mechanisms as described above.
**Satisfactory evidence must precede scale up.**	Although some encouraging evidence does exist [14-17, 36, 74], strategies targeting aquatic stage mosquitoes, including systematic larviciding remain underdeveloped and have yet to be evaluated on scales that are meaningful for scale-up as priority malaria prevention measures in Africa.

**Figure 2:**
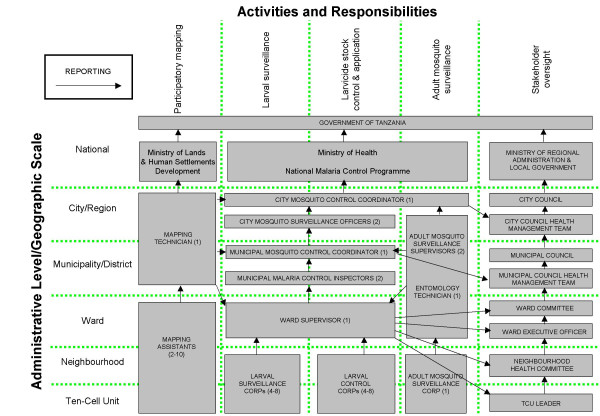
Reporting structure of the UMCP, presented as a matrix of activities which are hierarchically layered over a range of spatial and administrative scales. The numbers presented in brackets describe the number of personnel assigned to each post in each administrative subunit rather than level (e.g. 2 municipal inspectors at each of 3 municipalities means that a total of 6 should be working for the programme at any time).

The reporting structure of the UMCP consists of a matrix of activities which are hierarchically layered over a range of spatial and administrative scales (Figure 2). At each spatial and administrative scale, the programme reports to relevant stakeholders but remains essentially autonomous in terms of day-to-day activities. Importantly, lines of reporting are carefully designed with respect to the guiding principles of Table 2 so that competing interests of staff are minimized with respect to their implementation, support and supervision duties. For example, CORPs responsible for larval surveillance, and those responsible for the application of larvicides, report separately to their ward supervisors. Furthermore, adult mosquito surveillance is implemented by a separate team which primarily reports to the city mosquito control coordinator and secondarily to the three municipal coordinators so that this data reporting line is collected and reported independently of staff responsible for maintaining low vector densities. The implementation of each activity, as well as their integration into a coordinated management system is described in detail below. All data sheets and standard operating procedures were translated in Kiswahili to ease the work of community-based staff.

### Participatory mapping

Although the use of remote sensing techniques for the detection of mosquito breeding habitats have proven useful [39], a large number of *An. gambiae *larval habitats are temporary and appear and disappear frequently in space and time especially in the urban context, which requires constant supervision. Maps of habitats need to be developed and updated on a weekly basis to keep up with the rapidly changing field situation. In this scenario, the use of remotely sensed imagery to accurately monitor habitats demands the analysis of images at multiple times, which is likely to face financial and technical (e.g. cloud coverage) constraints.

**Figure 3:**
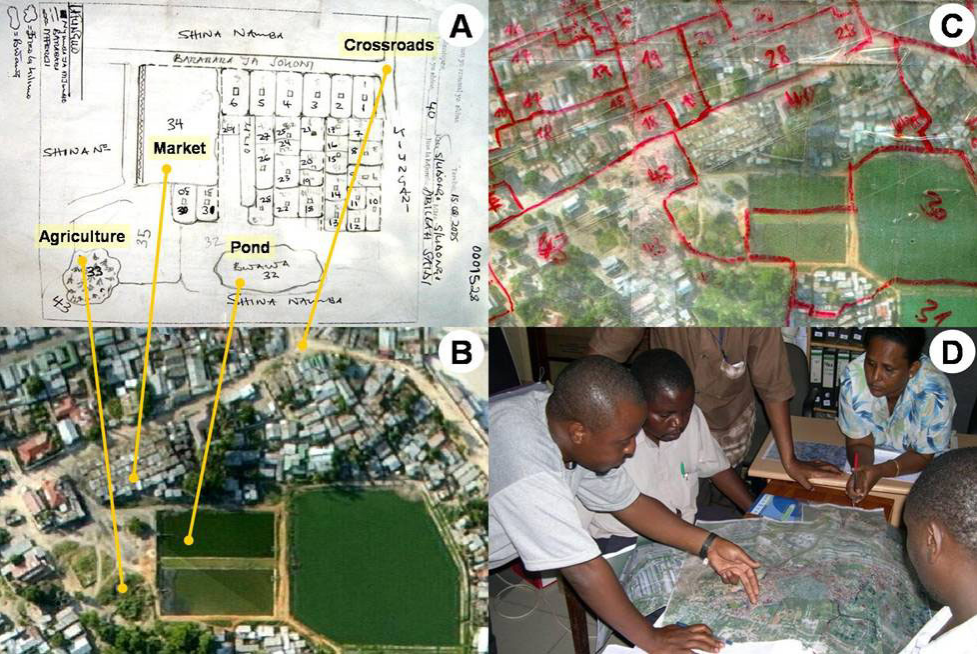
**Example of a sketch map, aerial picture and field map**. **A**. Sketch map of TCU no. 40 in Kurasini ward, Shimo la Udongo neighbourhood, as drawn by the responsible CORP. Features comprise plots with continuous numbering, streets, drains, agricultural areas and ponds. **B**. The same area on an aerial picture. The yellow lines connect identical features on the sketch maps and the aerial picture. **C**. The same area on the laminated map used in the field. The features to be mapped (TCU boundaries and numbers) were marked with non-permanent red marker pens. **D**. Project management team discussing over the field map of a whole ward, and deciding on necessary follow-up actions. Reproduced from Dongus et al. 2007 [28].

Before any surveillance or control activities can be successfully implemented, the boundaries of all targeted areas must be mapped thoroughly in a way that is useful to both the highest levels of city management and the community-based staff responsible for executing most of the programme's activities. A simple community-based mapping procedure that requires no electronic devices in the field was, therefore, developed [28], which formalizes ground-based sketch maps using laminated aerial photographs in the field and then digitizes them using Geographical Information Systems (Figure 3). Initial estimates from the first three wards mapped indicated that over 30% of the study area had not been included in the first round of sketch mapping by larval surveillance CORPs, mostly because they were non-residential or industrial areas that do not exist on local government residential lists [28]. This procedure, described in detail elsewhere [28], allows rapid identification and inclusion of these key areas for sketch mapping and routine mosquito control, as well as more equal distribution of work to field staff.

A key feature of this mapping procedure is that it allows every square meter of the study area to be assigned to a specific geographic unit known as a Ten Cell Unit (TCU) and a specific subunit within that TCU referred to as a plot [28]. This in turn allows each of the constituent TCUs in each ward and neighbourhood to be assigned to specific individual CORPs for weekly larval surveillance and larvicide application. Crucially, plots are small enough to allow unambiguous description of individual habitats by CORPs and subsequent identification by supervisory staff in the field. This can be achieved by using a larval habitat surveillance form in conjunction with the corresponding TCU sketch map and plot description form [see Additional file 1]. This mapping procedure provides an essential frame of reference for weekly routine mosquito surveillance and insecticide application, as well as the supervision of these activities by management staff.

### Surveillance of potential *Anopheles *habitats

All essential standard operating procedures, posters and forms for adapting and reproducing the larval surveillance systems described below are available as an online supplement [see Additional files 2, 3, 4, 5, 6]. Approximately 90 larval surveillance CORPs were employed at any given time during the study and these were each assigned defined areas based initially on local knowledge of habitat abundance, difficulty of terrain and geographic scale of their own neighbourhoods. This workload was subsequently redistributed following detailed participatory mapping [28]. In general, CORPs were recruited through local administrative leaders known as street chairmen and received minimal emoluments (Tanzanian Shillings (TShs) 3,000/day or US$ 2.45/day) as volunteer workers through a system developed by the municipal councils of Dar es Salaam for sundry small-scale maintenance tasks such as road cleaning [24,29]. All CORPs are assigned to a single neighbourhood or subset of TCUs from that neighbourhood [28] under the oversight of a single supervisor for the entire ward. CORPs follow predefined schedules of TCUs which they are expected to survey each day of the week, collecting forms from their ward supervisor at the Ward Executive Office each morning and returning them each afternoon. The return of forms each afternoon is normally used to discuss the day's observations so that the supervisor can follow these up in a timely manner. The schedule of TCUs visited by surveillance CORPs follows one day after the application of microbial insecticides so that indicators of operational shortcoming, such as the presence of late-stage (3^rd ^or 4^th ^instar) mosquito larvae, can be reacted to in sufficient time to prevent unwanted emergence of adult mosquitoes.

Every potential *Anopheles *habitat found in each plot is described by using a standardized form [see Additional file 5] and classified as one of the following habitat types: 1: Puddles & tyre tracks, 2: Swampy areas, 3: Mangrove swamps/Saltwater marshes 4: Drains/Ditches, 5: Construction pits/foundations/man-made holes, 6: Water storage containers, 7: Rice paddies, 8: Ridge and furrow agriculture known as *Matuta*, 9: Habitats associated with other agriculture, 10: Streams/river beds, 11: Ponds, 12: Others [see Additional files 2, 3, 4, 5, 6]. It is important to note that once a habitat is identified and assigned a habitat identification number, that number is retained for all subsequent rounds of surveillance so that a) the identity of those habitats can be unambiguously allocated and followed up in the field and b) the dynamics of larval populations in habitats of different types and characteristics can be assessed. Thus, when habitats contain no water, they are still recorded but described as being dry. The presence of mosquito larvae and pupae are determined by dipping potential breeding sites [40]. Up to 10 dips are taken with a white 350 ml dipper. Anopheline and culicine larvae are differentiated macroscopically in the dipper according to whether they float parallel with the water surface (anophelines) or hang down from the surface (culicines) [41]. No further differentiation to species level is attempted. Records on presence or absence are taken for both genera separately. If larvae are present the sizes of the larvae are observed and classified as early (1^st ^and 2^nd ^instars) or/and late (3^rd ^and 4^th ^instars) stages. Morphological differentiation of pupae from different genera is very difficult and impracticable under field conditions in an operational malaria control programme implemented by staff with basic training [23,37]. Pupae are, therefore, not differentiated between *Anopheles *and other genera. The approximate size, depth and associated vegetation for each habitat are also recorded [see Additional file 5].

**Figure 4:**
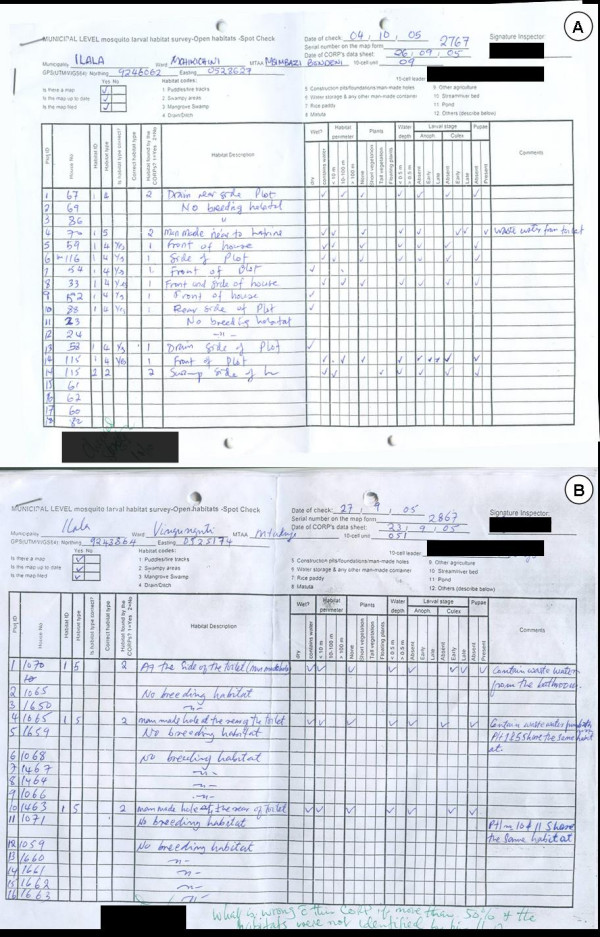
Examples of spot-checking forms [see Additional file 5] for Municipal Mosquito Control Inspectors. **A**. A typical example signed on the bottom left by a City Mosquito Surveillance Officer to show it has been checked for consistency and signs of problems requiring corrective action by management at city, municipal and ward level. **B**. An example of where an inspector has found poor coverage of potential habitats for *Anopheles *larvae by a CORP but failed to highlight it or record any corrective action. Note the query of the City Mosquito Surveillance Officer at the bottom.

The characteristics of the CORPs forms are also captured in the corresponding forms used by Municipal Mosquito Control Inspectors (MMCIs) who assure quality control of CORPs work independently of their ward supervisors (Figure 4). All MMCIs conduct weekly spot checks of six randomly assigned TCUs in their municipality, assessing the accuracy of the data collected by the CORP through direct on-the-spot observation. Spot checking of six TCUs takes approximately two days per week allowing enough time for the implementation of other duties e.g. supervision of data collection and training activities nevertheless ensuring that each larval survey CORP is visited at least once every two months. Additional larval habitats identified by the MMCI that had not been detected by the CORPs are recorded and additional clear discrepancies between the records of the CORPs and the observations of the inspector documented. It should be noted that although the observations of the inspectors are shared with the respective ward supervisors, they are primarily reported to the Municipal Mosquito Control Coordinator who takes responsibility for managing the Ward Supervisors.

### Larvicide application and stock management

After one year of baseline data collection on habitat and larval seasonality and adult abundance the UMCP staff reviewed the performance of larval surveillance CORPs and Ward Supervisors for all 15 wards in order to select one ward from each municipality for larval control interventions in the following year. The research team based the decision of which wards will receive larviciding and which wards will be compared with the intervention wards mainly on the proven ability of the ward supervisors and ward-based CORPs to implement the required task. Specifically, their ability to collect, understand, use and submit high quality data during the baseline data collection period was the primary criterion for choosing these high priority wards. Since the success of larval control interventions largely depends on good management skills and supervision, the UMCP team selected the best performing wards for the evaluation of the first year's intervention, whilst also striving to improve the performance of the remaining wards. One ward from each municipality, namely Buguruni, Mikocheni and Kurasini, were chosen for larviciding. In an attempt to facilitate representative comparison and analysis, one non-intervention ward from each municipality, namely Vingunguti, Mwananyamala and Keko, were selected *a priori *on the same basis as the intervention wards. Along with the intervention wards, these non-intervention wards were targeted for particularly rigorous maintenance of larval surveillance standards so that valid evaluations of larvicide impact upon larval populations could be made. This choice of a limited number of controls (non-intervention wards) was considered essential to ensure that the laboriously-collected larval data from both, intervention and non-intervention areas, were similar in terms of their extent and intensity for the first year's evaluation. In parallel, all remaining wards were subsequently evaluated and targeted for re-training activities or staff replacement, so that by the end of March 2007 all wards showed comparable performance.

Larviciding is implemented exclusively with microbial insecticides, specifically *Bacillus thuringiensis *var. *israelensis *strain AM65-52 (*Bti*; VectoBac^® ^Valent BioSciences Corporation, VBC, USA) and *Bacillus sphaericus *strain 2362 (*Bs*; VectoLex^®^, VBC, USA) because they are 1) highly efficacious against African malaria vectors, 2) selective in action, 3) environmentally safe to non-target organisms, 4) unlikely to result in resistance when used in combination or when only *Bti *is used, 5) safe for human handling and consumption, 6) easy to handle by staff with minimal training and protective measures, and 7) their impact can be easily monitored [35,36,41-44]. *Bti *is efficacious in all types of habitats but is less potent in high concentrations of organic matter, such as open sewers, and closed habitats, such as pit latrines and septic tanks. *Bti *needs to be applied weekly, but is relatively cheap compared with *Bs *[36]. Nevertheless, *Bs *has the advantage of being efficacious in very polluted water and even recycling by propagating itself in the cadavers of the mosquito larvae it kills [45-51]. Although *Bs *can have a residual effect and may not require weekly application, its efficacy in open habitats is difficult to predict. Furthermore, the habitat monitoring requirements to enable timely re-application and the decision making process necessary to decide when and where to apply a larvicide with residual effect might be a source for errors. Therefore, the application of *Bs *was not considered appropriate for the start of a programme. Moreover, *Bs *formulations are about three times more expensive than *Bti *formulations [36] and need to be applied in higher dosages to produce a persistent residual effect [35] which is likely to be less cost-effective than labour intensive treatment with *Bti *[52]. In closed habitats which are not exposed to solar radiation and support densities of culicine mosquitoes that are high enough to enable sustained recycling, a single treatment with a sufficient dosage of *Bs *can be reliably expected to suppress emergence for several weeks [51,53-55].

Two formulations of larvicides are used in the programme: water-dispersible granules (WDG) are applied as aqueous suspensions using Solo^® ^475 knapsack sprayers, whereas corn granules (CG) are applied by hand. CG was preferred for the vast majority of habitats that are open to the sun. Although hand application of CG treats large areas less rapidly and less evenly than WDG, it is broadly applicable under different environmental conditions. Moreover, it can be readily applied by community-based personnel with minimum training. Granules can penetrate vegetation to reach targeted water surfaces and can be distributed further than liquid aerosols, thereby allowing treatment of less accessible sites. CG was also preferred for treating closed habitats because it is easy to apply to such domestic mosquito sources by CORPs and even the house owners. Liquid application of WDG with knapsack sprayers was preferred for extensive areas of stagnant water with little emergent or floating vegetation that might prevent the sprayed aerosol from reaching the water surface.

Based on evaluations of *Bti *and *Bs *in western Kenya [35,36], the formulations-dosage combinations described in Table 3 were recommended for larval control, although in practice these dosages were often accidentally exceeded especially by inexperienced staff and in very small habitats. Training materials and detailed guidelines for insecticide application, based on locally implemented calibration exercises, were prepared in a participatory manner and refined through early field piloting [see Additional files 7 and 8]. While open habitats with the potential to produce *Anopheles *are treated weekly by Mosquito Control CORPs assigned to neighbourhoods or portions of neighbourhoods, closed habitats are treated every three months by small teams of additional CORPs working through entire wards on a quarterly cycle.

**Table 3:** Formulation-dosage combinations recommended to UMCP staff to achieve 100% control of mosquito larvae within 24 hours.

Product^a^	Active	Dosage		Application
			
	Ingredient^b^	kg/hectare	g/m^2^	Cycle
				
*Open habitats *^c^				
VectoLex^® ^WDG (650 ITU/mg)	Bs	2.0	0.20	1 week
VectoBac^® ^WDG (3000 ITU/mg)	Bti	0.4	0.04	1 week
VectoLex^® ^CG (50 ITU/mg)	Bs	30	3	1 week
VectoBac^® ^CG (200 ITU/mg)	Bti	10	1	1 week
				
*Closed habitats *^c^				
VectoLex^® ^CG (50 ITU/mg)	Bs	10	1	3 months

^a ^ITU = International Toxic Units, describes the potency of larvicide, the higher the number, the more toxic is 1 mg the less is needed to kill 100% of larvae within 24 hrs

^b ^*Bti*; *Bacillus thuringiensis *var *israelensis*, *Bs*; *Bacillus sphaericus*

^c ^See box 1 for definitions.

The specificity of these microbial insecticides makes stock control substantially easier because they do not have any uses, other than mosquito control, which avoids financial incentive for theft, misuse or misappropriation. Nevertheless, insecticide stocks are carefully managed at a central storage site and distributed to locked cabinets in each ward office on a weekly basis. Insecticide stocks are distributed on a 'first-in, first-out' basis and decentralized stocks at the ward level are replenished weekly on the basis of consumption and projected need. Simple, but readily verifiable records of the daily use of insecticide by each individual CORP allows decentralized detection and correction of inappropriate use rates by Ward Supervisors and other management personnel [see Additional file 7] in a manner similar to programmes for indoor residual spraying of chemical insecticides in southern Africa [56]. Consumption rates at the ward level can also be reconciled with city level records at the central storage and delivery facility. These central stock management procedures also allow timely ordering of new stock which is currently sourced from the USA and therefore entails a delay of at least two months between ordering and delivery by surface freight.

### Adult mosquito surveillance

It was originally planned that the CORPs would also report densities of adult mosquitoes at sentinel sites distributed throughout the study area using Mbita-design bed net traps [57-59]. However, 181 full night samples with these traps executed over two months yielded over 4,000 *Culex*, *Mansonia *and *Aedes *of various species, but only one *An. gambiae sensu lato *caught in one of the traps placed outdoors. While the very low sensitivity of Mbita traps is consistent with other reports [60], additional observations suggest a broader limitation to existing trapping methods for malaria vectors in Dar es Salaam. Further investigation showed that CDC light traps beside occupied bed nets, indoor pyrethrum spray catch and Mbita bed net traps all failed to catch significant numbers of *Anopheles *indoors in Dar es Salaam, while three nights of outdoor human landing catch at one location yielded 136 *An. gambiae s.l*., 30 other *Anopheles *and 806 culicines, two nearby Mbita traps (one placed indoor and another outdoor) caught only 176 culicines and no *Anopheles *on the same nights. Two nearby CDC-light traps placed beside occupied untreated bed nets (one indoors and one outdoors), which is normally a reliable trapping method for malaria vectors in sub-Saharan Africa [58], captured 423 culicines, but only three *An. gambiae s.l*. and 14 other *Anopheles*. Notably, all *An. gambiae s.l*. caught in light traps were found in traps placed outdoors and it has since been shown, through detailed behavioural studies, that *An. gambiae *and *An. arabiensis *are both predominantly exophagic in this highly urbanized environment [26]. The inability of CDC light traps and pyrethrum spray knockdown to capture *An. gambiae s.l*. in modern Dar es Salaam contrasts with previous programmes up to 1996, suggesting that this behavioural shift is a relatively recent adaptation, possibly resulting from increased bed net use and house screening.

This unexpected difficulty in monitoring adult mosquitoes was overcome by conducting human landing catches as an interim monitoring and evaluation measure while alternative trapping technologies were developed to replace it. Detailed protocols and training materials for the adult mosquito surveillance procedures are not provided for adaptation elsewhere because this cannot be considered a routine procedure for wide-scale programmatic use. The potential health risks associated with human landing catches necessitate careful consideration, justification and ethical review. The human landing catches executed in these early stages of the Dar es Salaam UMCP are undertaken as an interim research tool only. Practical, safe and effective new surveillance procedures have since been developed to prototype stage and will be reported elsewhere after full evaluation in terms of efficacy and effectiveness (NJ Govella, personal communication).

The procedures applied to monitor and evaluate mosquito densities [26] are described briefly as follows. One resident was recruited from each of the 67 neighbourhoods in the study area and employed as an Adult Mosquito Surveillance CORP to conduct one full night of human landing catch each week. All human landing catches are done outdoors. Each CORP is assigned four sampling sites which are distributed approximately evenly across his neighbourhood. For safety reasons, these are typically within walled compounds but are nevertheless chosen on the basis of not only the location, but also the co-operation of the residents and accessibility of the site to city-level supervisors for unannounced spot checks. Once every four weeks at each location, human landing catch are conducted from 6 pm to 6 am for 45 minutes of each hour, allowing 15 minute breaks for rest. Each afternoon a city level team led by two Adult Mosquito Control Supervisors distributes a kit to each CORP scheduled to work that night. The kit consists of netting-covered cups for each hour's catch, an aspirator and a simple form upon which each hour's catch can be recorded so that, upon random inspection at any hour of the night, the recordings and content of the cup can be reconciled. Each morning the kits, with all caught mosquitoes in their respective cups, are collected and returned to a central laboratory. All collected mosquitoes are identified morphologically to genus and, in the case of *Anopheles*, to species complex level [61]. Members of the *An. gambiae *species complex are further resolved to sibling species level by polymerase chain reaction [62]. The sporozoite infection status of each mosquito gets determined by enzyme-linked immuno-absorbent assay [63].

### Integration and coordination

**Figure 5:**
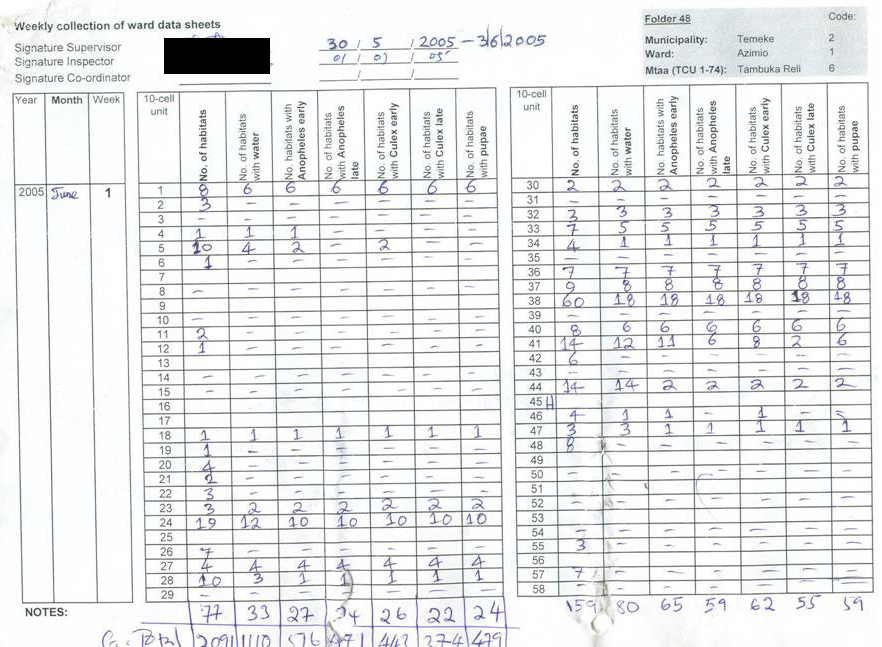
Example of a completed weekly ward summary form [see Additional file 9] filled out by the Ward Supervisor and totalled along the bottom with a pocket calculator to enable rapid entry into monthly report templates at the municipal level.

Larval surveillance data are primarily summarized and interpreted at the level of the Ward Supervisors to enable the rapid response of larvicide application to observed operational failures. This is accomplished in a practical, affordable and scaleable manner using weekly summary forms [see Additional file 9], which are filled out each afternoon by the supervisor when the Larval Surveillance CORPs under his/her oversight return the filled forms from their work that morning. The total number of habitats and the subset of those which contain water and mosquito larvae of various stages are totalled from each form (and the TCU it represents) provided by the CORPs by simply counting the number of ticks in each column (see Figure 4 which closely resembles the equivalent form for CORPs). These totals are then entered in the supervisor's weekly summary sheet, inspected immediately for signs of poor larvicide application, and totalled for each neighbourhood when all its TCUs have been completed (Figure 5). Supervisors are expected to note any such indicators of programme failure and consequent follow-up action on these forms, signing and dating all such notes, as well as the confirmation that they have read and checked the form before filing. This approach formalizes the obligation to read and respond to all larval surveillance data within 24 hours, and allows unambiguous assessment of performance and responsibility by municipal and city-level management. Furthermore, it simplifies, accelerates and decentralizes an otherwise vast data aggregation burden without using any computing technology beyond that provided by a pocket calculator.

**Figure 6:**
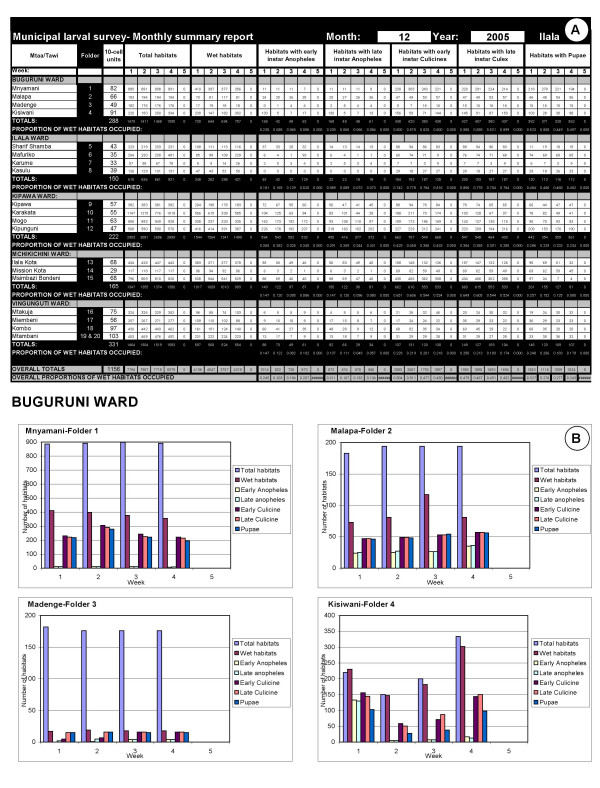
Example of a mosquito larval surveillance component in a municipal monthly report template. **A**. The overall data entry table in which each row corresponds to one, or occasionally two (see bottom row for example of a very large neighbourhood) folders, each containing 4 or 5 sequential weekly ward summary forms and respective sets of CORPs larval surveillance forms. Note that weeks overlapping two months are assigned to specific calendar months in advance so that each operational month has a predefined start and end date, spanning exactly 4 or 5 weeks. **B**. A typical automatically generated chart summarizing the observed distribution of larval habitat abundance and mosquito occupancy in one ward.

**Figure 7:**
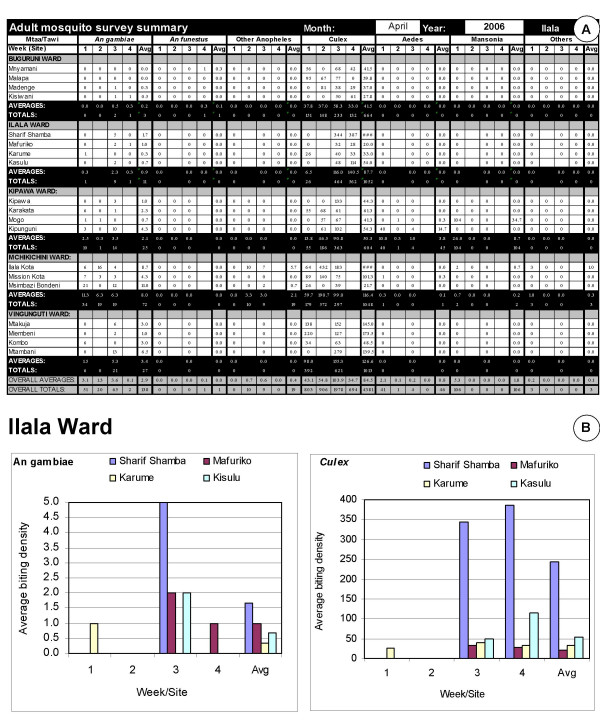
Example of a mosquito adult surveillance component in a municipal monthly report template. **A**. The overall data entry table (empty fields indicate missing data) **B**. A typical automatically generated chart summarizing the observed distribution of adult mosquitoes.

All the Larval Surveillance CORPs' forms are collated in order of their TCU numbers in pre-labelled folders with the ward supervisor's summary sheet on top of the cluster of TCUs it summarizes. These folders are provided to the Municipal Mosquito Control Coordinator (MMCC) each week. The MMCC or the MMCIs directly under his/her supervision then checks that all forms have been filled out and submitted correctly, recording the results of this quality control exercise in a checklist designed for that purpose [see Additional file 9]. The totals for each neighbourhood in this checklist, at the bottom of each ward supervisor's summary form (Figure 5), are then entered into a password protected excel spreadsheet template, tailored to each municipality. This template automatically generates summary statistics, tables and charts [see Figures 6 and 7] that form the backbone of the MMCCs monthly report to the City Mosquito Control Coordinator (CMCC). More importantly, the MMCC is responsible for identifying and reacting to signs of programme failure in the content of these forms within a week of their occurrence, documenting any actions taken in writing on those forms. These standard, automatically generated tables and charts are supplemented with written narratives summarizing successes, failures and responses to these monthly observations, as well as plans and requests for support to implement further action. A crucial part of the MMCCs duties is to coordinate, assess and execute corrective action in relation to the observations of his/her inspectors when conducting random spot checks to assure the quality of data reported by larval surveillance CORPs (Figure 4). The results of these quality control assessments by the MMCIs are also entered into the municipal monthly report template for examination by the CMCC and his/her two City Mosquito Surveillance Officers (CMSOs). The MMCC also receives a summary of the adult mosquito surveillance data for that week directly from the city-level Adult Mosquito Surveillance Supervisors so that this independent and more direct assessment of programme impact can be used to rigorously triangulate and interpret the larval surveillance data. This data are also included in the monthly municipal report with a preformatted component of the spreadsheet which automatically generates summary statistics and charts.

**Figure 8:**
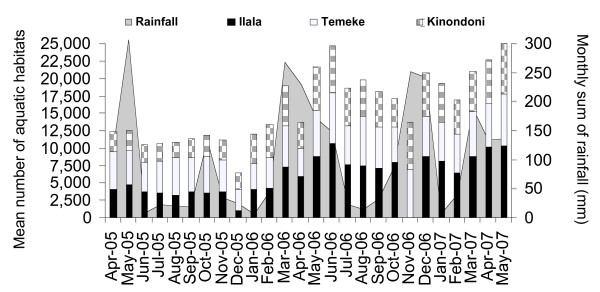
Monthly average of aquatic habitats surveyed in the three municipalities Kinondoni, Ilala and Temeke from February 2005 to March 2007 in relation to rainfall.

**Figure 9:**
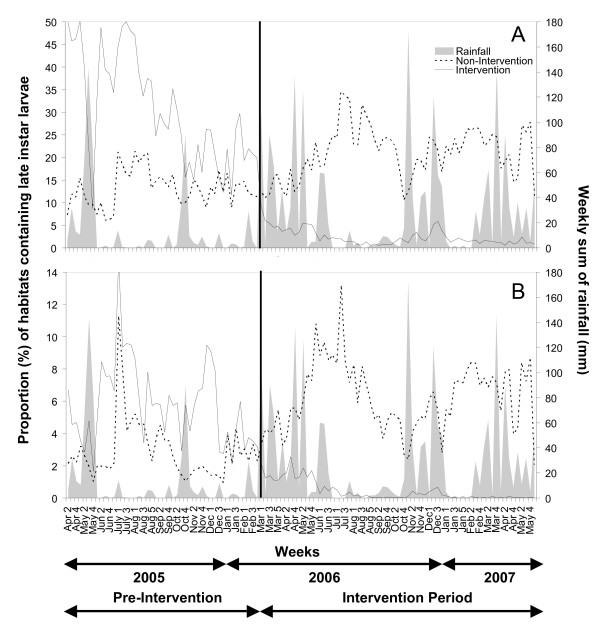
Impact of seasonal rainfall variation and larvicide application on aquatic-stage mosquito populations between April 2005 and June 2007. Larvicide application started in the intervention sites in March 2006 week number 1. **A**: Proportion of aquatic habitats containing late instar culicine larvae at weekly surveys. **B: **Proportion of aquatic habitats containing late instar anopheline larvae at weekly surveys.

**Figure 10:**
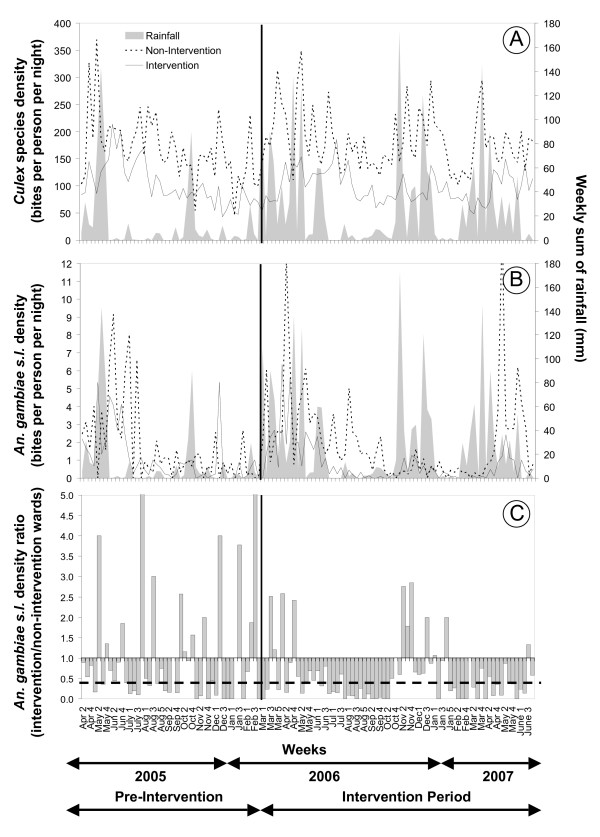
Impact of seasonal rainfall variation and larvicide application on weekly adult mosquito densities between April 2005 and June 2007. **A**. Rainfall and densities of adult *Culex *species, **B**. Rainfall and densities of adult *Anopheles gambiae s.l*., **C**. The ratio of densities of *An. gambiae s.l*. in intervention wards relative to non-intervention wards. The line representing the x-axis in panel **C **represents equivalence of densities in intervention and *a priori *selected non-intervention wards while the vertical black line represents the initiation of larviciding activities. The thick, broken horizontal line in panel **C **represents the ratio of exposure estimated to be provided by an insecticide-treated net in urban Dar es Salaam [26].

The City Mosquito Control Coordinator (CMCC) expects to receive the previous month's municipal reports in the first week of each month and is expected to provide verbal feedback, as well as annotated comments, on these reports in a meeting with the CMSOs, MMCCs and MMCIs to be held on or before the end of the second week of the month. The CMCC collates these data and adds them to existing records to generate a series of trend graphs and summary statistics that quantify and illustrate the progress of the programme in terms of impact on larval (Figure 8 and 9) and adult-stage mosquitoes (Figure 10). By the start of 2007, the CMCC had begun presenting these reports at bimonthly coordination meetings with the partners of the primary donor for the programme at that time (US President's Malaria Initiative of the United States Agency for International Development).

### Analyses

To describe changes in mosquito densities associated with larviciding the percentage reduction in mosquito densities in larviciding areas was calculated using an established formula [35,42,64]which takes into account that natural changes (for instance through predation or changes in climatic conditions) in the mosquito populations are taking place over time at the same level and rate in both treated (intervention) and untreated (non-intervention) sites. Therefore, the percentage reduction is defined as follows:

% reduction = 100 - (C_1_/T_1 _× T_2_/C_2_) × 100

where C_1 _and C_2 _describe the average density of mosquitoes in untreated (non-intervention) sites during baseline and intervention periods, and T_1 _and T_2 _describe the average density of mosquitoes in intervention sites during baseline (when no larviciding took place yet) and intervention periods (when larvicides were applied weekly) [64]. All figures presented as "percentage reduction" throughout the paper have been calculated using this formula.

All measured adult mosquito biting densities were multiplied by 1/0.75 to get biting rates for a full hour [26]. Generalized estimating equations (GEE) were run with SPSS 15.0 to calculate differences in mosquito biting rates and EIR between intervention and non-intervention areas with ten-cell units as a subject unit, log linked mosquito densities and intervention and non-intervention areas as the factor (Table 4). In order to adjust for total exposure indoors and outdoors, outdoor mosquito densities were multiplied by the ratio of the total true human exposure (the sum of the hourly mean of the indoor and outdoor biting rates, weighted according the proportion of time human beings typically spend in these two compartments) divided by the total outdoor biting rate as estimated previously [26]. These ratios were derived from an in depth mosquito survey which was conducted during the main rainy season in 2006 (*An. gambiae*: 0.67, *An. funestus*: 0.725, *Anopheles coustani*: 0.448 and *Culex*: 0.94) [26].

**Table 4:** Comparison of mean human biting rates (HBR) of *An. gambiae s.l. and Culex sp*. and entomological inoculation rate (EIR) for *An. gambiae s.l*. in the intervention and non-intervention wards during baseline and first year of intervention. 95% confidence intervals in parenthesis.

	**Pre-Intervention**^a^			**First intervention year **^b^			**Percentage Reduction**
			
	**Non-Intervention Wards**	**Intervention Wards**	***p***^c^	**Non-Intervention Wards**	**Intervention Wards**	***p***	
**Annual mean**							
Daily HBR *An. gambiae*	0.93 (0.60–1.46)	0.72 (0.51–1.02)	0.367	0.94 (0.57–1.56)	0.50 (0.38–0.68)	0.040	31.3%
Annual EIR *An. gambiae*	1.05 (0.68–1.65)	0.81 (0.58–1.15)		1.06 (0.64–1.77)	0.56 (0.43–0.77)		
Daily HBR *Culex sp*.	173.9 (140.7–214.9)	86.8 (72.7–103.7)	<0.001	171.5 (137.2–214.3)	86.1 (70.9–104.4)	<0.001	0%
**Dry season mean (July-August-September)**							
Daily HBR *An. gambiae*	0.59 (0.32–1.11)	0.46 (0.29–0.72)	0.505	1.17 (0.56–2.47)	0.12 (0.08–0.20)	<0.001	86.8%
EIR *An. gambiae*	0.67 (0.36–1.26)	0.52 (0.33–0.81)		1.32 (0.63–2.79)	0.14 (0.09–0.22)		
Daily HBR *Culex sp*.	196.3 (157.9–244.0)	98.4 (82.2–117.9)	<0.001	151.1 (125.3–192.0)	86.1 (67.1–110.6)	<0.001	0%

^a ^April 2005 – March 2006; March 2006 has been included in the calculation for the baseline year since reductions of adult mosquitoes due to larviciding cannot be expected earlier than 3–4 weeks into the intervention [36].

^b ^April 2006 – March 2007.

^c ^Generalized estimating equations (GEE) were used to analyse pre-intervention data and data from the first year of intervention, respectively. In each analyses mean densities are compared between non-intervention and intervention sites. Ten-cell units were used as a subject unit, log linked mosquito densities and intervention and non-intervention areas as the factor.

### Ethics

All participants provided informed consent. No persons in high risk groups, namely people under 18 years or women of reproductive age, were recruited to conduct human landing catches. Furthermore, the catchers are screened every week for malaria by microscopic examination of thick smear peripheral blood samples and treated with artemisinin-based combination therapy when diagnosis was positive. Research clearance was obtained from the Medical Research Coordination Committee of the National Institute of Medical Research in Tanzania (NIMR/HQ/R.8a/Vol. IX/279) the Tanzanian Commission of Science and Technology (No. 2004-69-MFS-2004-24) and Durham University's Ethics Advisory Committee (No. 03 EAC R131).

## Results

Overall, the vector surveillance and management systems developed in Dar es Salaam allowed timely collection, interpretation and reaction to field-collected entomologic data with reaction times at ward, municipal and city levels of one day, week and month, respectively. In fact, the vector density patterns as presented in Figure 9 and 10 were drafted into manuscript format figures within three weeks of their collection through these standard low-technology procedures, therefore serving as an instant monitoring and teaching tool. In contrast, more complex, research driven analyses (Table 4), which require elaborate data entry procedures, can only be achieved with several months delay.

**Figure 11:**
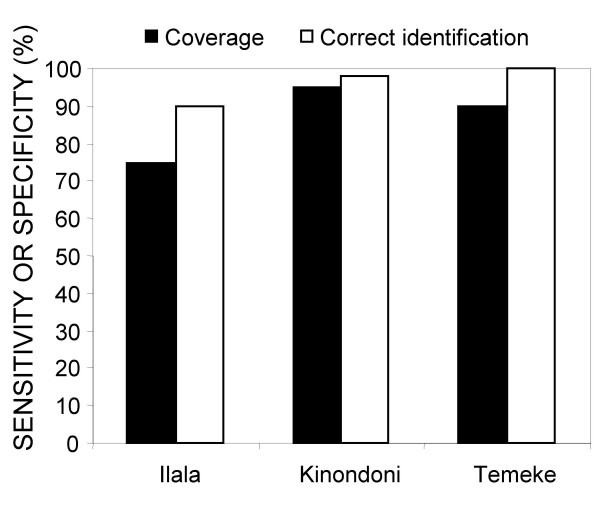
Proportion of habitats successfully detected (sensitivity) and correctly identified (specificity) by larval surveillance CORPs in November 2005, as determined from the random on-site spot checks of the Municipal Mosquito Control Inspectors using methodology essentially identical to earlier evaluations of larval surveillance [24].

The implementation of the programme through local community-based staff led to high community acceptance and support. The procedures and staff management systems described, greatly improved standards of larval surveillance relative to that reported at the outset of this programme [24]. Vanek and others [24] reported that only 42% of potential *Anopheles *habitats were detected by CORPs prior to the introduction of the programme management systems described here. By the end of 2005, the independent spot checks of the Municipal Mosquito Control Inspectors revealed that all three municipalities had larval surveillance coverage levels exceeding 75% (Figure 11). Based on this result the decision was taken to implement larviciding in three selected wards since substantial reductions of malaria exposure and burden for resident populations [10-12] were expected if such coverage levels could be approached with actual larvicidal control.

Larviciding began in three wards in the first week of March 2006 (Figure 1). By that time more than 65,000 potential *Anopheles *habitats spread out over a 55 km^2 ^area occupied by more than 612,000 people were surveyed on a weekly basis. At any sampling date, between 10 and 50% of all habitats contained water (Figure 8).

The first year of larviciding successfully reduced the number of larval habitats (Figure 9). In the three non-intervention wards the proportion of habitats that contained late instar anopheline and culicine larvae increased from March 2006 onwards by an average of 53% and 37%, respectively, as compared to the baseline year. This is probably associated with more rainfall in 2006 (1526 mm) compared to 2005 (979 mm) leading to an increase in fresh water and suitable habitats (Figure 8). In marked contrast, the number of habitats with anophelines and culicines both fell in average by over 90% in the three intervention wards as compared to the baseline year. Overall percentage reduction in *Anopheles *larval habitat abundance was 96.5% assuming that without larviciding larval populations would have risen by the same rate as in non-intervention wards [64].

The vast majority of 245,927 adult mosquitoes collected in the year before intervention were culicines represented by *Culex *sp. (97.7%), *Mansonia *sp. (0.9%) and *Aedes *sp. (0.4%). Only 1% (2,468) of these were anophelines. *An. gambiae s.l*. represented 76.6% (1,864) of the anophelines and was by far the most common vector. Only a small number of *An. funestus *(85; 3.5%) were identified through the adult surveillance system. Laboratory analyses confirmed transmission by both *An. gambiae s.l*. and *An. funestus *with sporozoite rates of 0.31% and 1.25% ([65], respectively. A sub-sample of 1,099 members of the *An. gambiae *species complex were identified as 75.6% *An. gambiae s.s*., 21.3% *An. arabiensis *and 3.1% *An. merus*.

Culicine mosquitoes were abundant in all study wards and showed little seasonality throughout the year (Figure 10A). During the baseline data collection the average culicine human biting rate was nearly twice as high in the wards chosen *a priori *as controls for the intervention and this proportion did not change during the intervention (Table 4) indicating that routine larvicide application did not suppress the nuisance biting rate.

Adult densities of the primary vector, *An. gambiae s.l*., were highly seasonal (Figure 10B). Although the mean *An. gambiae s.l*. human biting rate and annual EIR was higher in the control wards than the intervention wards during the baseline year, this difference was not significant (Table 4). In contrast, in the first 12 months of intervention, the mean human biting rate and annual EIR remained approximately the same in the non-intervention wards (Table 4) but decreased by one third in wards where larval control was implemented following the general trend observed in the larval surveys. The difference in transmission intensity between non-intervention and intervention wards was significant (p = 0.04) in the first year of larval control (Table 4) even though an overall percentage reduction of 31.3% might appear modest compared to the impact shown on larval habitat abundance. Notably, the dry season larviciding in July-August-September 2006 led to a percentage reduction in transmission by 87% when compared with the same time period pre-intervention and non-intervention sites. In marked contrast to the pre-intervention year, weekly mean adult mosquito densities in intervention areas were constantly lower than those in non-intervention areas for six consecutive months from May to October 2006, and for five consecutive months from mid January to mid June 2007 (Figure 10C). However, little to no effect was achieved during the primary (March-June) and secondary (October-December) rainy seasons in 2006 (Figure 10B). Larviciding was only begun with the onset of the main rains of 2006 and it took several weeks for programme staff to refine their performance based on hands-on experience. Although the proportion of habitats containing late instar larvae decreased from the start of larviciding, it is important to note that the actual numbers of habitats available increased substantially in March 2006 (Figure 8), resulting in significant larval development and possibly emergence. Thus, although adult *An. gambiae s.l*. densities in intervention wards steadily dropped till the end of September 2006 (Figure 10), the introduction of the intervention came too late to prevent the bulk of transmission resulting from the main rains from March to May 2006.

**Figure 12:**
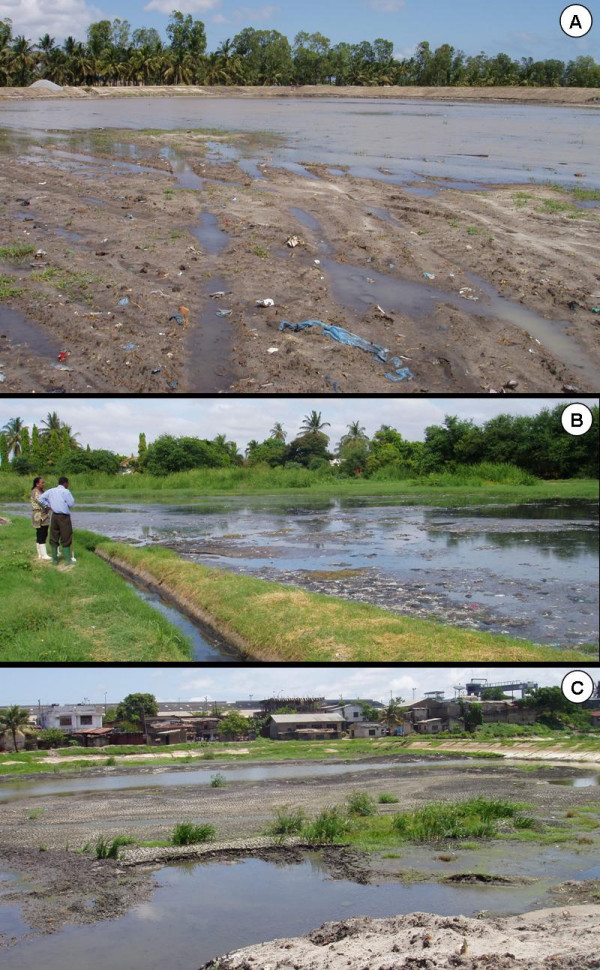
Examples of inaccessible but productive *Anopheles *aquatic habitats in the wards of Buguruni (**A**), Mikocheni (**B**) and Kurasini (**C**) during the period October to December 2006. Note that all the open soil surfaces depicted are in fact very soft mud which is impossible to walk across. Although these ponds had been freshly drained for maintenance, their low porosity, and the rainfall which immediately followed their exposure, resulted in abundant and stable surface water in multiple inaccessible depressions on the surface for two months. These areas closely resemble similarly challenging sites in flooding river valleys of West Africa which can be rigorously controlled with powered granule-blowing equipment [42].

An additional challenge confronted the programme staff during the short rains at the end of 2006. Simultaneous rains and municipal maintenance of waste water settlement ponds in each of the intervention wards generated substantial areas of inaccessible larval habitats ideal for *An. gambiae s.l*. on the surface of freshly drained mud flats (Figure 12). Crucially, these three water treatment facilities were located within 100 meters of at least one adult mosquito surveillance site each so their influence upon recorded *An. gambiae s.l*. densities was substantial. Once these ponds had been fully renovated and these areas either dried out or were filled up, malaria vector densities were once again successfully controlled. Nevertheless, because of programme limitations during both seasonal rainfall peaks in 2006, the overall impact on malaria transmission for the first intervention year was very modest. Preliminary results from the main rains (April-June) in 2007 (Figure 10) though indicate an improvement in the operational procedures which led to a percentage reduction of transmission by 71% as compared to the same time period at baseline and by 62% as compared to the start of the intervention in 2006.

## Discussion

After only one year of operational larviciding in Dar es Salaam a clear impact of the intervention on malaria vectors was demonstrated. Overall anopheline larval abundance was reduced by 96% in the intervention wards compared to historical and contemporary controls which consequently resulted in a significant reduction of 31% of malaria transmission by *An. gambiae s.l*.. Furthermore, preliminary analyses of parasitological surveys (Y. Geissbuehler and M.C. De Castro, personal communication). showed that the larviciding was associated with an overall reduction of 40% (p < 0.001) of *P. falciparum *infection prevalence in the study population and that highest impact was achieved during the dry season of 2006. Interestingly, the majority of infected mosquitoes in Dar es Salaam were found during the dry seasons which also coincided with maximum larval control success (Y. Geissbuehler, personal communication).

The control of nuisance mosquitoes remained unsatisfactory. Similar to observations in other urban centres in East Africa, where anti-larval measures for malaria control were implemented [66], the overall culicine densities remained high in the intervention wards which might be explained by the large number of closed habitats like pit latrines, soakage pits, septic tanks and water storage tanks, which were not included in the weekly larvicide applications. The three-month cycle for interventions targeted at closed habitats is probably too long to suppress larval development in these often highly polluted breeding sites. Furthermore, no rigorous system existed for monitoring coverage of these habitats, to which access is often difficult or not possible at all. While targeting the interventions at *Anopheles *breeding sites makes economic sense, it may not be practicable. Culicine mosquitoes are responsible for over 100 bites per exposed person per night in Dar es Salaam. Targeting *Anopheles *habitats only would most likely lead to the withdrawal of the communities' support as has been shown in the past [30-32]. Nevertheless, *Culex *control appears not worth doing unless the numbers can be reduced sufficiently to convince inhabitants that larval control, in general, is a good idea. Therefore, new strategies including the implementation of environmental modifications need to be urgently developed to address the nuisance biting problem in Dar es Salaam.

The UMCP's unique feature is the surveillance and management system described here which proved to be practical and affordable [52] and allowed operational response times to changing ecological and programmatic conditions that were previously unthinkable at this scale. The strong involvement of community-based staff, local capacity building, direct governmental participation and commitment in all phases of the programme, data-driven decision making and hands-on technical and programmatic support from national and international partners constitute a strong basis for future sustainability of control activities and have been pointed out to be important factors for success in malaria control programmes [18-20].

Despite the overall encouraging impact on malaria transmission, the wet season results in 2006 were clearly unsatisfactory. Nevertheless, it needs to be cautioned that adult mosquito sampling was most likely somewhat biased towards overestimating the contribution of the settlement ponds illustrated in Figure 12. Furthermore, detailed spatial analyses of the data need to be carried out to investigate the possibility of immigration of adult mosquitoes from non-treated areas outside the relatively small intervention wards. This might have contributed to the overall modest difference in adult densities between control and intervention wards which stands in sharp contrast to the observations of larval abundance. It is noteworthy, however, that the levels of suppression achieved before and after the short rains in late 2006 comfortably exceeded recent estimates [26] for the personal protection against exposure provided by an insecticide-treated net in this urban setting (Figure 10C).

To achieve effective control, larviciding programmes must clearly suppress transmission not only in the dry season when mosquitoes are most vulnerable but also when their numbers peak during and after the wet season. Both wet seasons in 2006 provided useful lessons and highlight the importance of long-term commitment for successful malaria control with larvicides in urban Africa. The first and most important wet season of 2006 illustrates the importance of being prepared for major transmission surges and the value of hands-on experience. Consistent with our observations of improving staff skills and performance, the impact of larviciding steadily increased following initiation, but the intervention was started too late for improving effectiveness to have a major impact on the intense peak of transmission in 2006.

Much of this can be attributed to the slow financing mechanisms for the programme at that time. All of the financial support for this programme was only secured in mid 2006, with limited interim pre-financing and insecticide donations provided in advance by the research partners at their own risk. These cash flow restrictions meant that equipment, supplies, personnel costs and training could not be assembled and coordinated before this key transmission season, so a vital opportunity to reduce malaria exposure was missed. For most of the programme's existence it has been necessarily pre-financed on an *ad hoc *(and therefore intermittent and unreliable) basis by its primary research partners, without which none of the data or methodologies presented would have been realized. The lack of sustainable funding has been identified as one of the major obstacles in the planning and implementation of mosquito control interventions in general [18,19,67] and a recent evaluation of malaria control programmes in Eritrea, Brazil, India and Vietnam [18] showed that sufficient and flexible financing, decentralized control of resources and local prioritisation of spending was key to success. As of March 2007, one of the research partners of the UMCP has instituted a risk-assessed pre-financing mechanism specifically to support smooth distribution of cash, equipment and supplies to the programme during the slow process of grant allocation and administration from donors. Such credit support from intermediary institutions is, however, likely to be the exception rather than the rule and stable funding mechanisms must be developed if larviciding programmes which rely on continuous weekly application cycles are to be stably implemented and supplied based on long-term development plans.

The unforeseen creation of major, inaccessible larval habitats during the short rainy season at the end of 2006 underlines the importance of experience and long-term commitment to programmes which rely so much on locally-specific tactical adaptation. While the need for powered granule blowers for occasionally difficult habitats [42] is now obvious, this was not the case at the outset of this endeavour. With the scheduled scale-up of the interventions to nine wards from June 2007 and 15 wards from June 2008 further surprises are anticipated. Solutions to such challenges are likely to be found, however, the maturation of the programme's capacity to tackle the full range of such operational challenges will require at least additional 1–2 years of practical implementation experience.

It is necessary to point out that the UMCP is currently a combination of an operational programme, a research project and a training platform to provide the evidence and capacity needed for future programmes. Therefore, the activities implemented to date are very comprehensive and intensive. As the programme matures there should be opportunity to scale down some of these activities. For example, the mapping and recording of every plot could be simplified since for a solely operational programme not each individual water body needs to be characterised by an individual ID number. Furthermore, while weekly application of larvicides to all aquatic habitats remains necessary, the weekly larval surveillance (follow up) of every single habitat could be reduced to spot checks of a representative number of randomly selected habitats every week for monitoring and evaluation purposes. Nevertheless, it needs to be emphasized that such strategies should only be developed and fine-tuned over time as the program staff gains more experience. To monitor the disease impact of a vector control programme household and malaria surveys [68] need to be implemented. Nevertheless, these need not to be necessarily part of the vector control programme but should be implemented through national disease monitoring and evaluation procedures, preferably integrated in health information systems for core health and poverty indicators that serve local, national and global needs [69].

## Conclusion

A novel management system for implementing systematic larviciding of malaria vectors in African cities, that includes an intensive monitoring and evaluation component, has exhibited considerable potential for sustained, rapidly responsive, data-driven and affordable application. Despite operational and financial limitations in the first year of intervention it could be demonstrated that large-scale larviciding programmes can reduce malaria transmission in urban Africa. The true programmatic value of larviciding though can only be established through longer-term programmes which are stably financed and allow the capacities of operational teams and infrastructures to mature through direct experience of locally relevant ecological, epidemiological and institutional challenges.

## Competing interests

The programme evaluated in this manuscript is partially supported by Valent BioSciences Corporation, a commercial manufacturer of microbial larvicides. Nevertheless, none of the funders of this work had any role neither in the analysis or interpretation of the results nor in the drafting of the manuscript.

## Authors' contributions

UF, KK, MCC, GW and GFK developed all standard operating procedures concerning larval surveillance and control in a participatory manner with field staff at city, municipal and ward level. YG, PPC, NJG were involved in the development of adult mosquito sampling protocols, field data collection and analyses. KK oversaw all activities implemented by the UMCP. UF, KK and GFK planned and oversaw the larval control intervention. MCC and KK created all databases. SD and DN developed and oversaw the participatory mapping. MJV and EMM helped with protocol refinement based on evaluation of CORPs' performance. DM, MT, SWL, HM and BHS were involved in the overall design of the UMCP and regular review of progress. UF and GFK were involved in the analyses of the data and drafted the manuscript. All authors read and approved the final manuscript.

## Additional files

The following files are available for download as low-resolution Adobe Acrobat^® ^files but can also be obtained as editable Microsoft Office files from the authors in CD or DVD format so that they can be adapted to alternative settings.

## Supplementary Material

Additional file 1 Participatory mapping guidelines and TCU mapping and description forms. The document presents the standard operating procedures and data collection forms used for habitat mapping in the UMCP, Dar es Salaam. Click here for file

Additional file 2 Larval surveillance guidelines and standard operating procedure for open habitats. The document presents the standard operating procedures developed for weekly mosquito larval surveillance. Click here for file

Additional file 3 Posters describing categories for open habitats. The file shows a poster developed for training ward based staff on identification of open, sun exposed mosquito breeding sites. Click here for file

Additional file 4 Posters describing categories for closed habitats. The file shows a poster developed for training ward based staff on identification of closed mosquito breeding sites. Click here for file

Additional file 5 Larval surveillance forms for open and closed habitats. The document presents all data collection sheets used by ward and city based staff for mosquito larval surveys. Click here for file

Additional file 6 Training presentation for larval surveillance. The document shows a training presentation for ward based staff on how to recognize mosquito larval habitats and how to characterise them according to the standard operating procedures. Click here for file

Additional file 7 Guidelines for larvicide application. The document presents information on microbial larvicides, calibration and the standard operating procedures developed for weekly larvicide application. Click here for file

Additional file 8 Training presentation for larvicide application. The document shows a training presentation for ward and city based staff on how to apply microbial larvicides. Click here for file

Additional file 9 Ward-level weekly summary form for larval surveillance data and form checklist for collation in pre-labelled folders and evaluation by municipal management. The document shows the data collection form used to prepare a weekly summary of the number of aquatic habitats and their colonisation with mosquito larvae. Click here for file
