# Supplementary material for: A tool box for operational mosquito larval control: preliminary results and early lessons from the Urban Malaria Control Programme in Dar es Salaam, Tanzania
Source: Malar J. 2008 Jan 25;7:20. doi: 10.1186/1475-2875-7-20 (PMC2259364; doi:10.1186/1475-2875-7-20)

Additional file 1:

This document has been produced and made available by the Dar es Salaam Urban Malaria Control Programme. Contact: Urban Malaria Control Programme, City Medical Office of Health, City Council, P.O. Box 63320, Dar es Salaam, Tanzania, Phone: +255 22 212 1649

Guidelines for 10-cell unit mapping to be carried out by the community owned resource persons and the wards malaria vector control supervisors

I. Introduction

To find **all** mosquito breeding habitats, you have first to **know each and every square metre** in your Mtaa. Each Mtaa is composed of several **10-cell** units, which now need to be divided into **plots**, typically numbering between 10 and 20 per 10-cell unit. The only sure way to do this is to **know who owns, occupies or uses which plot of land** regardless of whether it is surveyed or unsurveyed. For the purposes of our programme, **a plot is defined as a specific physical area with an identifiable owner, occupant, or user and with clearly defined boundaries within one specific 10-cell unit.** A **plot is our basic access unit** for surveying larval habitats**.** In the built up areas, a plot is that area covered by, and surrounding a house that is owned or occupied by a named and identifiable person. In the seemingly no-man's land, a plot is that unit that a specific person owns, claims to own, or he/she regularly uses. Thus, when we refer to an "owner" of a "plot", this goes beyond just those surveyed plots with legal owners to include river valleys, open fields, swamps, cultivated areas etc. **Knowledge of who owns, occupies or uses a certain plot is very important if you are to gain unlimited and regular access in future as this is the person who has the power to say yes or no!** Consequently, to find, name and define the plots within a 10-cell unit, you **must** be accompanied by the **10-cell unit leader** or their representative from that 10-cell unit and those from the adjoining 10-cell units**.** The purpose of conducting a mapping exercise is to lay a platform that will guide the larval habitat survey. It is only after every metre square within a 10-cell unit has been assigned to a specific plot that you can start a larval habitat survey. However, even before you can start walking around finding out who owns which plot of land, it is important that the community members are made aware of **who you are**, **where you are from**, **what you are doing, why you are doing it, of what benefit is it to them, and how they can be part of it.** These questions are addressed though proper and continuous community sensitisation.

II. Step-by-step guide for plot mapping

1. First, obtain the 10-cell mapping forms (**Annex 1**) from your supervisor at the ward level.
2. Go to the specific 10-cell unit that you intent to map and get in touch with the 10-cell leader. Explain clearly to him what you are doing and request him to take you on a detailed guided tour of his 10-cell unit. In this tour, let him take you from plot to plot and to **all** plots within his 10-cell unit. Explain to the 10-cell unit leader that exhaustive mapping is important for conducting a thorough larval search and eventual larval control. In defining the 10-cell unit boundaries, it is important to involve **the 10-cell unit leaders** of the adjoining 10-cell units. Explain to the 10-cell unit leaders that unless the boundaries are correctly and mutually agreed upon, mosquitoes will breed in these boundary areas and fly into the 10-cell units.
3. On the 10-cell unit mapping form, fill in the date, the name of the Municipality, the Ward, the Mtaa, the 10-cell unit number and the name of the 10-cell unit leader.
4. Once on a specific plot, assign an identification number (**Plot ID**) to it and fill in this number in the column named "Plot ID" in the 10-cell unit mapping form. If it is within a surveyed/built up area, also include the house number in the column named "House Number" in the 10-cell unit mapping form. For each and every 10-cell unit, assigning of plot ID numbers should be independent of the plot numbers of the other 10-cell units.
5. Then, ask who owns, occupies or regularly uses the plot and write down his/her name in the column named "Owner's Name" in the 10-cell unit mapping form.
6. With the help of the owner, occupant or regular user, clearly define the boundaries making a rough sketch of the plot on a piece of paper. This will assist you in constructing a map for all plots in that 10-cell unit (**see step 9**). Since two or more 10-cell units may share some of the open areas, it is important to involve all the **10-Cell Unit Leaders** from the adjoining 10-cell units to define boundaries for plots as well as those for the 10-cell units. Great care should be taken when defining boundaries so that no part of the boundary is left unassigned to a plot. Therefore, the only way to define a boundary is to know what is on the other side of the boundary i.e. another plot in a different 10-cell unit, or in a different ward. This will ensure **complete** and **full** coverage of each and every square metre of a 10-cell unit. For areas covered by common facilities and infrastructure like roads, rail, drains etc, assign them to one plot with a specified plot ID number (look at how the drain in annex **2 B** has been allocated to plots).
7. Describe in details the location of the plot such that even a stranger to the 10-cell unit can locate it using your description. Fill in this description in the column named "Plot location description (where is it in the 10-cell unit) and its basic characteristics" in the 10-cell unit mapping form.
8. Explore the plot and describe its basic characteristics (for example, is it flat, flooded, what is growing there, rocky, hilly, cultivated, construction ongoing, well or poorly drained etc.) in the column named "Plot location description (where is it in the 10-cell unit) and its basic characteristics" in the 10-cell unit mapping form. However, if there is no unique feature or characteristics in the plot, then describing its location in step **7** above will be enough.
9. After you have defined all the plots in a 10-cell unit, have completed steps 5-10 above for each and every plot in that 10-cell unit and have agreed on the 10-cell unit boundary with the leaders of the adjoining 10-cell units, on a separate page named "10-cell unit plots map" (**Annex 1B**), draw a map of the 10-cell unit to include all the plots you have described in it. Remember to include the Plot ID number for each plot on the map. Also fill in the date, the municipality, the ward, the 10-cell unit number and the name of the 10-cell leader at the top part of this 10-cell unit plots map form.
10. After the map is completed move to the next 10-cell unit and repeat the above procedure.

11. Later, when checking the quality of your 10-cell unit mapping, either the ward supervisor, or the municipal malaria control inspector for vector control, will assist you fill in the GPS readings in the column named "GPS" in the 10-cell unit mapping form.
12. Attached (**Annex 2**) is a hypothetical example on how to go about the 10-cell unit mapping exercise. Study in carefully as this will help you develop an idea on how to carry out this exercise.
13. After the 10-cell unit plots map forms are filled in and the maps drawn, they should be taken to the ward office. From here the supervisor will take them for photocopying at the Municipal Malaria Control Coordinator's office. He (the supervisor) will receive copies of the filled in forms and maps to take them back to the Community Owned Resource Persons for their day-to-day reference.

NB: Remember that you will use the filled-in 10-cell unit mapping forms to guide you in your larval survey exercise and therefore you should fill them in carefully and accurately!!

Always fill in the forms using black or black ball pens.

Annex 1A: 10-cell unit plots map form

10-cell unit plots map form

Municipality:______________________________ Ward:_____________________________ MTAA:________________________________ 10-cell unit:________________________
Date_____________________________

Plot ID	House Number	Owner's name	Plot location decription (where is it in the 10-cell unit) and its basic characteristics	GPS (UTM/WGS84)	
				Northing	Easting

Signatures: Spervisor______________________
CORP_________________________

Fomu ya ramani ya shina Tarehe:___/___/______

Manispaa___________________Kata________________ Mtaa__________________
Namba ya shina______________ Jina la Mjube__________________

Annex 2: a hypothetical example on how to go about the 10-cell unit mapping exercise.
Below are two diagrams (**A** & **B**). Study them carefully and then read the notes that follow.

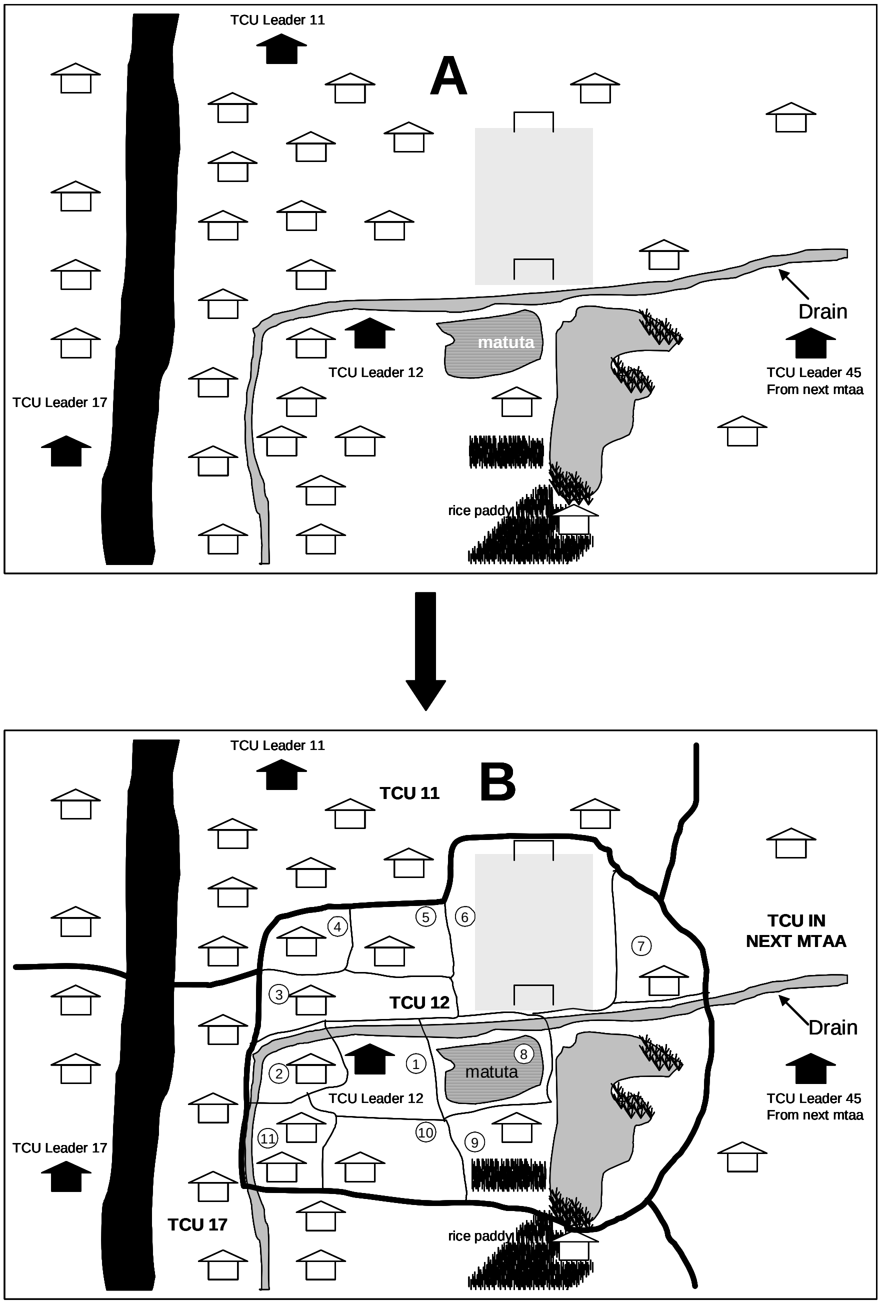

Notes on the Diagrams

- Diagram **A** represents how a part of Dar es Salaam City would look like to any other person who is not interested in 10-cell units mapping whereas diagram **B** represents what we would like to achieve in our 10-cell unit mapping exercise. Note that the two diagrams represent the same and one area.
- Diagram **A** represents how things appear to us (on the ground & in our minds) before carrying out the 10-cell units mapping exercise whereas diagram **B** represent how things will be on paper and in our minds after carrying out the 10-cell units mapping.

- The **dark** houses represent the 10-cell units leaders' houses in this particular locality. Therefore there are 4 10-cell units represented in this diagram, 3 (Numbers 11, 12 & 17) are in the same Mtaa while 1 (number 45) is from another Mtaa.
- Now, assume that today, you want to carry out a plot mapping exercise in 10-cell unit number 12 located in a Mtaa called Mtambani in Vingunguti Ward of Ilala Municipality.

1. The first step would be to collect the 10-cell unit mapping form from your ward supervisor (the vector control supervisor for Vingunguti Ward).
2. Then you would move to the 10-cell unit number 12 and contact its leader (**TCU Leader 12**). After explaining the purpose of your visit to the TCU Leader 12, ask him to take you on a detailed guided tour of his 10-cell unit. In this tour, let him take you from plot to plot and to **all** plots (11 in this case). Then follow steps the follow steps **3-12** as explained in the **Step-by-step guide for plot mapping.**

After the exercise, you should have a **completed 10-cell unit mapping form** and **a map** for that 10-cell unit (**See below**).
NB: See how the drain has been associated with specific plots.
Note that the 4 10-cell units leader should be involved in defining the boundaries of **TCU 12**

Completed 10-cell unit mapping form for 10-cell unit number 12 shown in diagrams A & B above

Plot Description Form

Municipality: Ilala Ward: Vingunguti MTAA: Mtambani 10-cell unit: 12

Date:24/06/04 10-cell unit leader:Abdalla Mwasiba

Plot ID	House Number	GPS	Owner's name	Short name for the plot	Plot location decription (where is it in the 10-cell uni	Basic Characteristics
1	Vngt/Mtb/13		Abdalla Mwasimba	Kwa Mjumbe	Next to the drain and large matuta farm	This is gerally a dry plot but there is a permanent drain running near it
2	Vngt/Mtb/10		Jonh Kahama	Corner	At the corner of the drain and next to Mjumbe's hous	A small plot and much is covered by the open drain often with water water
3	Vngt/Mt/75		Joseph Kibwana	Plot ndefu	A long plot from TCU 17 boundary to footbal pitch	Dry but near a permanently wet drain
4	Vngt/Mtb/22		Mwanahawa Ali	Dukani	At the corner bordering TCU 11 & TCU 17	Has a burst pipe most of the time with water filling most of the small drains
5	Vngt/Mtb/13		Juma Kajembe	Mwembeni	Bordering the football pitch and TCU11	Prone to flooding during the wet season. Even in the dry season parts of it are wet
6	No house		Vijana wa Vingunguti FC	Uwanjani	Football pitch bordering TCU 11 and the drain	Flooded during wet season. Has several house-construction foundations the fringes
7	Vngt/Mbt/103		Bakari Jaku	Pembeni	Bordered by the drain, the footbal pitch and TCU11	Flooded most of the times with a big part covered by leeds and tall grass
8	No house		Mukurima Mashuhuri	Shama safi	Bordered by Mjumbes plot, pond, and drain	Usually cultivated, has several irrigation channells & man-made holes & matuta
9	Vngt/Mtb/44		Edith Ikupa	Kwenye bwawa	Bordered by drain, footbal pitch, TCU17 & TCU45	Wet throughout the year with regular rice cultivation, has a big pond.
10	Vngt/Mtb/65		Cyrus Makumi	Mgema	South of Mjumbe's plot & bordering TCU 17	Half of it normally wet and the other half normally dry
11	Vngt/Mtb/07		Mwanahamisi Juma	Nyuma Mbili	Bordering drain on the West & TCU17 on the South	Gerally a dry plot but there is a permanent drain running near it

Signatures: Spervisor____________________
CORP____________________
10-cell leader____________________

Completed 10-cell unit plot map for 10-cell unit number 12 shown in diagrams A & B

10-cell unit plots map form

Municipality: Ilala Ward: Vingunguti Mtaa: Mtambani 10-cell Unit: 12

Date:24/6/04 10-cell unit leader: Abdalla Mwasiba

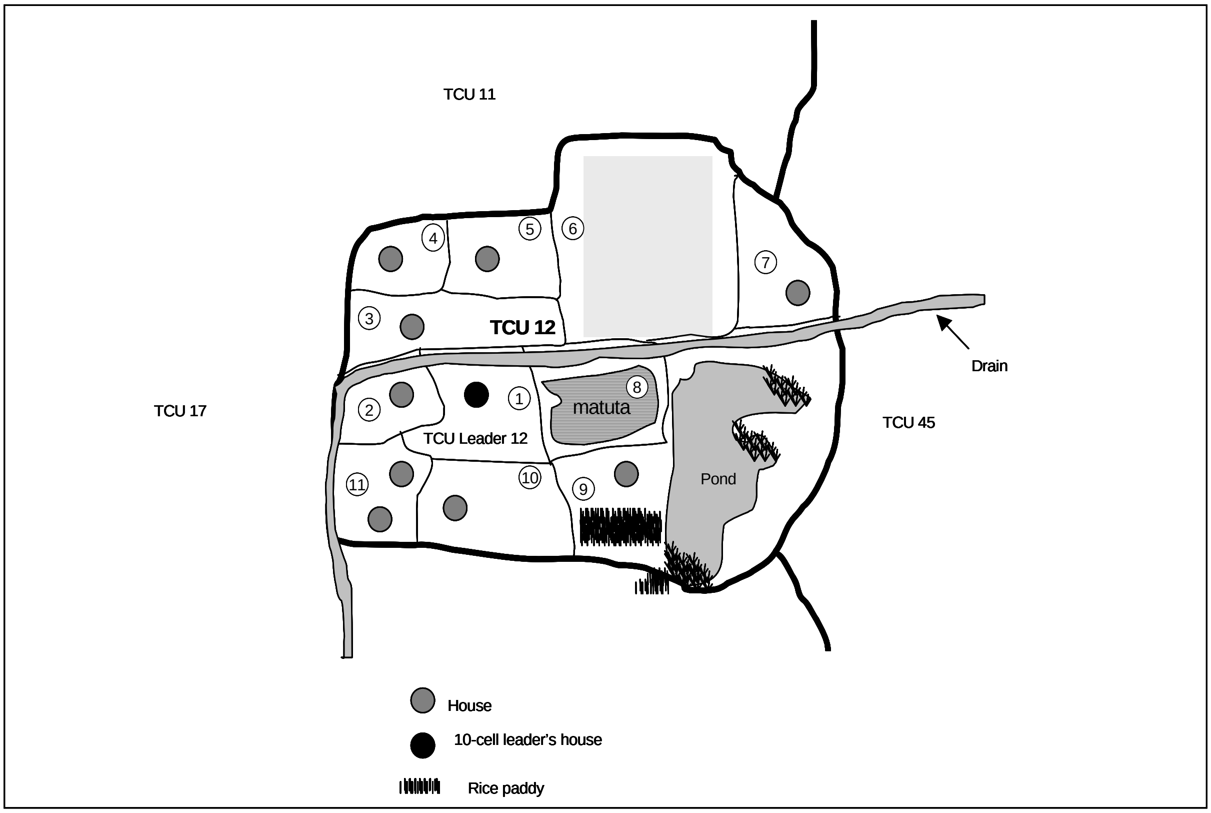

Supplement: Additional file 1 — Participatory mapping guidelines and TCU mapping and description forms. The document presents the standard operating procedures and data collection forms used for habitat mapping in the UMCP, Dar es Salaam. [file 1475-2875-7-20-S1.pdf]
