# Supplementary material for: A tool box for operational mosquito larval control: preliminary results and early lessons from the Urban Malaria Control Programme in Dar es Salaam, Tanzania
Source: Malar J. 2008 Jan 25;7:20. doi: 10.1186/1475-2875-7-20 (PMC2259364; doi:10.1186/1475-2875-7-20)
Supplement: Additional file 2 — Larval surveillance guidelines and standard operating procedure for open habitats. The document presents the standard operating procedures developed for weekly mosquito larval surveillance. [file 1475-2875-7-20-S2.pdf]

Additional file 2:

This document has been produced and made available by the Dar es Salaam Urban Malaria Control Programme. Contact: Urban Malaria Control Programme, City Medical Office of Health, City Council, P.O. Box 63320, Dar es Salaam, Tanzania, Phone: +255 22 212 1649

GUIDELINES TO SEARCHING FOR MOSQUITO BREEDING HABITATS (STAGNANT WATER) AND CONDUCTING LARVAL SURVEY

Revised March 2005

Background:
Although all mosquitoes breed in water the available type of breeding habitat is likely to change at different times of the year. *Anopheles* larvae prefer water that is exposed to the sun, whilst *Culex* larvae can be found everywhere. *Anopheles* larvae and especially pupae are usually concentrated in certain parts of large breeding sites, which make larval collection difficult. Edges of sites and patches of vegetation are often places where larvae can be found; sun exposure and wind also determine where mosquito larvae occur. Since mosquitoes breed in almost any kind of water body it is important to check all water bodies during a larval survey.

This larval survey is designed to inform us of the distribution of the aquatic stages of disease transmitting mosquitoes over space and time. After collecting baseline data on mosquito habitats and larvae for one year we plan to begin larval control operations in selected areas. The larval surveys will help us target future control activities.

Our Goal: To survey all potential mosquito breeding habitats (all stagnant water bodies) in 15 wards of urban Dar es Salaam in order to plan efficiently the interventions for larval control from 2006 onwards.

Why do we need to collect data on mosquitoes in a malaria control programme?

To control the mosquitoes that transmit malaria, we have to know them well! We need to have the basic but accurate information that is essential for proper planning of our control measures. We need to know **WHAT** kind of mosquitoes we are going to target, **WHEN** are we going to target them, **WHERE** we going to target them and **HOW** are we going to target them.

Therefore, we need to identify:
- If malaria mosquitoes are present in the area, and if present, which ones?
- Which other mosquitoes are around that are not malaria mosquitoes.
- Where are the different mosquitoes breeding?
- Are the breeding places available throughout the year?
- How do the breeding places look like, how do they differ?
- How can we prevent mosquitoes from breeding in the various sites?

All this information is necessary to design a powerful mosquito larval control operation and to assess, in the following years, whether we have been successful in our control operations. We should be able to compare the mosquito densities before and after larvicide treatments or environmental management and see a remarkable reduction in the number of mosquitoes. Furthermore, The Demographic Survey Teams of the programme are

collecting data on malaria cases in the preparation phase as well as during the larval control activities to show if we have an impact on malaria in the community with our control operation. A good baseline data collection period is most important if a control operation is to be successful in future! Therefore all field staff involved ought to have **great interest, motivation, responsibility and enthusiasm** to make the programme work! If we succeed during this pilot phase, the programme can be continued, expanded and improved for the benefit of all inhabitants of Dar Es Salaam City.

Mosquito Larval Survey

Why do we carry out larval survey?

We carry out larval survey in order to:

- Identify potential mosquito breeding habitats (stagnant water bodies) and ascertain the presence or absence of mosquito larvae in them.
- Determine the availability of mosquito breeding habitats around the year (during dry and rainy seasons).
- Determine the preferred larval habitats for mosquitoes.
- Describe changes in mosquito larvae densities over time.
- Assess the impact of mosquito control activities on larval abundance.

Where do mosquitoes breed?

- Mosquitoes breed only in water! The larvae cannot survive anywhere else, they DO NOT breed in grass or bushes.
- Mosquitoes can breed in any kind of water and therefore **ALL** kinds of water bodies have to be checked for mosquito larvae in a larval survey.
- Mosquitoes do not breed in fast running water of rivers, but can breed at the edges where the water is not moving fast, in cattle hoof prints along a river and in slow flowing drains.

To identify ALL mosquito larval habitats it is essential to be exhaustive and check **all** possible breeding places (**any stagnant water body**), even those that are hard to reach, this enables determination of the types of habitats most likely to harbour the larvae of mosquitoes

Mosquito habitat types to be distinguished in larval surveys

In this larval survey, we try to characterise the breeding habitats (water bodies) we find, to investigate which habitats are the most common habitats and which of them are most attractive and productive for mosquito larvae. In our programme, we will survey **Open Habitats** weekly and **Closed Habitats** every 3 months.

What are Open Habitats?

Open habitats are defined as water bodies that are exposed to the open air and light. This means that light can reach the water surface, also plants can grow inside. In most of these sites the water can be readily reached with the dipper.

What are Closed Habitats?

Closed Habitats are in contrast to the open habitats defined as water that can be found in closed and dark environments. Often it will be more complicated to reach the water surface with the dipper since openings to access have to be identified and opened in many cases.

We want to characterise the open and closed mosquito larval habitats of Dar es Salaam following closely the definitions below.

Open Habitats: Habitat Codes (see data sheet) and Habitat definition

1: Puddles and Tyre Tracks

Puddles are small to medium sized stagnant water areas. Most puddles are less than 10 m in perimeter (<10 m). Some of them might though reach between 10 and 100 m in perimeter (10-100 m). The source of the water is rain water and water run off. The water is shallow, less than 0.5 m deep (<0.5 m). Tyre tracks are just as special type of a puddle. Vehicles often leave tracks in the ground especially if the ground is wet. These tracks/depressions hold water longer than the surrounding areas and thus serve as potential mosquito breeding grounds.

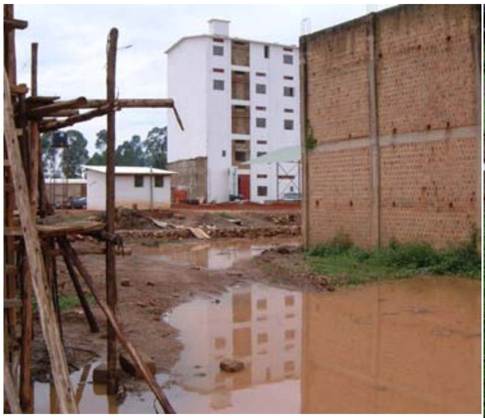

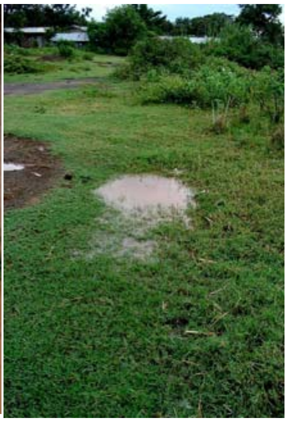

2: Swampy Areas

The habitat code Swampy Areas summarises a number of different looking water bodies. They all have in common that because of a very high ground water table there is water standing on the ground for quite some time during the year or even continuously. The source of the water is ground water, but can additionally be fed by rainwater.

Swampy areas are for example areas that border a large water body like a river or creek where water is permanent throughout the year. Often can the water here inside the swamp be deep (>0.5 m). The vegetation is often characterised by tall reeds (left photo) and/or floating plants.

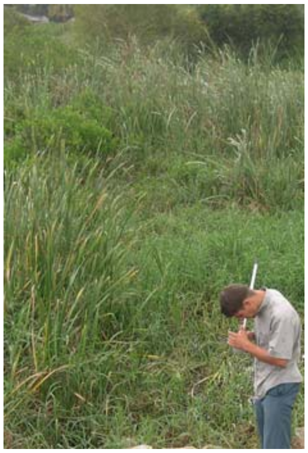

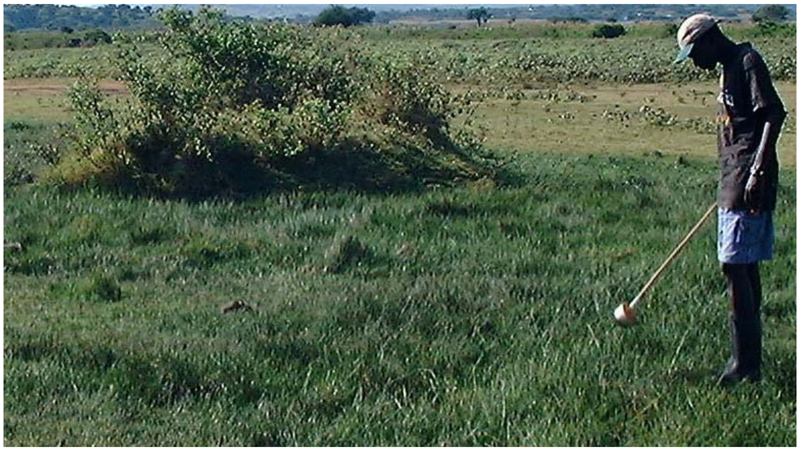

Other swampy areas might be characterised by short grassy vegetation (right photo) where water stands due to high water table or due to a spring/seepage that brings water from the ground to the surface.

3: Mangrove swamp

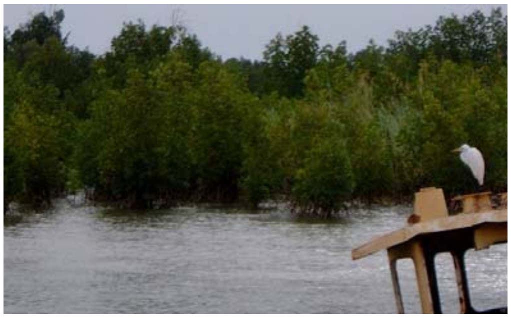

These are areas near the sea only, they can not be found far away from the sea. They have mangrove trees growing with water underneath. The water is tidal because it comes from the sea but some small pools might remain throughout. The water is salty.

4: Drains and Ditches

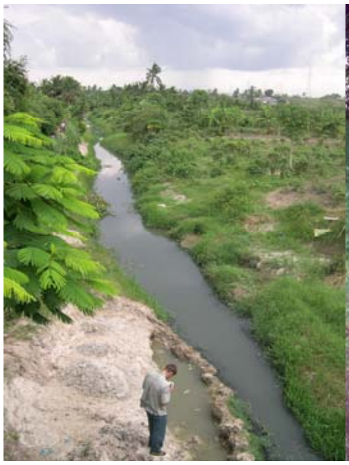

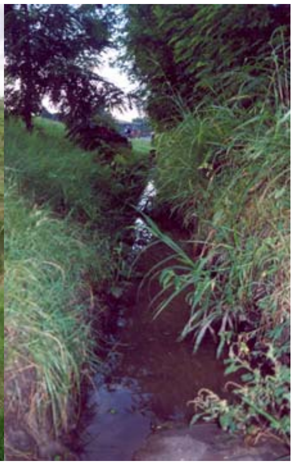

Drains and ditches are man-made and constructed for the purpose of getting rid of water or to irrigate an area. Drains specifically are constructed for water to flow and therefore to drain water from or irrigate the area. However, most of them get blocked with litter thus holding water for longer duration. Ditches are also man-made but do not necessarily support water to flow, they support stagnant water bodies. Drains and ditches can be cement lined but also can just be dug in the ground. It is important to notice that they are man-made and made for a specific purpose to channel the water. They can also be small for example to channel water from a tap to the garden, as long as that channel is man-made. It would be very desirable if you could describe what type of a drain or ditch you are recording in the comment area of your data sheet.

5: Construction pits, foundations and man-made holes

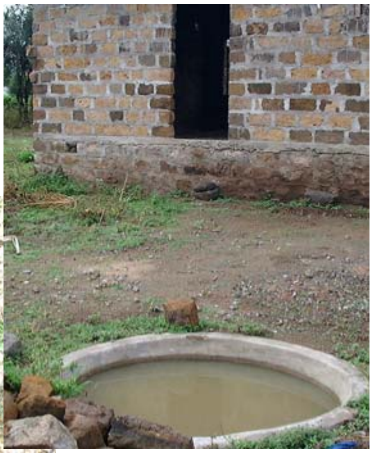

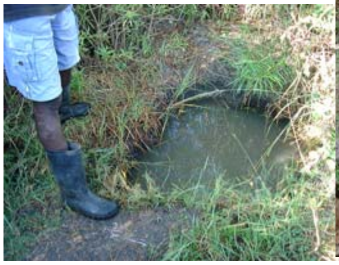

These are small to medium sized man-made habitats that can collect water, for example unfinished constructions of pit latrines, holes in the ground for rubbish collection, holes for water collection or storage, holes for ground water collection for irrigation (wells), foundations of houses that will be built, any man-made pit structure that holds water and is open (also pits holding water from the bathroom). These habitats are usually in the ground and are therefore not moveable.

6: Water storage or other Man-made containers:

Any container that holds water that could serve mosquitoes to breed in for example open water storage tanks, barrels, tyres, buckets, clay pots, livestock feeding trays. Most of these habitats are therefore on top of the ground and can be moved from one place to another (except big open cemented water tanks etc.)

7: Rice paddy (Rice field)

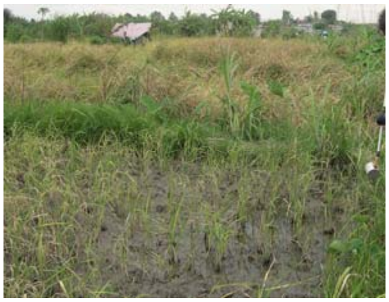

These are plots where rice grows. Those plots can be flooded for longer periods of time. Larvae can mainly be found on the edges of the fields. You need to pay close attention to fields that are drying up because the water is collected in small pools all over the field. The mosquito larvae can then be concentrated in very small water collections that might not easily be found.

8: Matuta

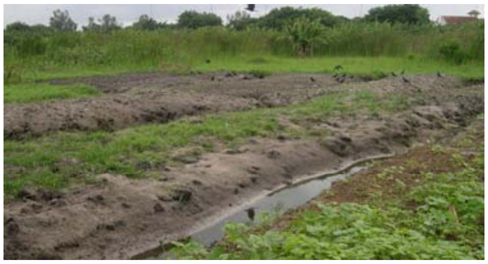

These are raised ridges on agricultural plots. The furrows created hold water for longer duration. The water in the furrows is not evenly distributed and therefore keen observation for larvae in very small depressions particularly on the fringes is important.

9: Other Agriculture

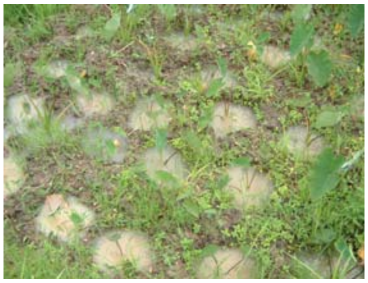

Besides Rice and Matuta other agricultural fields might provide stagnant water bodies for mosquito larvae. The water might be supplied by irrigation or by a high water table, or even rainfall.

10: Stream and River beds

Streams and Rivers are usually fast flowing water bodies that are not good for mosquito larvae to develop in. But with these streams and rivers there are often fringe area associated where the water only moves very slowly or is stagnant in areas where water pools along the river and stream edges. These fringe areas can provide good breeding habitats for mosquito larvae. Also rivers and streams that are drying up leave stagnant, pooling water behind that can serve as larval habitats.

11: Ponds

Ponds are medium to large in size. Ponds are permanent water bodies or are at least present for several months in the year. They might decrease in size with the dry season. Ponds are at least during the rainy season more than 0.5 m deep (>0.5 m, in the middle of habitat). Ponds can contain tall vegetation and floating plants, mosquito larvae are usually associated with the shallow edges of ponds.

12: Others (please describe them)

Under this category you can record any other stagnant water bodies that could be mosquito larval habitats that do not fit under any of the above-described habitats. Before you decide to record a habitat under category 12, please make sure you have checked the definitions of habitat categories 1 to 11 to make sure this habitat type is not considered there. Please, describe the habitat recorded under category 12 in the comment section of the data sheet. Be in your description as detailed as possible.

Closed Habitats: Habitat Codes (see data sheet) and Habitat definition

There are fewer types of closed habitats than the open ones. We want to distinguish between the following:

Pit latrines: These are dug on the ground and often contain water in closed and dark environments. They are good breeding habitats for culicine mosquitoes.

Soakage pits: These are closed pits connected to the latrines and often contain water. They serve as breeding grounds for culicine mosquitoes.

Septic tanks: These are constructed as underground (closed) waste storage containers. They are normally sealed but if they have a small opening, and contain water, mosquitoes do breed in them.

Others: Here you can record any other **closed** habitat that you encounter that does not fall under the definitions above. Please describe the habitat in the comment section.

Sampling mosquito larvae

The most common and easiest technique to investigate the presence or absence mosquito larvae in a habitat is dipping.

ALWAYS have:

- A dipper. A dipper can vary in shape and size, including small pans, soup ladles etc. A dipper should be light in colour inside to see the larvae easily.
- A Pen/pencil, a notebook, and the standard data recording forms/sheets

Sometimes (If need be)

- A pipette,
- Vials to collect specimen (sometimes if the samples are needed for identification).
- Ethanol to kill specimen and preserve them immediately (when the samples are needed for further processing)
- Bigger bottles or suitable containers to transport larvae alive (If live specimens are needed)

Where to do a mosquito larval search in the habitat (stagnant water body)

Note that preferred (but not restricted) sites where *Anopheles* larvae can be found, are:

- sunlit water bodies or the sun-exposed area of a water body,
- edges of water bodies,
- around low vegetation e.g. grass tuffs, round swimming debris and leaves,
- in-between floating vegetation
- except in very small sites, *Anopheles* larvae are usually NOT evenly distributed over the entire surface area.

The dipping technique

- While dipping, you should take care so that your shadow is cast away from the habitat as larvae are very sensitive and will dive to the bottom once your shadow is cast on the water
- Lower the dipper gently in an angle of 45° just below the surface so that water flows in together with any larvae that might be present. The important point to note here is that we sample by displacement suction and not by scooping. The diagram below how dipping should be done.
- Take care not to disturb the water too much as this will make larvae dive downwards. If the water is disturbed, wait for three minutes before continuing dipping.

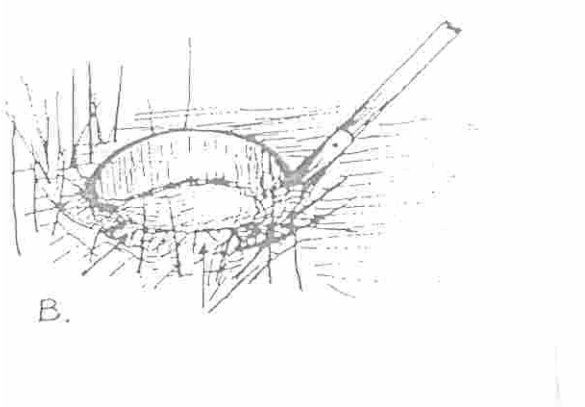

- When lifting the water, take care not to spill the water containing the larvae and pupae.
- Hold dipper steadily until larvae and pupae rise to the water surface in the dipper (this can take several minutes, especially for older instars).

- **Take at least 10 dips per habitat in different locations where mosquito larvae can be expected (edges of habitats, around vegetation, shallow areas etc.). In the case of water channels and drains or large swamps or mangrove swamps, walk along/around the habitat and take up to 60 dips/habitat to investigate for the presence of mosquito larvae.**
- If specimen are needed for further studies in the laboratory collect larvae and pupae by means of a pipette and transfer them to a bottle or vials, label the vials (date, name of sampling habitat), throw the water on the ground.
- REMEMBER that *Anopheles* mosquito densities are often quite low compared with other genera, and therefore, you have to extend your time and efforts to detect them! Furthermore, sampling pupae is extremely difficult because they are very sensitive and fast, the slightest disturbance and they disappear (dive down), additionally they are even more clustered at one spot than larvae, and therefore you should thoroughly search the habitats for pupae.

Where there is dense, floating vegetation:

- Disturb the water thus causing larvae and pupae to sink below the surface
- Clear away vegetation with the dipper and wait a few minutes for larvae and pupae to return to surface
- In clumps of vegetation e.g. grass, press dipper into it so that water flows in.

For extremely small habitats like hoof prints, you can sample larvae or pupae either with a very small sieve, with a spoon, with a pipette, or make direct observation: It can be helpful to stir the water with a stick to make it muddy and wait for the larvae and pupae to rise because they are now easily seen against the muddy background.

Step-by-step guide to searching for mosquito larval habitats, characterization of the habitats and filling in the data forms

Introduction

All the data on mosquito larval habitats is recorded in the forms provided. It is therefore important that the forms are **filled in correctly so that they reflect the true picture on the ground.** Therefore, it is important that we know what to fill in the forms and how to fill in them. We have two types of forms **1**) **Open Habitats Forms** and **2**) **Closed Habitats Forms**. Please follow the guidelines step by step as given below:

Open Habitats (these are visited once every week)

1. First, obtain the open habitats larval survey forms from your supervisor at the ward level. The **Basic Operational Unit** for our programme is the **10-cell unit** and thus the forms are designed accordingly. Also carry with you the 10-cell unit maps and their description forms that you had made during the mapping exercise
2. Go to the specific 10-cell unit that you had previously mapped into plots. Once on a 10-cell unit, fill in the date, the name of the Municipality, the Ward, and the Mtaa on the top part of the form. Also fill in the 10-cell unit number and the name of the 10-cell unit leader
3. Then, with the help of your 10-cell unit map, move from plot to plot as shown on your map.
4. Once on a specific plot within that 10-cell unit, fill in its **Plot ID** number as it appears on your map. If the plot has a house, fill in the house number.
5. Then walk exhaustively and keenly on the plot to searching for mosquito breeding habitats. Sometimes, you may not find a habitat within a plot, then write '**No Habitat' (but once you had found a certain habitat on the compound that might now be dry, record its number from previous mappings and record dry)**
6. Once a habitat is located, assign it a number (**Habitat ID**), and then fill its type (**Habitat type**) on the form using the **habitat codes** provided on the top part of the form for open habitats. If you are not sure of the habitat type, refer to the notes and pictures on different habitat types.

7. Give a brief but accurate **description** and location of the habitat on the column labelled '**Habitat description**'. This description will help you remember each and every habitat they way you have arranged them in your form. Always describe habitats in a way that you can easily remember which they are and where they are.
8. Since each and every habitat will be visited every week, the habitat type might change with time e.g. from a matuta to a rice paddy. If the habitat is the same as it was the last time you visited it, then fill in **1** in the column with the question '**Same habitat type from first visit**?' If the habitat type has changed, then fill in **2** and then fill in the code for the **habitat type** it has changed to in the column labelled '**New habitat type'**.
9. After filling in the above information on a habitat, **the actual data collection begins**. This is what should be filled in each column. Tick (√) where appropriate.

Wet? Here you observe if the habitat contains water or it is dry and tick (√) the appropriate box. The recording of when the habitat is wet or dry help us in judging how stable the habitat is over time. A stable habitat will always pose the danger of continuously producing mosquitoes.

Habitat perimeter: Here many people get confused. Perimeter means the distance all round the habitat. You get the perimeter by walking round the habitat. Approximately, each step that you walk is a meter. For example, a drain can be half a step wide but 45 steps long as shown below. If you walk around it, then you will walk 91 steps. The perimeter of the drain will be 91 meters and for the habitat perimeter the column '**10-100'** in the form should be ticked (√**).**

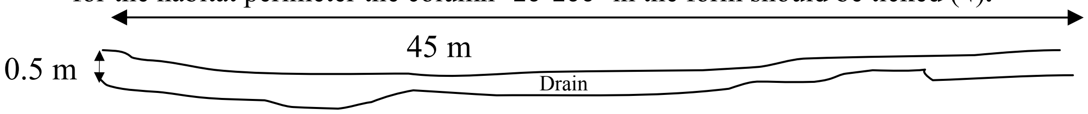

Important here is that you measure the perimeter of the water body not necessarily of the whole habitat that could contain water. So the drain might be very long but might have only water in a short area, measure the short area of water only. If a habitat is dry, meaning it does not contain any water then you can not measure any thing so the column remains blank.

The importance of estimating the habitat perimeter is to enable us calculate the amount of larvicide to be used during the larval control operations. Larvicide dosage is always calculated in terms of hectares of water surface to be covered. Therefore, the perimeter of a habitat is a good estimate of the area covered by that habitat.

Plants: The presence or absence of plants in a habitat determines the kind of larvicide to be used and the method of application. Observe the habitat for the presence or absence of plants and tick (√) where appropriate. We want to distinguish between short vegetation (not higher than your knee) and tall vegetation (much higher than your knee), floating vegetation that can be found on the water surface or no vegetation at all. Multiple ticks are possible.

Water depth: The depth of water determines the stability of a habitat as well as the type of mosquito breeding in it. Use the handle of your dipper to estimate the depth and tick (√) where appropriate. Remember, when the habitat is dry, you can not measure any water depths and therefore this column will be left blank.

Larval stage: To fill this column, you **must dip** the habitat. Take at least 10 dips per habitat in different locations where mosquito larvae can be expected (edges of habitats, around vegetation, shallow areas etc.). In the case of water channels and drains or large swamps or mangrove swamps, walk along/around the habitat and take up to 60 dips/habitat to investigate for the presence of mosquito larvae.

Record by ticking (√) all the larval stages (**Early, Late, or Both**) that you see. Recording of the stage or stages of the larvae in a given habitat is important as the larvicide only kill the larvae at a

certain stage. Therefore, we need to know what stage the larvae are before we can treat the habitat with the larvicide. Late instars larvae are indicators of poor or no larvicide treatment in the recent days. Remember that it is possible to have different larval stages in the same habitat at the same time.

Pupa: It is important to check and record the presence or absence of pupae this is the final stage of the mosquito in water. Their presence or absence helps us in judging whether a larval control operation has been successful or not. Record the presence or absence of pupae by ticking (√) the appropriate box.

Comments: Note down anything that you think is important in your larval survey exercise on the '**Comments**' column of the form. Make good use of the comment section.

10. After a careful and exhaustive searching, and after dipping for larvae in all the habitats move to the next plot.
11. After you have completed all the plots in a 10-cell unit, move to the next 10-cell unit and repeat the above procedure.

Closed Habitats

For a survey of the closed habitats, you will be looking for **Closed Habitats** like **Pit Latrines**, **Septic Tanks**, **Soakage Pits** and **Other types of closed habitats** like **Covered Waste Water Storage Tanks** in build up areas (Inside compounds/Houses)**.** Therefore, the focus is on houses within the **10-cell** units.

1. First, obtain the closed habitats larval survey forms from your supervisor at the ward level. The **Basic Operational Unit** for our programme is the **10-cell unit** and thus the forms are designed accordingly. Also carry with you the 10-cell unit maps and their description forms that you had made during the mapping exercise.

2. Go to the specific 10-cell unit that you had previously mapped into plots. Once on a 10-cell unit, fill in the date, the name of the Municipality, the Ward, the Mtaa, the 10-cell unit number and the name of the 10-cell unit leader on the top part of the form.

3. Then, with the help of your 10-cell unit map, move from plot to plot as shown on your map.

4. Once on a specific plot within that 10-cell unit, fill in its **Plot ID** number as it appears on your map. Then fill in the house number.

5. Then ask to be shown the toilets, the soak pits, the septic tanks, and any other structure associated with human waste and wastewater disposal.

6. For each and every type of the above listed habitats that you find in that compound/house, assign it an Identification Number (**Habitat ID**). For example, if you enter a house/compound and the first type of habitat you find is a Soakage pit, assign it 1 on the '**Habitat ID'** column in the form. If the second that you find is an underground wastewater storage tank, then assign it 2 on the '**Habitat ID'** column in the form.

7. Once a habitat has been identified, use the **Habitat codes** provided at the top of the form to assign it a number for its type on the column labelled '**Habitat Type'** in the form. For example, if it is a soakage pit, its habitat type is coded **3**, and therefore you fill in **3** in the column labelled '**Habitat Type'** in the form.

8. Then give a brief description of the habitat in a way that will assist you always remember it.

9. After filling in the above information on a habitat, **the actual data collection begins**. This is what should be filled in each column. Tick (√) where appropriate.

   Wet: Check whether the habitat is dry or it contains water and tick (√) where applicable. Habitats that contain water are always potential in producing mosquitoes.

   Condition of the toilet: For pit latrines examine whether their conditions are **good**, b**ad** or **full** and tick (√) accordingly in the column named **'Condition of the latrine'.**

   Habitat perimeter: Approximate the perimeter of the habitat by walking around it and tick (√) in the column corresponding to its size in the '**Habitat perimeter'** section of the form.

   Water depth: Approximate the depth of the habitat using the handle of your dipper or a longer stick and tick (√) where appropriate in the '**Water depth'** section of the form.

   Larval stage: To fill this column, you **must dip** the habitat. Use the dipper with a long handle to sample. Record by ticking (√) all the larval stages (**Early, Late, or Both**) that you see.

   Pupae: Record the presence or absence of pupae by ticking (√) the appropriate box.

   Comments: Note down anything that you think is important in your larval survey exercise in the '**Comments**' column of the form. For example, a pit latrine cannot be sampled because the hole is very narrow, or it is very deep, note this down in the '**Comments**' column of the form.

10. After a careful and exhaustive searching, and after dipping for larvae in all the habitats move to the next plot.

11. After you have completed all the plots in a 10-cell unit, move to the next 10-cell unit and repeat the above procedure.
