# Supplementary material for: A tool box for operational mosquito larval control: preliminary results and early lessons from the Urban Malaria Control Programme in Dar es Salaam, Tanzania
Source: Malar J. 2008 Jan 25;7:20. doi: 10.1186/1475-2875-7-20 (PMC2259364; doi:10.1186/1475-2875-7-20)
Supplement: Additional file 3 — Posters describing categories for open habitats. The file shows a poster developed for training ward based staff on identification of open, sun exposed mosquito breeding sites. [file 1475-2875-7-20-S3.pdf]

Open Habitats (12 habitats codes)

1: Puddles and Tyre Tracks

Puddles
- small to medium sized
- stagnant water
- water source = rain water and water run off
- shallow water = less than 0.5 m deep (<0.5 m)

Tyre tracks
- made by wheels of vehicles
- these tracks/depressions hold water longer than the surrounding areas, these are potential mosquito breeding grounds

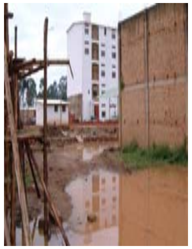
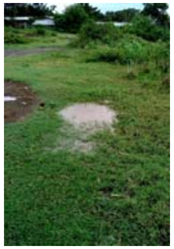

2: Swampy Areas

- very high ground water table, but can be fed by rainwater
- can border a large water body like a river or creek
- tall reeds and/or floating plants (left photo)
- short grassy vegetation with water seepage (right photo)

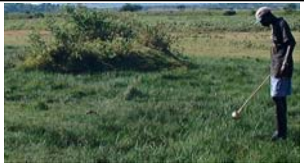

3: Mangrove swamp

- near the sea only
- salty water
- water is tidal
- small pools
- mangrove trees growing with water underneath

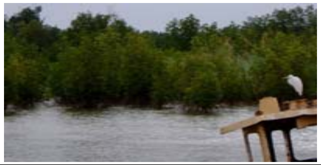

4: Drains and Ditches

- man-made for getting rid of water or to irrigate an area
- the water should flow, but if blocked with litter, the drains and ditches can become stagnant water bodies
- cement lined or just dug in the ground

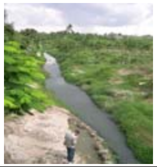
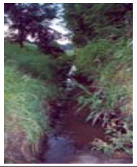

5: Construction pits, foundations and man-made holes:

- small to medium sized
- man-made habitats to collect water
- open stagnant water
- in the ground and not moveable
- examples: unfinished constructions of pit latrines, holes in the ground for rubbish collection, holes for water collection or storage, holes for ground water collection for irrigation (wells), foundations of houses

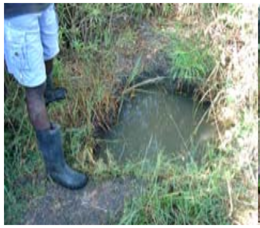
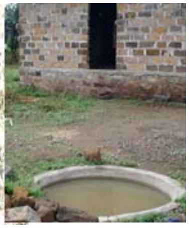

6: Water storage or other Man-made containers:

- any container that holds water for more than one week
- examples: open water storage tanks, barrels, tyres, buckets, clay pots, livestock feeding trays
- can be moved from one place to another (except big open cemented water tanks etc.)

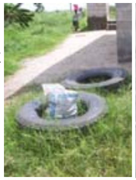
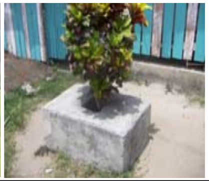

7: Rice paddy (Rice field)

- plots where rice grows
- when fields are drying up the mosquito larvae can then be concentrated in very small water pools

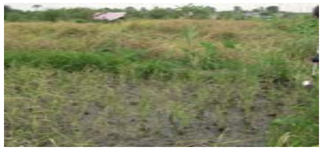

8: Matuta

- raised ridges on agricultural plots
- furrows created to hold water for longer duration

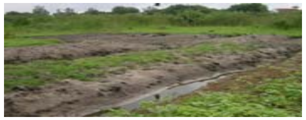

9: Other Agriculture

- other agricultural area that might provide stagnant water bodies for mosquito larvae
- water source = irrigation or high water table or rainfall

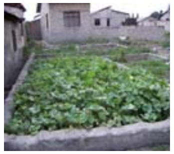
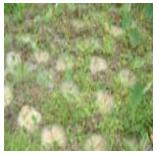

10: Streams and River beds

- flowing water bodies
- the edge of the stream or river, where the water is slow moving or stagnant and when streams and rivers are drying up leave stagnant pooling water that can serve as larval habitats

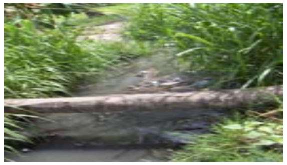

11: Ponds

- medium to large in size
- open water
- permanent water or present for several months in the year
- tall vegetation and floating plants

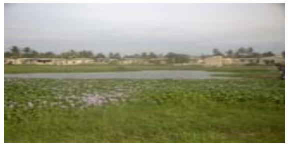

12: Others (please describe them)

- any other stagnant water bodies that could be mosquito larval habitats and do not fit under any of the habitat categories 1 to 11
- In the comment section of the data sheet, the description should be as detailed as possible

This document has been produced and made available by the Dar es Salaam Urban Malaria Control Programme. Contact: Urban Malaria Control Programme, City Medical Office of Health, City Council, P.O. Box 63320, Dar es Salaam, Tanzania, Phone: +255 22 212 1649
