# Supplementary material for: A tool box for operational mosquito larval control: preliminary results and early lessons from the Urban Malaria Control Programme in Dar es Salaam, Tanzania
Source: Malar J. 2008 Jan 25;7:20. doi: 10.1186/1475-2875-7-20 (PMC2259364; doi:10.1186/1475-2875-7-20)
Supplement: Additional file 4 — Posters describing categories for closed habitats. The file shows a poster developed for training ward based staff on identification of closed mosquito breeding sites. [file 1475-2875-7-20-S4.pdf]

Closed Habitats (4 habitats codes)

1: Pit latrines: dug in the ground and often contain water

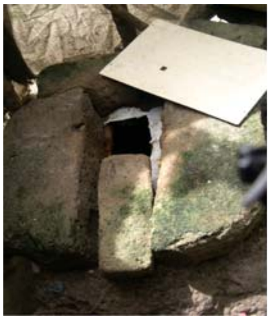
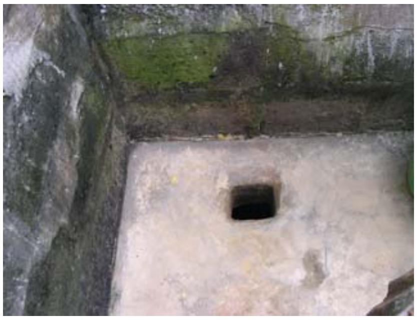
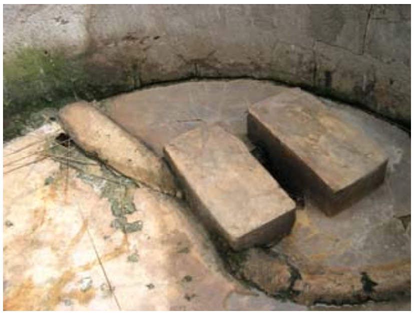

2: Septic tanks:

- underground (closed) waste storage containers
- normally sealed but may have a small opening

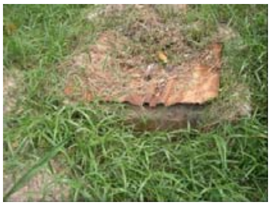
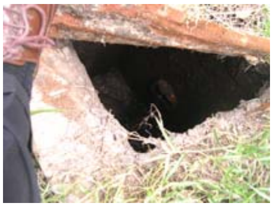
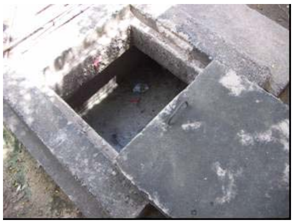
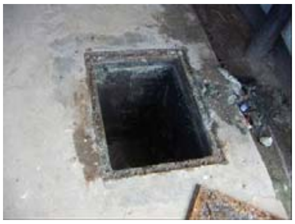
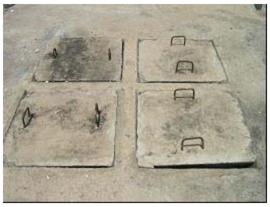
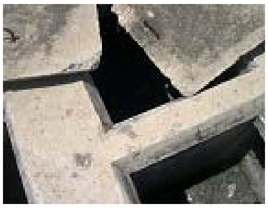

3: Soakage pits: closed pits connected to the latrines and often contain water

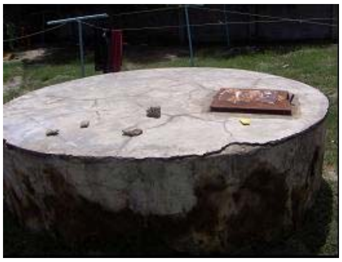
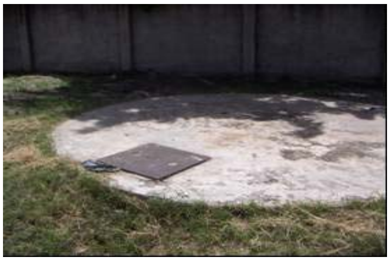
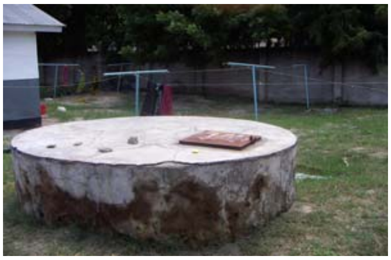

4: Others:

- any other **closed** habitat that does not fall under the definitions above
- please describe the habitat in the comment section

This document has been produced and made available by the Dar es Salaam Urban Malaria Control Programme. Contact: Urban Malaria Control Programme, City Medical Office of Health, City Council, P.O. Box 63320, Dar es Salaam, Tanzania, Phone: +255 22 212 1649
