# Supplementary material for: A tool box for operational mosquito larval control: preliminary results and early lessons from the Urban Malaria Control Programme in Dar es Salaam, Tanzania
Source: Malar J. 2008 Jan 25;7:20. doi: 10.1186/1475-2875-7-20 (PMC2259364; doi:10.1186/1475-2875-7-20)
Supplement: Additional file 5 — Larval surveillance forms for open and closed habitats. The document presents all data collection sheets used by ward and city based staff for mosquito larval surveys. [file 1475-2875-7-20-S5.pdf]

# Ward level mosquito larval habitat survey - Open habitats

**Serial number of this form** ______________________________

**Serial number on the map form** ______________________________

**Ten cell unit identifier** ______________________________

Municipality:______________________ Ward:_________________________ MTAA:________________________ 10-cell unit:______________________ 10-cell leader:_________________________

**Habitat codes:**

1: Puddles&tire tracks
2: Swampy areas
3: Mangrove Swamp / Saltwater marsh
4: Drain/Ditch
5: Construction pits/foundations/man-made holes
6: Water storage container
7: Rice paddy
8: Matuta
9: Other agriculture
10: Stream/river bed
11: Pond
12: Other (describe below)

| Plot ID | Habitat ID | Habitat type | Same habitat type from last visit? 1=Yes 2=No 3=First visit | Previous habitat type | Habitat description | House number | Wet? | | Habitat perimeter | | | Plants | | | | Water depth | | Larval stage Anoph. | | | Larval stage Culex | | | Pupae | | Comments |
|---|---|---|---|---|---|---|---|---|---|---|---|---|---|---|---|---|---|---|---|---|---|---|---|---|---|---|
| | | | | | | | dry | Contains water | < 10 m | 10-100 m | > 100 m | None | Short vegetation | Tall vegetation | Floating plants | < 0.5 m | > 0.5 m | Absent | Early | Late | Absent | Early | Late | Absent | Present | |
| | | | | | | | | | | | | | | | | | | | | | | | | | | |
| | | | | | | | | | | | | | | | | | | | | | | | | | | |
| | | | | | | | | | | | | | | | | | | | | | | | | | | |
| | | | | | | | | | | | | | | | | | | | | | | | | | | |
| | | | | | | | | | | | | | | | | | | | | | | | | | | |
| | | | | | | | | | | | | | | | | | | | | | | | | | | |
| | | | | | | | | | | | | | | | | | | | | | | | | | | |
| | | | | | | | | | | | | | | | | | | | | | | | | | | |
| | | | | | | | | | | | | | | | | | | | | | | | | | | |
| | | | | | | | | | | | | | | | | | | | | | | | | | | |
| | | | | | | | | | | | | | | | | | | | | | | | | | | |
| | | | | | | | | | | | | | | | | | | | | | | | | | | |
| | | | | | | | | | | | | | | | | | | | | | | | | | | |
| | | | | | | | | | | | | | | | | | | | | | | | | | | |
| | | | | | | | | | | | | | | | | | | | | | | | | | | |
| | | | | | | | | | | | | | | | | | | | | | | | | | | |
| | | | | | | | | | | | | | | | | | | | | | | | | | | |
| | | | | | | | | | | | | | | | | | | | | | | | | | | |
| | | | | | | | | | | | | | | | | | | | | | | | | | | |
| | | | | | | | | | | | | | | | | | | | | | | | | | | |
| | | | | | | | | | | | | | | | | | | | | | | | | | | |
| | | | | | | | | | | | | | | | | | | | | | | | | | | |
| | | | | | | | | | | | | | | | | | | | | | | | | | | |
| | | | | | | | | | | | | | | | | | | | | | | | | | | |
| | | | | | | | | | | | | | | | | | | | | | | | | | | |
| | | | | | | | | | | | | | | | | | | | | | | | | | | |
| | | | | | | | | | | | | | | | | | | | | | | | | | | |
| | | | | | | | | | | | | | | | | | | | | | | | | | | |
| | | | | | | | | | | | | | | | | | | | | | | | | | | |
| | | | | | | | | | | | | | | | | | | | | | | | | | | |

CORPS Signature:____________________________ _______ Date:________/________/__________ Supervisors Signature:____________________________ Date of check :________/________/__________

## Ward level mosquito larval habitat survey - Closed habitats

**Serial number of this form** ______________________

**Serial number on the map form** ______________________

**Ten cell unit identifier** ______________________

Municipality:_____________________ Ward:________________________ MTAA:_______________________ 10-cell unit:_____________________ 10-cell leader:________________________

**Habitat codes:** 1 - Pit Latrine 2 - Soakage Pit 3 - Septic Tank 4 - Other_____________________

| Plot ID | House number | Habitat ID | Habitat type | Habitat description | Wet? | | Condition of the latrine | | | Habitat perimeter | | | Water depth | | Larval stage | | | Pupae | | Comments |
|---|---|---|---|---|---|---|---|---|---|---|---|---|---|---|---|---|---|---|---|---|
| | | | | | dry | contains water | Good | Bad | Full | < 10 m | 10-100 m | > 100 m | < 0.5 m | > 0.5 m | None | Early | Late | Present | Absent | |
| | | | | | | | | | | | | | | | | | | | | |
| | | | | | | | | | | | | | | | | | | | | |
| | | | | | | | | | | | | | | | | | | | | |
| | | | | | | | | | | | | | | | | | | | | |
| | | | | | | | | | | | | | | | | | | | | |
| | | | | | | | | | | | | | | | | | | | | |
| | | | | | | | | | | | | | | | | | | | | |
| | | | | | | | | | | | | | | | | | | | | |
| | | | | | | | | | | | | | | | | | | | | |
| | | | | | | | | | | | | | | | | | | | | |
| | | | | | | | | | | | | | | | | | | | | |
| | | | | | | | | | | | | | | | | | | | | |
| | | | | | | | | | | | | | | | | | | | | |
| | | | | | | | | | | | | | | | | | | | | |
| | | | | | | | | | | | | | | | | | | | | |
| | | | | | | | | | | | | | | | | | | | | |
| | | | | | | | | | | | | | | | | | | | | |
| | | | | | | | | | | | | | | | | | | | | |
| | | | | | | | | | | | | | | | | | | | | |
| | | | | | | | | | | | | | | | | | | | | |
| | | | | | | | | | | | | | | | | | | | | |
| | | | | | | | | | | | | | | | | | | | | |
| | | | | | | | | | | | | | | | | | | | | |
| | | | | | | | | | | | | | | | | | | | | |
| | | | | | | | | | | | | | | | | | | | | |

CORPS signature:_________________ _________ Date:________/________/__________ Supervisors signature:_____________________ Date of check :________/________/__________

## Taarifa ya uchunguzi wa mazalio ya wazi ya mbu ngazi ya kata

**Serial namba ya fomu hii** ______________________________

**Serial namba ilioko kwenye fomu ya ramani ya shina hili** ______________________________

**Namba ya shina ya pekee** ______________________________

Manisipaa:____________________ Kata:____________________ Mtaa:________________________ Namba ya shina:________________ Jina la Mjumbe:_______________________________________

**Aina ya mazalio:**
1: Dimbwi na kashata za matairi ya magari
2: Mbojimboji/ziwa la matope
3: Bwawa maji chumvi/Mikoko
4: Mfereji/Kijito
5: Shimo/Ujenzi/Mchanga
6: Chombo cha kuhifathia maji
7: Mpunga
8: Matuta
9: Aina nyingine ya kilimo
10: Mto
11: Bwawa maji baridi
12: Aina Nyingine (Elezea)

| Namba ya utambulisho wa ploti | Namba ya zalio | Aina ya zalio | Aina ya zalio inafanana na ulipo zuru awali 1=Ndio 2=Hapana 3=jipya | Aina ya zalio la awali | Maelezo kuhusu zalio | Namba ya Nyumba. | Maji? | | Ukumbwa wa zalio | | | Mimea | | | | Kiina cha maji | | Hatua za viluwiluwi | | | | | | Mabuu | | Maoni |
|---|---|---|---|---|---|---|---|---|---|---|---|---|---|---|---|---|---|---|---|---|---|---|---|---|---|---|
| | | | | | | | | | | | | | | | | | | Anoph. | | | Culex | | | | | |
| | | | | | | | Hakuna | Yapo | < 10 m | 10-100 m | > 100 m | Hakuna | Majani mafupi | Majani marefu | Majani elea | < 0.5 m | > 0.5 m | Hakuna | Wodogo | Wakubwa | Hakuna | Wadogo | Wakubwa | Hakuna | Wapo | |
| | | | | | | | | | | | | | | | | | | | | | | | | | | |
| | | | | | | | | | | | | | | | | | | | | | | | | | | |
| | | | | | | | | | | | | | | | | | | | | | | | | | | |
| | | | | | | | | | | | | | | | | | | | | | | | | | | |
| | | | | | | | | | | | | | | | | | | | | | | | | | | |
| | | | | | | | | | | | | | | | | | | | | | | | | | | |
| | | | | | | | | | | | | | | | | | | | | | | | | | | |
| | | | | | | | | | | | | | | | | | | | | | | | | | | |
| | | | | | | | | | | | | | | | | | | | | | | | | | | |
| | | | | | | | | | | | | | | | | | | | | | | | | | | |
| | | | | | | | | | | | | | | | | | | | | | | | | | | |
| | | | | | | | | | | | | | | | | | | | | | | | | | | |
| | | | | | | | | | | | | | | | | | | | | | | | | | | |
| | | | | | | | | | | | | | | | | | | | | | | | | | | |
| | | | | | | | | | | | | | | | | | | | | | | | | | | |
| | | | | | | | | | | | | | | | | | | | | | | | | | | |
| | | | | | | | | | | | | | | | | | | | | | | | | | | |
| | | | | | | | | | | | | | | | | | | | | | | | | | | |
| | | | | | | | | | | | | | | | | | | | | | | | | | | |
| | | | | | | | | | | | | | | | | | | | | | | | | | | |
| | | | | | | | | | | | | | | | | | | | | | | | | | | |
| | | | | | | | | | | | | | | | | | | | | | | | | | | |
| | | | | | | | | | | | | | | | | | | | | | | | | | | |
| | | | | | | | | | | | | | | | | | | | | | | | | | | |
| | | | | | | | | | | | | | | | | | | | | | | | | | | |
| | | | | | | | | | | | | | | | | | | | | | | | | | | |
| | | | | | | | | | | | | | | | | | | | | | | | | | | |
| | | | | | | | | | | | | | | | | | | | | | | | | | | |
| | | | | | | | | | | | | | | | | | | | | | | | | | | |
| | | | | | | | | | | | | | | | | | | | | | | | | | | |
| | | | | | | | | | | | | | | | | | | | | | | | | | | |
| | | | | | | | | | | | | | | | | | | | | | | | | | | |
| | | | | | | | | | | | | | | | | | | | | | | | | | | |
| | | | | | | | | | | | | | | | | | | | | | | | | | | |
| | | | | | | | | | | | | | | | | | | | | | | | | | | |

Jina La CORP: __________________________ __________________ Tarehe:______/______/______

Jina La Supervisor: __________________________________ Tarehe:_______/_______/_________

## Fomu ya mazalio ya mbu kwenye vyoo na makaro Ngazi ya kata

**Serial namba ya fomu hii** ______________________________

**Serial namba ilioko kwenye fomu ya ramani ya shina hili** ______________________________

**Namba ya shina ya pekee** ______________________________

Manisipaa:______________________ Kata:______________________ Mtaa:__________________________ Namba ya shina:_________________ Jina la Mjumbe:__________________________________________

**Aina ya zalio:** 1 - Choo cha shimo 2 - mashimo ya maji machafu 3 - Mashimo ya maji taka 4 - Mengineyo________________

| Namba ya Utambulisho wa ploti | Namba ya nyumba. | Namba ya zalio | Aina ya zalio | Maelezo kuhusu zalio | Maji yapo? | | Hali ya choo | | | Ukubwa wa zalio | | | Kina cha maji | | Hatua za viluwiluwi | | | Mabuu | | Maoni |
|---|---|---|---|---|---|---|---|---|---|---|---|---|---|---|---|---|---|---|---|---|
| | | | | | kavu | kina maji | Nzuri | Mbaya | Kimejaa | < 10 m | 10-100 m | > 100 m | < 0.5 m | > 0.5 m | Hakuna | Wadogo | Wakubwa | Yapo | Hakuna | |
| | | | | | | | | | | | | | | | | | | | | |
| | | | | | | | | | | | | | | | | | | | | |
| | | | | | | | | | | | | | | | | | | | | |
| | | | | | | | | | | | | | | | | | | | | |
| | | | | | | | | | | | | | | | | | | | | |
| | | | | | | | | | | | | | | | | | | | | |
| | | | | | | | | | | | | | | | | | | | | |
| | | | | | | | | | | | | | | | | | | | | |
| | | | | | | | | | | | | | | | | | | | | |
| | | | | | | | | | | | | | | | | | | | | |
| | | | | | | | | | | | | | | | | | | | | |
| | | | | | | | | | | | | | | | | | | | | |
| | | | | | | | | | | | | | | | | | | | | |
| | | | | | | | | | | | | | | | | | | | | |
| | | | | | | | | | | | | | | | | | | | | |
| | | | | | | | | | | | | | | | | | | | | |
| | | | | | | | | | | | | | | | | | | | | |
| | | | | | | | | | | | | | | | | | | | | |
| | | | | | | | | | | | | | | | | | | | | |
| | | | | | | | | | | | | | | | | | | | | |
| | | | | | | | | | | | | | | | | | | | | |
| | | | | | | | | | | | | | | | | | | | | |
| | | | | | | | | | | | | | | | | | | | | |
| | | | | | | | | | | | | | | | | | | | | |
| | | | | | | | | | | | | | | | | | | | | |
| | | | | | | | | | | | | | | | | | | | | |
| | | | | | | | | | | | | | | | | | | | | |
| | | | | | | | | | | | | | | | | | | | | |
| | | | | | | | | | | | | | | | | | | | | |
| | | | | | | | | | | | | | | | | | | | | |

Jina La CORP: _______________ _______________ Tarehe:______/______/_______

Jina La Supervisor: ___________________________________ Tarehe:______ /________/_______

# Municipal mosquito larval habitat spot check - Open habitats

**Serial number of this form** ____________________

**Serial number on the map form** ____________________

**Ten cell unit identifier** ____________________

Municipality:_____________________ Ward:________________________ MTAA:______________________ 10-cell unit:_____________________ 10-cell leader:________________________
GPS(UTM/WGS84): Northing_________________________ Easting_______________________________ Date of CORP's data sheet:________/________/__________
Serial number on the map form___________________________

| | Yes | No |
|---|---|---|
| Is there a map? | | |
| Is the map accurate? | | |
| Does map match city copy? | | |

**Habitat codes:**
1: Puddles/tire tracks
2: Swampy areas
3: Mangrove Swamp
4: Drain/Ditch
5: Construction pits/foundations/man-made holes
6: Water storage & any other man-made container
7: Rice paddy
8: Matuta
9: Other agriculture
10: Stream/river bed
11: Pond
12: Others (describe below)

| Plot ID | House No. | Habitat ID | Habitat type | Is habitat type correct? | Correct habitat type | Habitat found by the CORPs? 1=Yes 2=No | Habitat Description | Wet? | | Habitat perimeter | | | Plants | | | | Water depth | | Larval stage | | | | | | Pupae | | Comments |
|---|---|---|---|---|---|---|---|---|---|---|---|---|---|---|---|---|---|---|---|---|---|---|---|---|---|---|---|
| | | | | | | | | | | | | | | | | | | | Anoph. | | | Culex | | | | | |
| | | | | | | | | dry | contains water | < 10 m | 10-100 m | > 100 m | None | Short vegetation | Tall vegetation | Floating plants | < 0.5 m | > 0.5 m | Absent | Early | Late | Absent | Early | Late | Absent | Present | |
| | | | | | | | | | | | | | | | | | | | | | | | | | | | |
| | | | | | | | | | | | | | | | | | | | | | | | | | | | |
| | | | | | | | | | | | | | | | | | | | | | | | | | | | |
| | | | | | | | | | | | | | | | | | | | | | | | | | | | |
| | | | | | | | | | | | | | | | | | | | | | | | | | | | |
| | | | | | | | | | | | | | | | | | | | | | | | | | | | |
| | | | | | | | | | | | | | | | | | | | | | | | | | | | |
| | | | | | | | | | | | | | | | | | | | | | | | | | | | |
| | | | | | | | | | | | | | | | | | | | | | | | | | | | |
| | | | | | | | | | | | | | | | | | | | | | | | | | | | |
| | | | | | | | | | | | | | | | | | | | | | | | | | | | |
| | | | | | | | | | | | | | | | | | | | | | | | | | | | |
| | | | | | | | | | | | | | | | | | | | | | | | | | | | |
| | | | | | | | | | | | | | | | | | | | | | | | | | | | |
| | | | | | | | | | | | | | | | | | | | | | | | | | | | |
| | | | | | | | | | | | | | | | | | | | | | | | | | | | |
| | | | | | | | | | | | | | | | | | | | | | | | | | | | |
| | | | | | | | | | | | | | | | | | | | | | | | | | | | |
| | | | | | | | | | | | | | | | | | | | | | | | | | | | |
| | | | | | | | | | | | | | | | | | | | | | | | | | | | |
| | | | | | | | | | | | | | | | | | | | | | | | | | | | |
| | | | | | | | | | | | | | | | | | | | | | | | | | | | |
| | | | | | | | | | | | | | | | | | | | | | | | | | | | |
| | | | | | | | | | | | | | | | | | | | | | | | | | | | |
| | | | | | | | | | | | | | | | | | | | | | | | | | | | |
| | | | | | | | | | | | | | | | | | | | | | | | | | | | |
| | | | | | | | | | | | | | | | | | | | | | | | | | | | |
| | | | | | | | | | | | | | | | | | | | | | | | | | | | |
| | | | | | | | | | | | | | | | | | | | | | | | | | | | |
| | | | | | | | | | | | | | | | | | | | | | | | | | | | |
| | | | | | | | | | | | | | | | | | | | | | | | | | | | |
| | | | | | | | | | | | | | | | | | | | | | | | | | | | |

Inspectors signature:_________________________________ _______ Date of check :______/______/_______ Municipal Coordinators signature:_____________________________ Date of check :______/______/_______

# Municipal mosquito larval habitat spot check - Closed habitats

**Serial number of this form** ____________________

**Serial number on the map form** ____________________

**Ten cell unit identifier** ____________________

Municipality:______________________ Ward:_________________________ MTAA:________________________ 10-cell unit:______________________ 10-cell leader:_________________________

GPS(UTM/WGS84): Northing ________________________ Easting_____________________________

| | Yes | No |
|---|---|---|
| Is there a map? | | |
| Is the map accurate? | | |
| Does map match city copy? | | |

Serial number on the map form____________________________

**Habitat codes:** 1 - Pit Latrine 2 - Soakage Pit 3 - Septic Tank 4 - Other_____________________

| Plot ID | House No. | Habitat ID | Is habitat type correct? | Correct habitat type | Habitat found by the CORPs? 1=Yes 2=No | Habitat Description | Wet? | | Condition of the latrine | | | Habitat perimeter | | | Water depth | | Culicine stage | | | Pupae | | Comments |
|---|---|---|---|---|---|---|---|---|---|---|---|---|---|---|---|---|---|---|---|---|---|---|
| | | | | | | | dry | contains water | Good | Bad | Full | < 10 m | 10-100 m | > 10 m | < 0.5 m | > 0.5 m | None | Early | Late | Absent | Present | |
| | | | | | | | | | | | | | | | | | | | | | | |
| | | | | | | | | | | | | | | | | | | | | | | |
| | | | | | | | | | | | | | | | | | | | | | | |
| | | | | | | | | | | | | | | | | | | | | | | |
| | | | | | | | | | | | | | | | | | | | | | | |
| | | | | | | | | | | | | | | | | | | | | | | |
| | | | | | | | | | | | | | | | | | | | | | | |
| | | | | | | | | | | | | | | | | | | | | | | |
| | | | | | | | | | | | | | | | | | | | | | | |
| | | | | | | | | | | | | | | | | | | | | | | |
| | | | | | | | | | | | | | | | | | | | | | | |
| | | | | | | | | | | | | | | | | | | | | | | |
| | | | | | | | | | | | | | | | | | | | | | | |
| | | | | | | | | | | | | | | | | | | | | | | |
| | | | | | | | | | | | | | | | | | | | | | | |
| | | | | | | | | | | | | | | | | | | | | | | |
| | | | | | | | | | | | | | | | | | | | | | | |
| | | | | | | | | | | | | | | | | | | | | | | |
| | | | | | | | | | | | | | | | | | | | | | | |
| | | | | | | | | | | | | | | | | | | | | | | |
| | | | | | | | | | | | | | | | | | | | | | | |
| | | | | | | | | | | | | | | | | | | | | | | |
| | | | | | | | | | | | | | | | | | | | | | | |
| | | | | | | | | | | | | | | | | | | | | | | |
| | | | | | | | | | | | | | | | | | | | | | | |
| | | | | | | | | | | | | | | | | | | | | | | |
| | | | | | | | | | | | | | | | | | | | | | | |
| | | | | | | | | | | | | | | | | | | | | | | |

Inspectors signature:________________________________ _______ Date of check :______/______/_______

Municipal Coordinators signature:___________________ _______ Date of check :______/______/_______
