# Supplementary material for: A tool box for operational mosquito larval control: preliminary results and early lessons from the Urban Malaria Control Programme in Dar es Salaam, Tanzania
Source: Malar J. 2008 Jan 25;7:20. doi: 10.1186/1475-2875-7-20 (PMC2259364; doi:10.1186/1475-2875-7-20)
Supplement: Additional file 6 — Training presentation for larval surveillance. The document shows a training presentation for ward based staff on how to recognize mosquito larval habitats and how to characterise them according to the standard operating procedures. [file 1475-2875-7-20-S6.pdf]

LARVAL SURVEYS FOR OPEN HABITATS

The Urban Malaria Control Program (UMCP), Dar es Salaam

This document has been produced and made available by the Dar es Salaam Urban Malaria Control Programme. Contact: Urban Malaria Control Programme, City Medical Office of Health, City Council, P.O. Box 63320, Dar es Salaam, Tanzania, Phone: +255 22 212 1649

WARD LEVEL mosquito larval habitat survey - Open habitats

WARD LEVEL mosquito larval habitat survey - Open habitats

Serial number of this form________________________________

Serial number on the map form____________________________

Date:________/________/__________

Municipality:____________________________ Ward:___________________________ MTAA:______________________________ 10-cell unit:_________________

GPS(UTM/WGS84): Northing___________________ Easting_______________________ 10-cell leader:___________

Habitat codes:

1: Puddles&tire tracks	5: Construction pits/foundations/man-made holes	9: Other agriculture
2: Swampy areas	6: Water storage container	10: Stream/river bed
3: Mangrove Swamp	7: Rice paddy	11: Pond
4: Drain/Ditch	8: Matuta	12: Other (describe below)

New

Plot ID	Habitat ID	Habitat type	Same habitat type from last visit? 1=Yes 2=No 3=First visit	Previous habitat type	Habitat description	House number	Wet?		Habitat perimeter			Plants				Water depth		Larval stage						Pupae		Comments
																		Anoph.			Culex					
							dry	Contains water	< 10 m	10-100 m	> 100 m	None	Short vegetation	Tall vegetation	Floating plants	< 0.5 m	> 0.5 m	Absent	Early	Late	Absent	Early	Late	Absent	Present	

This document has been produced and made available by the Dar es Salaam Urban Malaria Control Programme. Contact: Urban Malaria Control Programme, City Medical Office of Health, City Council, P.O. Box 63320, Dar es Salaam, Tanzania, Phone: +255 22 212 1649

How to fill in the data sheets

- Plot No.
- House No.
- Habitat ID.

All **unique** and **continuous**

- example

This document has been produced and made available by the Dar es Salaam Urban Malaria Control Programme. Contact: Urban Malaria Control Programme, City Medical Office of Health, City Council, P.O. Box 63320, Dar es Salaam, Tanzania, Phone: +255 22 212 1649

2 habitat types in same plot

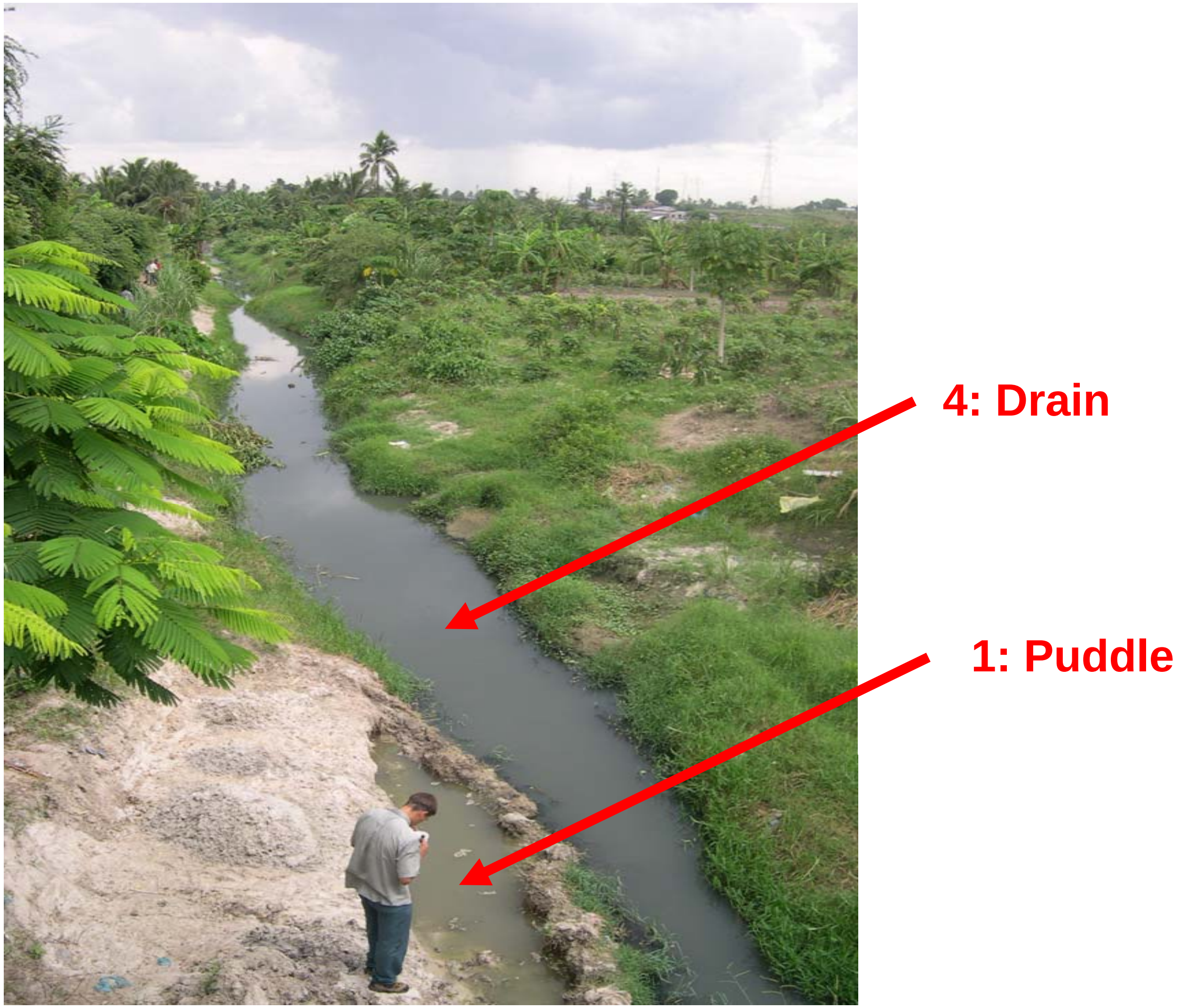

This document has been produced and made available by the Dar es Salaam Urban Malaria Control Programme. Contact: Urban Malaria Control Programme, City Medical Office of Health, City Council, P.O. Box 63320, Dar es Salaam, Tanzania, Phone: +255 22 212 1649

Ward level - data sheet

WARD LEVEL mosquito larval habitat survey - Open habitats

Serial number of this form_____0793____________________
Serial number on the map form__0891____________________
Date:___**7**____**/**__**4**_____**/**___**05**_____

Municipality:___Kinondoni________________ Ward:____Ndgumbi________________ MTAA:___Vigaeni______________________ 10-cell unit:_____38________________
GPS(UTM/WGS84): Northing____________________ Easting________________________ 10-cell leader:____Omary Bauari_______

	Yes	No
Is there a map	X	
Is the map up to d	X	
Is the map filed	X	

Habitat codes:

1: Puddles&tire tracks
2: Swampy areas
3: Mangrove Swamp
4: Drain/Ditch
5: Construction pits/foundations/man-made holes
6: Water storage container
7: Rice paddy
8: Matuta
9: Other agriculture
10: Stream/river bed
11: Pond
12: Other (describe below)

Plot ID	Habitat ID	Habitat type	Same habitat type from last visit? 1=Yes 2=No 3=First visit	Previous habitat type	Habitat description	House number	Wet? dry	Wet? Contains water	Habitat perimeter < 10 m	Habitat perimeter 10-100 m	Habitat perimeter > 100 m	Plants None	Plants Short vegetation	Plants Tall vegetation	Plants Floating plants	Water depth < 0.5 m	Water depth > 0.5 m	Larval stage Anoph. Absent	Larval stage Anoph. Early	Larval stage Anoph. Late	Larval stage Culex Absent	Larval stage Culex Early	Larval stage Culex Late	Pupae Absent	Pupae Present	Comments
7	1	4	1	4	large drain with flowing water			X			X		X	X			X	X			X			X		Irrigating local agriculture
7	2	1	1	1	small open shallow puddle			X	X			X				X			X		X			X		beside the drain

Habitat type = 1 to 12 codes

Habitat ID = how many different habitats in one plot

This document has been produced and made available by the Dar es Salaam Urban Malaria Control Programme. Contact: Urban Malaria Control Programme, City Medical Office of Health, City Council, P.O. Box 63320, Dar es Salaam, Tanzania, Phone: +255 22 212 1649

Dry or No habitat ???

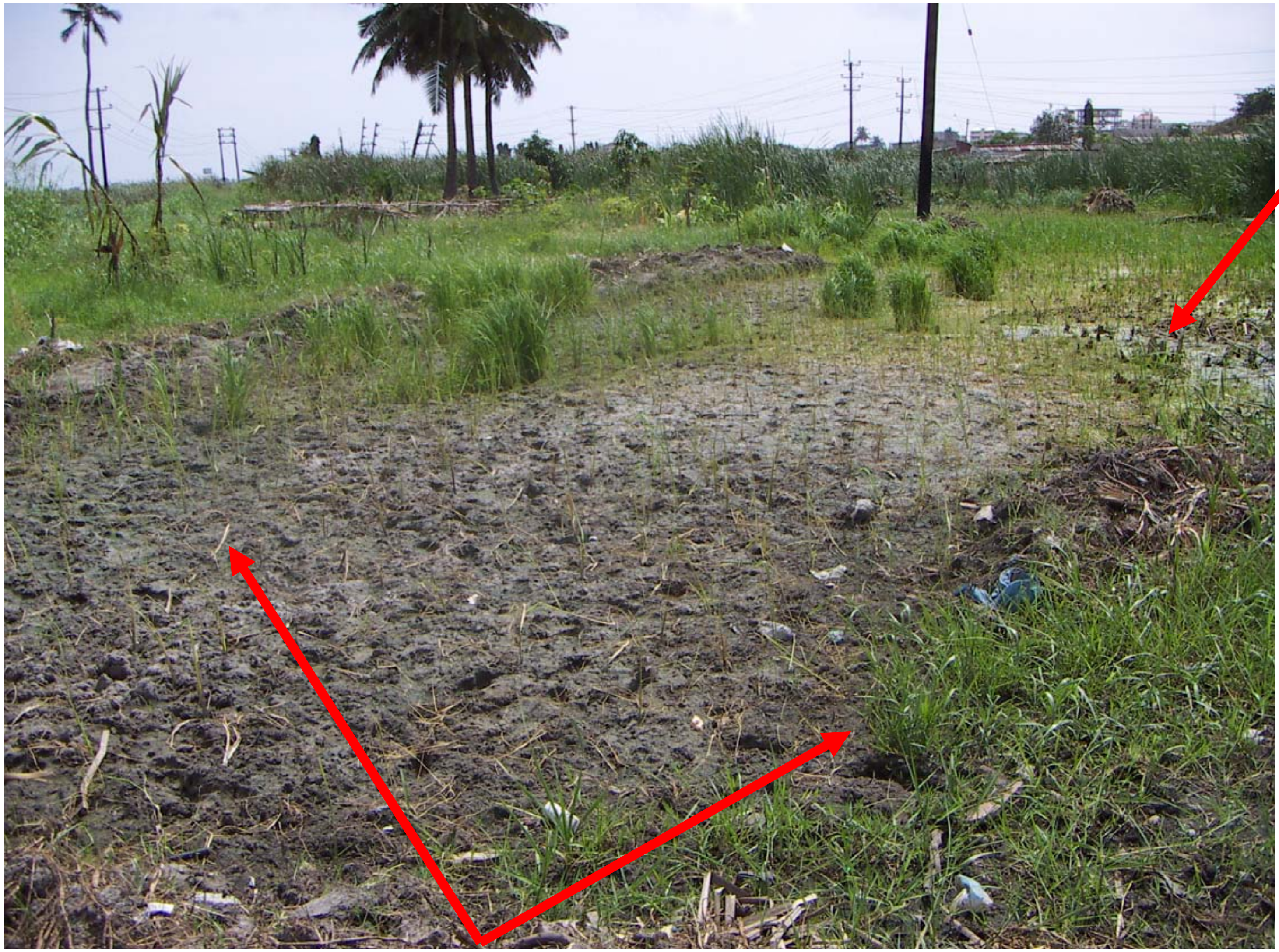

11: Pond

7: Rice field - dry habitat (with the potential of being a larval breeding site)

This document has been produced and made available by the Dar es Salaam Urban Malaria Control Programme. Contact: Urban Malaria Control Programme, City Medical Office of Health, City Council, P.O. Box 63320, Dar es Salaam, Tanzania, Phone: +255 22 212 1649

Wet / Dry / no habitat

- **<u>Both</u>** wet and dry sites need description

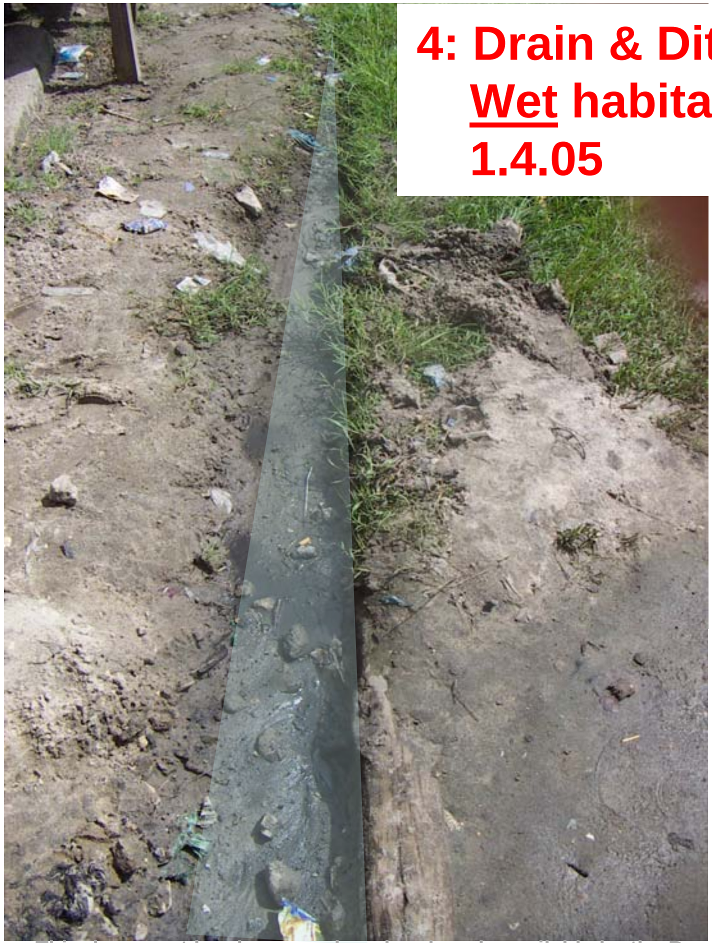

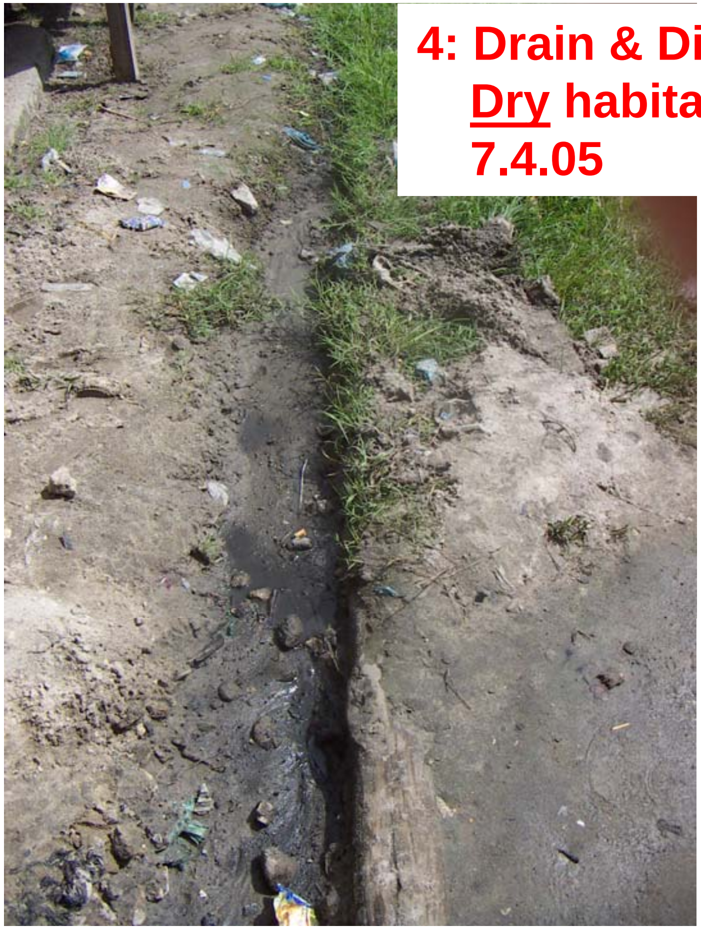

This document has been produced and made available by the Dar es Salaam Urban Malaria Control Programme. Contact: Urban Malaria Control Programme, City Medical Office of Health, City Council, P.O. Box 63320, Dar es Salaam, Tanzania, Phone: +255 22 212 1649

2 weeks of ward level data sheets

WARD LEVEL mosquito larval habitat survey - Open habitats

Serial number of this form_____0723___________________
Serial number on the map form__0333___________________
Date:___1____/__4_____/___05_____

Municipality:___Kinondoni_______________ Ward:____Ndgumbi________________ MTAA:___Vigaeni____________________ 10-cell unit:_____38_______________
GPS(UTM/WGS84): Northing___________________ Easting_________________________ 10-cell leader:____Omary Bauari_______

Habitat codes:

1: Puddles&tire tracks	5: Construction pits/foundations/man-made holes	9: Other agriculture
2: Swampy areas	6: Water storage container	10: Stream/river bed
3: Mangrove Swamp	7: Rice paddy	11: Pond
4: Drain/Ditch	8: Matuta	12: Other (describe below)

Plot ID	Habitat ID	Habitat type	Same habitat type fr last visit? 1=Yes 2: 3=First visit	Previous habitat ty	Habitat description	House number	Wet? dry	Wet? Contains water	Habitat perimeter < 10 m	Habitat perimeter 10-100 m	Habitat perimeter > 100 m	Plants None	Plants Short vegetation	Plants Tall vegetation	Plants Floating plants	Water depth < 0.5 m	Water depth > 0.5 m	Larval stage Anoph. Absent	Larval stage Anoph. Early	Larval stage Anoph. Late	Larval stage Culex Absent	Larval stage Culex Early	Larval stage Culex Late	Pupae Absent	Pupae Present	Comments
3	1	4	1	4	water flowing = water tap on	22		X		X			X			X		X				X		X		inbetween the houses

Same code = same site habitat & no more man-made construction

WARD LEVEL mosquito larval habitat survey - Open habitats

Serial number of this form_____0456___________________
Serial number on the map form__0333___________________
Date:___7____/__4_____/___05_____

Municipality:___Kinondoni_______________ Ward:____Ndgumbi________________ MTAA:___Vigaeni____________________ 10-cell unit:_____38_______________
GPS(UTM/WGS84): Northing___________________ Easting_________________________ 10-cell leader:____Omary Bauari_______

Full habitat description even if dry

Habitat codes:

1: Puddles&tire tracks	5: Construction pits/foundations/man-made holes	9: Other agriculture
2: Swampy areas	6: Water storage container	10: Stream/river bed
3: Mangrove Swamp	7: Rice paddy	11: Pond
4: Drain/Ditch	8: Matuta	12: Other (describe below)

Plot ID	Habitat ID	Habitat type	Same habitat type last visit? 1=Yes 3=First visit	Previous habitat	Habitat description	House number	Wet? dry	Wet? Contains water	Habitat perimeter < 10 m	Habitat perimeter 10-100 m	Habitat perimeter > 100 m	Plants None	Plants Short vegetation	Plants Tall vegetation	Plants Floating plants	Water depth < 0.5 m	Water depth > 0.5 m	Larval stage Anoph. Absent	Larval stage Anoph. Early	Larval stage Anoph. Late	Larval stage Culex Absent	Larval stage Culex Early	Larval stage Culex Late	Pupae Absent	Pupae Present	Comments
3	1	4	2	4	water tap turned off this week	22	X																			inbetween the houses

This document has been produced and made available by the Dar es Salaam Urban Malaria Control Programme. Contact: Urban Malaria Control Programme, City Medical Office of Health, City Council, P.O. Box 63320, Dar es Salaam, Tanzania, Phone: +255 22 212 1649

Habitat perimeter (m)

- walk around and **count your steps**
- one **step** = one **meter** (1m)

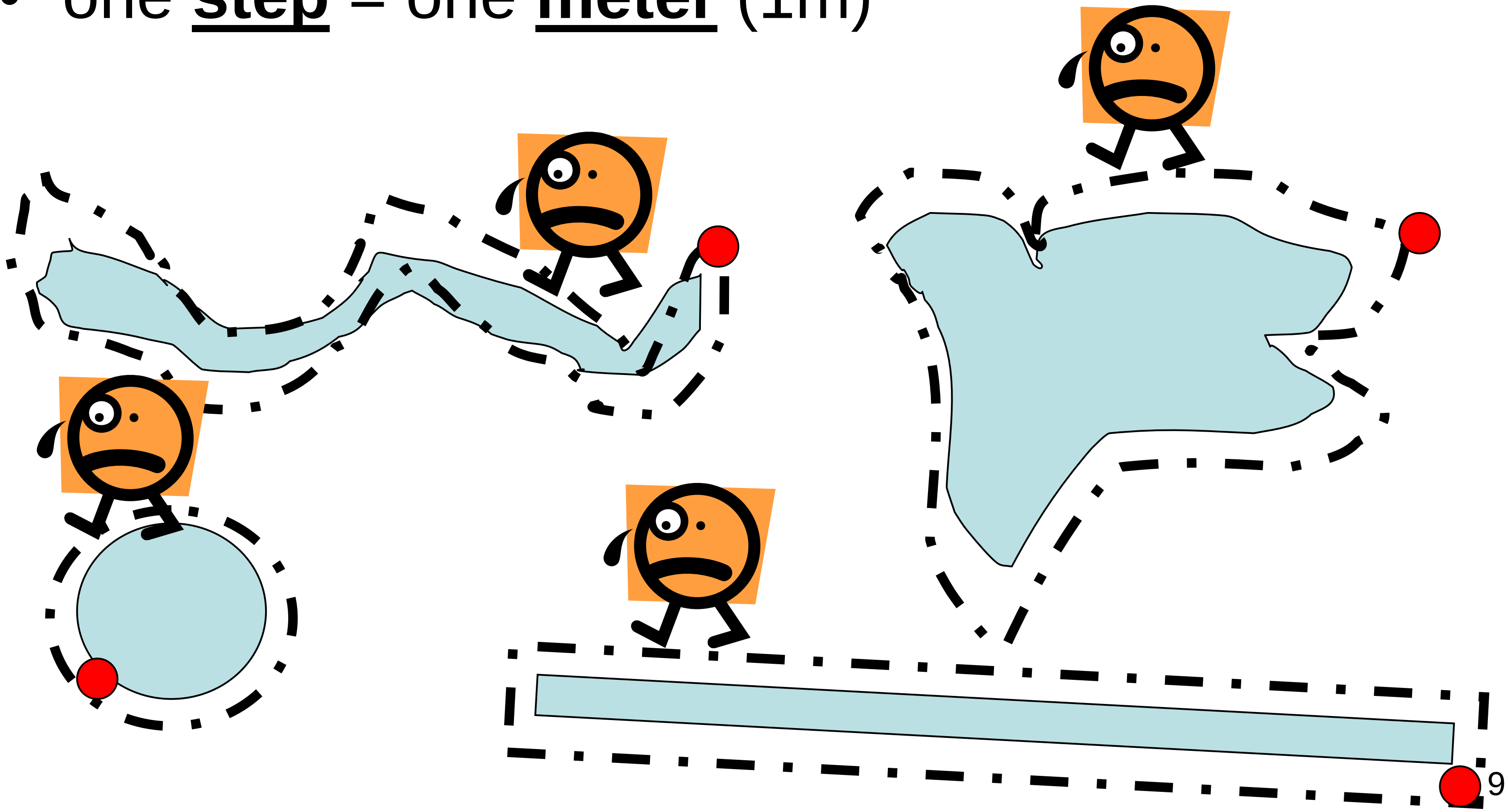

This document has been produced and made available by the Dar es Salaam Urban Malaria Control Programme. Contact: Urban Malaria Control Programme, City Medical Office of Health, City Council, P.O. Box 63320, Dar es Salaam, Tanzania, Phone: +255 22 212 1649

Plants Height

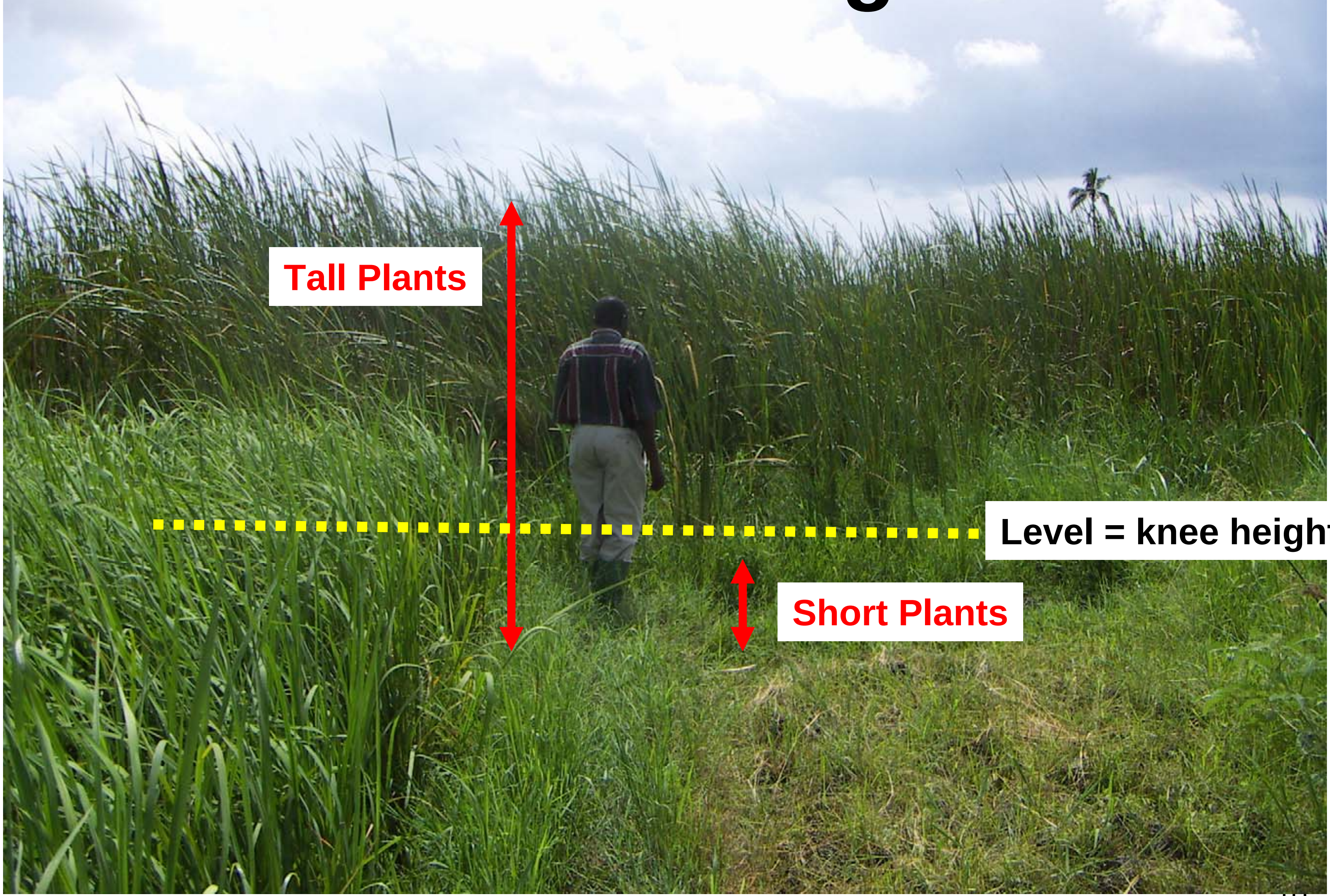

This document has been produced and made available by the Dar es Salaam Urban Malaria Control Programme. Contact: Urban Malaria Control Programme, City Medical Office of Health, City Council, P.O. Box 63320, Dar es Salaam, Tanzania, Phone: +255 22 212 1649

Floating Plants

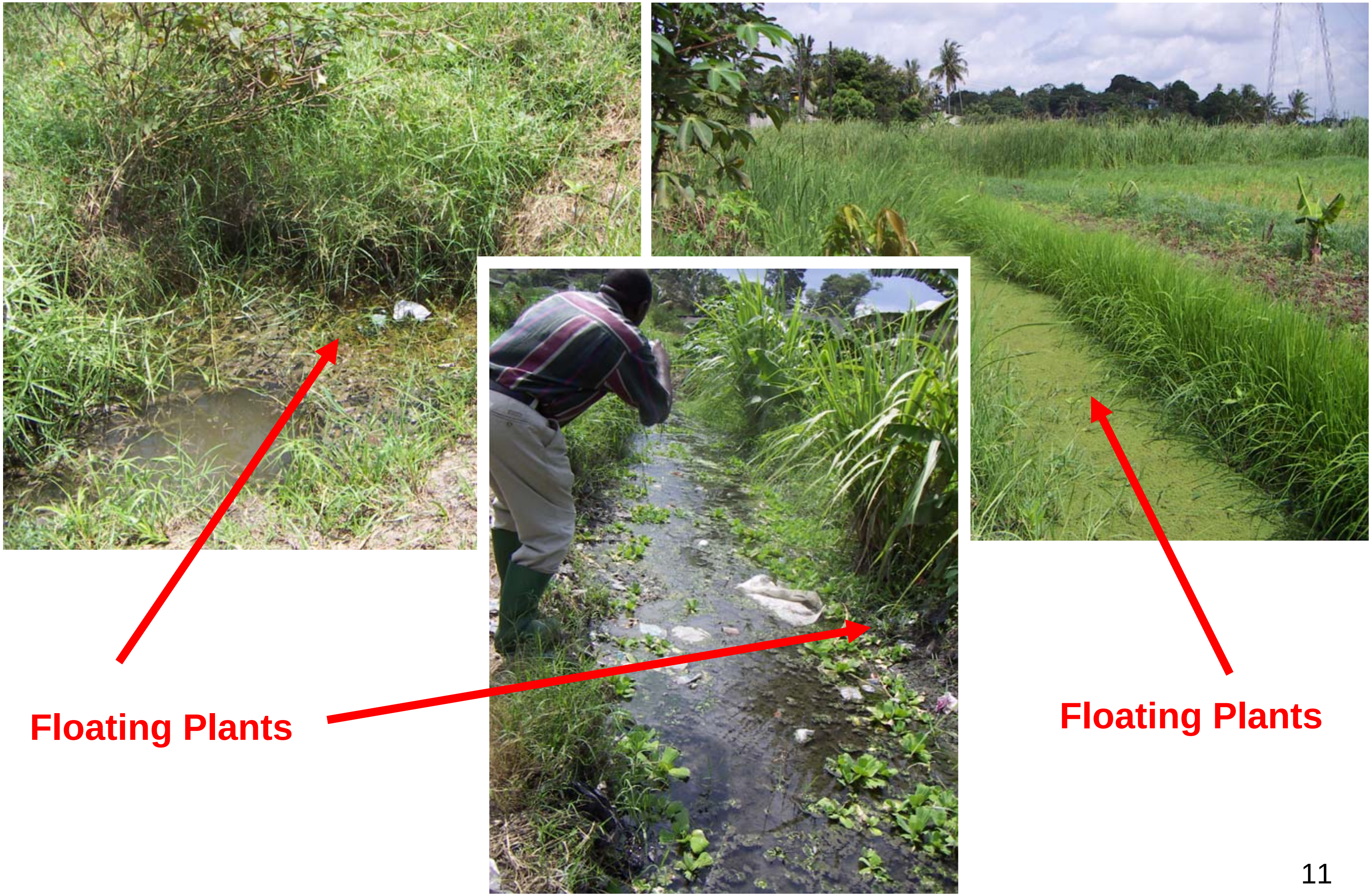

This document has been produced and made available by the Dar es Salaam Urban Malaria Control Programme. Contact: Urban Malaria Control Programme, City Medical Office of Health, City Council, P.O. Box 63320, Dar es Salaam, Tanzania, Phone: +255 22 212 1649

Water depth

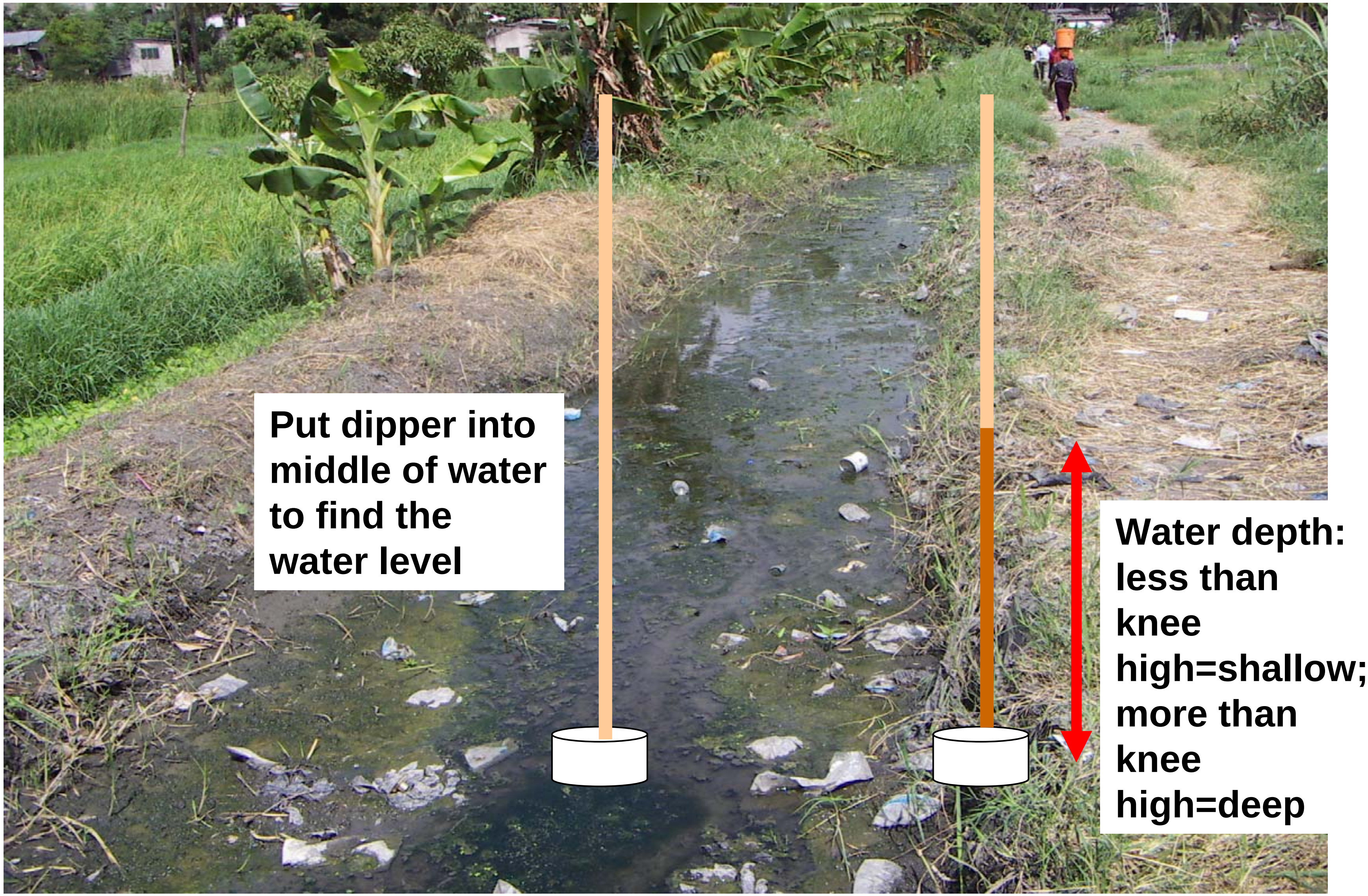
Put dipper into middle of water to find the water level
Water depth: less than knee high=shallow; more than knee high=deep

Mosquito types

- Mosquitoes breed in **all types of water**, it is important to **check all** water bodies during a larval survey.

- **Anopheles** larvae and **Culex** larvae physically distinguishable but the pupae are **not** physically distinguishable

This document has been produced and made available by the Dar es Salaam Urban Malaria Control Programme. Contact: Urban Malaria Control Programme, City Medical Office of Health, City Council, P.O. Box 63320, Dar es Salaam, Tanzania, Phone: +255 22 212 1649

Mosquito types

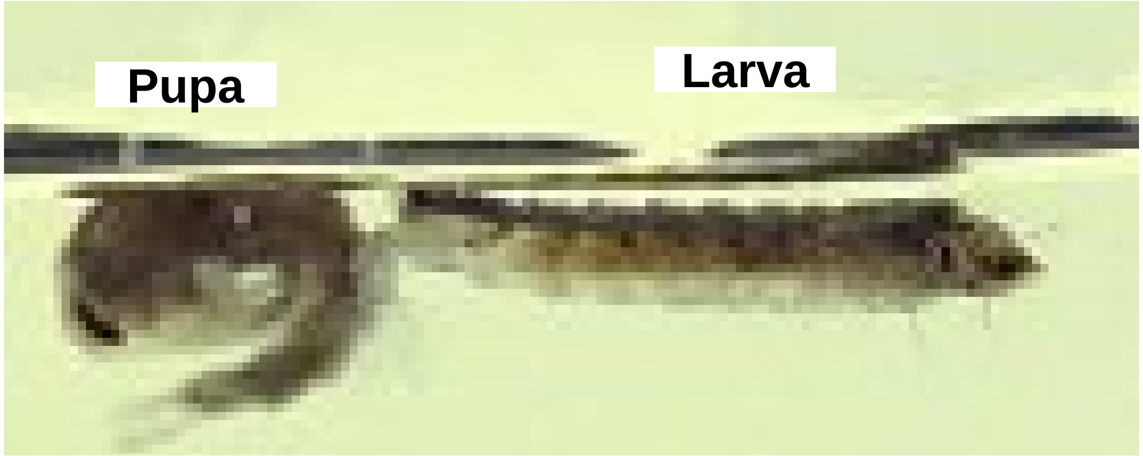

***Anopheles* larva**
has no obvious siphon and lies parallel to the water surface

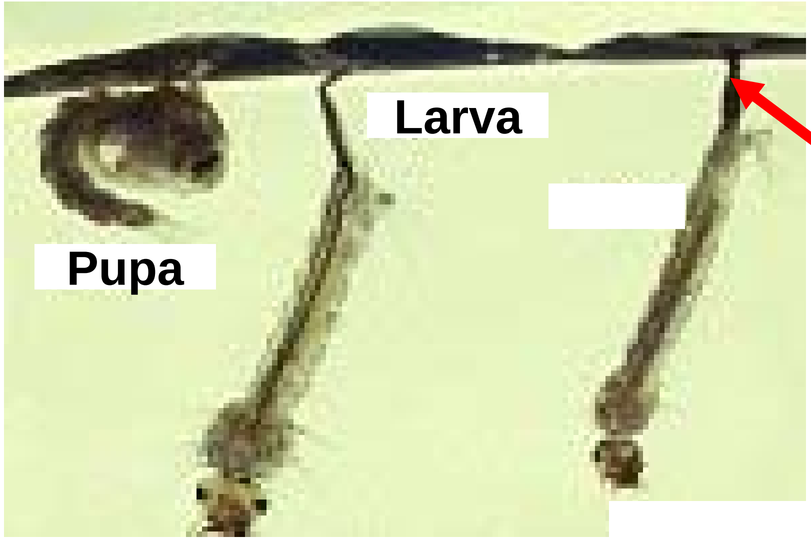

Straw-like siphon

***Culex* larva**
hang down from the water surface at an angle

This document has been produced and made available by the Dar es Salaam Urban Malaria Control Programme. Contact: Urban Malaria Control Programme, City Medical Office of Health, City Council, P.O. Box 63320, Dar es Salaam, Tanzania, Phone: +255 22 212 1649

Signature and date from inspectors

WARD LEVEL mosquito larval habitat survey - Open habitats

Serial number of this form_______________________________

Serial number on the map form___________________________

Date:________/________/__________

Municipality:____________________________ Ward:___________________________ MTAA:______________________________ 10-cell unit:_______________________

GPS(UTM/WGS84): Northing___________________ Easting_______________________

Habitat codes:

10-cell leader:___________________________________

1: Puddles&tire tracks
2: Swampy areas
3: Mangrove Swamp
4: Drain/Ditch
5: Construction pits/foundations/man-made holes
6: Water storage container
7: Rice paddy
8: Matuta
9: Other agriculture
10: Stream/river bed
11: Pond
12: Other (describe below)

Plot ID	Habitat ID	Habitat type	Same habitat type from last visit? 1=Yes 2=No 3=First visit	Previous habitat type	Habitat description	House number	Wet?		Habitat perimeter			Plants			Water depth		Larval stage						Pupae		Comments
																	Anoph.			Culex					
							dry	Contains water	< 10 m	10-100 m	> 100 m	None	Tall vegetation	Floating plants	< 0.5 m	> 0.5 m	Absent	Early	Late	Absent	Early	Late	Absent	Present	

CORPS signature Date

Inspectors signature Date

This document has been produced and made available by the Dar es Salaam Urban Malaria Control Programme. Contact: Urban Malaria Control Programme, City Medical Office of Health, City Council, P.O. Box 63320, Dar es Salaam, Tanzania, Phone: +255 22 212 1649

Habitat type = 12 codes

1: Puddles and Tyre Tracks
2: Swampy Areas
3: Mangrove swamp
4: Drains and Ditch
5: Construction pits, foundations and man-made holes
6: Water storage or other Man-made containers:
7: Rice paddy (Rice field)
8: Matuta
9: Other Agriculture
10: Stream and River beds
11: Ponds
12: Others (please describe them)

This document has been produced and made available by the Dar es Salaam Urban Malaria Control Programme. Contact: Urban Malaria Control Programme, City Medical Office of Health, City Council, P.O. Box 63320, Dar es Salaam, Tanzania, Phone: +255 22 212 1649

Is the site natural or man-made?

Natural
2: Swampy Areas
3: Mangrove swamp
10: Stream and River beds
11: Ponds

Man-made
1: Puddles and Tyre Tracks
4: Drains and Ditches
5: Construction pits, foundations, man-made holes
6: Water storage or other man-made containers
7: Rice paddy (Rice field)
8: Matuta
9: Other Agriculture

Next slide

Is it freshwater or salt water?

Freshwater
2: Swampy Areas
10: Stream and River
11: Ponds

Saltwater
3: Mangrove swamp

Is the water stagnant or flowing or should it be flowing?

Stagnant
2: Swampy Areas
11: Ponds

Flowing
10: Stream and River

Drain is straight and man-made and river meanders and is natural

This document has been produced and made available by the Dar es Salaam Urban Malaria Control Programme. Contact: Urban Malaria Control Programme, City Medical Office of Health, City Council, P.O. Box 63320, Dar es Salaam, Tanzania, Phone: +255 22 212 1649

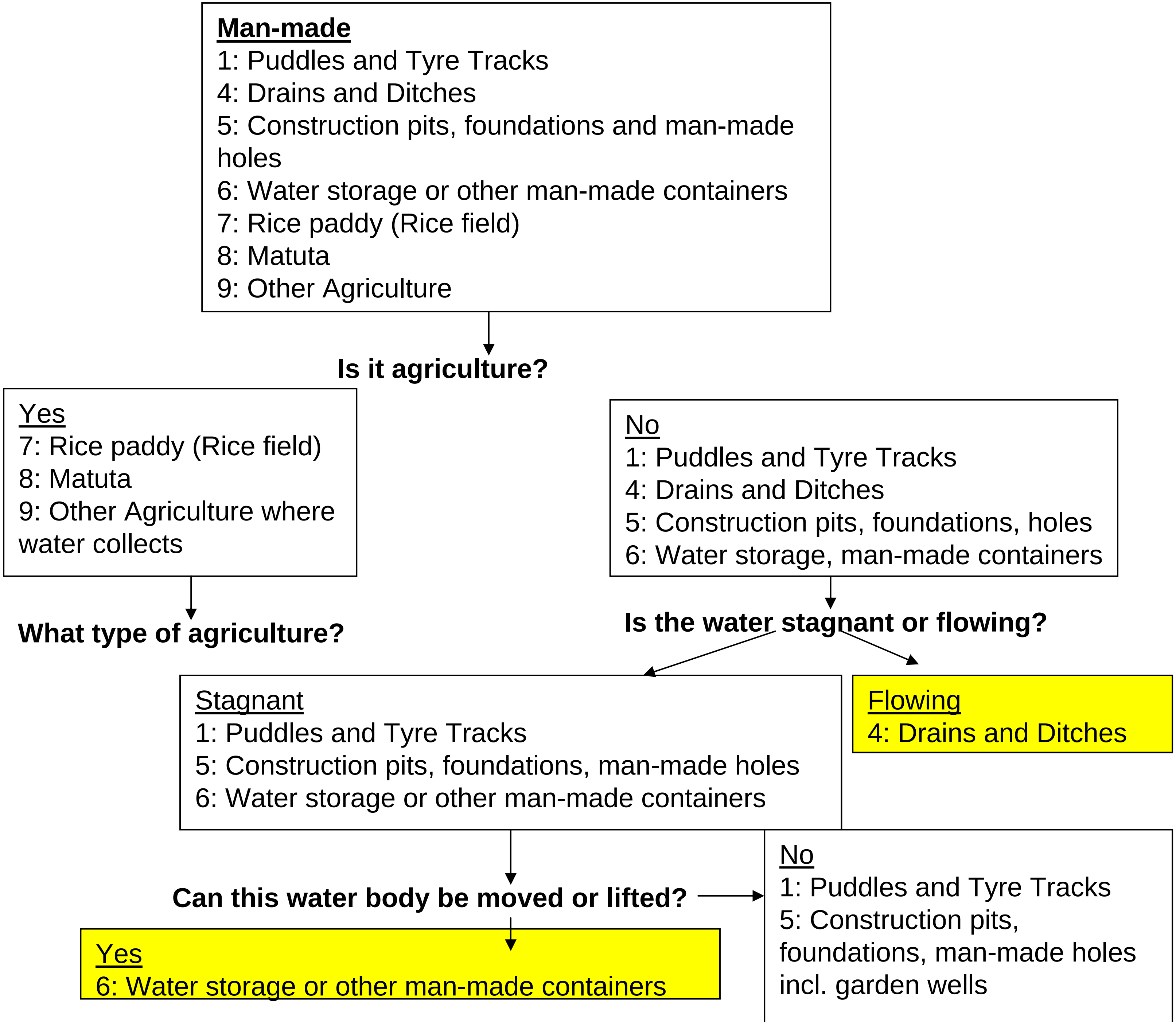
Man-made
1: Puddles and Tyre Tracks
4: Drains and Ditches
5: Construction pits, foundations and man-made holes
6: Water storage or other man-made containers
7: Rice paddy (Rice field)
8: Matuta
9: Other Agriculture
Is it agriculture?
Yes
7: Rice paddy (Rice field)
8: Matuta
9: Other Agriculture where water collects
What type of agriculture?
No
1: Puddles and Tyre Tracks
4: Drains and Ditches
5: Construction pits, foundations, holes
6: Water storage, man-made containers
Is the water stagnant or flowing?
Stagnant
1: Puddles and Tyre Tracks
5: Construction pits, foundations, man-made holes
6: Water storage or other man-made containers
Flowing
4: Drains and Ditches
Can this water body be moved or lifted?
Yes
6: Water storage or other man-made containers
No
1: Puddles and Tyre Tracks
5: Construction pits, foundations, man-made holes incl. garden wells

1: Puddles and Tyre Tracks

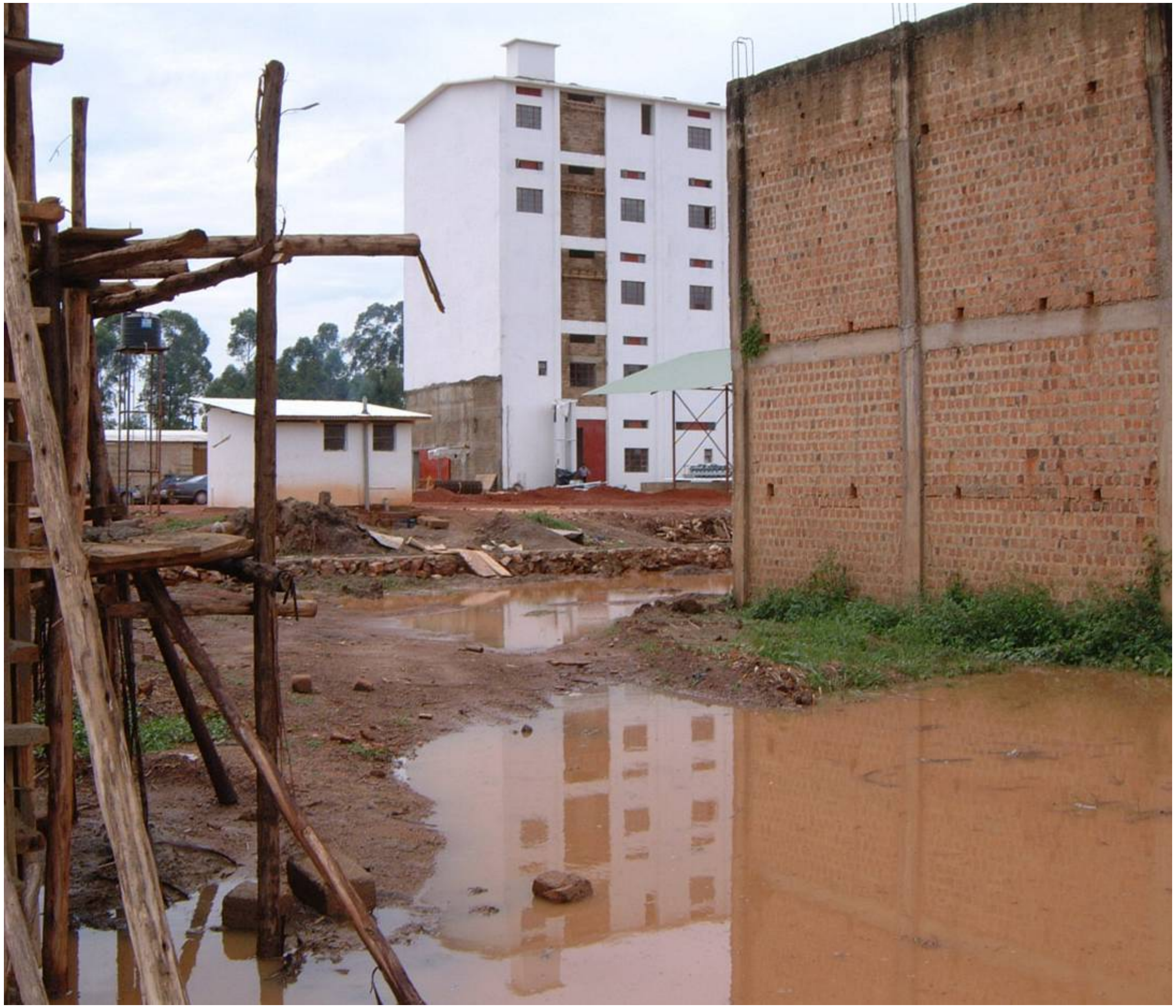

This document has been produced and made available by the Dar es Salaam Urban Malaria Control Programme. Contact: Urban Malaria Control Programme, City Medical Office of Health, City Council, P.O. Box 63320, Dar es Salaam, Tanzania, Phone: +255 22 212 1649

1: Puddles and Tyre Tracks

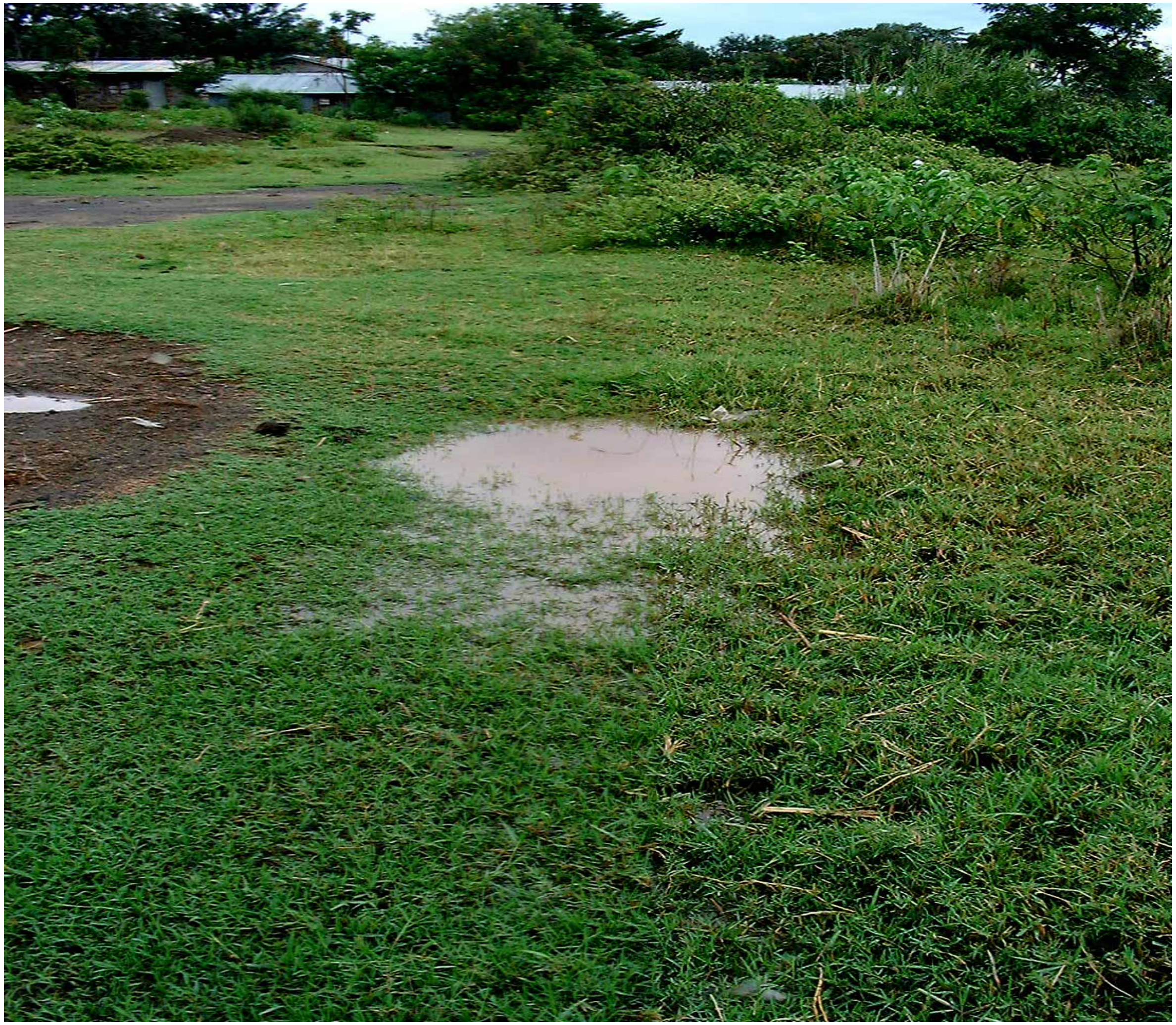

This document has been produced and made available by the Dar es Salaam Urban Malaria Control Programme. Contact: Urban Malaria Control Programme, City Medical Office of Health, City Council, P.O. Box 63320, Dar es Salaam, Tanzania, Phone: +255 22 212 1649

2: Swampy Areas

- very high ground water table
- water present always or most of the year
- water source = ground water & rainwater
- often border a large water body e.g. river
- usually depth >0.5 m
- often tall reeds, short grass or / & floating plants

This document has been produced and made available by the Dar es Salaam Urban Malaria Control Programme. Contact: Urban Malaria Control Programme, City Medical Office of Health, City Council, P.O. Box 63320, Dar es Salaam, Tanzania, Phone: +255 22 212 1649

2: Swampy Areas

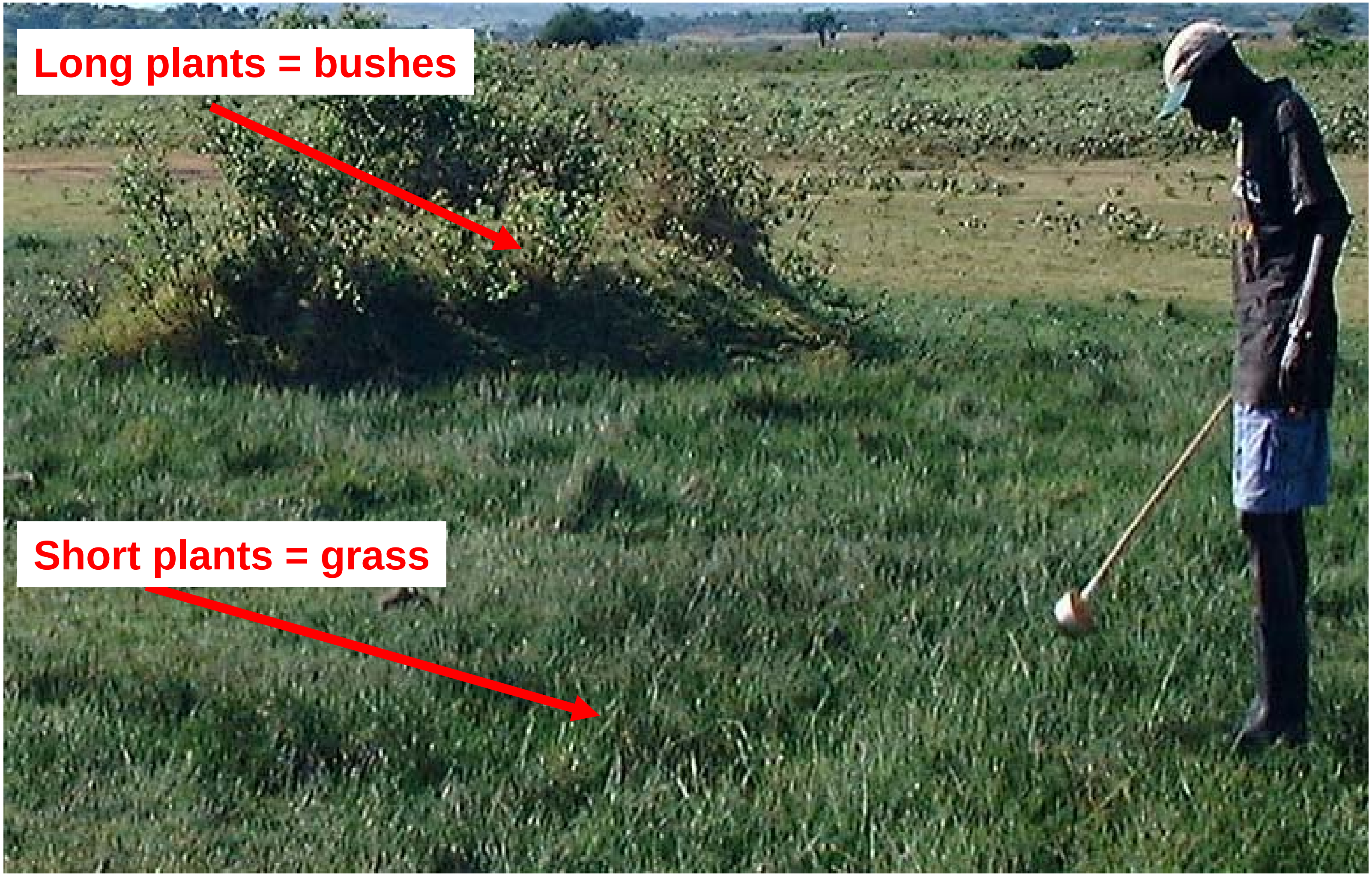

This document has been produced and made available by the Dar es Salaam Urban Malaria Control Programme. Contact: Urban Malaria Control Programme, City Medical Office of Health, City Council, P.O. Box 63320, Dar es Salaam, Tanzania, Phone: +255 22 212 1649

2: Swampy Areas

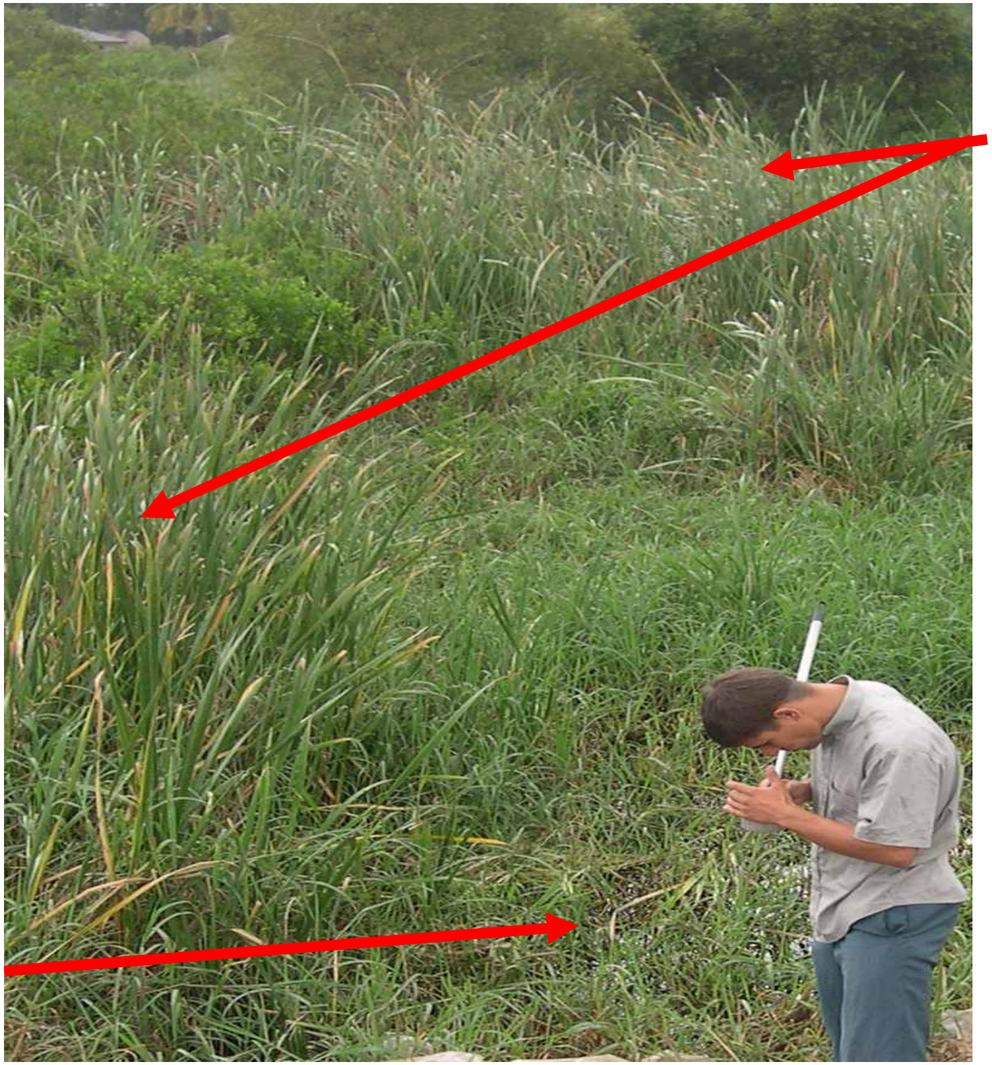

Long plants = reeds

Short plants = grass

This document has been produced and made available by the Dar es Salaam Urban Malaria Control Programme. Contact: Urban Malaria Control Programme, City Medical Office of Health, City Council, P.O. Box 63320, Dar es Salaam, Tanzania, Phone: +255 22 212 1649

3: Mangrove swamp

- usually near the sea = **salty** water from the sea
- mangrove trees growing with water underneath
- mangrove trees **roots** exposed
- water is tidal, when **tide out**:

  - small pools
  - crab holes in mud
  - shells on mangrove tree barks

This document has been produced and made available by the Dar es Salaam Urban Malaria Control Programme. Contact: Urban Malaria Control Programme, City Medical Office of Health, City Council, P.O. Box 63320, Dar es Salaam, Tanzania, Phone: +255 22 212 1649

3: Mangrove swamp

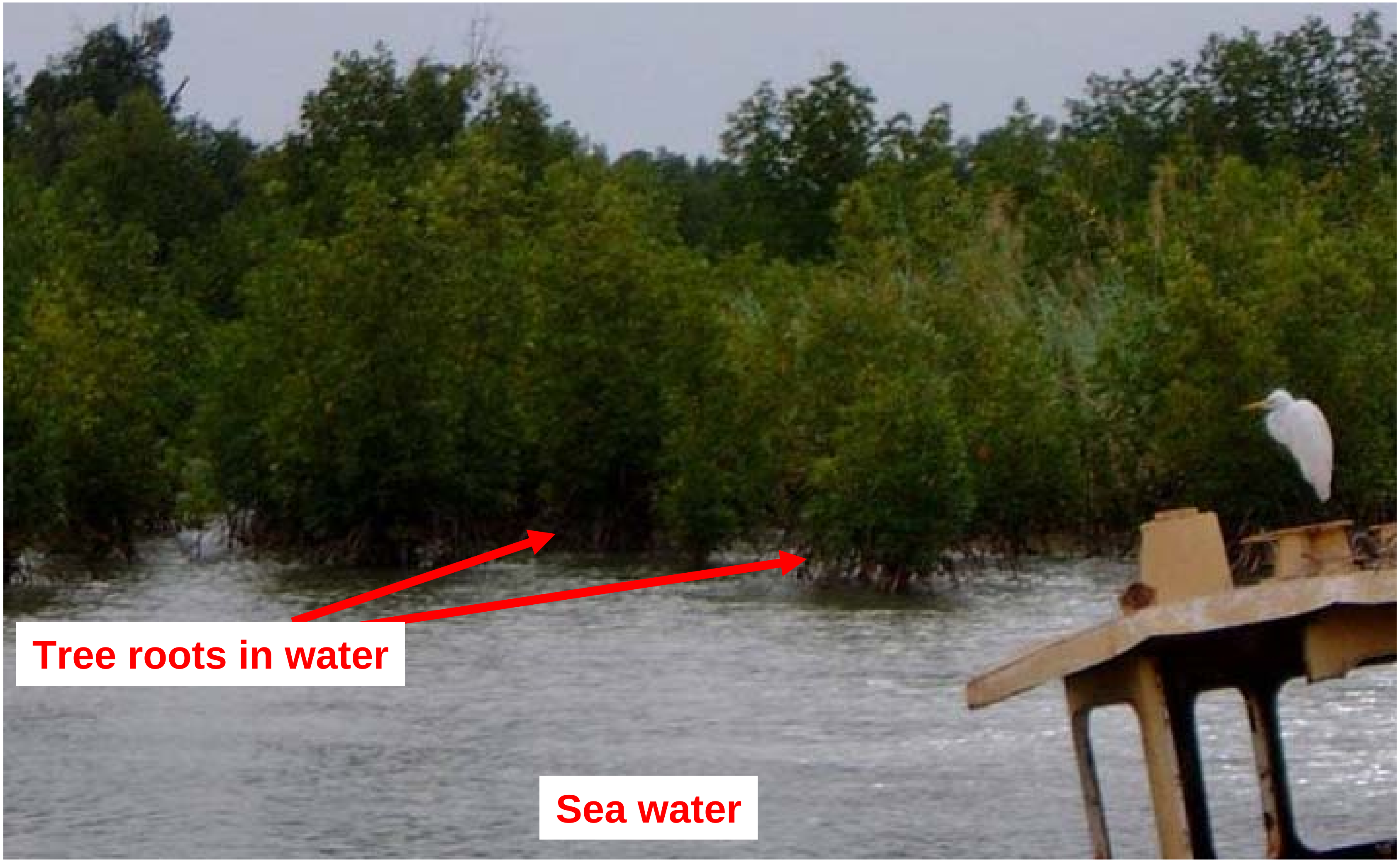

This document has been produced and made available by the Dar es Salaam Urban Malaria Control Programme. Contact: Urban Malaria Control Programme, City Medical Office of Health, City Council, P.O. Box 63320, Dar es Salaam, Tanzania, Phone: +255 22 212 1649

3: Mangrove swamp

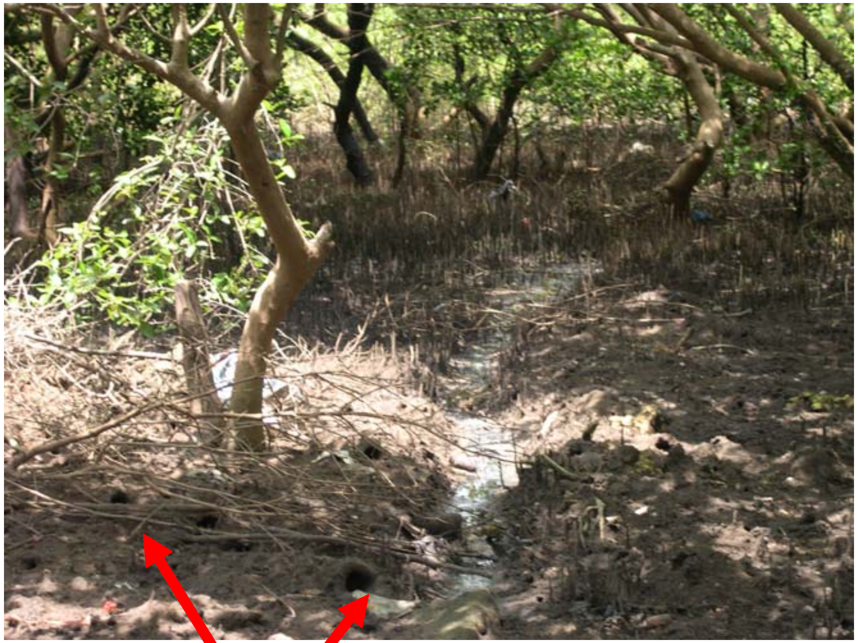

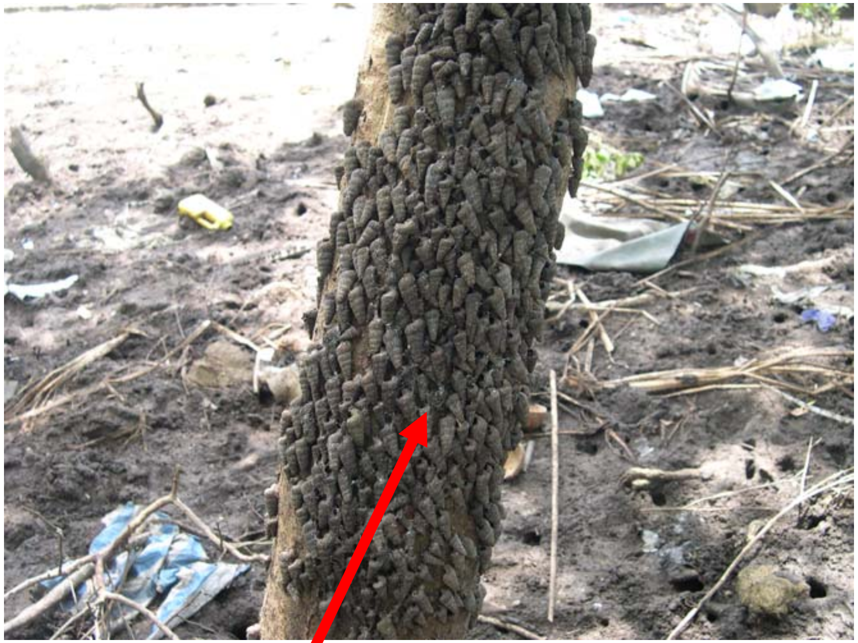

Crab holes

Sea shells on the mangrove tree

This document has been produced and made available by the Dar es Salaam Urban Malaria Control Programme. Contact: Urban Malaria Control Programme, City Medical Office of Health, City Council, P.O. Box 63320, Dar es Salaam, Tanzania, Phone: +255 22 212 1649

4: Drains and Ditches

- man-made
- Usually getting rid of water **or** to irrigate
- flowing water

  or if blocked with litter = stagnant water
- can be cement lined **or** just be dug in the ground

This document has been produced and made available by the Dar es Salaam Urban Malaria Control Programme. Contact: Urban Malaria Control Programme, City Medical Office of Health, City Council, P.O. Box 63320, Dar es Salaam, Tanzania, Phone: +255 22 212 1649

4: Drains and Ditches

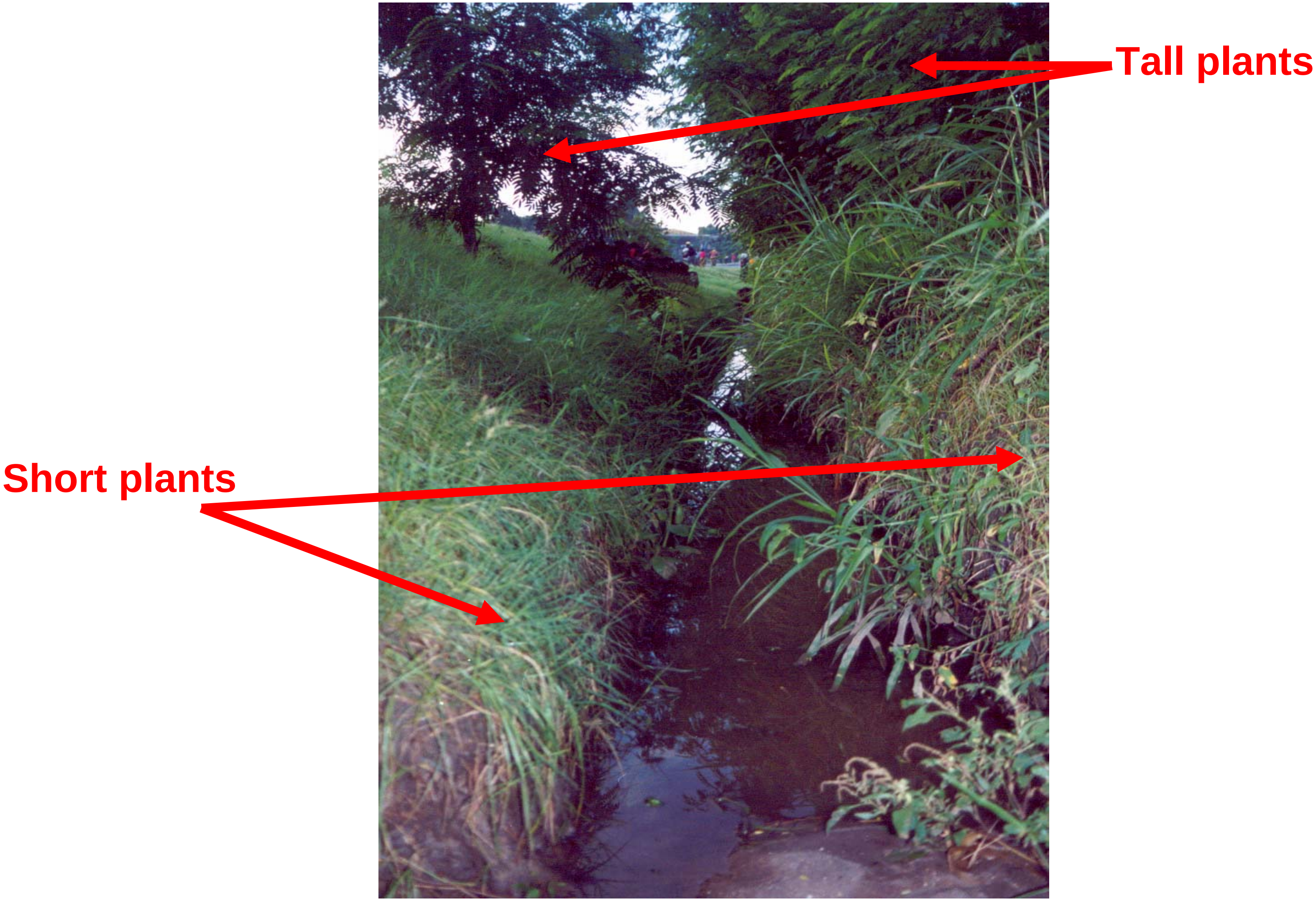

This document has been produced and made available by the Dar es Salaam Urban Malaria Control Programme. Contact: Urban Malaria Control Programme, City Medical Office of Health, City Council, P.O. Box 63320, Dar es Salaam, Tanzania, Phone: +255 22 212 1649

4: Drains and Ditches

Dry Habitat

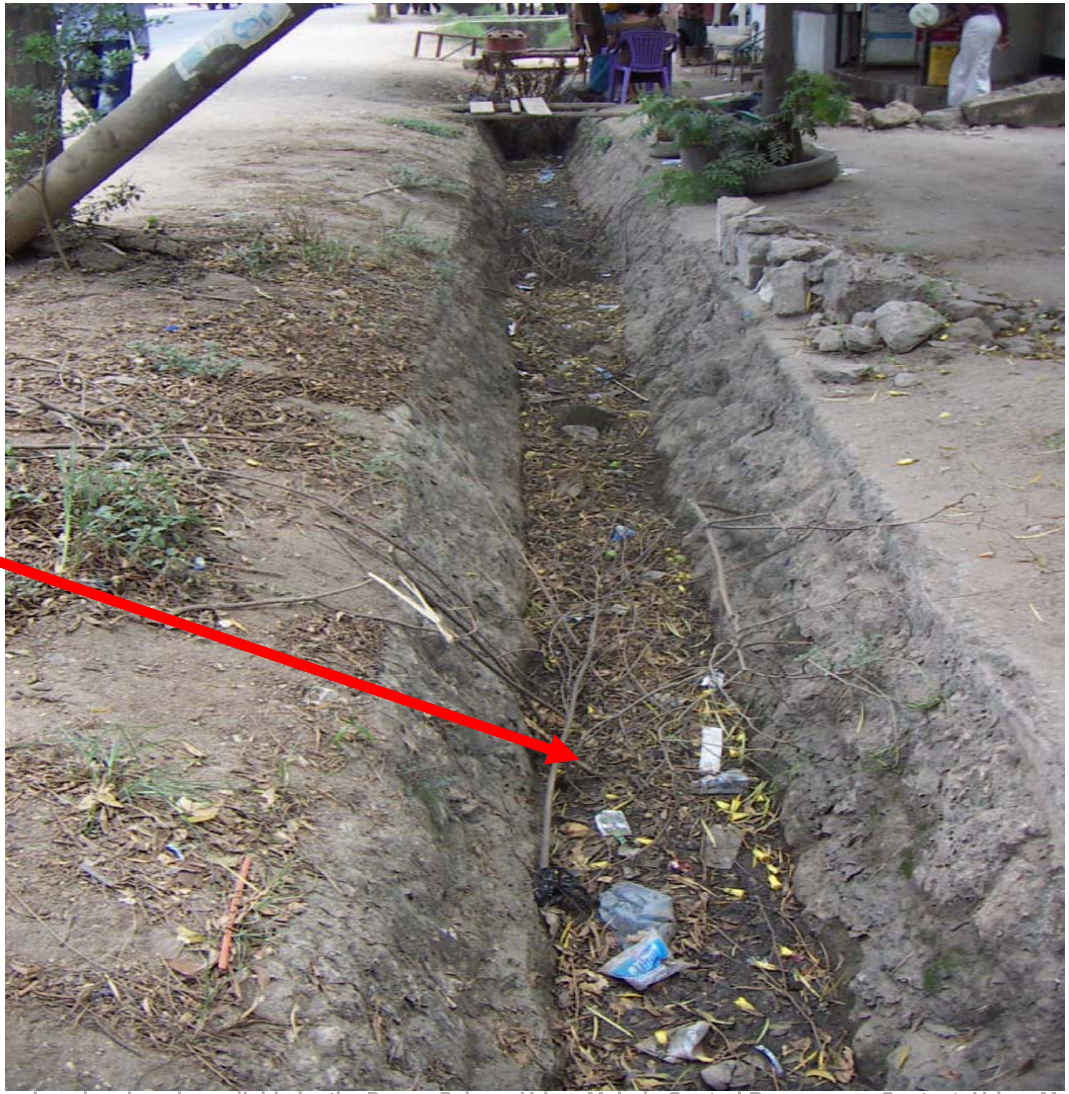

This document has been produced and made available by the Dar es Salaam Urban Malaria Control Programme. Contact: Urban Malaria Control Programme, City Medical Office of Health, City Council, P.O. Box 63320, Dar es Salaam, Tanzania, Phone: +255 22 212 1649

5: Construction pits, foundations and man-made holes

- small to medium sized
- man-made habitats
- stagnant water
- water source = rain or ground water (garden wells), or filled by people
- function to collect water
- habitats in the ground - **<u>not</u>** moveable

This document has been produced and made available by the Dar es Salaam Urban Malaria Control Programme. Contact: Urban Malaria Control Programme, City Medical Office of Health, City Council, P.O. Box 63320, Dar es Salaam, Tanzania, Phone: +255 22 212 1649

5: Construction pits, foundations and man-made holes

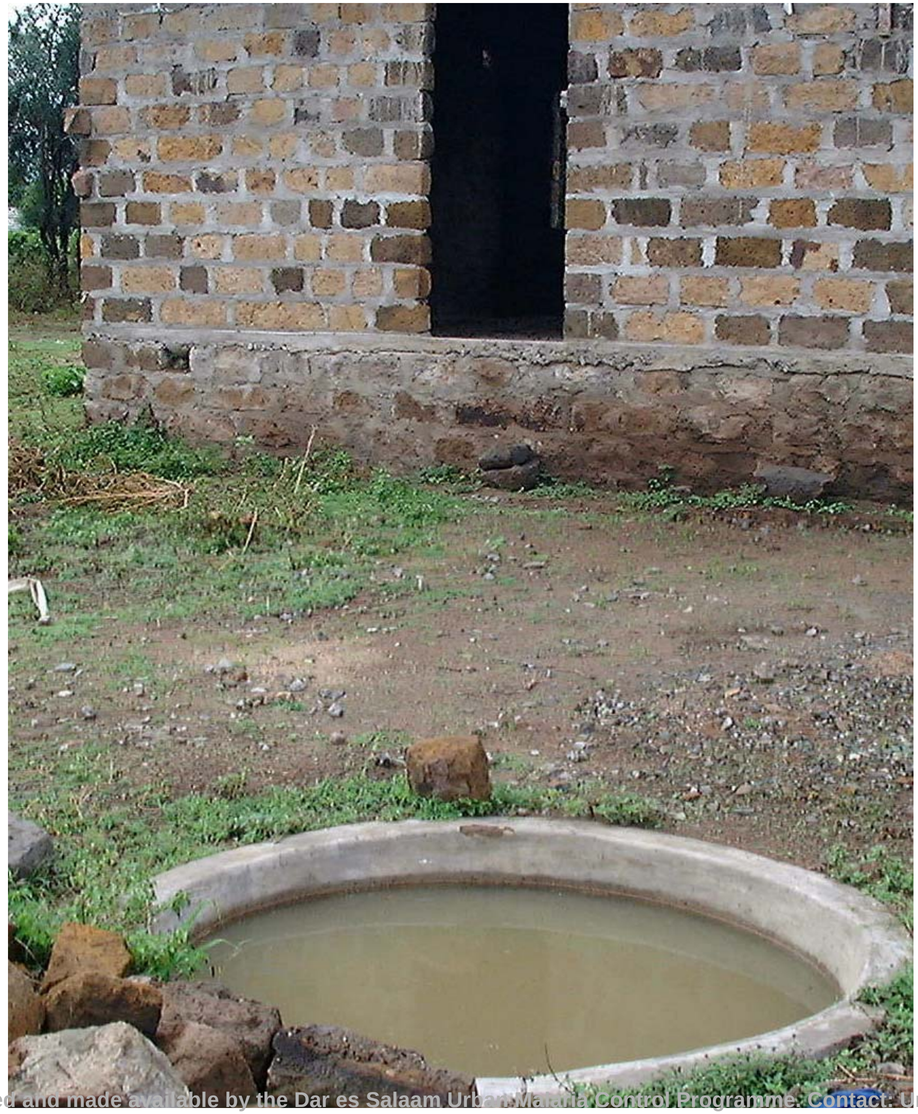

This document has been produced and made available by the Dar es Salaam Urban Malaria Control Programme. Contact: Urban Malaria Control Programme, City Medical Office of Health, City Council, P.O. Box 63320, Dar es Salaam, Tanzania, Phone: +255 22 212 1649

5: Construction pits, foundations and man-made holes

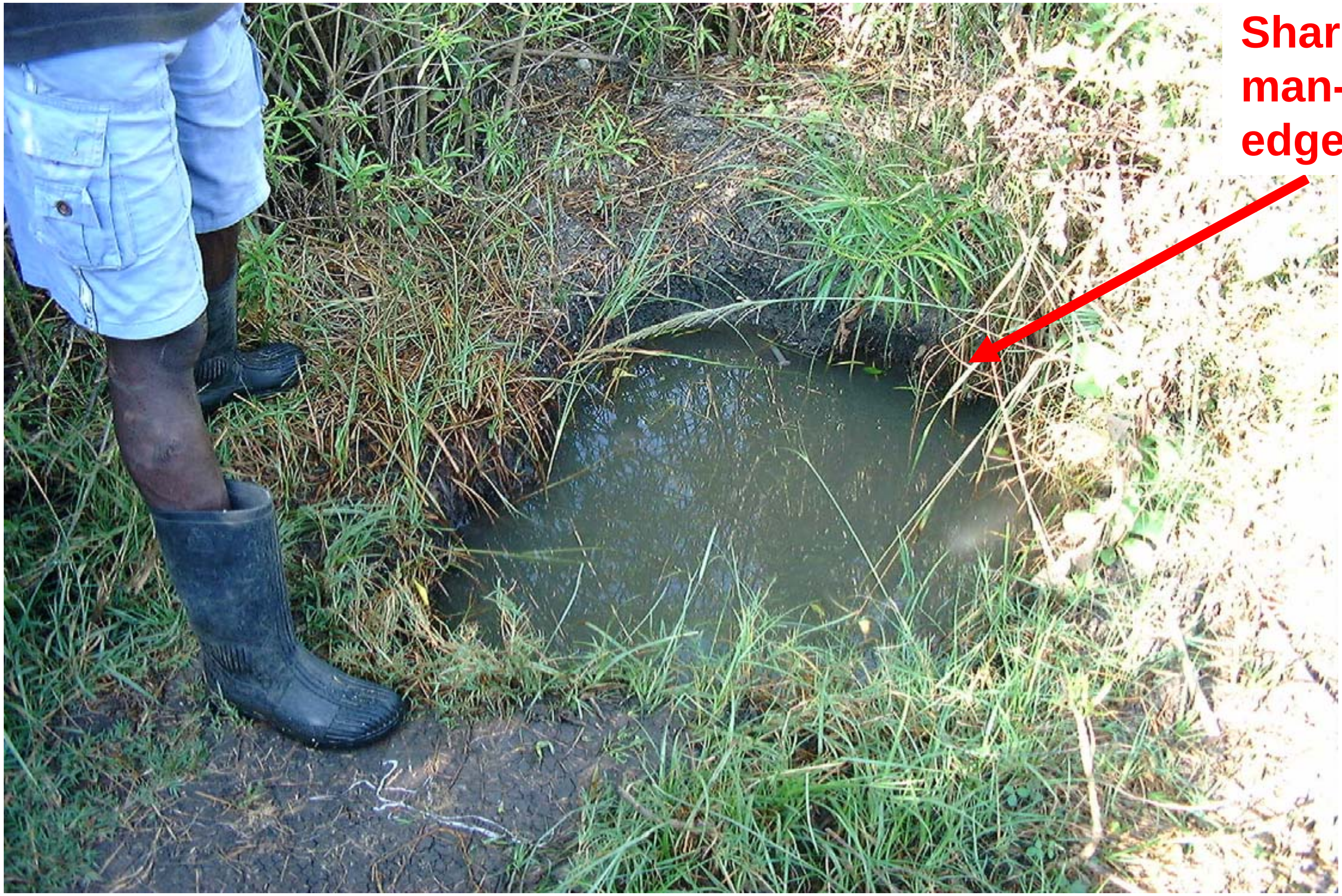

Sharp man-made edges

This document has been produced and made available by the Dar es Salaam Urban Malaria Control Programme. Contact: Urban Malaria Control Programme, City Medical Office of Health, City Council, P.O. Box 63320, Dar es Salaam, Tanzania, Phone: +255 22 212 1649

5: Construction pits, foundations and man-made holes

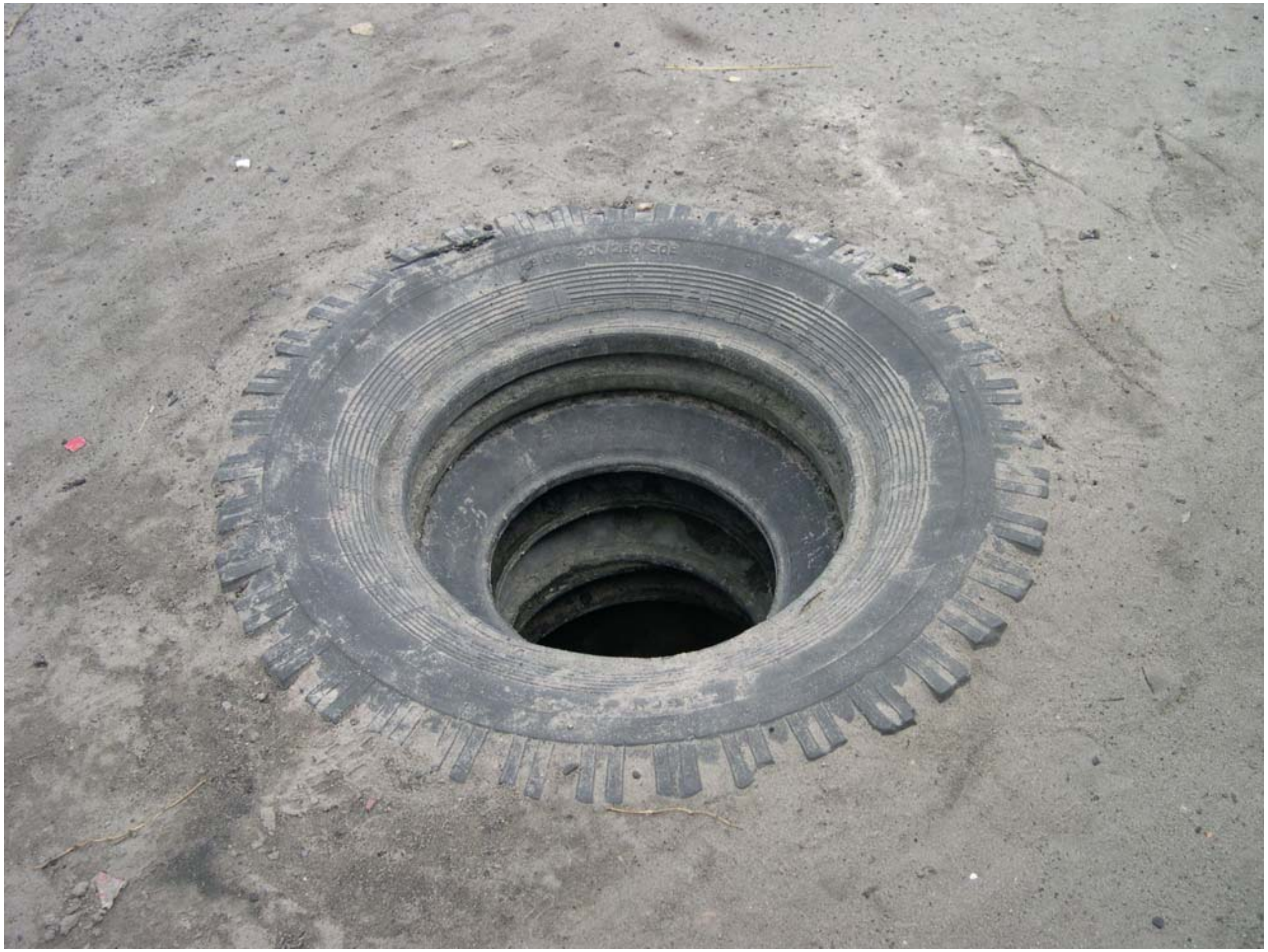

This document has been produced and made available by the Dar es Salaam Urban Malaria Control Programme. Contact: Urban Malaria Control Programme, City Medical Office of Health, City Council, P.O. Box 63320, Dar es Salaam, Tanzania, Phone: +255 22 212 1649

6: Water storage or other Man-made containers:

- **any** container that holds water that could serve mosquitoes to breed (which were left for **more than a week**)

- **open water** storage tanks, barrels, tyres, livestock feeding trays

- Do not record all small buckets, flower pots, watering cans etc, since the water will be used and their position changed

This document has been produced and made available by the Dar es Salaam Urban Malaria Control Programme. Contact: Urban Malaria Control Programme, City Medical Office of Health, City Council, P.O. Box 63320, Dar es Salaam, Tanzania, Phone: +255 22 212 1649

6: Water storage or other Man-made containers:

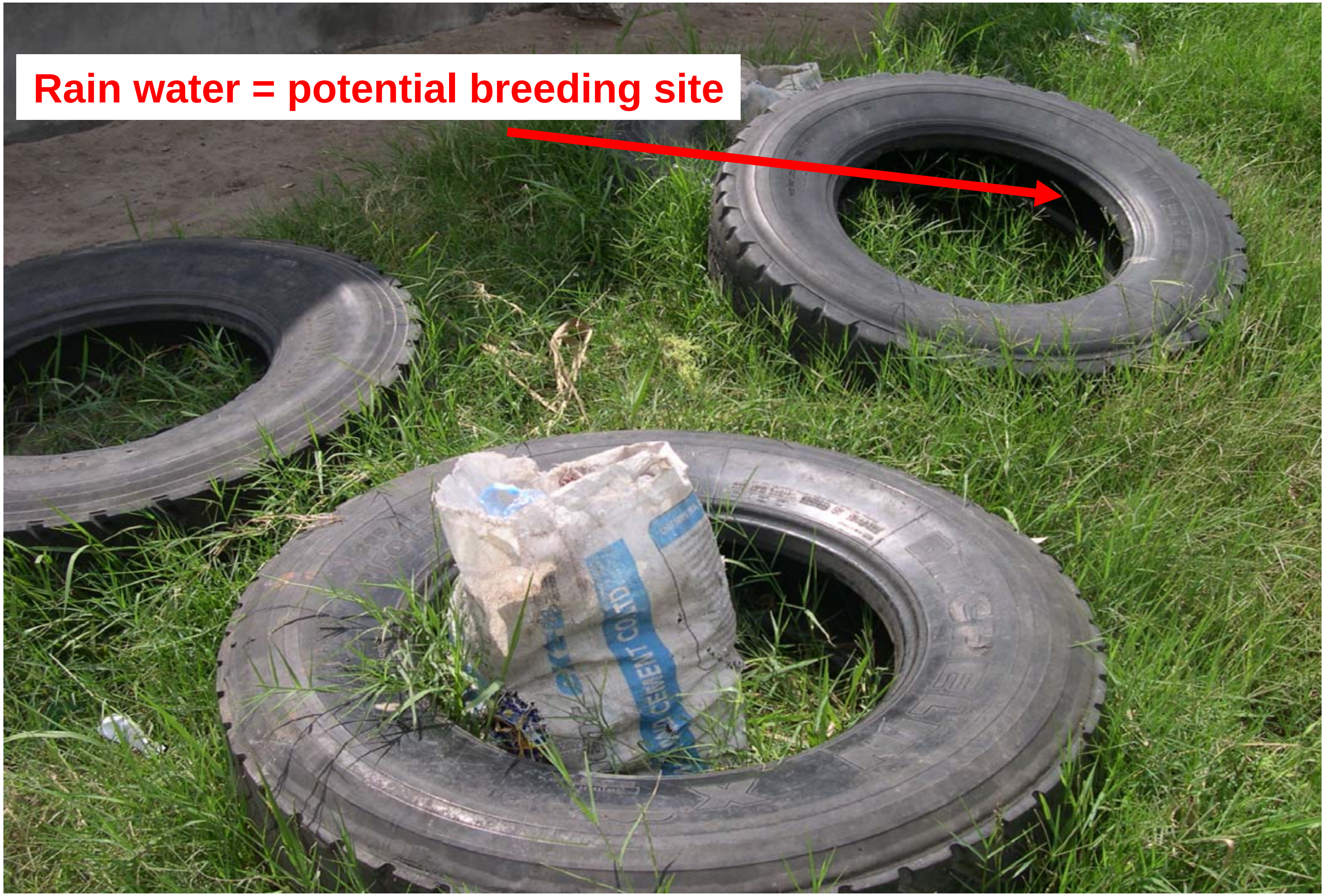

This document has been produced and made available by the Dar es Salaam Urban Malaria Control Programme. Contact: Urban Malaria Control Programme, City Medical Office of Health, City Council, P.O. Box 63320, Dar es Salaam, Tanzania, Phone: +255 22 212 1649

7: Rice paddy (Rice field)

- plots where rice grows
- drying up = small pools = concentrated mosquito larvae

This document has been produced and made available by the Dar es Salaam Urban Malaria Control Programme. Contact: Urban Malaria Control Programme, City Medical Office of Health, City Council, P.O. Box 63320, Dar es Salaam, Tanzania, Phone: +255 22 212 1649

7: Rice paddy (Rice field)

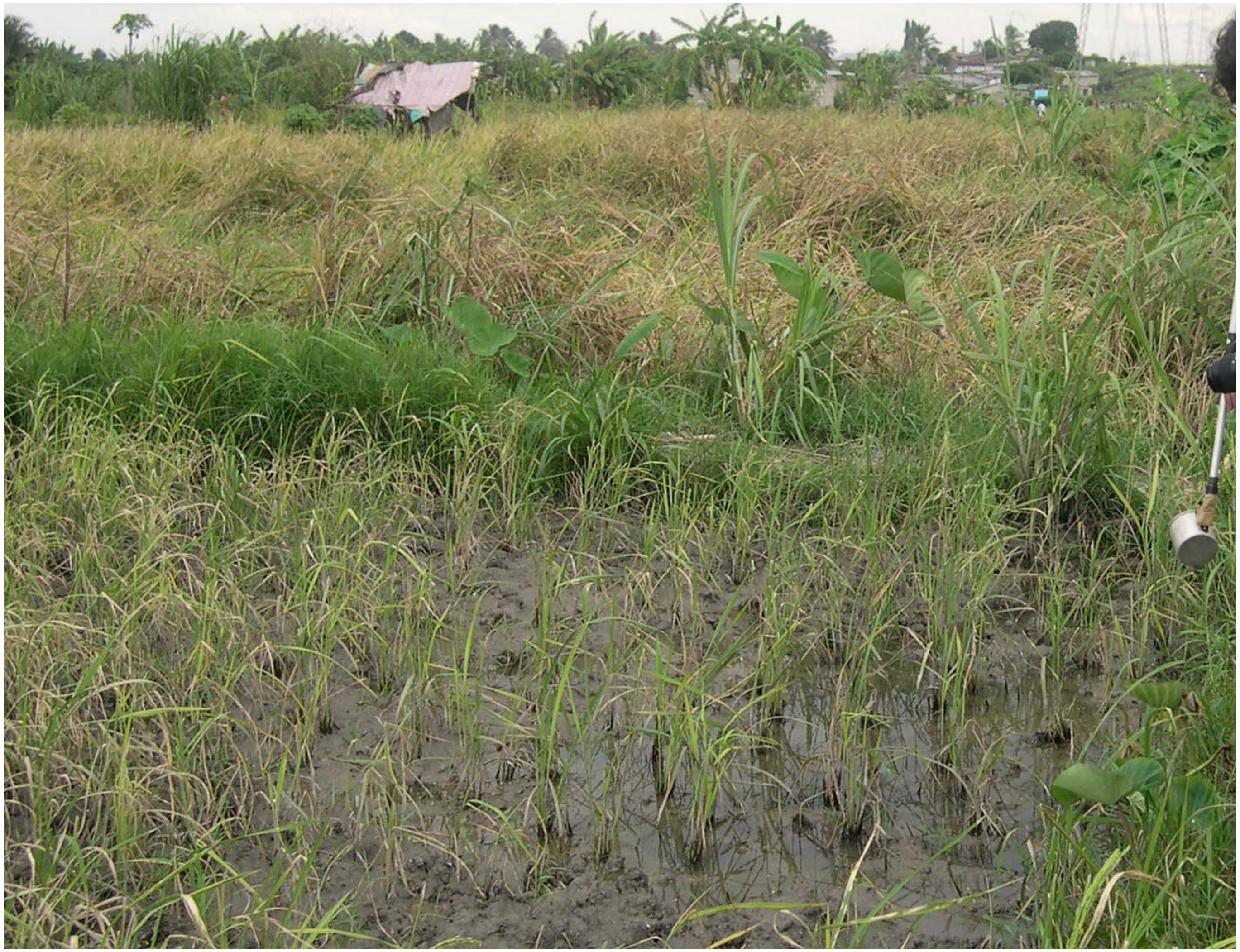

This document has been produced and made available by the Dar es Salaam Urban Malaria Control Programme. Contact: Urban Malaria Control Programme, City Medical Office of Health, City Council, P.O. Box 63320, Dar es Salaam, Tanzania, Phone: +255 22 212 1649

7: Rice paddy (Rice field)

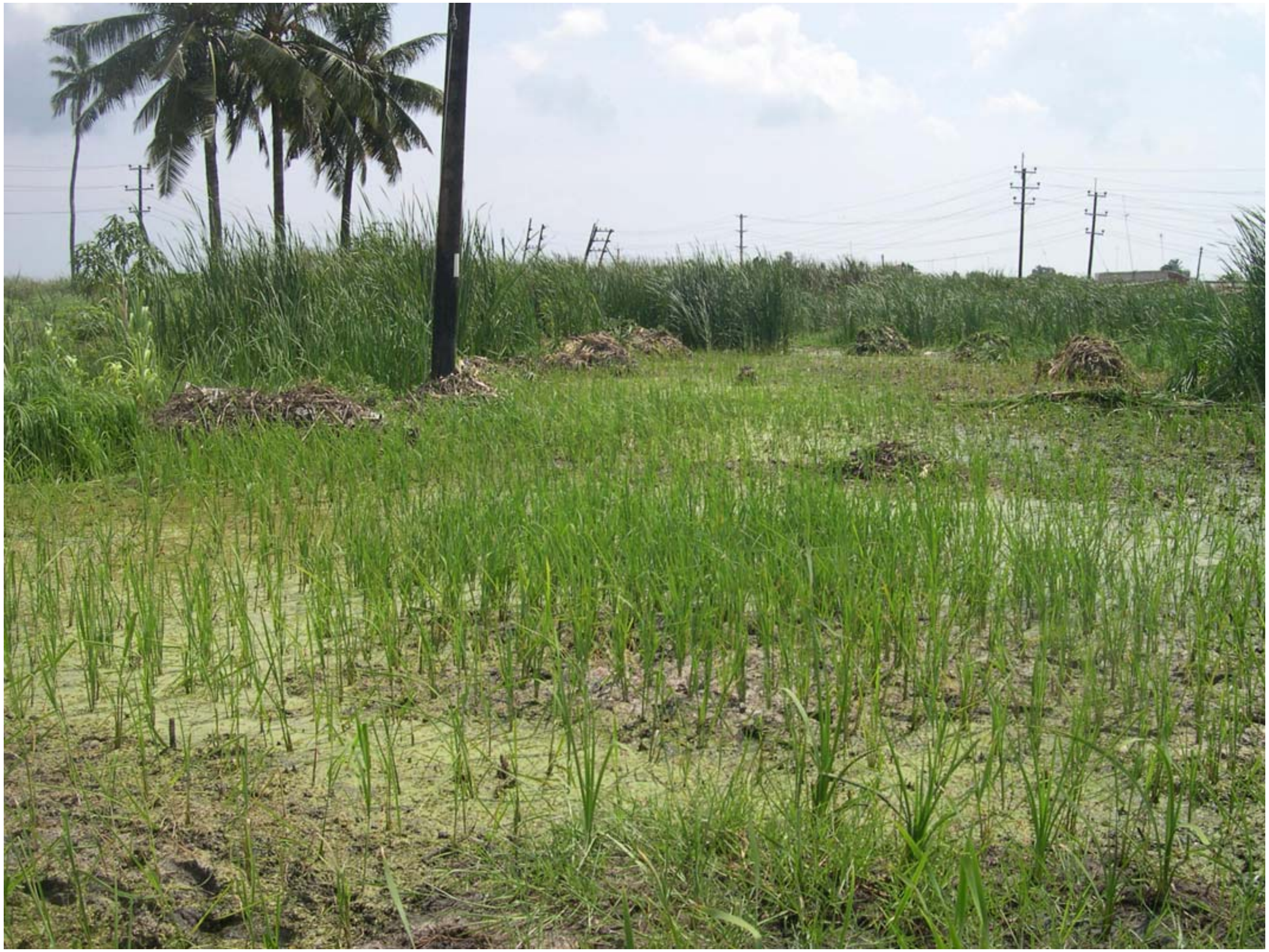

This document has been produced and made available by the Dar es Salaam Urban Malaria Control Programme. Contact: Urban Malaria Control Programme, City Medical Office of Health, City Council, P.O. Box 63320, Dar es Salaam, Tanzania, Phone: +255 22 212 1649

8: Matuta

- **raised ridges** on agricultural plots
- **man-made** furrows = hold water for longer duration
- larvae in very small depressions

This document has been produced and made available by the Dar es Salaam Urban Malaria Control Programme. Contact: Urban Malaria Control Programme, City Medical Office of Health, City Council, P.O. Box 63320, Dar es Salaam, Tanzania, Phone: +255 22 212 1649

8: Matuta

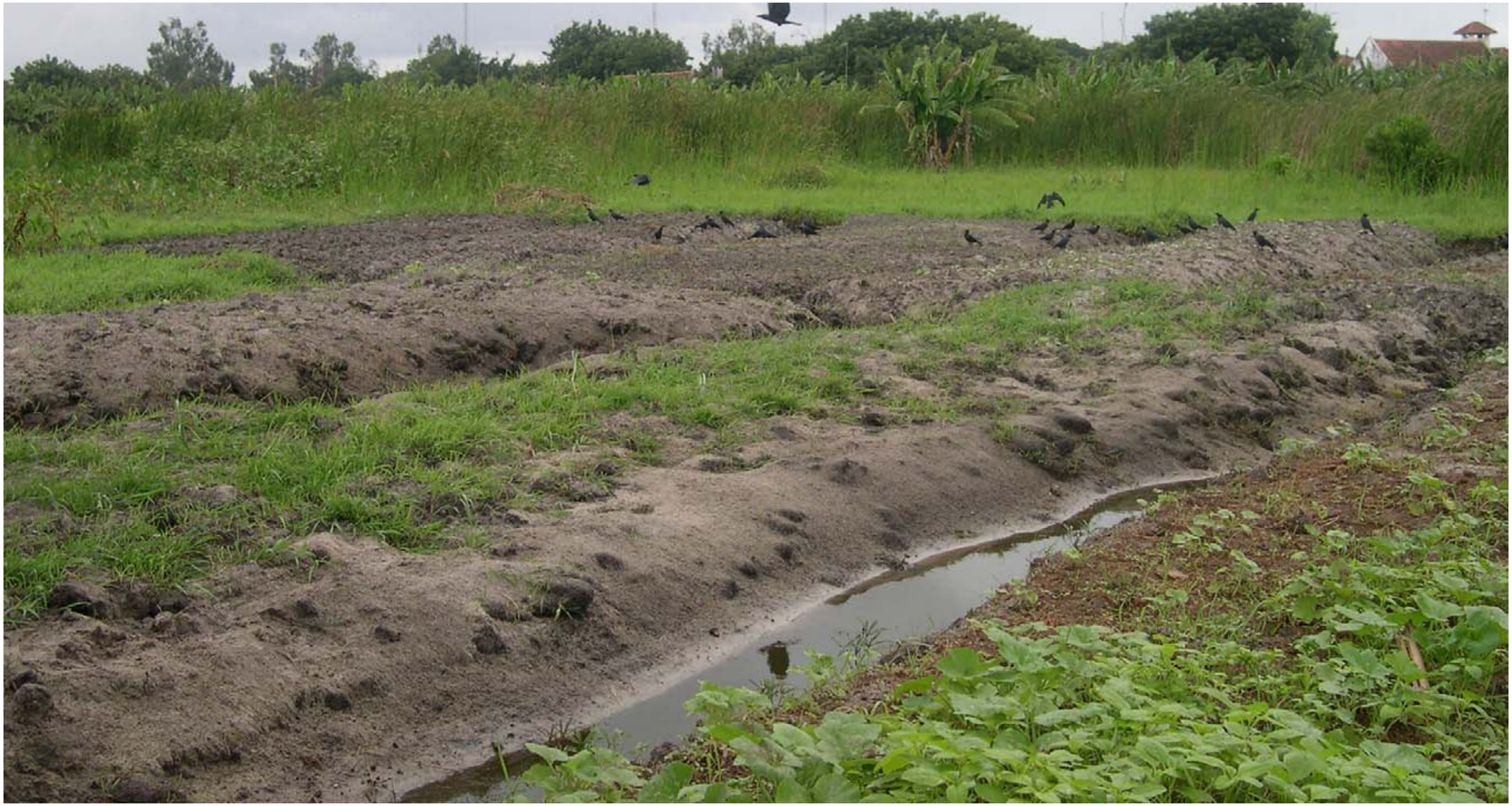

This document has been produced and made available by the Dar es Salaam Urban Malaria Control Programme. Contact: Urban Malaria Control Programme, City Medical Office of Health, City Council, P.O. Box 63320, Dar es Salaam, Tanzania, Phone: +255 22 212 1649

8: Matuta

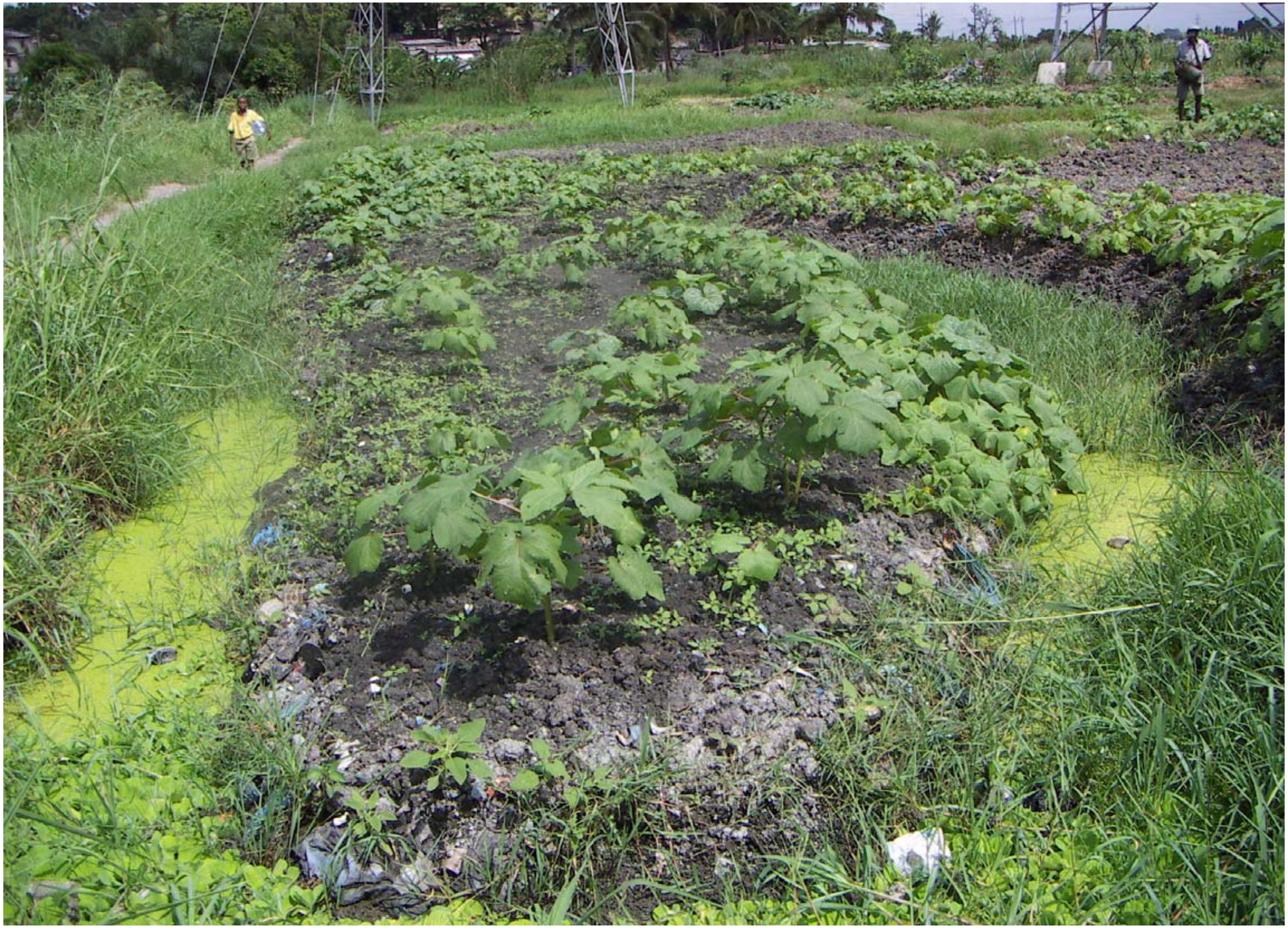

This document has been produced and made available by the Dar es Salaam Urban Malaria Control Programme. Contact: Urban Malaria Control Programme, City Medical Office of Health, City Council, P.O. Box 63320, Dar es Salaam, Tanzania, Phone: +255 22 212 1649

9: Other Agriculture

- **stagnant** water bodies
- water source = irrigation or rainfall or high water table

This document has been produced and made available by the Dar es Salaam Urban Malaria Control Programme. Contact: Urban Malaria Control Programme, City Medical Office of Health, City Council, P.O. Box 63320, Dar es Salaam, Tanzania, Phone: +255 22 212 1649

9: Other Agriculture

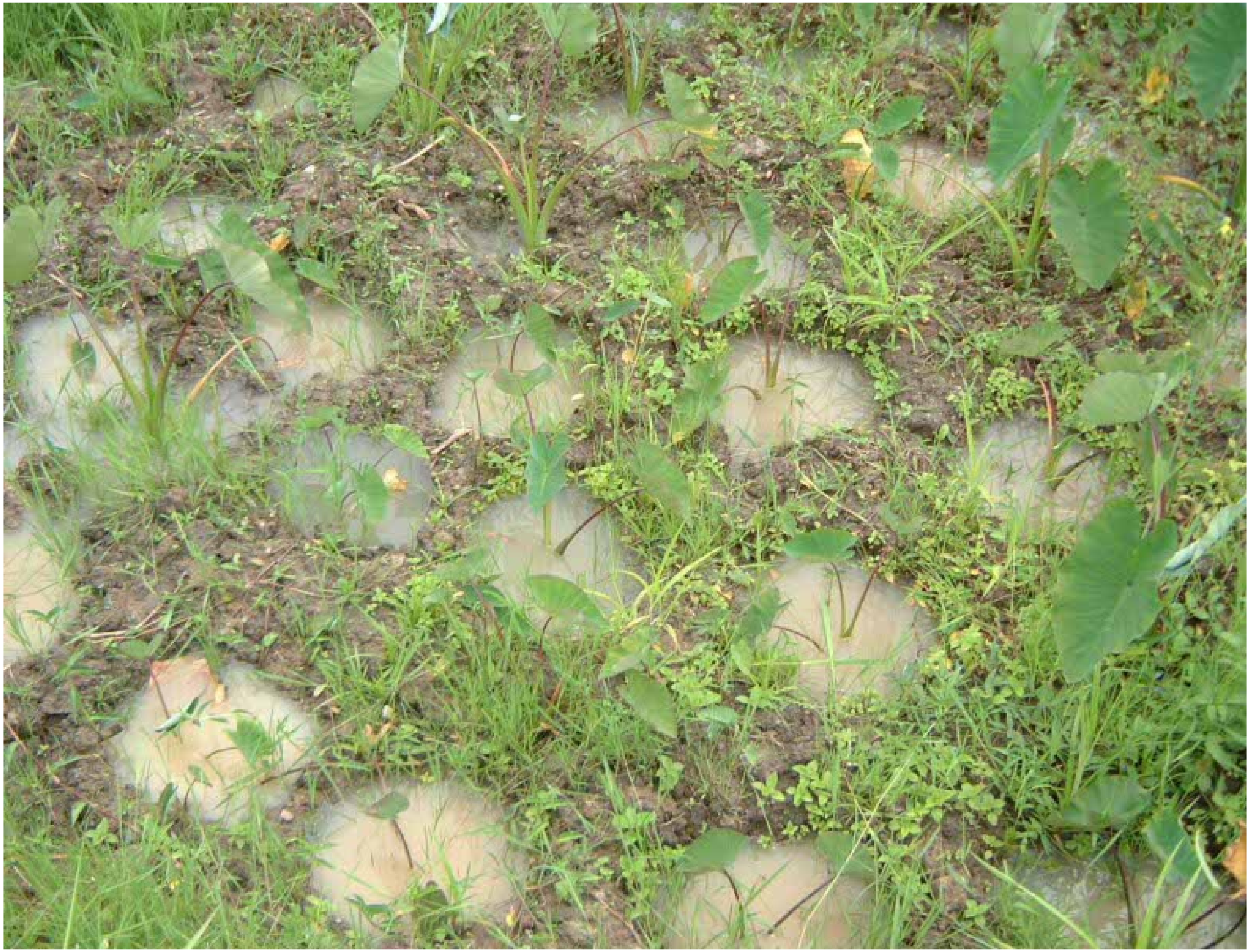

This document has been produced and made available by the Dar es Salaam Urban Malaria Control Programme. Contact: Urban Malaria Control Programme, City Medical Office of Health, City Council, P.O. Box 63320, Dar es Salaam, Tanzania, Phone: +255 22 212 1649

10: Stream and River beds

- **Fast or slow flowing** water, although it can be seasonal
- Natural, not man-made
- twisting course **not** straight as for ditches and drains
- mosquito larvae habitats usually at

  -edges very slow flow or stagnant

  -seasonal rivers and creeks dry up at certain times in year and leave stagnant pooling water

This document has been produced and made available by the Dar es Salaam Urban Malaria Control Programme. Contact: Urban Malaria Control Programme, City Medical Office of Health, City Council, P.O. Box 63320, Dar es Salaam, Tanzania, Phone: +255 22 212 1649

10: Stream and River beds

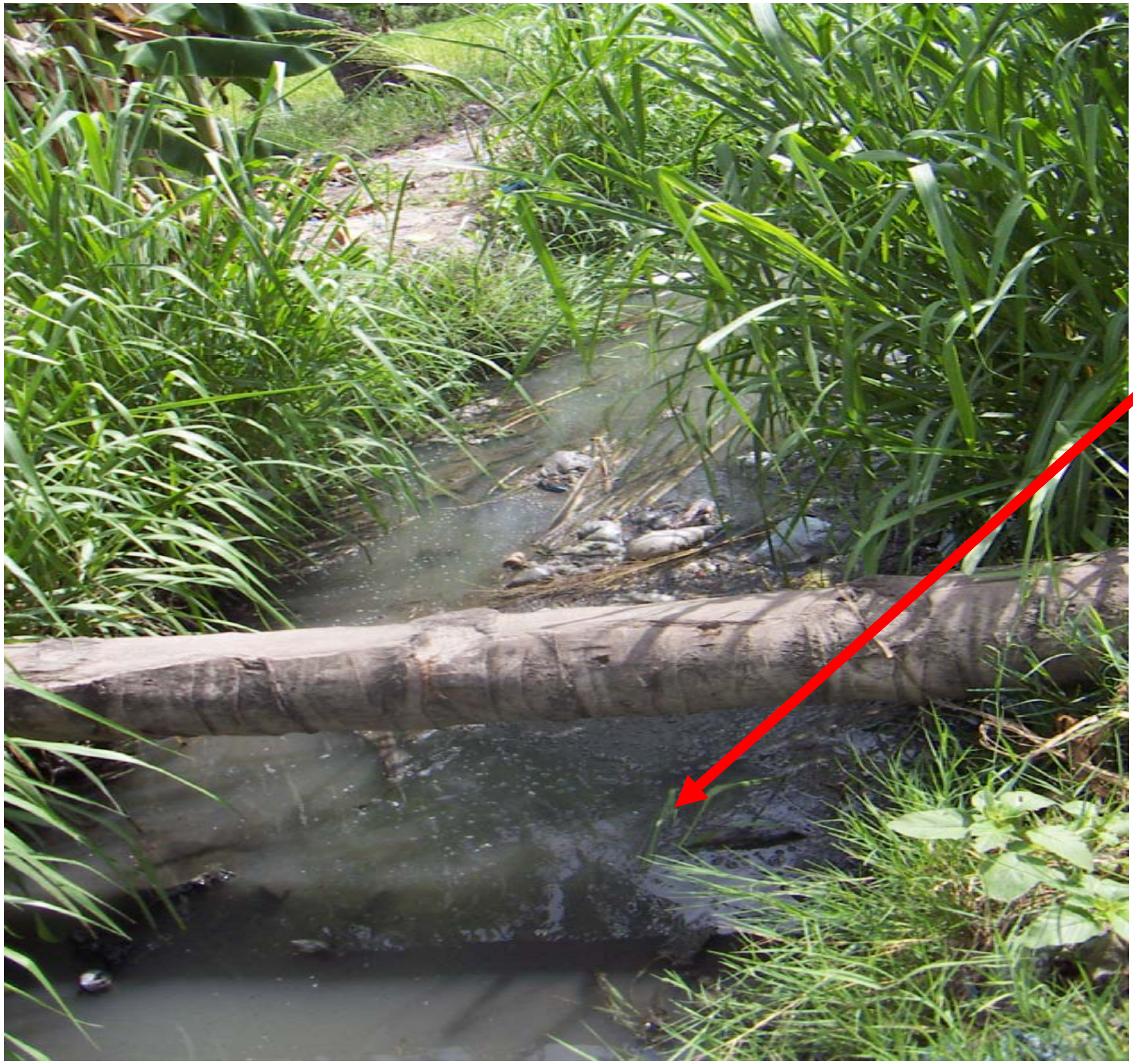

Flow = water current

This document has been produced and made available by the Dar es Salaam Urban Malaria Control Programme. Contact: Urban Malaria Control Programme, City Medical Office of Health, City Council, P.O. Box 63320, Dar es Salaam, Tanzania, Phone: +255 22 212 1649

10: Stream and River beds

10: River

2: Swampy Areas

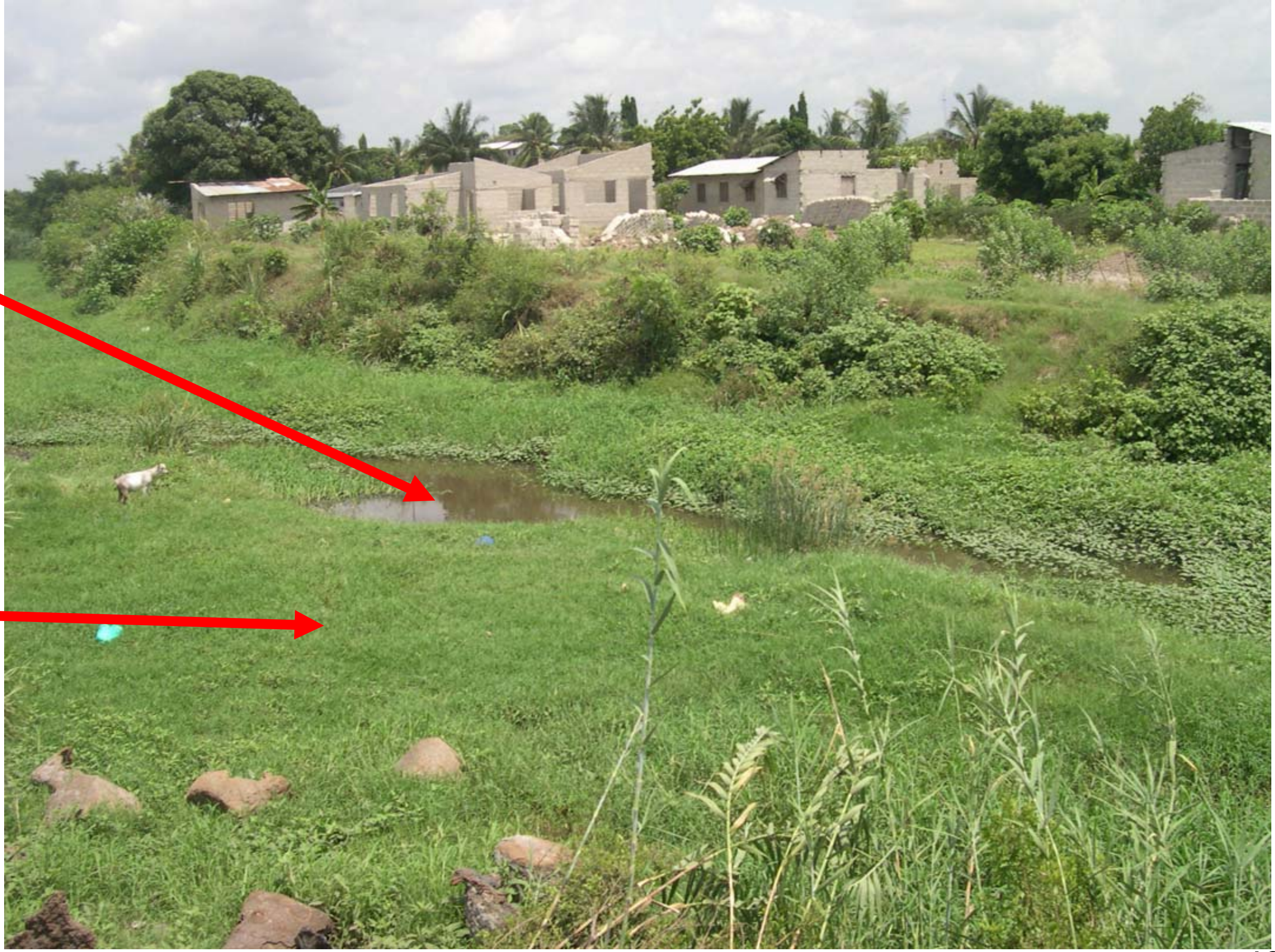

This document has been produced and made available by the Dar es Salaam Urban Malaria Control Programme. Contact: Urban Malaria Control Programme, City Medical Office of Health, City Council, P.O. Box 63320, Dar es Salaam, Tanzania, Phone: +255 22 212 1649

11: Ponds

- medium to large size **stagnant** water
- water present for several months in the year
- rainy season (depth can be >0.5 m, in the middle of habitat)

This document has been produced and made available by the Dar es Salaam Urban Malaria Control Programme. Contact: Urban Malaria Control Programme, City Medical Office of Health, City Council, P.O. Box 63320, Dar es Salaam, Tanzania, Phone: +255 22 212 1649

11: Ponds

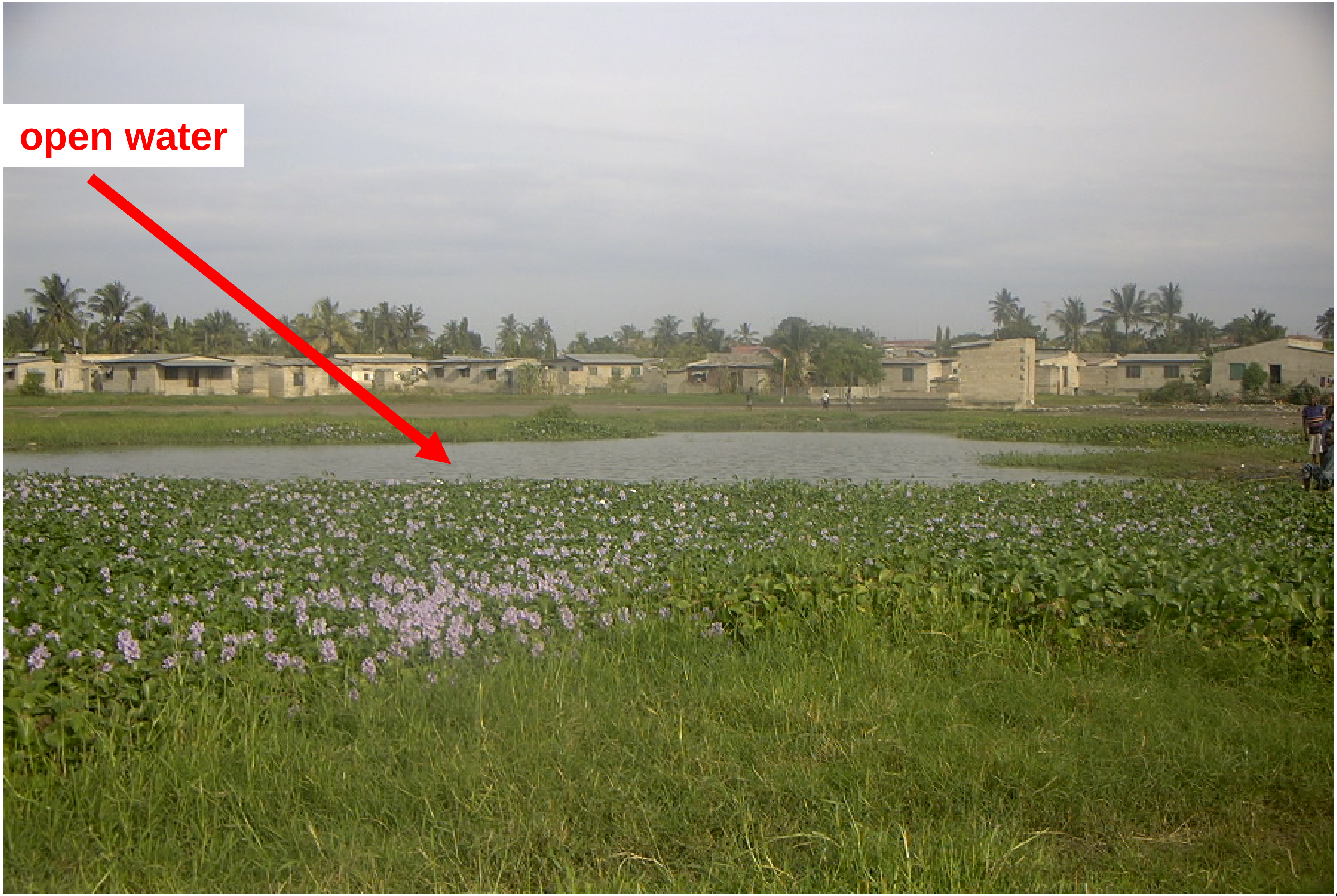

This document has been produced and made available by the Dar es Salaam Urban Malaria Control Programme. Contact: Urban Malaria Control Programme, City Medical Office of Health, City Council, P.O. Box 63320, Dar es Salaam, Tanzania, Phone: +255 22 212 1649

12: Others

- any other stagnant water bodies that could be mosquito larval habitats
- please make sure you have **checked** the definitions of habitat categories 1 to 11
- please **describe** the habitat recorded under category 12

This document has been produced and made available by the Dar es Salaam Urban Malaria Control Programme. Contact: Urban Malaria Control Programme, City Medical Office of Health, City Council, P.O. Box 63320, Dar es Salaam, Tanzania, Phone: +255 22 212 1649
