# Supplementary material for: A tool box for operational mosquito larval control: preliminary results and early lessons from the Urban Malaria Control Programme in Dar es Salaam, Tanzania
Source: Malar J. 2008 Jan 25;7:20. doi: 10.1186/1475-2875-7-20 (PMC2259364; doi:10.1186/1475-2875-7-20)
Supplement: Additional file 7 — Guidelines for larvicide application. The document presents information on microbial larvicides, calibration and the standard operating procedures developed for weekly larvicide application. [file 1475-2875-7-20-S7.pdf]

Additional file 7:

This document has been produced and made available by the Dar es Salaam Urban Malaria Control Programme. Contact: Urban Malaria Control Programme, City Medical Office of Health, City Council, P.O. Box 63320, Dar es Salaam, Tanzania, Phone: +255 22 212 1649

Larval Control Interventions in Dar es Salaam

GUIDELINES

Background Information and Operational Procedures
March 2006

First Intervention Phase March 2006 to March 2007: Intervention Ward Selection

Following preliminary data analyses and field visits 3 wards have been selected as intervention sites (1 from each municipality) for 2006 while the other 12 wards will remain untreated controls. Of these wards where no insecticides will be applied this year, 3 (1 from each municipality) have been selected to be compared with the intervention wards for final analyses. The selection of the sites was based on the following observations:

All study wards have shown to greatly differ during the baseline data collection period in their habitat numbers available, the proportion of available habitats colonised by *Anopheles* larvae, the density and seasonality of adults found in houses and the malaria prevalence. The research team based the decision of which wards will receive larviciding and which wards will be compared with the intervention wards mainly on the proven ability of the ward supervisors and ward-based CORPs to implement the required task. Specifically, their ability to collect, understand, use and submit high quality data during the baseline data collection period was the primary criterion for choosing these high priority wards.

Specific Objectives of the Mosquito Larval Control Pilot Studies in 2006

- To identify and characterise all potential aquatic habitats of culicine and vector anophelines in the study wards and to study their availability over time
- To study seasonal larval population dynamics of Culex and vector anophelines
- To establish the level of biting intensity by anopheline and culicine mosquitoes and determine human malaria exposure, measured as the entomological inoculation rate (EIR) during the dry and rainy seasons
- To determine the prevalence of malaria infections in the population
- To implement the microbial larval control intervention in 3 study communities (wards)
- To ensure community consent and cooperation

Study Hypothesis

Larval mosquito control in urban Dar es Salaam where malaria transmission is relatively low and focal will decrease densities of adult mosquitoes to such an extent that malaria transmission will also decline and reduce the level of malaria infection prevalence in local communities/wards where larviciding takes place.

Timeline

- Collection of baseline data from March 2005 to February 2006:
    - Availability of aquatic habitats (weekly)
    - Colonisation of habitats with Anopheles mosquitoes (weekly)
    - Adult mosquito densities in houses (weekly)
    - Malaria prevalence and incidence in population (twice per year in each ward)
- Training on application of microbial larvicides in February 2006

- Implementation of weekly monitoring and larviciding in intervention sites from March 2006 to March 2007
    - Monitoring and Evaluation of intervention from March 2006 to March 2007 will use the same surveillance system described above for the baseline period
    -

Pilot study design

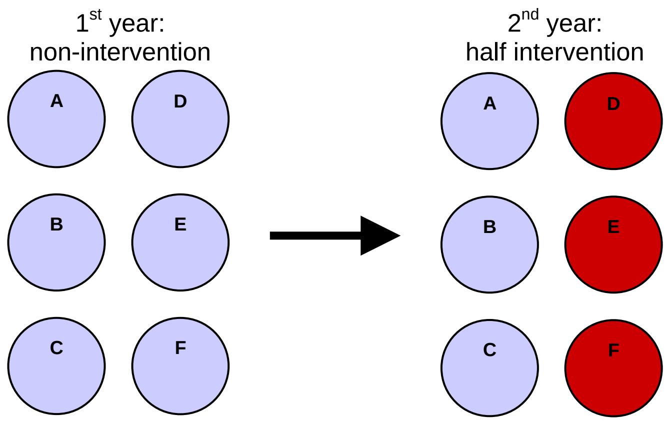

Site Selections

Non-Intervention Sites

- Keko
- Vingunguti
- Mwananyamala

Intervention Sites

- Kurasini
- Buguruni
- Mikocheni

Bacillus formulations – Background

- Discovery of the mosquitocidal Bacteria strains of *Bacillus thuringiensis* var. *israelensis* (*Bti*) and *Bacillus sphaericus* (*Bs*) during the mid-1970s
- Advantages of microbial larvicides:
    - highly effective (need very little to kill mosquito larvae)
    - selective in action (kill only mosquito and blackfly larvae in recommended dosage)
    - environmentally safe to non-target organisms (other organisms living in water like those that feed on mosquito larvae will not be killed)
    - Safe for human handling and consumption: Microbials are natural mosquito diseases that can in no way harm humans. In fact **WHO recommends it for drinking water**.
    - easy and safe to handle
- Resistance: *Bs* can introduce resistance but this can be reversed by rotating with an alternative insecticide. Resistance to *Bti* has **never** been observed in over 30 years of use around the world.

Bacillus formulations - Mode of action

- *Bacillus* is a bacteria that forms spores when conditions become adverse.
- During formation of spores a special protein is produced
- This protein is toxic to mosquito larva but only when eaten by them.
- The mosquito-killing protein is activated by digestive enzymes and alkaline pH in midgut of the mosquito larvae
- These special proteins then attack the midgut causing the formation of pores (small holes) and destruction of the cells that line the midgut
- Midgut pH drops to neutral
- Larvae can no longer digest food and die
- Only mosquito and blacklfy larvae provide conditions in gut to activate the mosquito-killing protein so the microbials do not affect any other living organism
- The toxins do not act on pupae because they do not feed anymore
- The younger the larvae the less toxin they need to digest to die, therefore they usually die quicker than late instars

Products
Commercially available products
Manufacturer: Valent BioSciences, Illinois, USA

We have to distinguish between two microbials and the two formulations of each microbial that might be used:
Microbials
- Bacillus thuringiensis var. israelensis (VectoBac®)
- Bacillus sphaericus (VectoLex®)

Formulations and application methods
- Water-dispersible Granule (WDG) applied as a liquid with knapsack sprayers
- Corn Granule (CG) applied by hand

Mode of application
water-dispersible granule (WDG) – diluted in water, applied as liquid with a knapsack sprayer
corn granule (CG) – applied as granular, undiluted finished product by hand

When to use what?

Liquid application with knapsack sprayer:
- Effective and easy to apply in sites that have little emergent or floating vegetation
- If there is large amount of emergent vegetation the spray may not penetrate the vegetation and get into the water

Granule application by hand:
- Slower to apply to large areas but broadly applicable, will reach the target in all circumstances
- Particularly effective in sites with emergent or floating vegetation that liquid applications cannot penetrate
- Granule penetrates vegetation and drops on water surface
- Granule can often be thrown a larger distance than liquid and can therefore be used to treat less accessible sites

Bti (VectoBac)
- In all habitats, less good in very polluted habitats (e.g. latrines)
- Needs to be applied weekly
- Cheap

Bs (VectoLex)
- In all habitats, also in very polluted water
- Can show an extended residual effect, application when late instar larvae occur, this needs weekly monitoring
- expensive

Application Dosages
Before the *Bti* and *Bs.* formulations can be used in the field, their actual potency and efficacy has to be evaluated against the different indigenous mosquito species. To assess the minimum effective dosage bioassays need to be carried out in the laboratory following World Health Organisation (WHO) guidelines. To assess the optimum effective dosages field trials either in natural or in artificial habitats need to be carried out. The outcome of these preliminary tests on larval control answer the following questions: What is the minimum and optimum effective dosage of the formulations against indigenous *Anopheles* and *Culex* mosquitoes? Is *Bti/Bs* suitable for the control of anopheline mosquitoes in the area? Which concentrations have to be used? In which intervals have re-treatments to take place? Which formulations are most powerful? Which are the best application methodologies?

Preparatory studies have been carried out at ICIPE, Mbita, western Kenya between 2002 and 2004. Following the results from these studies recommended formulations and dosages for open, potentially *Anopheles*-producing habitats are shown in the table below:

To achieve 100% control of mosquito larvae in any habitat in 24 hours, use:

VectoLex WDG (650 ITU/mg)	2.0 kg/ha	0.20 g/m2
VectoBac WDG (3000 ITU/mg)	0.4 kg/ha	0.04 g/m2
VectoLex CG (50 ITU/mg)	30 kg/ha	3 g/m2
VectoBac CG (200 ITU/mg)	10 kg/ha	1 g/m2

ITU = International Toxic Units, describes the potency of larvicide, the higher the number, the more toxic is 1mg the less is needed to kill 100% of larvae within 24hrs.
Always note Lot number & ITU of product used in the field, ITU and Lot number are indicated on the product.

Any Bti product (VectoBac) NEEDS to be applied in WEEKLY intervals. Bti products do not have any longer residual effect.

Selection of Larvicide for Dar es Salaam in 2006
We will take two approaches to two different categories of habitats. Open habitats which are exposed to sunlight and hence potential sources of *Anopheles* will be treated directly by the program *Mosquito Control CORPs* with Bti (VectoBac) only. For closed habitats in domestic settings which are not exposed to sunlight and produce no *Anopheles* but lots of nuisance culicine mosquitoes, small amounts of Bs (VectoLex) will be provided to households by programme staff.

Since we deal with highly polluted habitats in the urban area we will double the optimum dosage as identified above for routine use in Dar es Salaam.

For open habitats we will apply:
VectoBac (Bti) CG at 1 gram per square meter (10 kg per hectare)
OR
VectiBac (Bti) WDG at 0.04 gram per square meter (0.4 kg per hectare)

For our first year larviciding we have decided to use only Bti (VectoBac) for open larval habitats. Bti will be applied as corn granule (CG) formulations for hand application and water dispersible granule (WDG) for application as a liquid with knapsack sprayers where this formulation is appropriate. We will use Bti only for open habitats and this product must be applied weekly because it has no residual activity but is the cheapest option and does not require any additional monitoring and decisions on re-application dates.

Application Equipment and Procedures

Liquid application: Solo 475 knapsack sprayers with a capacity of 14 L will be used to apply water dispersible granular (WDG) formulations. They are an effective method of application in sites that have little emergent vegetation. If there is a large amount of emergent vegetation the spray may not penetrate and get into the water. The selected knapsack sprayers are relatively light and simple to use. They use compressed air above the spray mixture to push the mixture out of the tank through a hose and nozzle. The output of the sprayer is dependent on the pressure used, the nozzle type and the speed of walking during the application. Calibration of the knapsack sprayers can be practiced easily following standard operating procedures. The WDG formulations are easy to use since they dissolve in water easily. Therefore, it can be directly mixed in the knapsack sprayer by adding the larvicide and filling the sprayer to its maximum mark. The sprayer needs to be shaken well before pressure is added to the spray mix. To fill a full tank of the Solo 475 sprayer, 400 grams of WDG powder can be dispersed in water by mixing with agitation in approximately half a tank of water (7L), adding the remainder of the water to achieve a total

volume of 14L, and then mixing vigorously for 2 minutes. To prepare a half a tank, mix 200g of powder with 3 to 4 liters of water and make up to 7 liters or the halfway mark in a similar fashion. Only when the powder is fully dispersed into liquid form can pressure be applied and application begin: An application pressure of approximately 3 bar is achieved and maintained by pumping a Solo 475 sprayer with a number 2 disk and no core to pressure setting number 3. Calibration in Dar es Salaam indicates a typical mosquito control CORP achieves a swath width of 10 m, a flow rate of 0.74 litres per minute, and a walking rate of 54 m. With this dilution, flow rate, walking speed and swath width, a full tank is expected to cover one full hectare but no more. This is equivalent to 10 x 100 meter swaths across a perfectly square area of one hectare (100m x 100m) or 1000 meters of continuous swath. The spray wand should be moved quickly and continuously across a 180° arc using a full swing while walking the length of the swaths.

Calibrated application specifications for liquid application:

- Dilution: 400 g of WDG for a full tank (14L) or 200g for a half tank (7L)
- Backpack configuration: **Number 2 disk with no core**.
- Pressure: Backpack setting number 3 (approximately 3 bar)
- Walking speed: Approximately 50 meters per minute
- Swath Width: 10 meters
- Expected usage rate: 1 full tank should treat one hectare or 1000 meters of swath length (eg 10 swaths across a perfectly square 1 hectare area: 100m x 100m). This means that each litre should last for approximately 70 meters of swath length.

Hand application: Granular formulations (CG) may be applied by hand, similar to scattering seeds. However, it takes practice to obtain an even application or maintain the recommended application rate. It is very important for the field staff to practice this exercise well to gain experience in achieving even coverage as per the recent calibration workshop. For hand application from granular formulation buckets are used on a carrying strap to be hung around the shoulders allowing it to rest on the belly. The carrying strap can be adjusted for individual comfort and effectiveness. As determined during the recent calibration workshop, our objective is to achieve a coverage rate of 1 gram of VectoBac CG per square meter (m^2), equivalent to 10 Kg per hectare. For medium to large areas (>$9m^2$ or 3m x 3m) with multiple habitats, this is best achieved by treating 3m-wide swaths with one handful spread over 10m of swath length. For smaller, distinct habitats, the area of the habitat should be measured and appropriate fractions of a handful (One handful = 25g) or a teaspoon (one teaspoon=2g) should be applied. For example, for a small habitat of approximately one meter squared, half a teaspoonful should be spread evenly by hand throughout the habitat. For a larger habitat of, for example 12 m^2 (3m ×4m), half a handful should be spread evenly across the habitat. For long, narrow (<1m) habitats such as remnants of foundation trenches running alongside walls, simply scatter granules in the target area as you walk the length of the habitat, aiming to cover 20-30m of habitat per handful of granules. For all these habitat types you can practice on surfaces where granules area readily seen, aiming to achieve even coverage with approximately 4 granules per 10 cm x 10 cm area. We summarize these application specifications for easy reference as follows:

Calibrated application specifications for liquid application:

- Coverage: Approximately 4 granules per 100 cm^2 or 10 cm x 10 cm area.
- Application rate for small to medium habitats: 1 teaspoon full per 2 m^2
- Swath width for habitats > 9 m^2 in size: 3 meters
- Application rate for swaths across habitats > 9 m^2 in size: 1 handful per 10 meters of swath length walked
- Application rate for long narrow habitats: 1 handful per 20 to 30 m of habitat length

Evaluation of Larval Control Success

In our study we hypothesize that in comparison to the non-intervention year and the non-intervention sites controlling the larval stages of mosquitoes in the 3 intervention wards will result in:

- Smaller proportion of habitats colonised by early instar mosquito stages.

- Late-instar larvae and especially pupae should be rare and extremely difficult to find.
- Much fewer (80% less than otherwise) adult *Anopheles* biting humans.
- Reduced malaria infection and illness in children.

Success depends on:

- Identification of **all** available aquatic habitats within the study area
- Treatment of **all** aquatic habitats in required dosages (e.g. treatment of drains for the full lengths) Proper performance of the larvicides
- Treatment at regular weekly intervals so that **no** late instar larvae are recorded in the sites
- **No** pupation and emergence takes place in any sites.

IMPLEMENTATION PROCEDURES & DATA RECORDING

Community sensitization

It is mandatory to inform and gain consent from the administration, community leaders and the community members before any larviciding can take place in the intervention areas. Community members are usually very concerned about any pesticide applied by research teams. There is usually the fear that pesticides applied on water could affect human beings or live stock.
District administration officials (and others) need to be visited and informed about the planned activities, their appearance at community sensitization meetings might be helpful. Community leaders need to be informed and with their help community meetings need to be held. Any questions and concerns of the community need to be answered to the best of your knowledge. Questions that can not be answered immediately need to be discussed with the scientists and information brought back to the community. **Families that farm in the intervention areas should be especially addressed to ensure that the information reaches them well, since those will be much concerned with the weekly larviciding and might fear for their crops or animals.** A community information leaflet and a frequently asked questions fact sheet will be distributed during the sensitization meetings.

Community sensitization will be done using various methods, these are:

1. Meetings with well known community members/leaders including Ten Cell leaders.
2. Public addressing using megaphone by passing with a car through all the mitaa just before the intervention
3. Public meeting with the community by using traditional ngomas
4. Distribution of leaflet and frequently asked questions at all meetings.
5. Availability of larvicides for Household Control of closed habitats (packaging of VectoLex CG)
   Leaflet and announcements to households from intervention areas to ward office/meeting point to pick up larvicides for mosquito control in pit latrines and other closed habitats

Field Staff – *Mosquito Control CORPs* and *Larval Mosquito Surveillance CORPs*

During the intervention year the weekly larval surveys will be implemented by the *Larval Mosquito Surveillance* CORPs following the same standard procedures as during the baseline data collection. Additionally, in the intervention wards a team of *Mosquito Control CORPs* has been recruited so that surveillance and control of **all** the habitats in the targeted wards are conducted separately. Larval surveillance and application of larvicides will be implemented independently (these two teams of CORPS **do not** cover the area together! Instead, the surveillance team follows, using the same lists of ten cell units two days later).

Mosquito Control CORPs for the 3 intervention wards for 2006 were recruited in January 2006 and have followed the Mosquito Larval Surveillance CORPs for a one month to familiarise themselves with the area of operations. Larviciding will start 1st March 2006. A special timetable has been developed for larval survey CORPs and spraymen specifying days of the week and TCUs to be

visited at these days. Spraymen will visit the TCUs first and apply larvicides to all aquatic sites. The CORP will survey the same TCUs one day later for larvae.

Larval Survey Data Recording – Mosquito larval surveillance CORPs
Larval habitat and density data will be recorded weekly in intervention and non-intervention wards following the same procedures and data sheets as for the baseline data collection. All available aquatic habitats will be recorded and larval presence noted. In the intervention wards the larval survey CORP monitors the activity of the sprayman in his/her respective area of responsibility. If the CORP identifies sites with late instar larvae, he needs to highlight them in the data sheet and report this observation back to the supervisor the same day when he/she brings the data sheets back to the ward office. All larval survey CORPs need to return their data sheets to the ward office after finishing the day's work and inform the supervisor verbally at the same day about any TCUs and sites where old larvae have been found and where larvicide application still needs to be done. The supervisor needs to discuss this with the spayman responsible for the area.

Larviciding Data Recording – Mosquito Control COPRs

In his area of responsibility (*mtaa* or part of an *mtaa*) the Mosquito Control CORPs will have to treat **ALL** available sites that contain water at the moment of the visit. This **must** happen weekly and irrespective of the presence or absence of larvae. Therefore, the Mosquito Control CORPs will not carry a dipper and will not record every single habitat that has been treated. The Mosquito Control CORPs searches every TCU that he or she is supposed to visit on this date (following the timetable prepared by supervisors and CMSOs) for any site that contains water (open habitats) using also the experiences gained from following the larval survey CORP during the first 4 weeks of training. **BUT** it is important that the Mosquito Control CORP does not only visit the sites he has learned to have water during his training but finds and treats **all** potential sites.

Note: The Mosquito Control CORPs are trained during the dry season! He will experience several times more habitats during the rains. Supervisors and Mosquito Control CORPs need to be trained to this effect and CMSOs need to remind them regularly.

The Mosquito Control CORP has to record the following information:
Week and date of application, TCU visited for larviciding, the total number of TCUs visited, the amount of larvicide received per day (as weight and indicated in data sheet by supervisor), amount of larvicide left after day's work (as weight and indicated in data sheet by supervisor) and the calculated amount of larvicide used per day (calculated and recorded in data sheet by supervisor).

A mosquito control CORP will have 1 data sheet for every day in the week (Mon, Tue, Wed, Thu, Fri), see example below:

Ward level larviciding - Open habitats

MUNICIPALITY:___Temeke____ WARD:_____Kurasini___ MTAA:_____Kurasini_______
Round (filled at City level):_____________________

Day	Date	TCU number	Wet habitats present?		Larvicide applied?		Comments
			YES	NO	YES	NO	
MON	01.01.06	001	x		x		
		002	x		x		
		005		x		x	
		007		x		x	
		008		x		x	
		009	x			x	no application because access was denied by residents

Total number of TCUs where larvicide application took place today: ___________
Amount of Larvicide received today (in kg): ______________________________
Amount of Larvicide left (in kg): _____________________________________
Amount of Larvicide used today (in kg): ________________________________

Mosquito Control CORP signature: ____________________ Supervisor's Signature: _________________

Records on Larvicide use and areas treated – Ward Supervisor

The ward supervisors (and the assistant supervisor in Mikocheni) need to keep daily records of the material released and returned per day and need to prepare a weekly summary of used material per Mosquito Control CORP:

- The larvicide will be stored at the ward offices in the intervention wards.
- The ward supervisors will hand out larvicides to the Mosquito Control CORP every morning between 7.00 and 8.00am.
- The released material has to be recorded per Mosquito Control CORP. Both, supervisor and Mosquito Control CORPs have to sign.
- A separate material recording sheet will be used for each Mosquito Control CORP and therefore for each area/Mtaa.
- The supervisor weighs the material and indicates the amount released in the his own larvicide release data sheet and in the ward level larviciding data sheet of the Mosquito Control CORP
- The Mosquito Control CORP returns in the afternoon after finishing the day's work to the ward office
- The supervisor weighs the remaining amount of larvicides and indicates this in his own and in the Larval Control CORP's data sheet and calculates the amount of larvicide used
- The larviciding data sheet of this day remains then in the ward office
- These datasheets need to be checked immediately when they are submitted and if there is no problem identified need to be filed in a separate file for larvicide application (1 file per Mtaa or subzone of Mtaa=1 Mosquito Control CORP)
- In case any problem can be identified from the data sheet the ward supervisor **must** discuss with the larval control COPRs and investigate further. The supervisor needs to discuss the problem with the inspector, plan and implement appropriate action promptly. In case problems arise that can not be addressed by the ward supervisor he/she should consult the inspector and, if necessary municipal coordinator immediately. If the problem still cannot be resolved promptly, help should be sought from the City Office **immediately**.

Larvicide Release Records

MUNICIPALITY: WARD: Mtaa: Sprayman's name:

Supervisor's name:

Week:	Day:	Date:	Amount of Granule received (in kg):	Signature Sprayman	Amount of granule returned (in kg)	Amount of granule used	Total number of TCUs treated (as per Mosquito Control CORP data sheet)	Signature Supervisor
1	Mon							
1	Tue							
1	Wed							
1	Thu							
1	Fri							
**1**	**Weekly Total:**							
2	Mon							
2	Tue							

Record on the daily release of larvicides will be taken on 1 data sheet per Mosquito Control CORPs per months. In this data sheet the supervisor also indicates how many TCU's have been treated according to the Mosquito Control CORP every day. At the end of the week the supervisor calculates the weekly total. This data sheet can then be sent back to the City with the weekly summary records from the larval surveys.

Culex Control in Closed Habitats

Closed habitats can not be managed by the spaymen of the program and they will focus on the open habitats only. Given that a large number of closed habitats (latrines, soakage pits, water tanks etc) produce a substantial number of nuisance Culex mosquitoes the community of the intervention wards might be disappointed because they might not feel a big reduction in nuisance biting. To increase community support we will offer larvicides for treatment of closed habitats for households free of charge. Small bags of granule will be made available at the mtaa office at

certain dates for households in the intervention wards. Members of those households can come to pick up the larvicide and a leaflet with directions for use and can treat the closed habitats themselves. We will use *Bacillus sphaericus* (Bs) granules (CG) for treatment of closed habitats. Bs is very effective in highly polluted water and has a long residual effect in closed habitats. Treatment of closed habitats has to take place every 2-3 months. One small bag of larvicide will contain 10 grams of granule which is sufficient to treat up to 10m^2 of water surface.

Organisation: Closed habitat treatment campaigns will be implemented every 3 months in all the intervention wards. The distribution of larvicides for householders will take place on Mtaa level at specific dates. Community sensitisation will take place a few days before the distribution to inform the community on which date and where they can come to collect larvicides for their closed habitats on household level. The Mtaa chairmen will be involved in the release of larvicides to ensure provision only to eligible households members. The householders name, address, type of closed habitats and number of larvicide bags will be recorded per Mtaa.

Storage and Distribution of Larvicides

The larvicides will be shipped to the City Office and will be stored at a central store (Kisutu Office). The keys for the store will be handled by City Council staff ONLY. Once a week, the necessary amount of larvicides will be delivered to the ward offices under supervision of the CMSOs. Records will be kept at the central store and at ward level, (account book for in and out need to be available). Ward supervisors have to sign for the weekly amount of larvicides they receive. The weekly supply will be delivered on Fridays. All ward offices will keep their larvicide stock in a dry and secure place that will be locked and can only be accessed by the ward supervisor. All four sites have been provided with locked cabinets for secure storage of larvicides.

Supervision and Support System for Intervention wards

Inspectors:

To support the intervention wards in the first year of larviciding one of the municipal inspectors has been assigned to the priority intervention ward and the non-intervention ward assigned for comparison in each municipality. The inspector will help the ward supervisors with all his/her duties, assists in problem solving, communication with City Office and will implement independent spot check to ensure good quality mosquito control in the intervention wards and data quality in non-intervention wards. Twelve randomly selected spot checks need to be implemented per week: 6 in the high priority (intervention plus comparison ward) and 6 in the lower priority (remaining three) wards; the visit of TCUs in the intervention ward need to be implemented 24-48 hrs after scheduled larvicide application by the sprayman (therefore the inspector has to check timetable of spraymen and plan day of spot check).

Municipal mosquito larval habitat spot check - Open habitats

Serial number of this form ____________________

Serial number on the map form ____________________

Ten cell unit identifier ____________________

Municipality:____________________ Ward:______________________ MTAA:______________________ 10-cell unit:____________________ 10-cell leader:______________________

GPS(UTM/WGS84): Northing______________________ Easting____________________________ Date of CORP's data sheet:_______/_______/_________

Serial number on the map form__________________________

	Yes	No
Is there a map?		
Is the map accurate?		
Does map match city copy?		

Habitat codes:

1: Puddles/tire tracks
2: Swampy areas
3: Mangrove Swamp
4: Drain/Ditch
5: Construction pits/foundations/man-made holes
6: Water storage & any other man-made container
7: Rice paddy
8: Matuta
9: Other agriculture
10: Stream/river bed
11: Pond
12: Others (describe below)

Plot ID	House No.	Habitat ID	Habitat type	Is habitat type correct?	Correct habitat type	Habitat found by the CORPs? 1=Yes 2=No	Habitat Description	Wet?		Habitat perimeter			Plants				Water depth		Larval stage						Pupae		Last application of larvicide as per timetable?	Comments
																			Anoph.			Culex						
								dry	contains water	< 10 m	10-100 m	> 100 m	None	Short vegetation	Tall vegetation	Floating plants	< 0.5 m	> 0.5 m	Absent	Early	Late	Absent	Early	Late	Absent	Present		

Additional targeted spot checks in areas of known larvae production or identified problem areas should be implemented by the inspector and ward supervisor throughout the week.

The second municipal inspector will be responsible for the remaining 3 lower priority wards in the municipality and will implement his/her routine duties. The routine TCU spot check data sheets remain the same as during the baseline data collection period except for 1 additional column where the latest larvicide application date (as per timetable of Mosquito Control CORPs) needs to be indicated in the intervention wards.

The results of the additional targeted spot checks in the intervention ward, identified problems and the action taken need to be included in the inspector's reports.

City Malaria Surveillance Officers:
CMSOs also need to implement independent spot checks in the 3 intervention wards weekly. Special attention needs to be given to areas where larval habitats are abundant. Spot checks should preferably take place 24-48 hrs after scheduled application. CMSOs should record the TCUs and habitats (Plot & Habitat ID) visited and the presence or absence of larval stages & pupae. The CMSOs should also enquire whether the spaymen has been seen by the community and record whether any sign of biocide granule (CG) can be seen. A special intervention spot check data sheet (see below) will help to record the observations. This data sheet can be used by CMSOs, inspectors and municipal coordinators. When ever late instar larvae or pupae can be observed in checked habitats or complains from the community are received immediate action has to be taken (contact ward supervisor, inspector and spraymen, identify source of and help solving problem).

Intervention Spot Checks

MUNICIPALITY: ________________ INTERVENTION WARD: ____________________

checked by (name and position): ______________________________

Date	Mtaa	TCU number	Plot ID	Habitat ID	Date of larviciding	Larval stage						Pupae		Sprayman seen?		Signs of CG?		Comments
						Anoph.			Culex									
						Absent	Early	Late	Absent	Early	Late	Absent	Present	Yes	No	Yes	no	

Research Permit
Bti and Bs products are not registered in Tanzania for individual or commercial use. Therefore, we applied for a research permit from the Tropical Pesticide Research Institute to use these products in the UMCP. Photocopies of the permit should be with all the ward supervisors.
