# Supplementary material for: A tool box for operational mosquito larval control: preliminary results and early lessons from the Urban Malaria Control Programme in Dar es Salaam, Tanzania
Source: Malar J. 2008 Jan 25;7:20. doi: 10.1186/1475-2875-7-20 (PMC2259364; doi:10.1186/1475-2875-7-20)
Supplement: Additional file 8 — Training presentation for larvicide application. The document shows a training presentation for ward and city based staff on how to apply microbial larvicides. [file 1475-2875-7-20-S8.pdf]

Calibration For Application of Microbial Mosquito Larvicides

Peter DeChant
Valent BioSciences Corporation
Libertyville, IL

Dar UMCP
Dar es Salaam
January 2006

VALENT BIOSCIENCES CORPORATION

Objective

Provide practical training in calibration for application of microbial mosquito larvicides for control of malaria vectors.

Agenda

- **The global malaria problem**
- Current strategies in malaria control
- Mosquito life cycle and control strategies
- Microbial mosquito larvicides
- Microbial mosquito larvicide formulations
- Application Equipment
- Calibration methods

A Global Problem

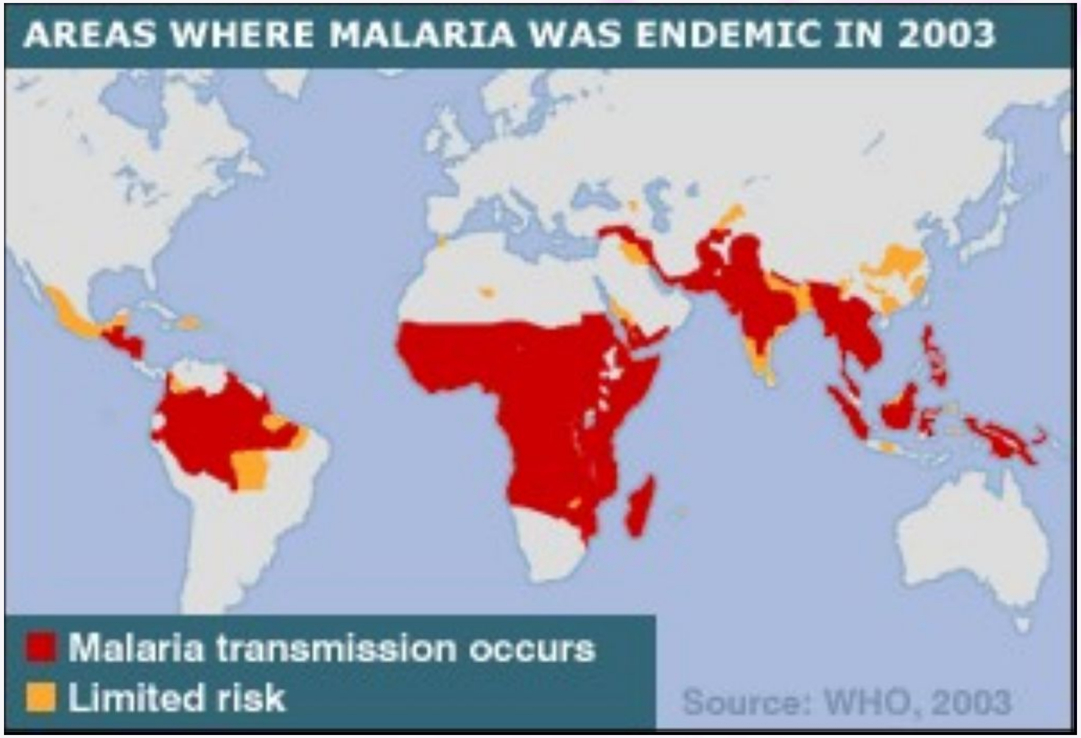

Image courtesy of The World Health Organization

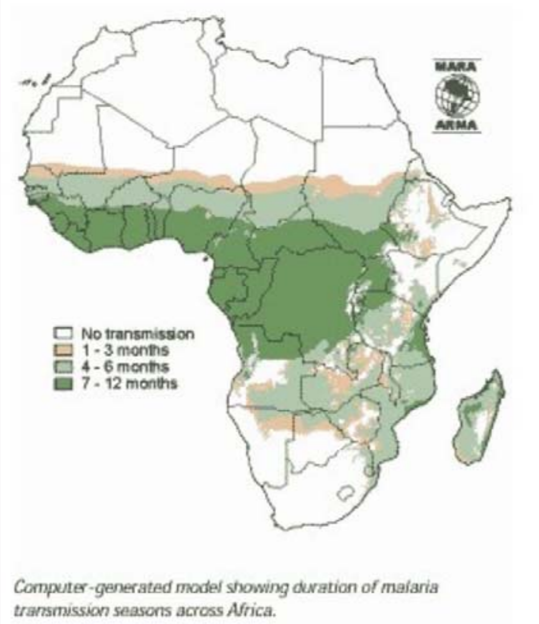

Image courtesy of Roll Back Malaria, World Health Organization

Dar UMCP
Dar es Salaam
January 2006

VALENT BIOSCIENCES CORPORATION

Malaria in Sub-Saharan Africa

Annual global burden of malaria (2002 estimates):

- **1.1 million deaths (mostly children)**
- **300-500 million cases**
- **44 million disability adjusted life years (DALYs)**
- **Reduction of GNP of more than half in Malaria endemic countries**

Over 90% of the disease burden is in sub-Saharan Africa, and almost all deaths (due to Plasmodium falciparum) occur in Africa.

The Special Programme for Research and Training in Tropical Diseases (TDR)

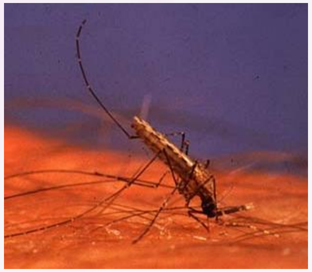

Malaria Control Challenges

- Vaccine not yet developed
- Multiple drug resistance
- Insecticide resistance
- Poverty (cause & effect)
- Infrastructure
- Local capacity

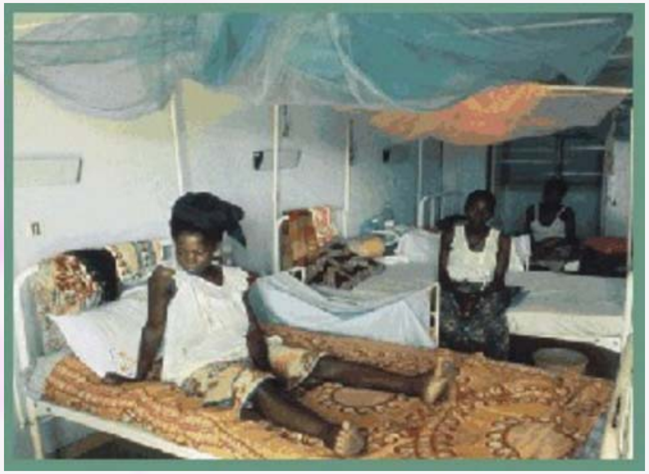

Image courtesy of The World Health Organization

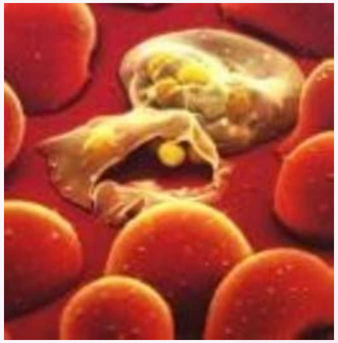

Copyright (c) traveldoctor.co.uk

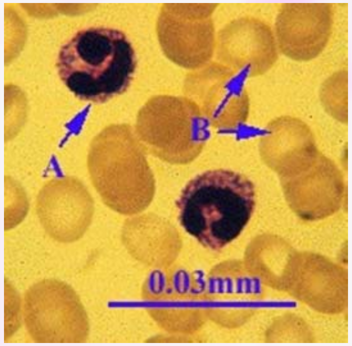

Copyright (c) 1998-2004 by
A. Richard Palmer & Ron Koss.

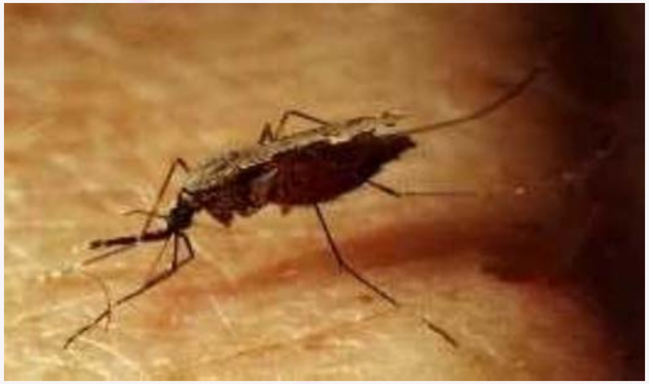

Image courtesy of The Ohio State University College of Biological Sciences

Dar UMCP
Dar es Salaam
January 2006

VALENT BIOSCIENCES CORPORATION

Agenda

- ✓ The global malaria problem
- ❑ **Current strategies in malaria control**
- ❑ **Mosquito life cycle and control strategies**
- ❑ Microbial mosquito larvicides
- ❑ Microbial mosquito larvicide formulations
- ❑ Application Equipment
- ❑ Calibration methods

Malaria Control Strategies

- Treating the ill and preventing transmission
  - Drug chemotherapies
  - Insecticide treated nets (ITN's)
  - **Larviciding**

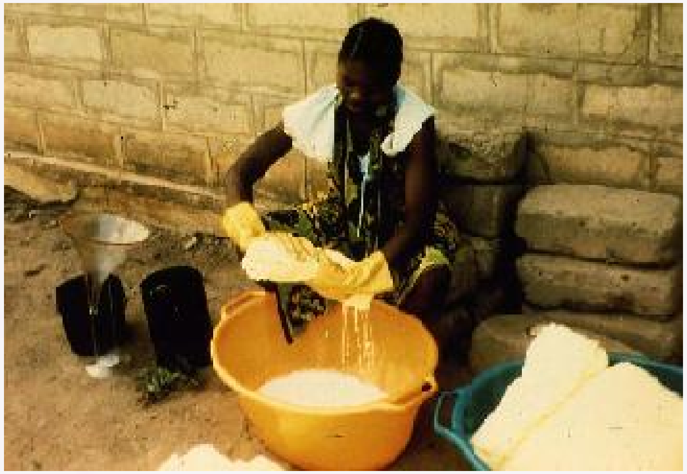

PHOTO COURTESY OF C.F. CURTIS - U of M Website

Mosquito Life Cycle

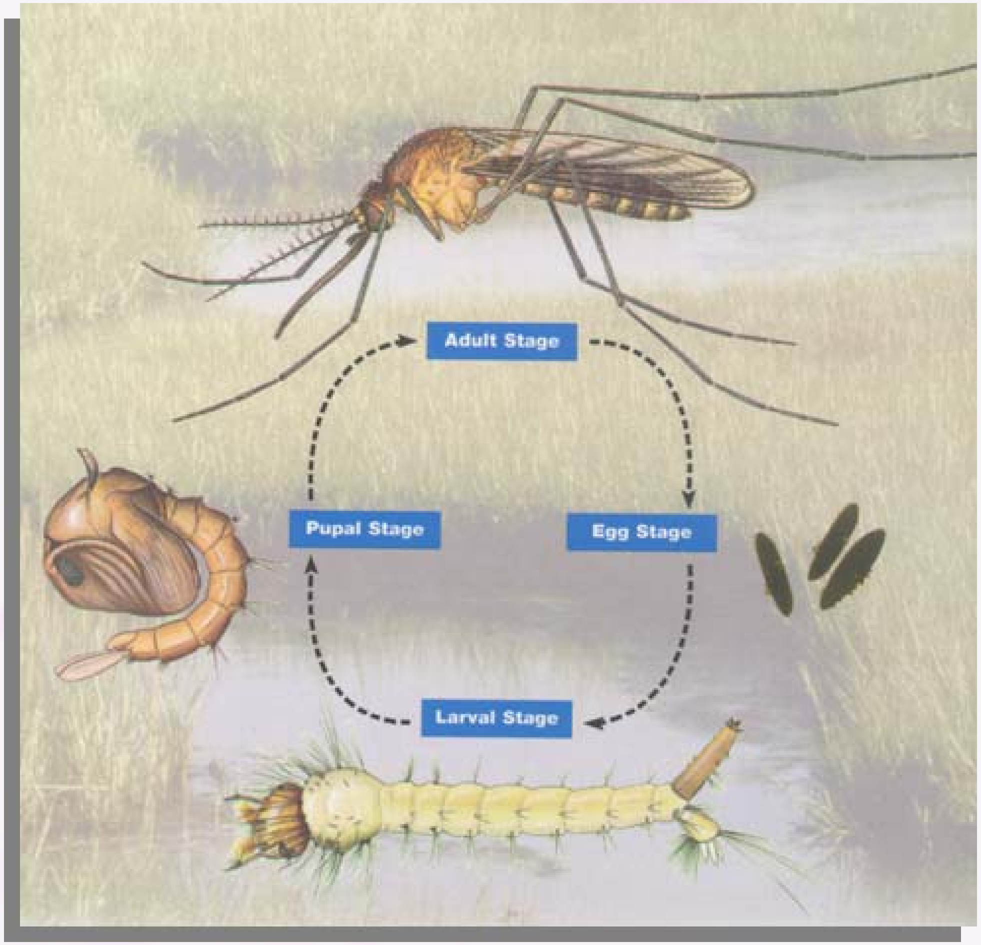

Methods of Mosquito Control

Source Reduction

Larviciding

Adulticiding

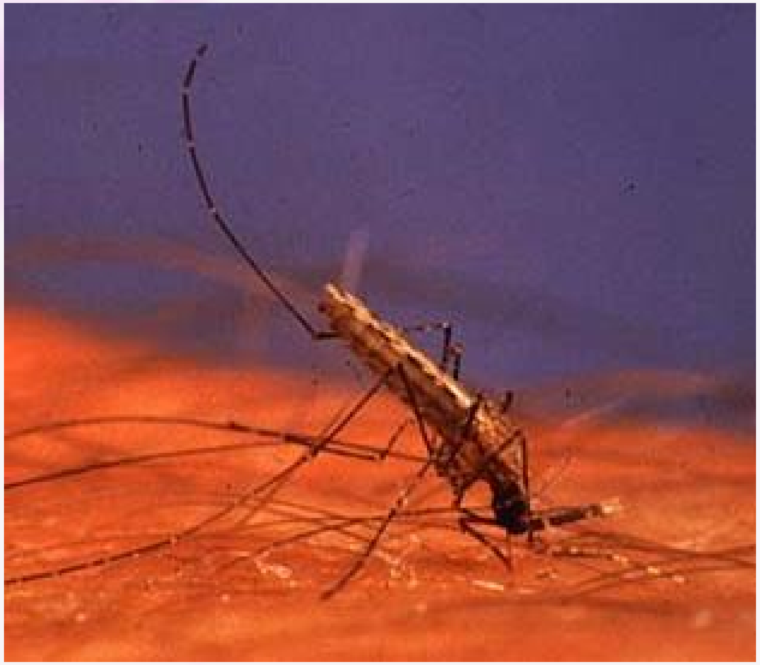

PHOTO COURTESY OF C.F. CURTIS - U of M Website

American Mosquito Control Association's Pesticide Environmental Stewardship Program Strategy Document

Dar UMCP
Dar es Salaam
January 2006

VALENT BIOSCIENCES CORPORATION

Source Reduction

(Environmental Management)

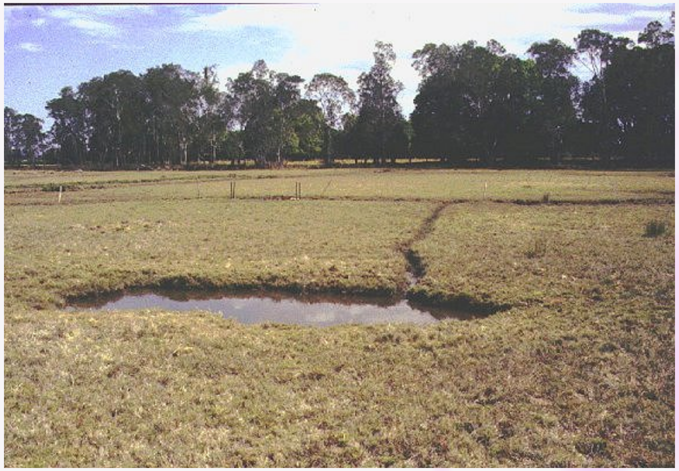

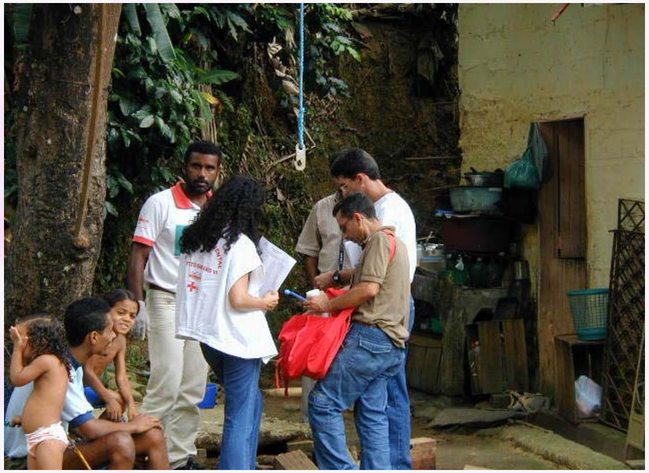

- Removal or reduction of mosquito larval habitats
  - Drainage
  - Sanitation or hygiene
  - Community Participation

VALENT BIOSCIENCES CORPORATION

Larviciding

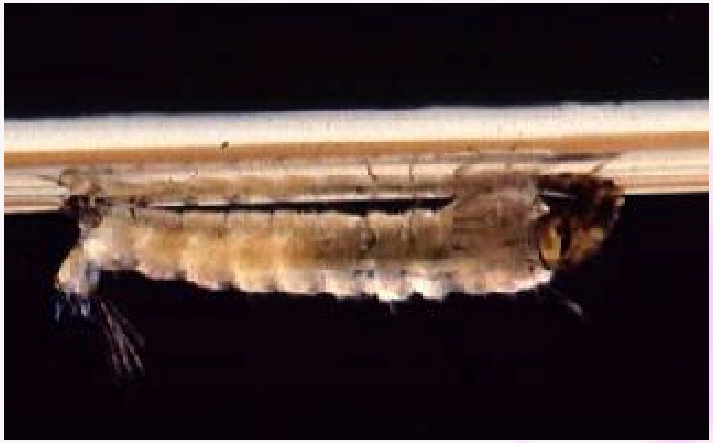

PHOTO COURTESY OF C.F. CURTIS - U of M Website

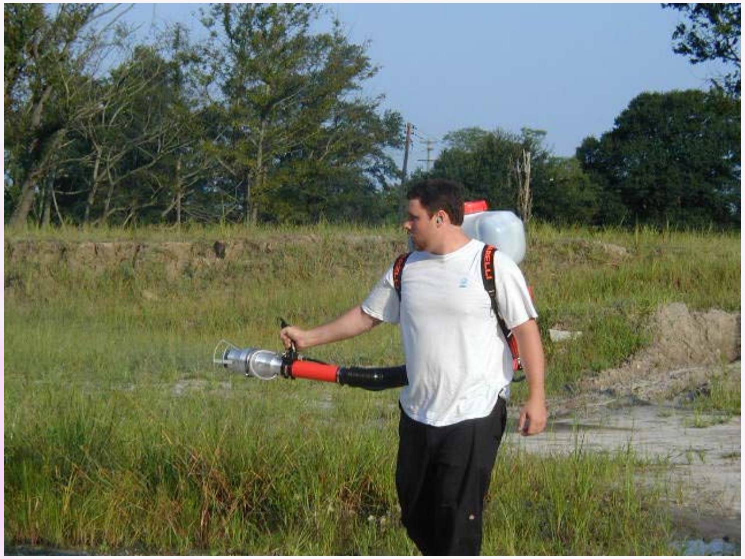

- Application of substances to kill mosquito larvae or pupae in water
  - Liquid spray, granular application, direct application
  - No loosers

Adulticiding

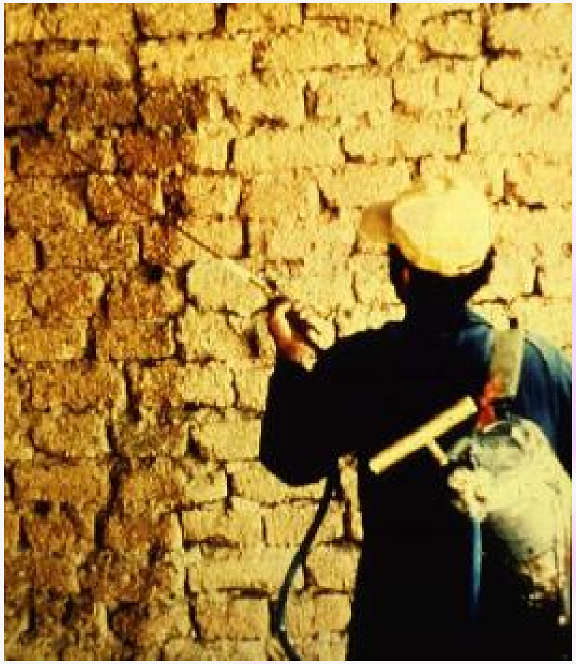

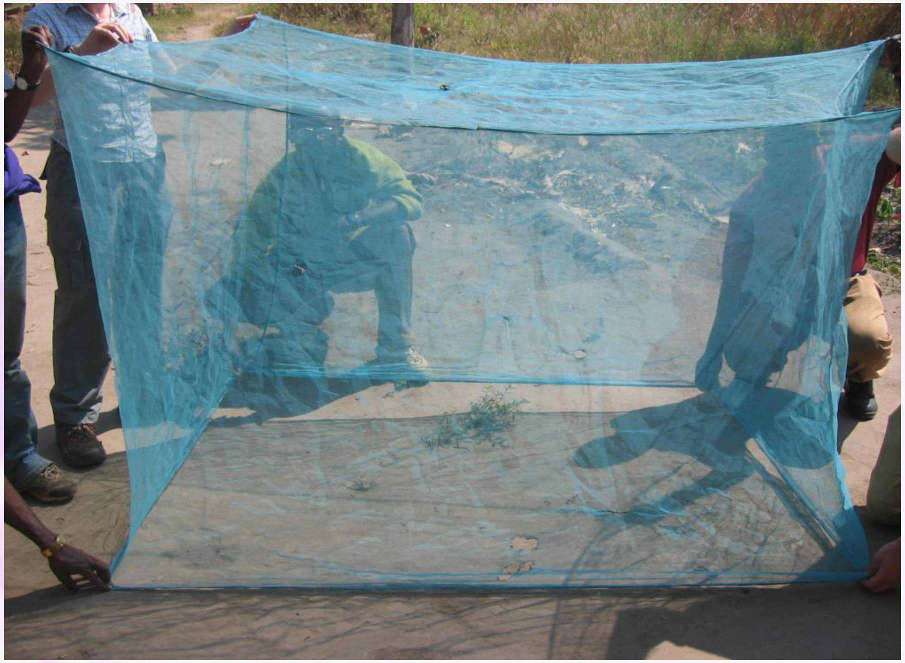

PHOTO COURTESY OF C.F. CURTIS - U of M Website

- Application of chemicals to kill adult mosquitoes
  - Residual spray & ITN.

Larviciding Philosophy (CIA)

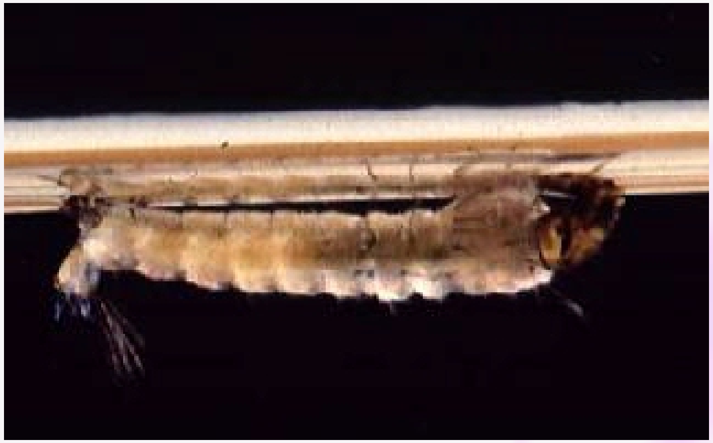

PHOTO COURTESY OF C.F. CURTIS - U of M Website

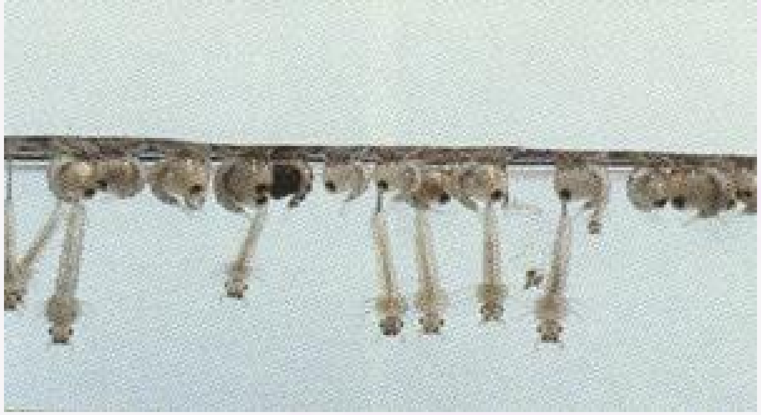

- Mosquito Larvae are generally:
  - **C**oncentrated
  - **I**mmobile
  - **A**ccessible
- Adult mosquitoes spread out over a much larger area.
- **CIA** = efficiency of larval control.

VALENT BIOSCIENCES CORPORATION

New Look at Environmental Management and Larval Control

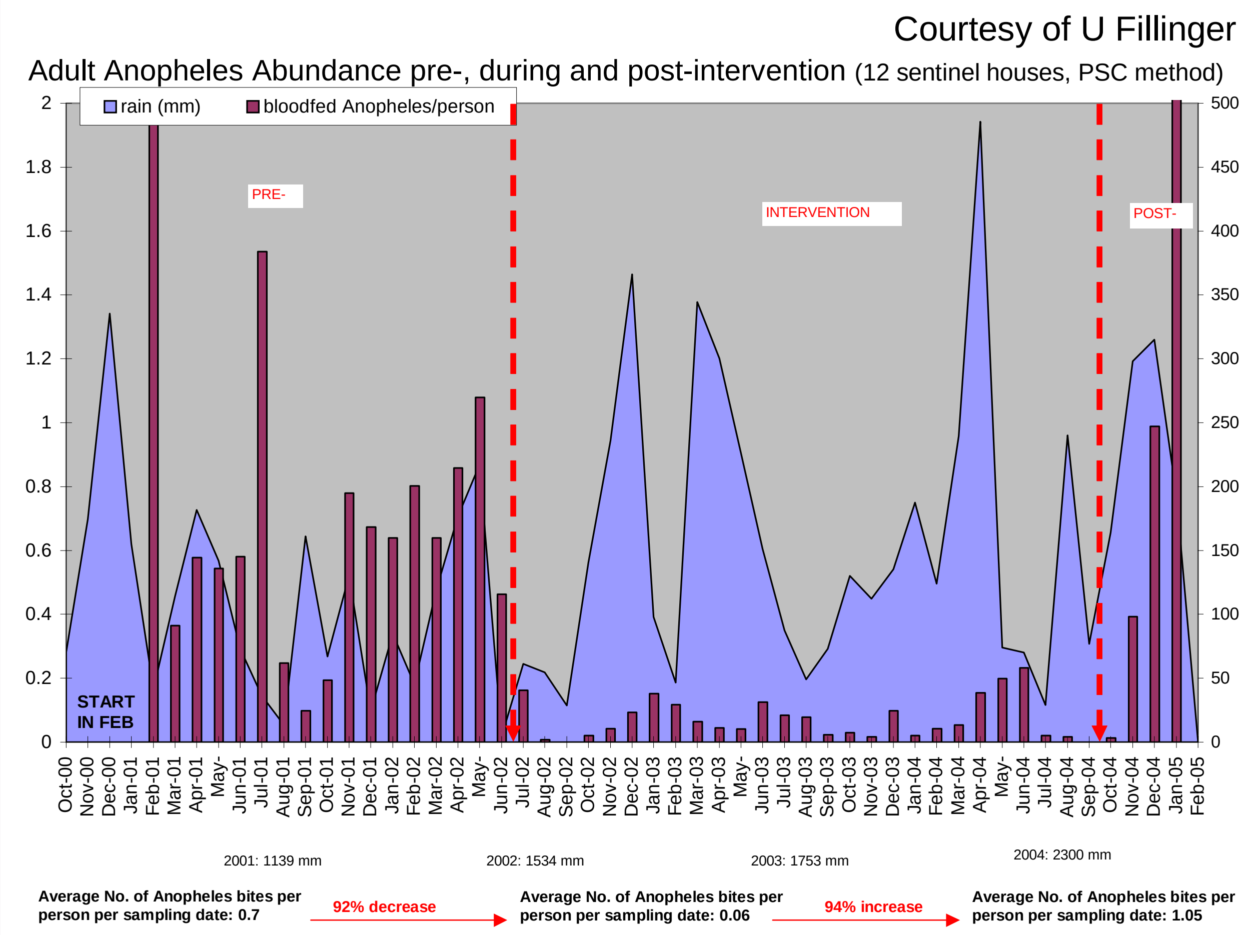

Dar UMCP
Dar es Salaam
January 2006

VALENT BIOSCIENCES CORPORATION

Agenda

- ✓ The global malaria problem
- ✓ Current strategies in malaria control
- ✓ Mosquito life cycle and control strategies
- ❑ **Microbial mosquito larvicides**
- ❑ Microbial mosquito larvicide formulations
- ❑ Application Equipment
- ❑ Calibration methods

Mosquito Larvicides

- Chemicals
  - OP's (temephos)
- Surface Agents
  - oils, monomolecular films
- **Microbials**
  - ***Bacillus thuringiensis israelensis (Bti)***
  - ***Bacillus sphaericus (Bs)***

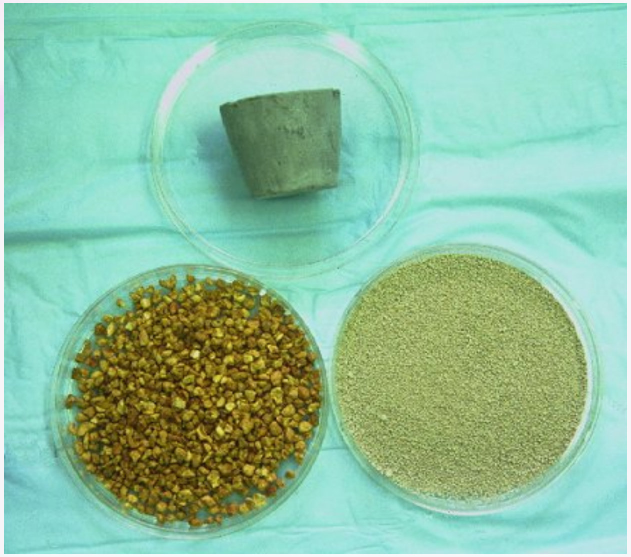

B. thuringiensis subsp. *israelensis* (*Bti*)

VectoBac = *Bti*

Bacteria that produces 5 toxins (ICP)
ICP = Insecticidal Crystal Protein
Protein is not toxic until digested by larvae

ICP

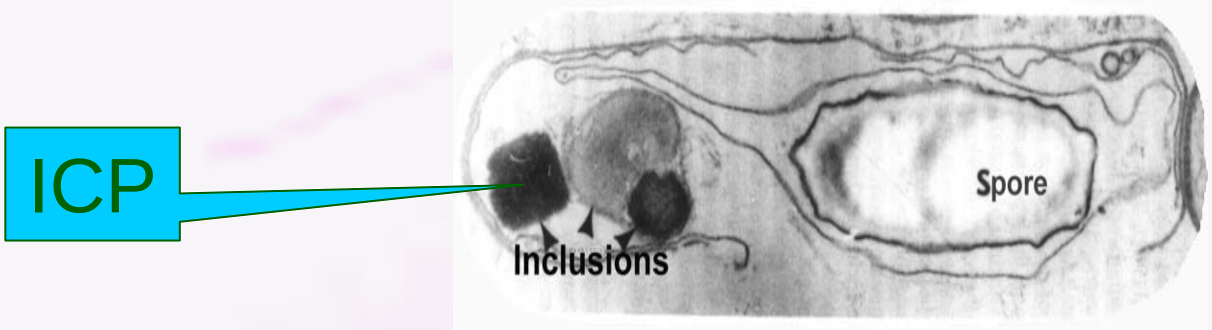

Bti

VALENT BIOSCIENCES CORPORATION

Bacillus sphaericus (Bs)

VectoLex = *Bs*

Bacteria that produces 2 toxins (ICP)
ICP = Insecticidal Crystal Protein
Protein is not toxic until digested by larvae

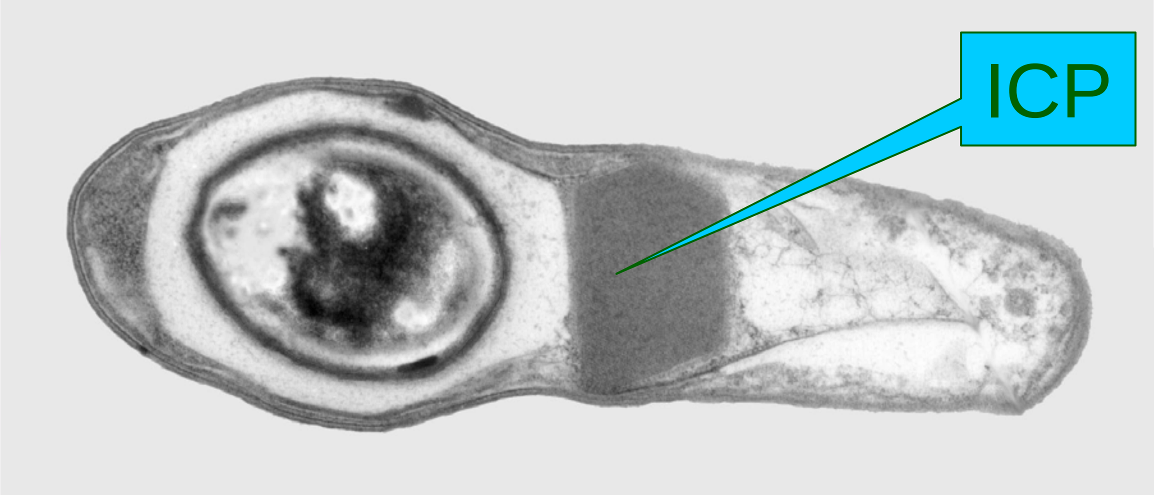

Figure courtesy of Jean-François Charles

Mode of Action

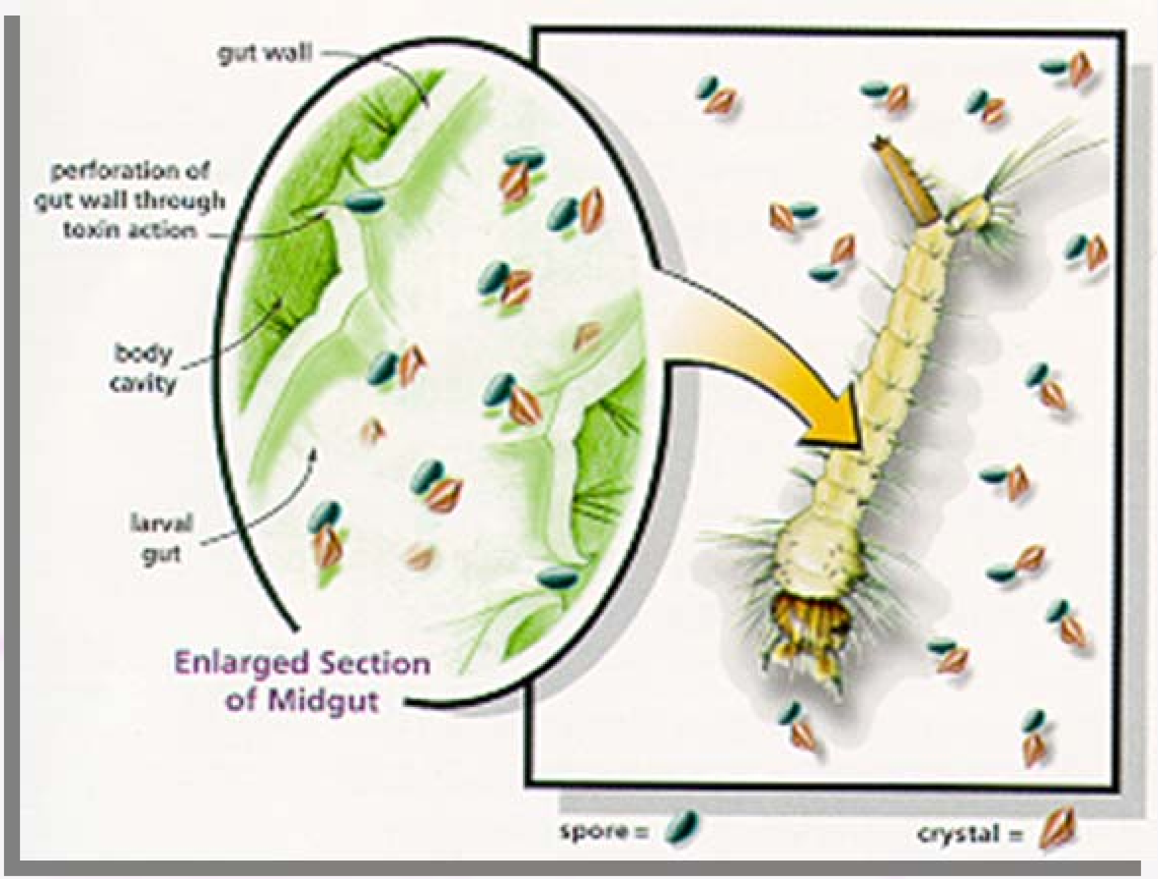

ICP

VALENT BIOSCIENCES CORPORATION

Insecticidal Crystal Protein

The larvae’s Last Meal

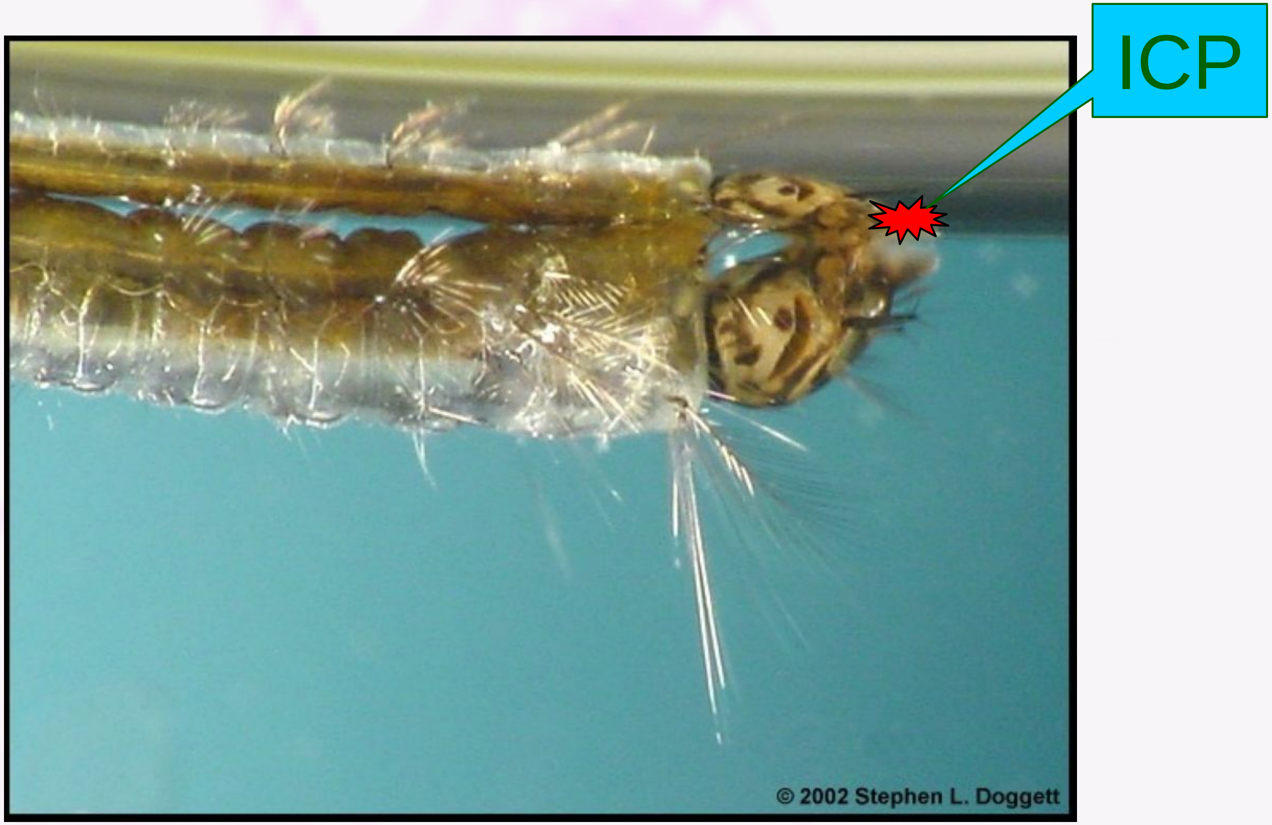

Figure courtesy of Stephen L. Doggett

Dar UMCP
Dar es Salaam
January 2006

VALENT BIOSCIENCES CORPORATION

OUR GOAL
"Give all the larvae a good FINAL meal."

- ICP's are not contact poisons
- Effective dose must be eaten by all larvae
- ICP's are not water soluble
  - Will not move laterally (diffusion)
- Total area needs to be evenly treated
- Must penetrate vegetation

OUR GOAL
“Give all the larvae a good FINAL meal.”

- KEYS TO OUR GOAL:
  - **FORMULATION**
    - Delivers ICP to the feeding zone
  - **APPLICATION**
    - Proper dose and even coverage

Agenda

- ✓ The global malaria problem
- ✓ Current strategies in malaria control
- ✓ Mosquito life cycle and control strategies
- ✓ Microbial mosquito larvicides
- ❑ **Microbial mosquito larvicide formulations**
- ❑ **Application Equipment**
- ❑ Calibration methods

MICROBIAL LARVICIDE FORMULATIONS

- **Granules (on corncob) - CG**
- **Water dispersible granules - WDG**
- Tablets - DT
- Water soluble pouches – WSP
- Aqueous suspensions - AS
- Technical powders - TP

VALENT BIOSCIENCES CORPORATION

VectoBac® and VectoLex® Formulations CG & WDG

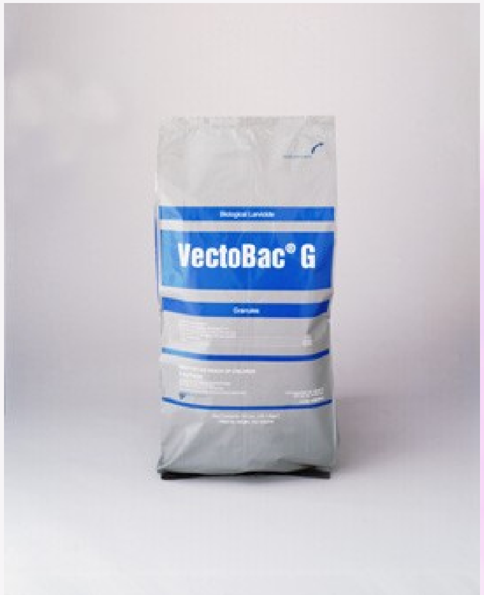

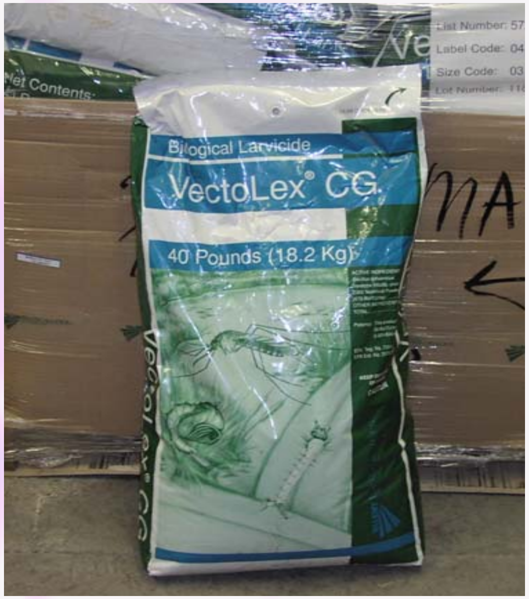

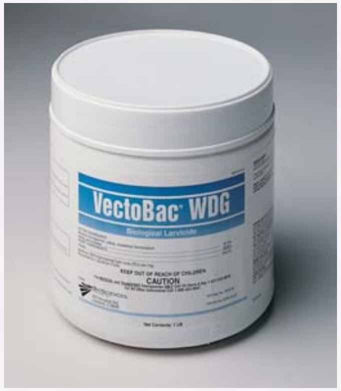

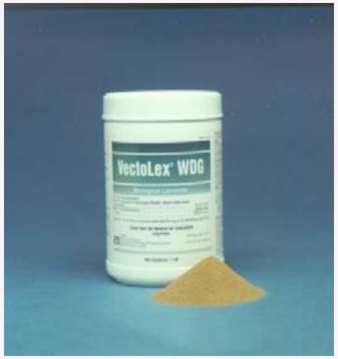

Dar UMCP
Dar es Salaam
January 2006

VALENT BIOSCIENCES CORPORATION

VectoLex® and VectoBac® CG

Granular formulations for dry application

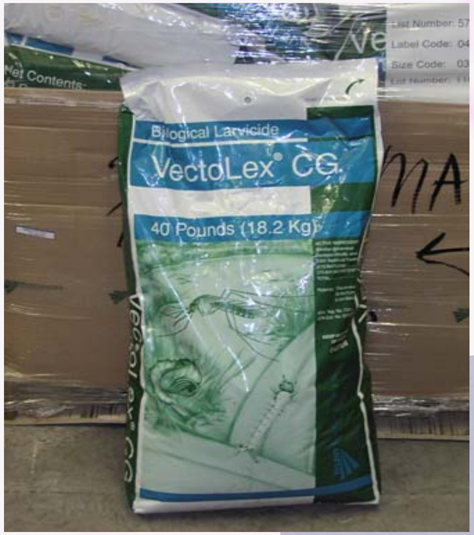

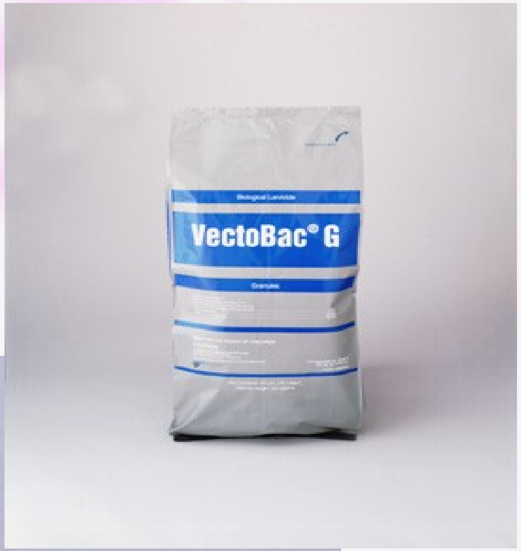

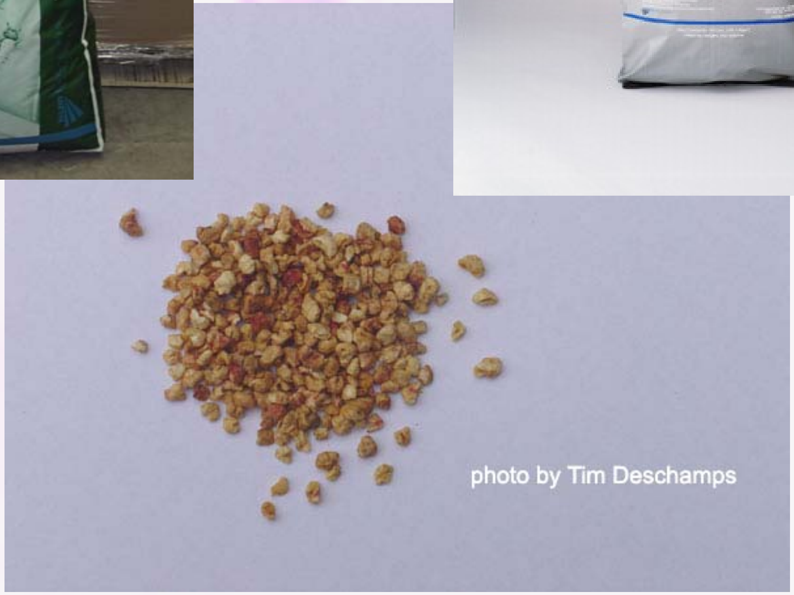

Dar UMCP
Dar es Salaam
January 2006

VALENT BIOSCIENCES CORPORATION

Why Choose CG Formulation?

- Stable formulations
- No mixing required
- Penetrates vegetation
- Can be hand applied to small areas easily by community members

Examples of Equipment for CG Application

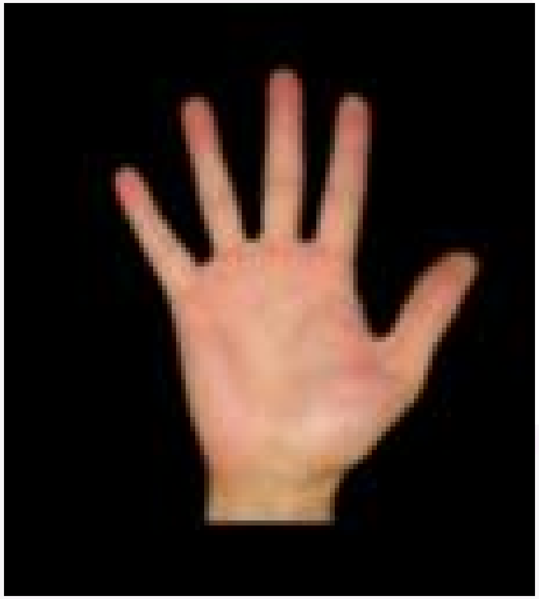

Hand

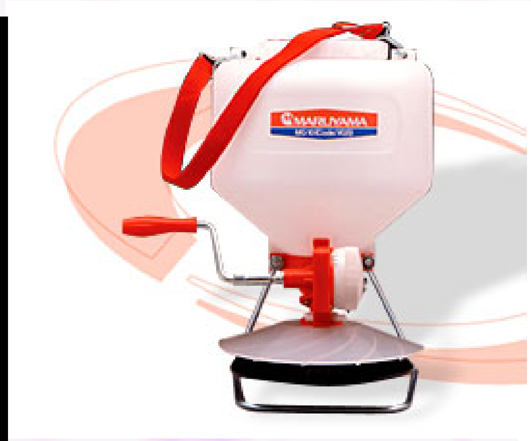

Manual

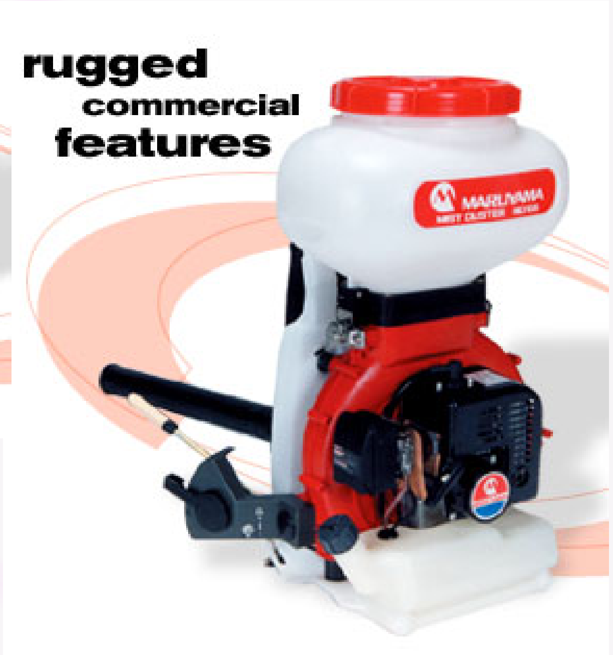

Power

Agenda

- ✓ The global malaria problem
- ✓ Current strategies in malaria control
- ✓ Mosquito life cycle and control strategies
- ✓ Microbial mosquito larvicides
- ✓ Microbial mosquito larvicide formulations
- ✓ Application Equipment
- ❑ **Calibration methods**

What is Calibration?

Dar UMCP
Dar es Salaam
January 2006

VALENT BIOSCIENCES CORPORATION

Why Calibrate?

Dar UMCP
Dar es Salaam
January 2006

Why Calibrate?

REMEMBER OUR GOAL

“Give all the larvae a good FINAL meal.”

- Accurate dose and even coverage of the larval habitat.
- Saves material, time and money.

VectoBac CG dose is 10 kg/ha

We aim to achieve this dose.

VectoBac® CG Calibration Method

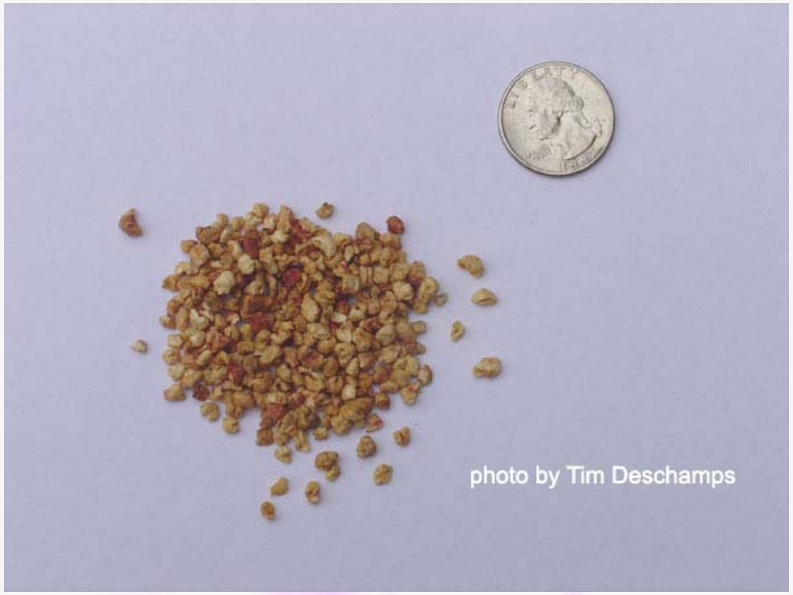

How do we apply the right amount?

Rate is 10 KG per hectare.

Think of this as granules per square meter.

Granules Are Applied By Hand

- Your hands and feet are the application tool.
- You must learn the weight of granules in your handful or measure with teaspoon.
- You must learn the distance of your step.
- Knowing these, you can develop the skill to make an even application at the correct dose.

Two Methods

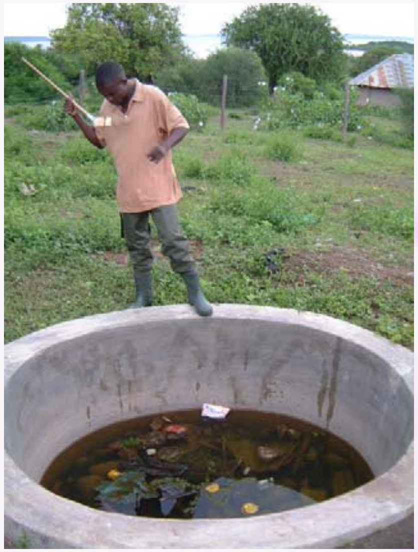

Small Areas

(<3 meters x 3 meters)

Large Areas

(>3 meters x 3 meters)

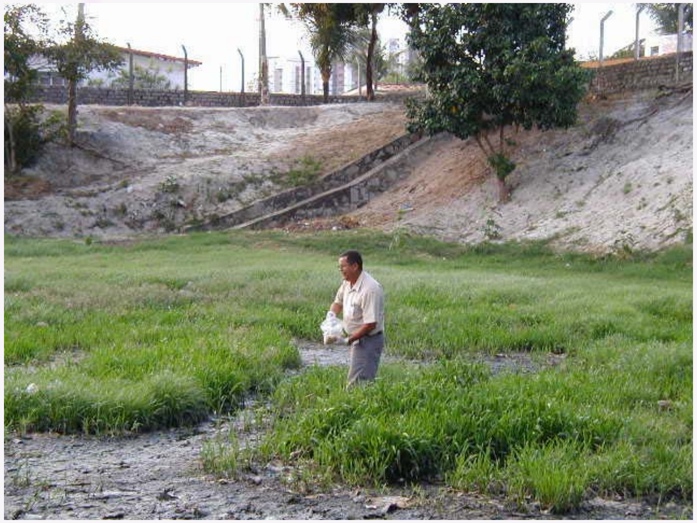

Hand Application of Granules

For Small Areas (< 3 meters x 3 meters)

- Rate = 1 gram per square meter (1/2 teaspoon)
- Know the size of the area
- Spread small amounts at a time to make application even.
- Was there enough to finish? (Was it too much?)
- Check if application "looks OK"

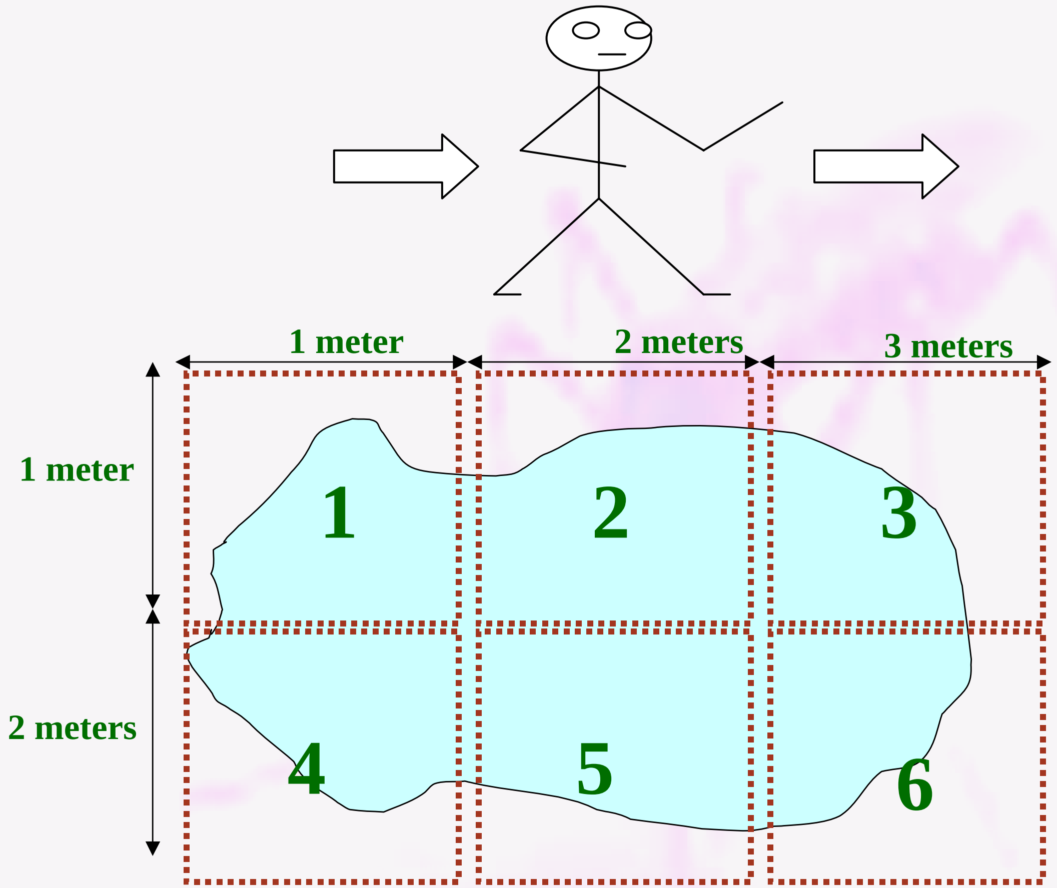

2 x 3 = 6 square meters

Needs 6 grams

Hand Application Rates

KG/HA	VectoBac CG<br>410 granules/gram	
	# PER M2	10 cm x 10 cm
5	205	2
**10**	**410**	**4**
15	615	6
20	820	8

Good Hand Application Rate

10 Kilogram/Hectare (AT LEAST FOUR)

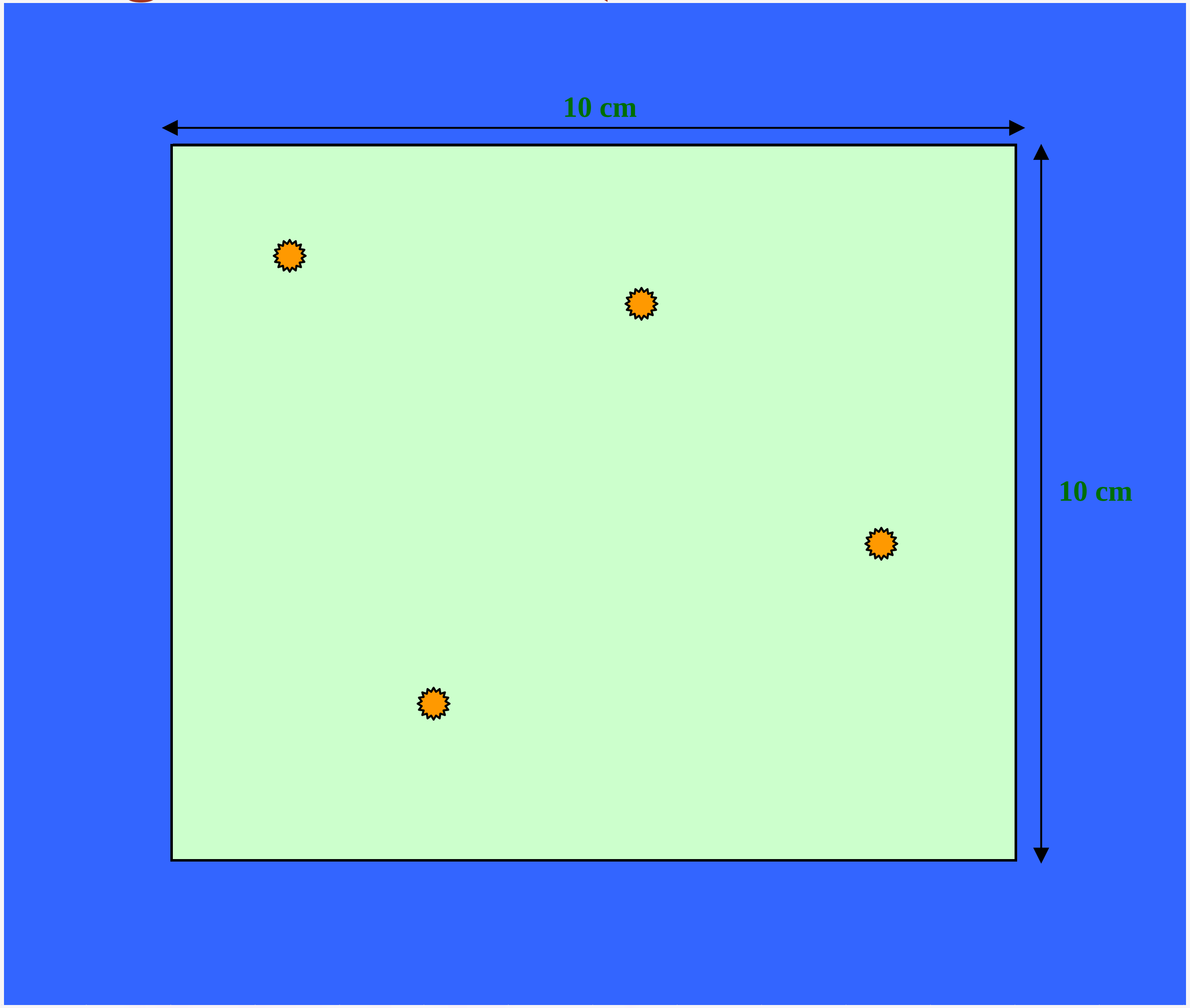

Dar UMCP
Dar es Salaam
January 2006

VALENT BIOSCIENCES CORPORATION

HAND APPLICATION RATES of CG

To Large Areas (> 3 meters x 3 meters)

- Your Walking STEP (meters per step)
- SWATH (3 meters wide)
- Weight of your HANDFUL (of granules)

- How many steps do we take per handful?

VALENT BIOSCIENCES CORPORATION

Calibration Steps for CG Hand Application

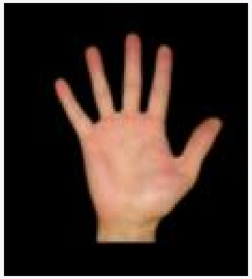

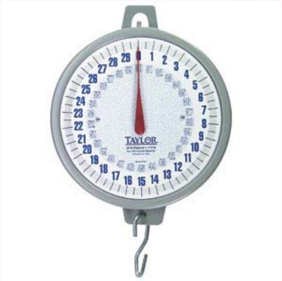

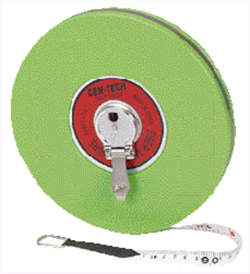

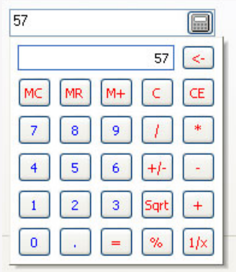

- Measure your STEP
- Know your SWATH (3 meters)
- Know your HANDFUL WEIGHT
- Determine how many steps for each HANDFUL

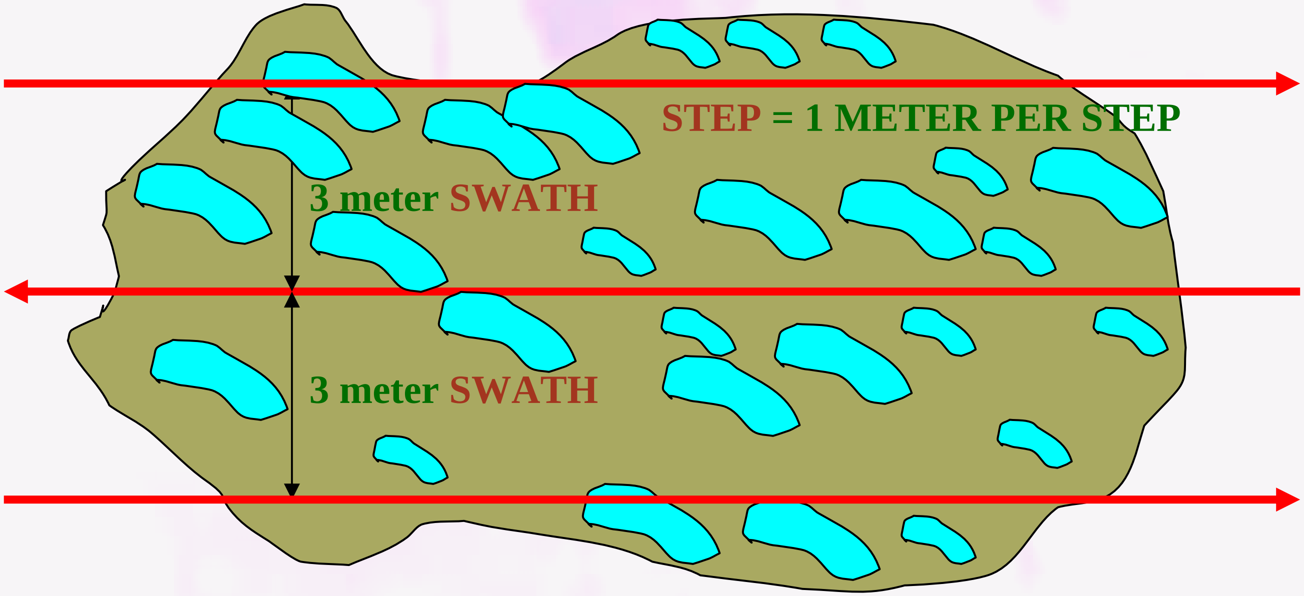

Dar UMCP
Dar es Salaam
January 2006

VALENT BIOSCIENCES CORPORATION

Hand Calibration for CG

RATE = (HANDFUL)/ (STEPS PER HADFUUL x SWATH x STEP)

STEPS PER HANDFUL = HANDFUL/(STEP x SWATH)

RATE = GRAMS PER SQUARE METER = 1 GRAM PER METER SQUARE
STEP = METERS PER STEP
SWATH = 3 METERS
HANDFUL = GRAMS PER HANDFUL

VALENT BIOSCIENCES CORPORATION

Example for VectoLex CG

RATE = GRAMS PER SQUARE METER = 1 GRAM PER METER SQUARE
STEP = METERS PER STEP = 1 METER
SWATH = 3 METERS
HANDFUL = GRAMS PER HANDFUL = 15

STEPS PER HANDFUL = 5
TOTAL HANDFULS = 12 HANDFULS

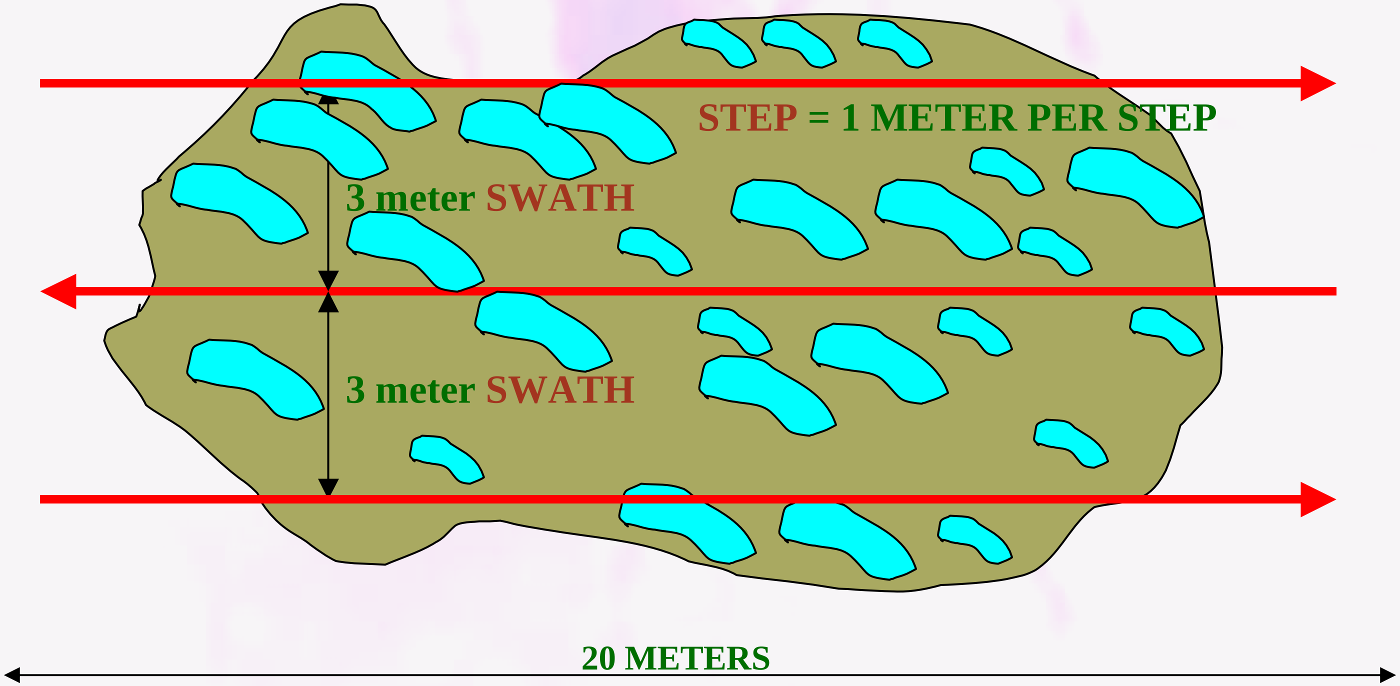

Dar UMCP
Dar es Salaam
January 2006

VALENT BIOSCIENCES CORPORATION

Verification of Application

- Does actual use match expected use.
  - Size of each area treated
  - Rates intended
  - Overall inventory vs use accounting
  - End of day match?
- Do the applications "look OK"

Spraying Strategies

- Responsibility for product & equipment

- Safety

- Team Work

- Material Transport – Backpack

- Start on edge
(Better to spray some land than miss water)

- Reporting material use

Take Pride in your work.
Every mosquito you kill could mean one less person getting malaria!

It will take teamwork roll back malaria.

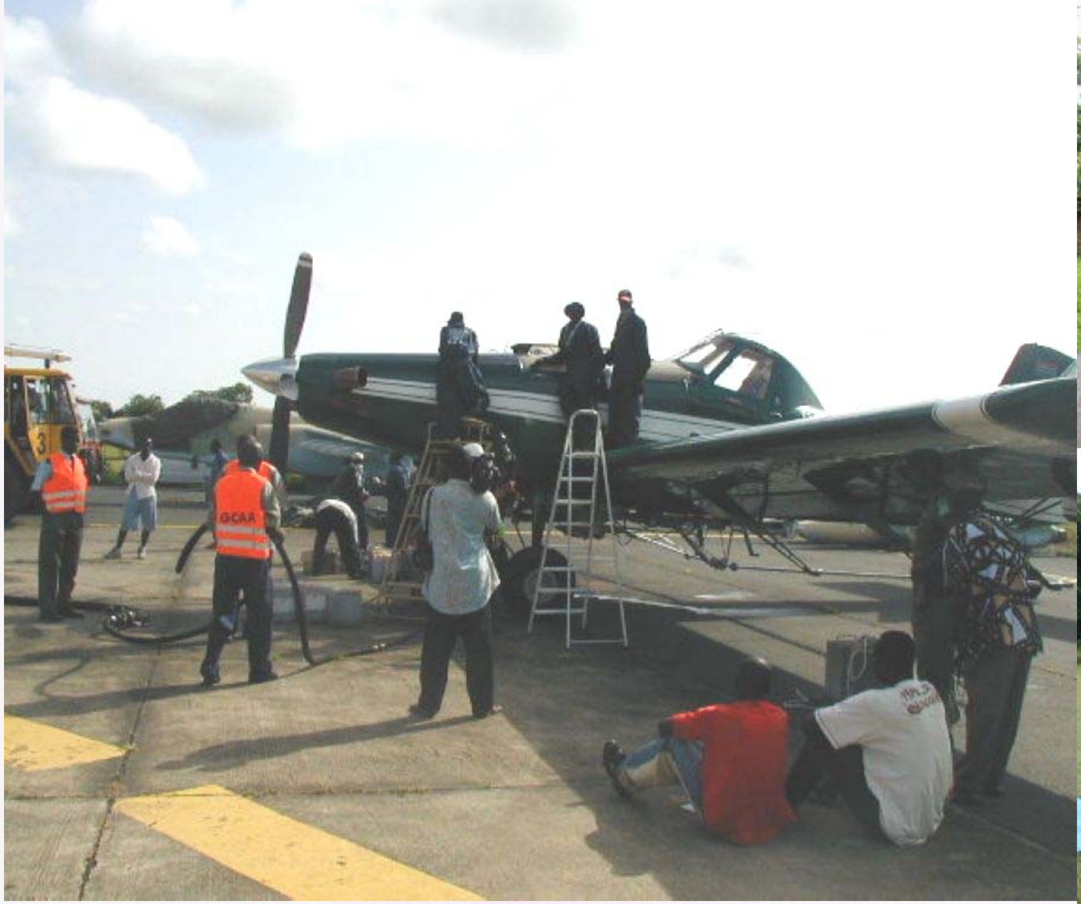

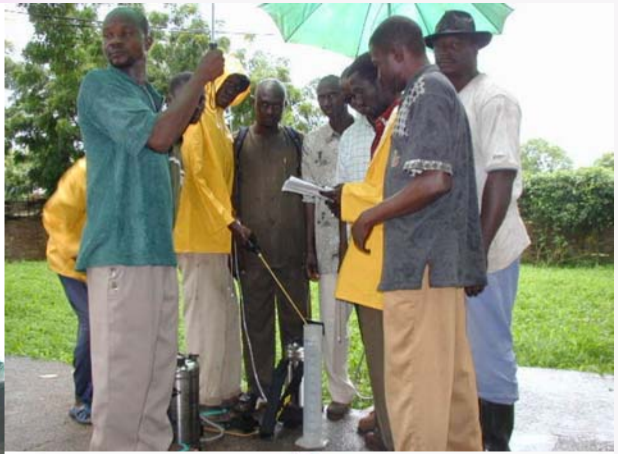

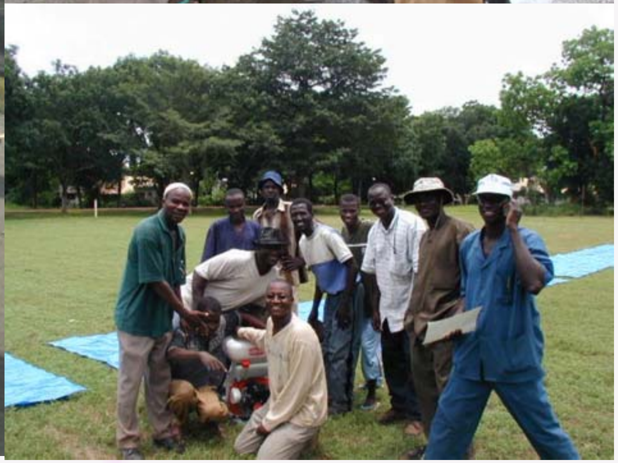

Dar UMCP
Dar es Salaam
January 2006

VALENT BIOSCIENCES.
CORPORATION

Let’s all push!

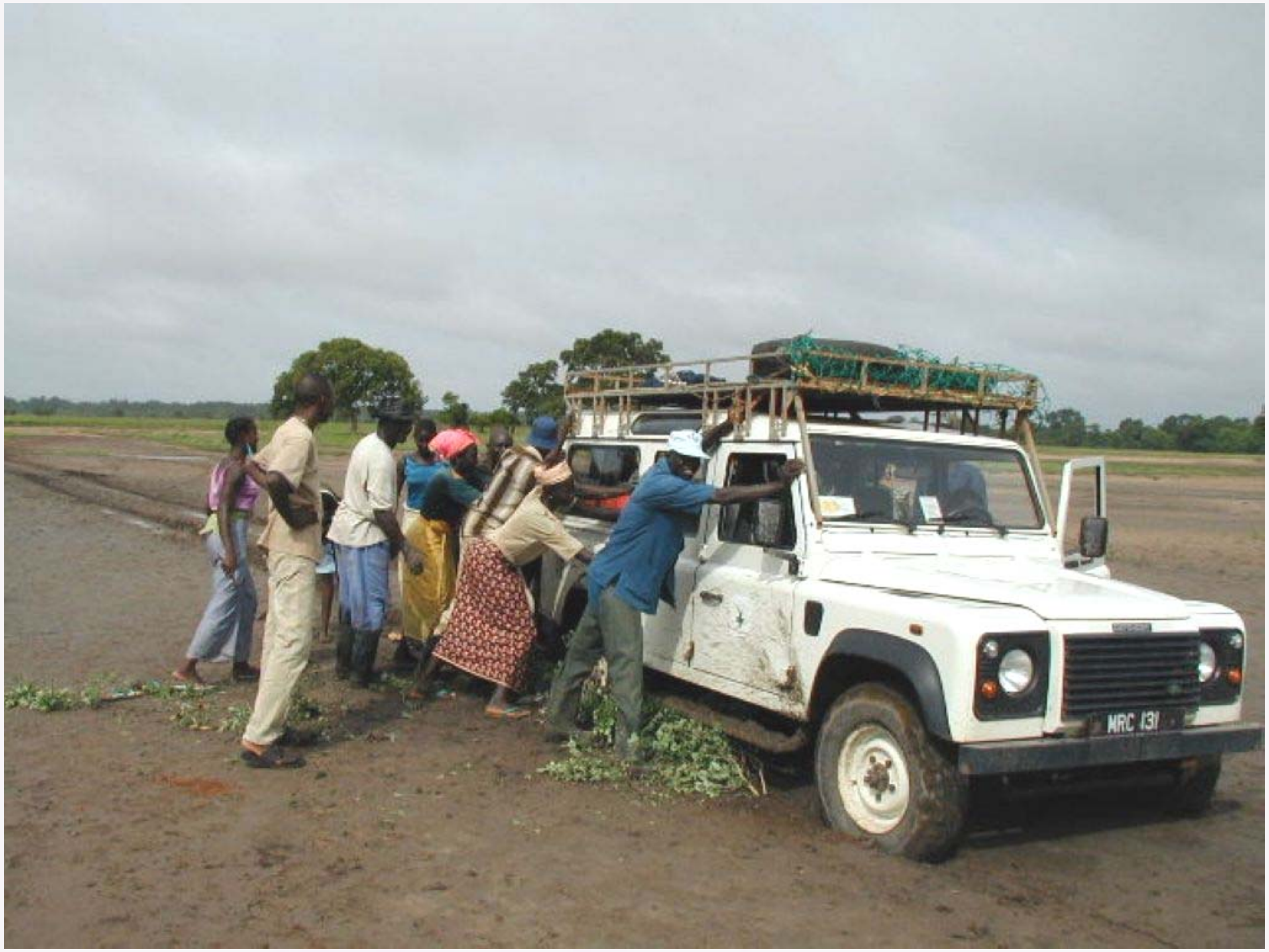

Dar UMCP
Dar es Salaam
January 2006

Calibration For Application of VectoBac WDG

Peter DeChant
Valent BioSciences Corporation
Libertyville, IL

Dar UMCP
Dar es Salaam
January 2006

VALENT BIOSCIENCES CORPORATION

Objective

Provide practical training in calibration for application of VectoBac WDG for control of malaria vectors.

Agenda

- **Microbial mosquito larvicide formulations**
- **VectoBac WDG**
- Application Equipment
- Calibration methods
- Verification

VALENT BIOSCIENCES CORPORATION

MICROBIAL LARVICIDE FORMULATIONS

- **Granules (on corncob) - CG**
- **Water dispersible granules - WDG**
- Tablets - DT
- Water soluble pouches – WSP
- Aqueous suspensions - AS
- Technical powders - TP

VALENT BIOSCIENCES CORPORATION

VectoBac® and VectoLex® Formulations CG & WDG

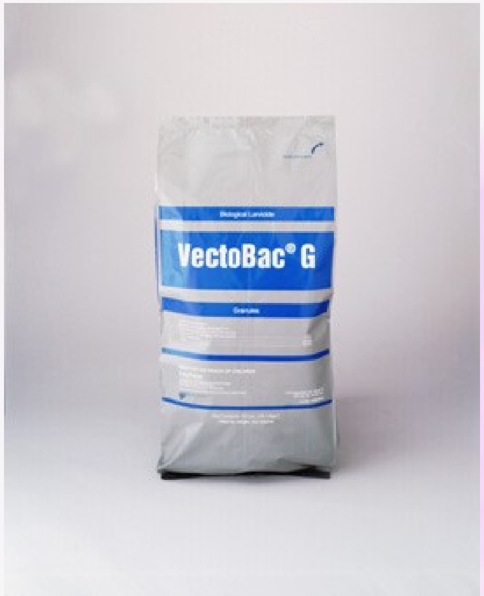

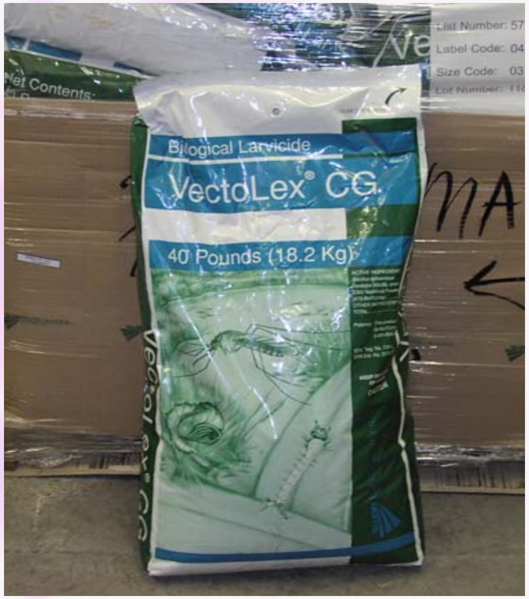

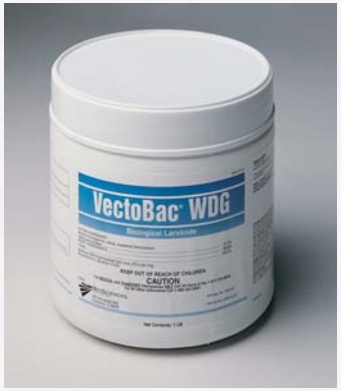

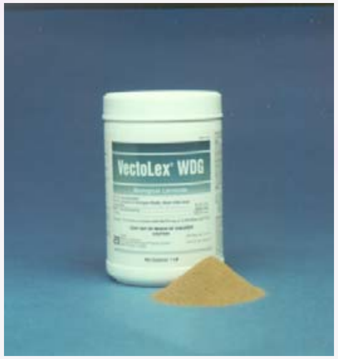

Dar UMCP
Dar es Salaam
January 2006

VALENT BIOSCIENCES CORPORATION

VectoBac® WDG

The stability of a granule with the application flexibility of a liquid.

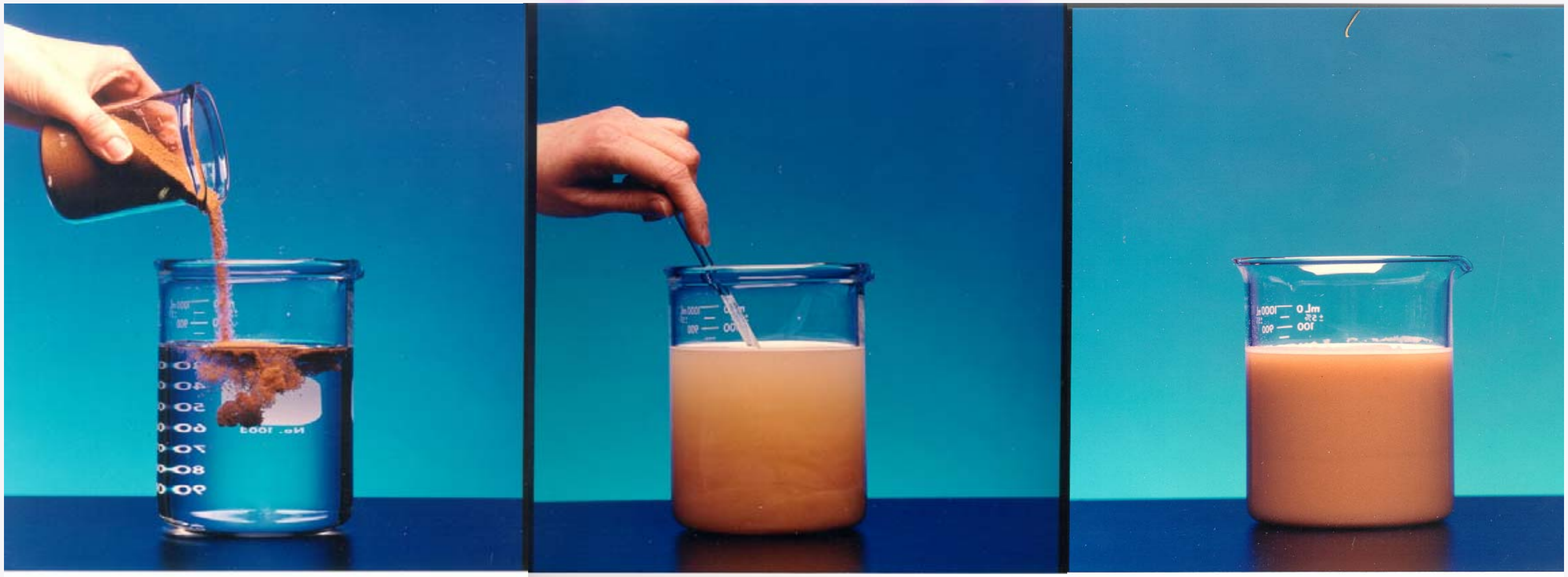

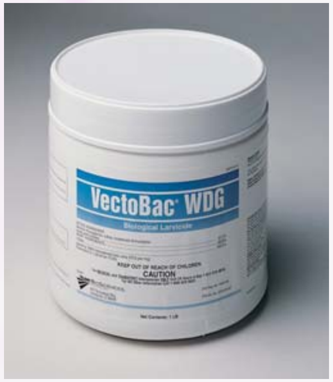

Dar UMCP
Dar es Salaam
January 2006

VALENT BIOSCIENCES CORPORATION

When Choose WDG Formulation?

- **Large areas with open water**
  - Breeding sites larger than 2000 sq meters (45m x 45m)
  - *Larger than 15 swaths x 15 swaths (granules)?*
  - Breeding sites requiring more than one pack (2 kg) of granules to treat
  - Inform Ward Supervisor and Inspector
- **Why?**
  - Less product to carry into the field
  - More area covered before returning
  - More economical
  - Backpack spray = wide swath

VectoBac WDG

Ideal particle size and suspension characteristics in water

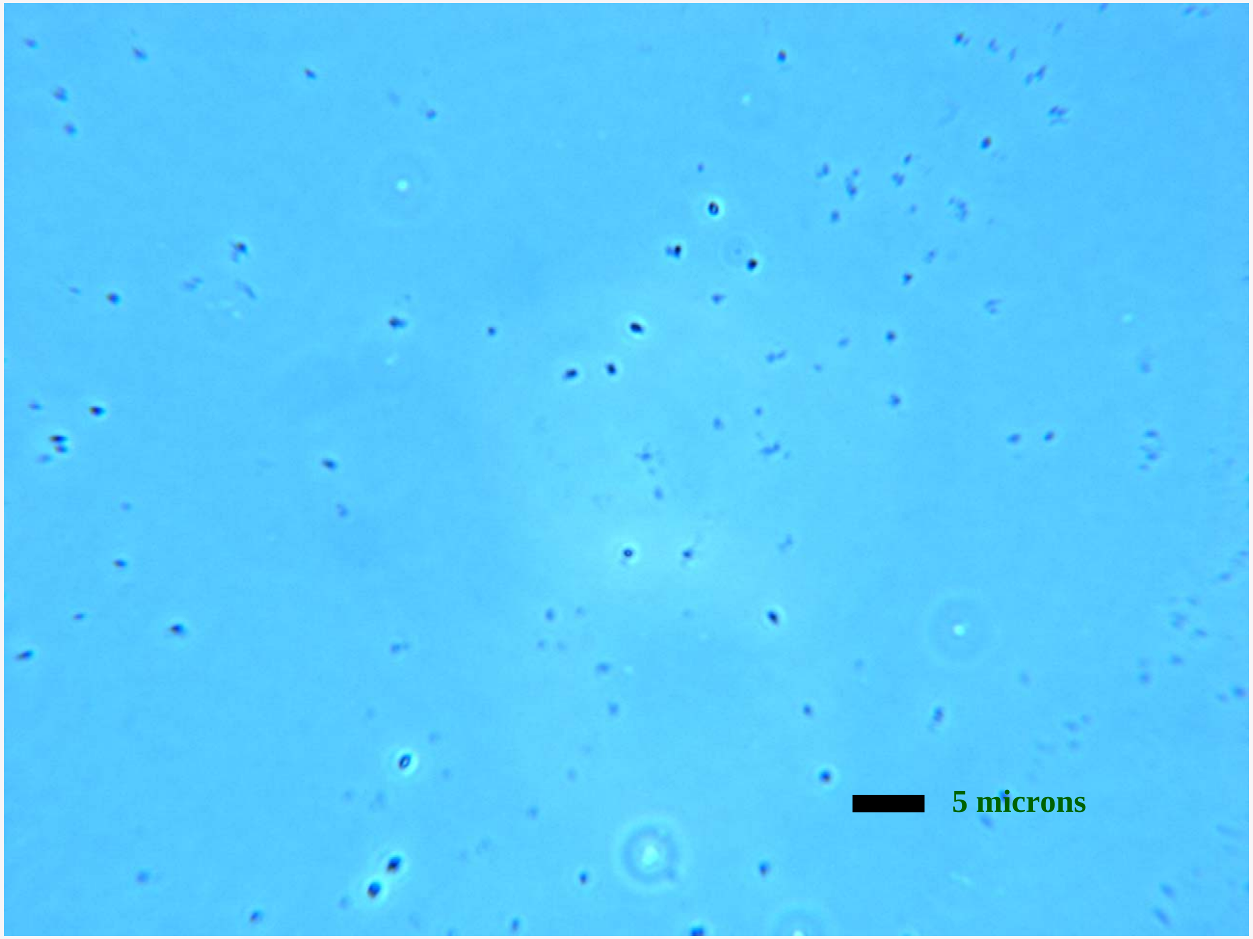

Dar UMCP
Dar es Salaam
January 2006

VALENT BIOSCIENCES CORPORATION

Insecticidal Crystal Protein - The larvae’s Last Meal

Small particles suspend in feeding zone

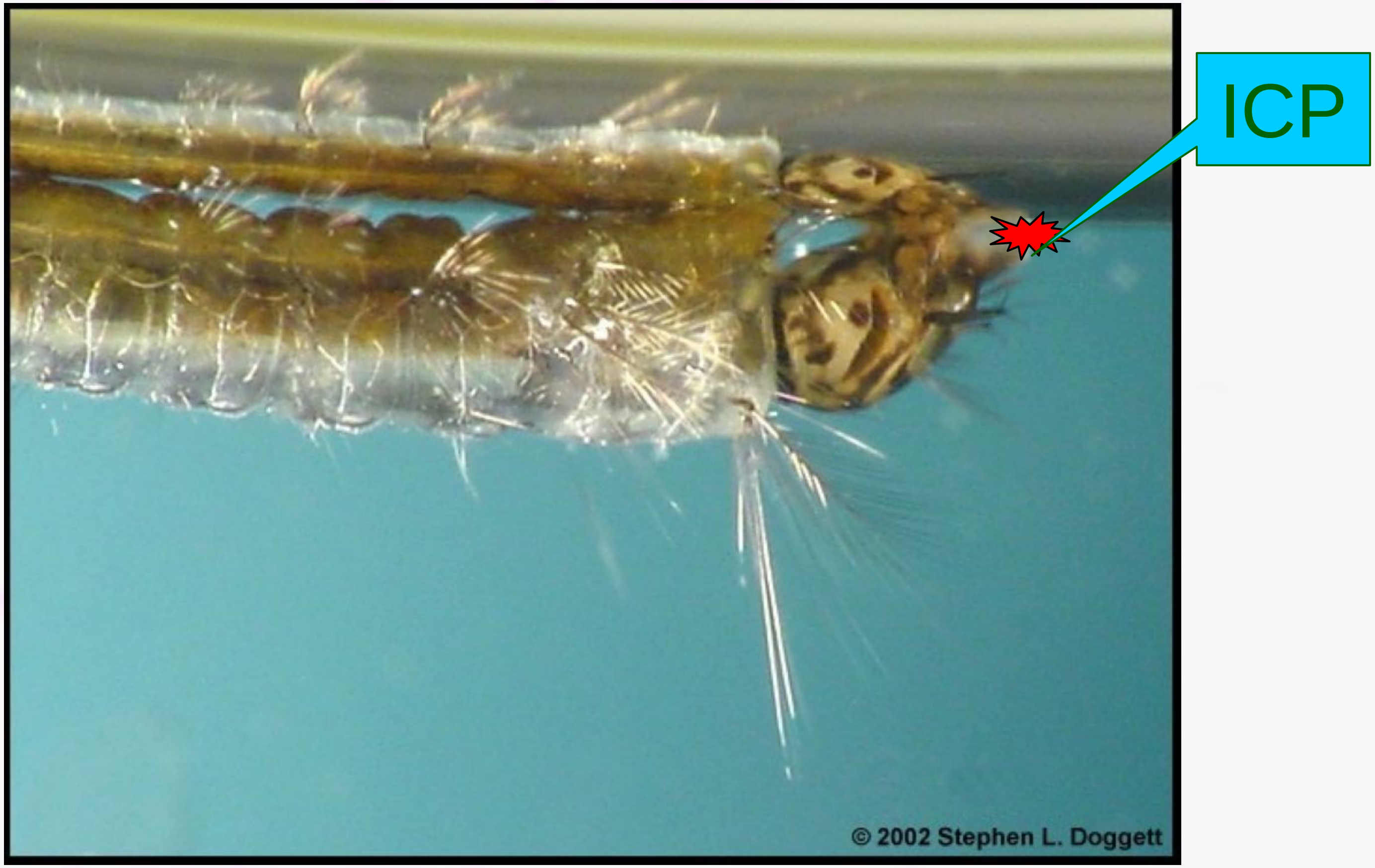

Figure courtesy of Stephen L. Doggett

VALENT BIOSCIENCES CORPORATION

Agenda

- ✓ Microbial mosquito larvicide formulations
- ✓ VectoBac WDG
- ❑ **Application Equipment**
- ❑ **Calibration methods**
- ❑ Verification

VALENT BIOSCIENCES CORPORATION

Examples of Spray Equipment for WDG Application

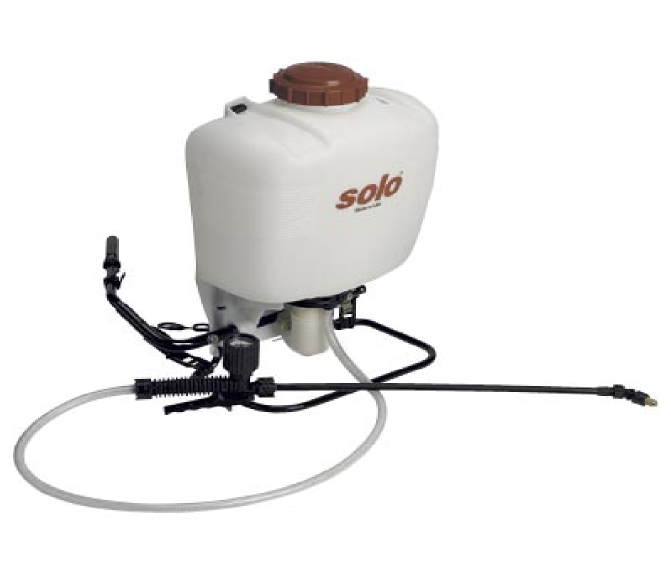

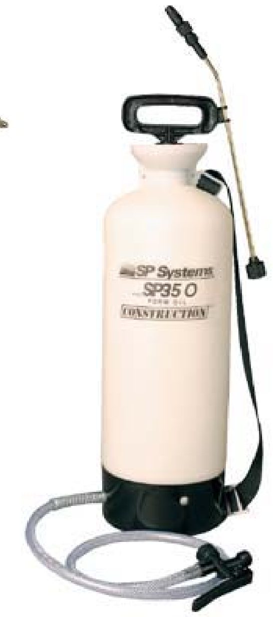

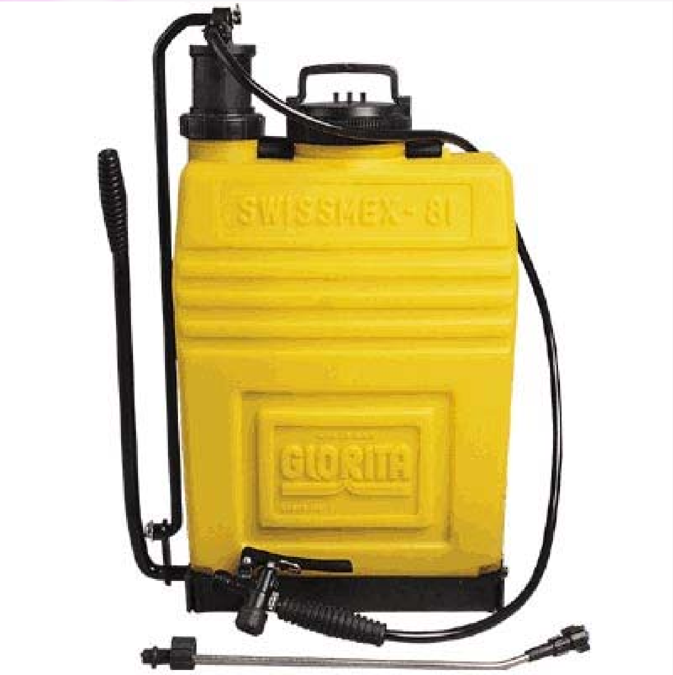

Dar UMCP
Dar es Salaam
January 2006

VALENT BIOSCIENCES CORPORATION

Why Calibrate?

REMEMBER OUR GOAL

"Give all the larvae a good FINAL meal."

- Accurate dose and even coverage of the larval habitat.
- Saves material, time and money.

VectoBac WDG dose is 400 gm/ha

We aim to achieve this dose.

Factors That Determine Application Rate

- SPEED of travel (meters per minute)
- width of SWATH (meters wide)
- FLOW rate of sprayer (liters per minute)
- DILUTION rate of product (grams per liter)

VALENT BIOSCIENCES CORPORATION

Calibration Steps for WDG

- Measure working SPEED
- Measure sprayer SWATH
- Measure sprayer FLOW
- Calculate DILUTION

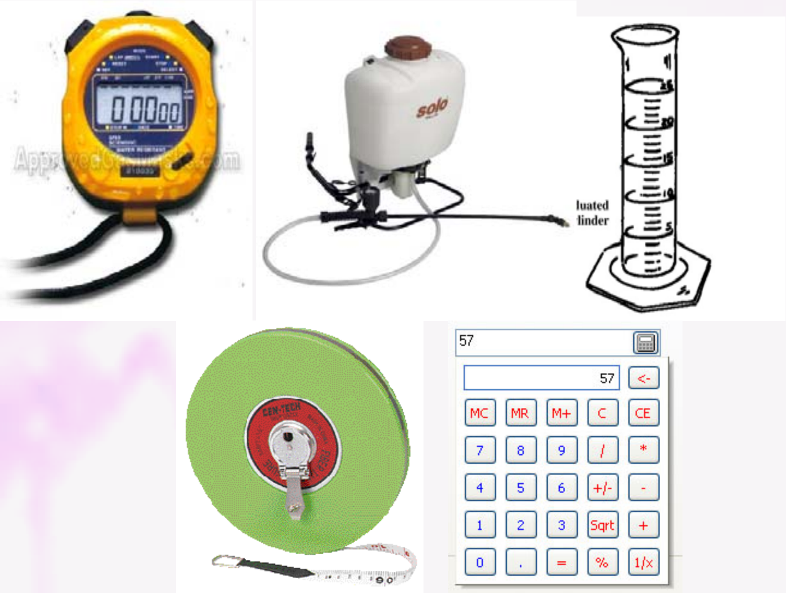

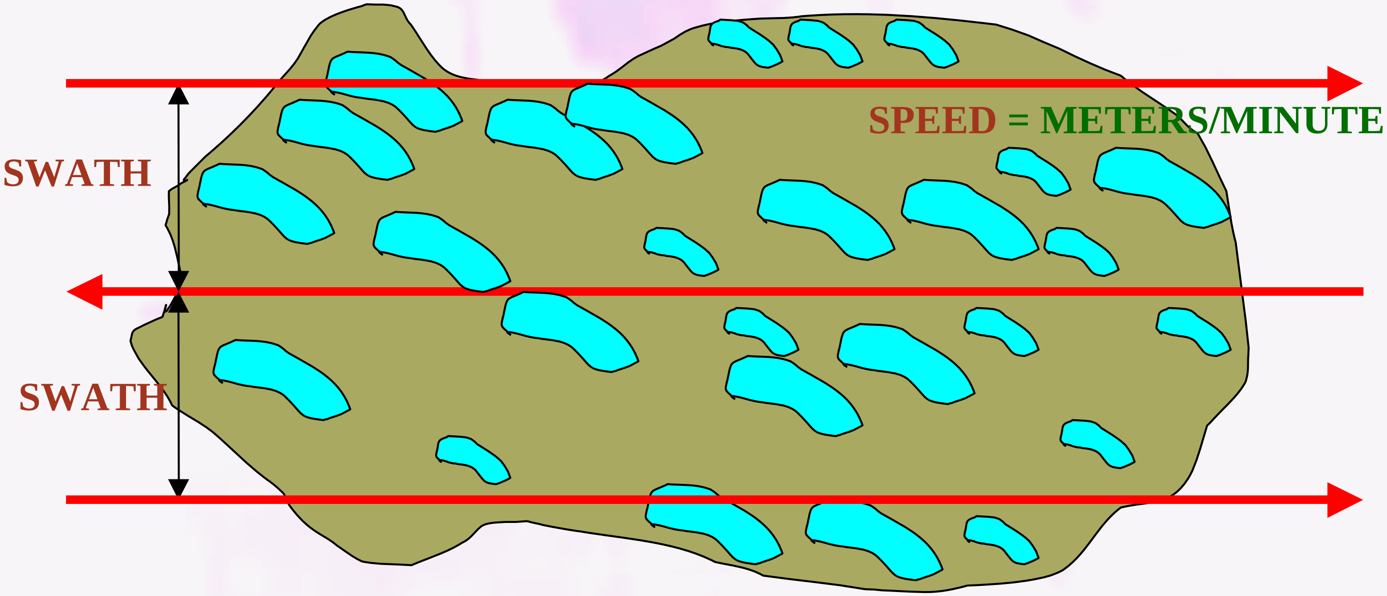

VALENT BIOSCIENCES CORPORATION

Calibration requirements

- 1 container of WDG
- 1 sprayer with D2 nozzle
- 1 Tape measure
- 1 Stop watch
- 1 Calculator
- Data forms
- Boots

Measure the distance

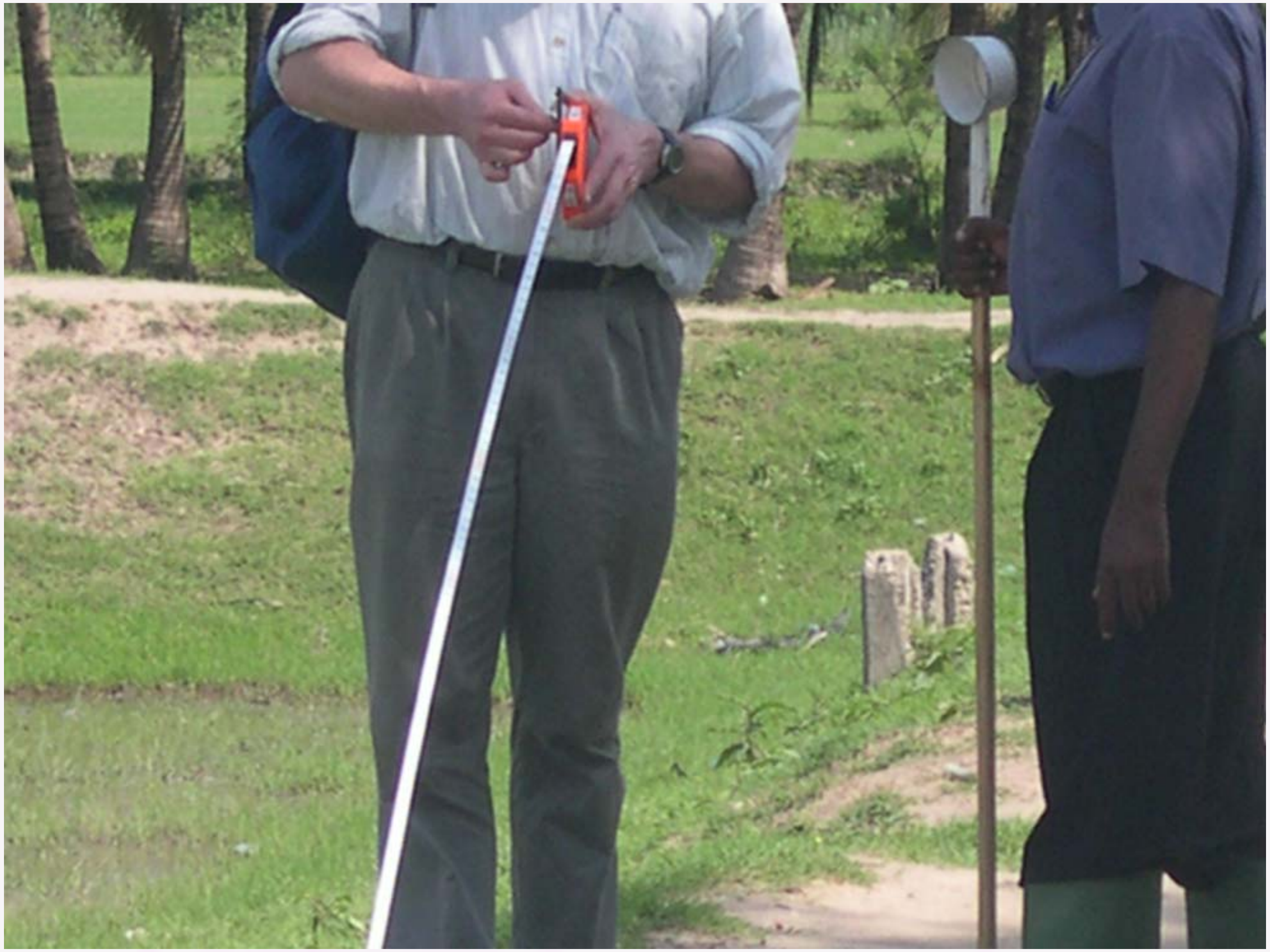

Measure the walking speed to determine the required dilution

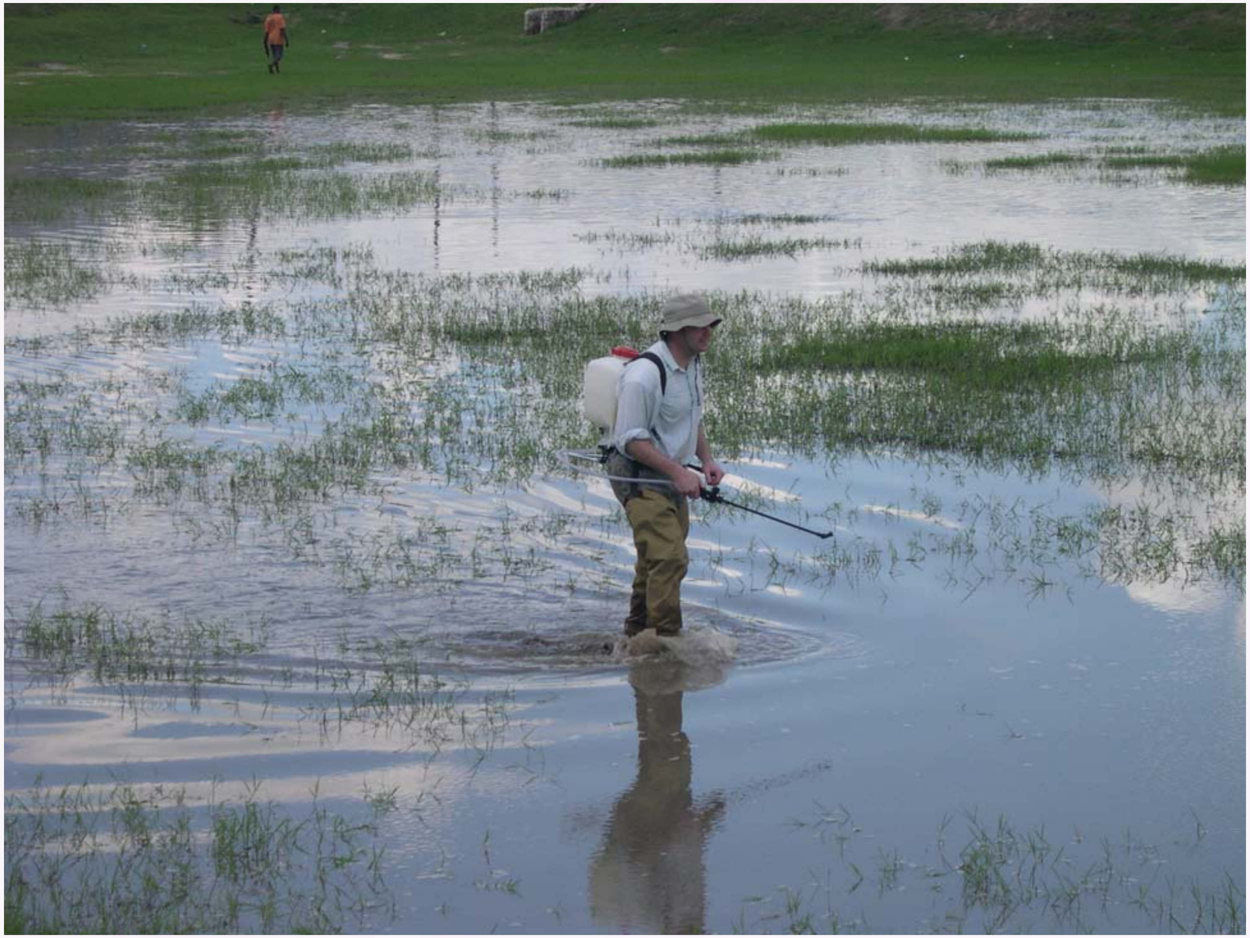

Dar UMCP
Dar es Salaam
January 2006

VALENT BIOSCIENCES CORPORATION

Measure the flow rate

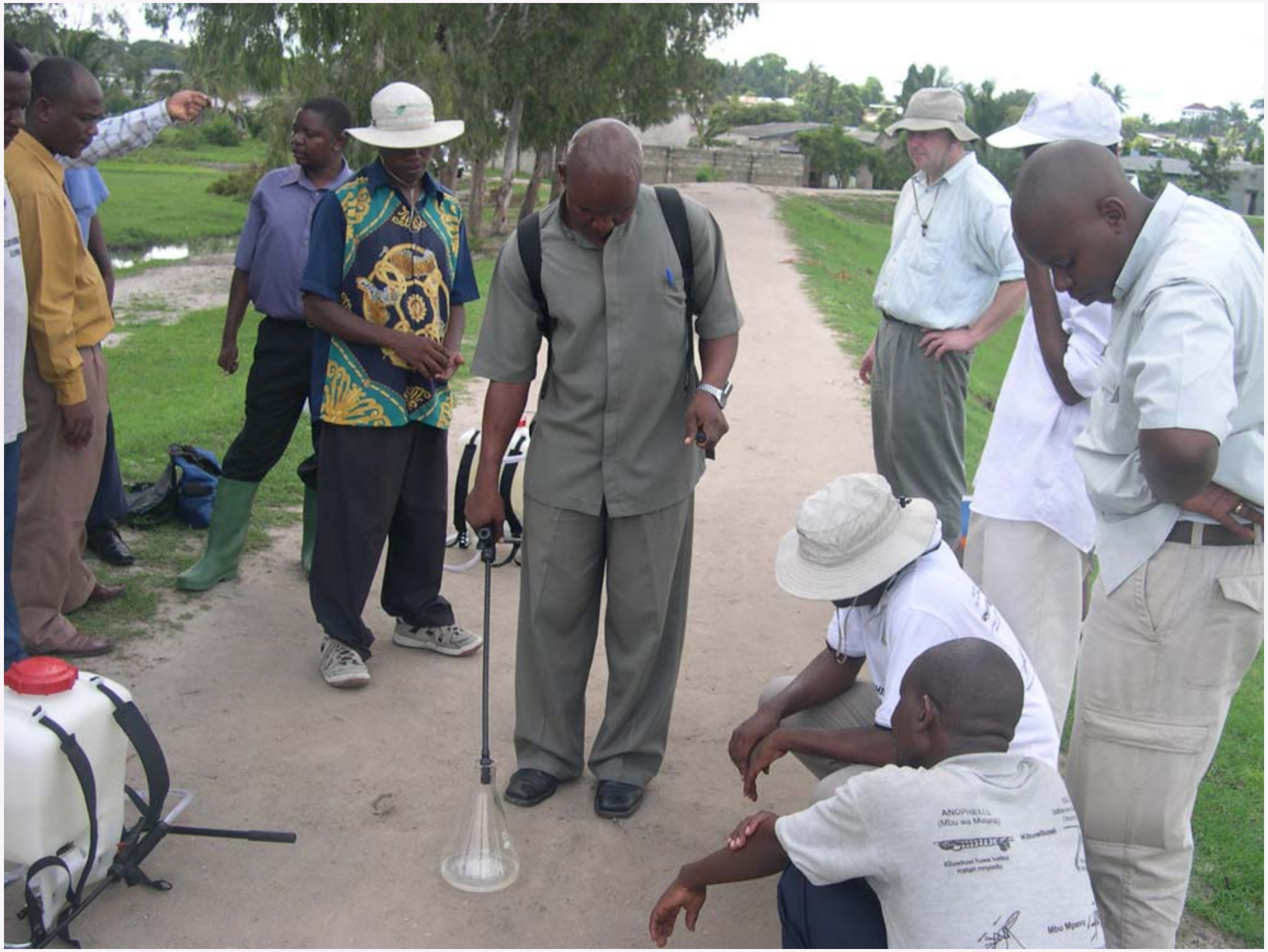

Dar UMCP
Dar es Salaam
January 2006

VALENT BIOSCIENCES CORPORATION

Backpack Spray Calibration for WDG

APPLICATION RATE = SPRAY RATE x DILUTION

(GRAMS PRODUCT PER HECTARE) = (LITERS SPRAYED PER HECTARE) X (GRAMS PRODUCT PER LITER)

DILUTION = APPLICATION RATE / SPRAY RATE

SPRAY RATE = (FLOW x 10,000)/ SPEED x SWATH

APPLICATION RATE = GRAMS PRODUCT APPLIED PER HECTARE
SPEED = METERS PER MINUTE
SWATH = METERS
FLOW = LITERS PER MINUTE
SPRAY RATE = LITERS OF SPRAY MIX APPLIED PER HECTARE
DILUTION = GRAMS PRODUCT PER LITER OF SPRAY MIX

Measuring Swath Width

- Find a flat, clean surface such as a parking lot.
- Measure “Full Swath”
  - “Full swath” will be equal to two times the projection distance using a 180 degree sweep to distribute the material.
- Apply product with appropriate sweep while stationary and measure width covered
- Subtract 50% for overlap

Results of Swath Tests - Meters

10 METER SWATH

(10 STEPS)

VALENT BIOSCIENCES CORPORATION

Measuring Flow Rates For WDG Sprays

- Flow rate of liquids measured with a graduated cylinder or other liquid measuring device.
- The spray pressure is maintained at a standard level, and spray is discharged into the cylinder for one minute.
- The flow rate per minute is determined by the volume of liquid in the cylinder.

Results of Flow Tests – Liters per Minute

AVERAGE = 0.74 LITERS PER MINUTE

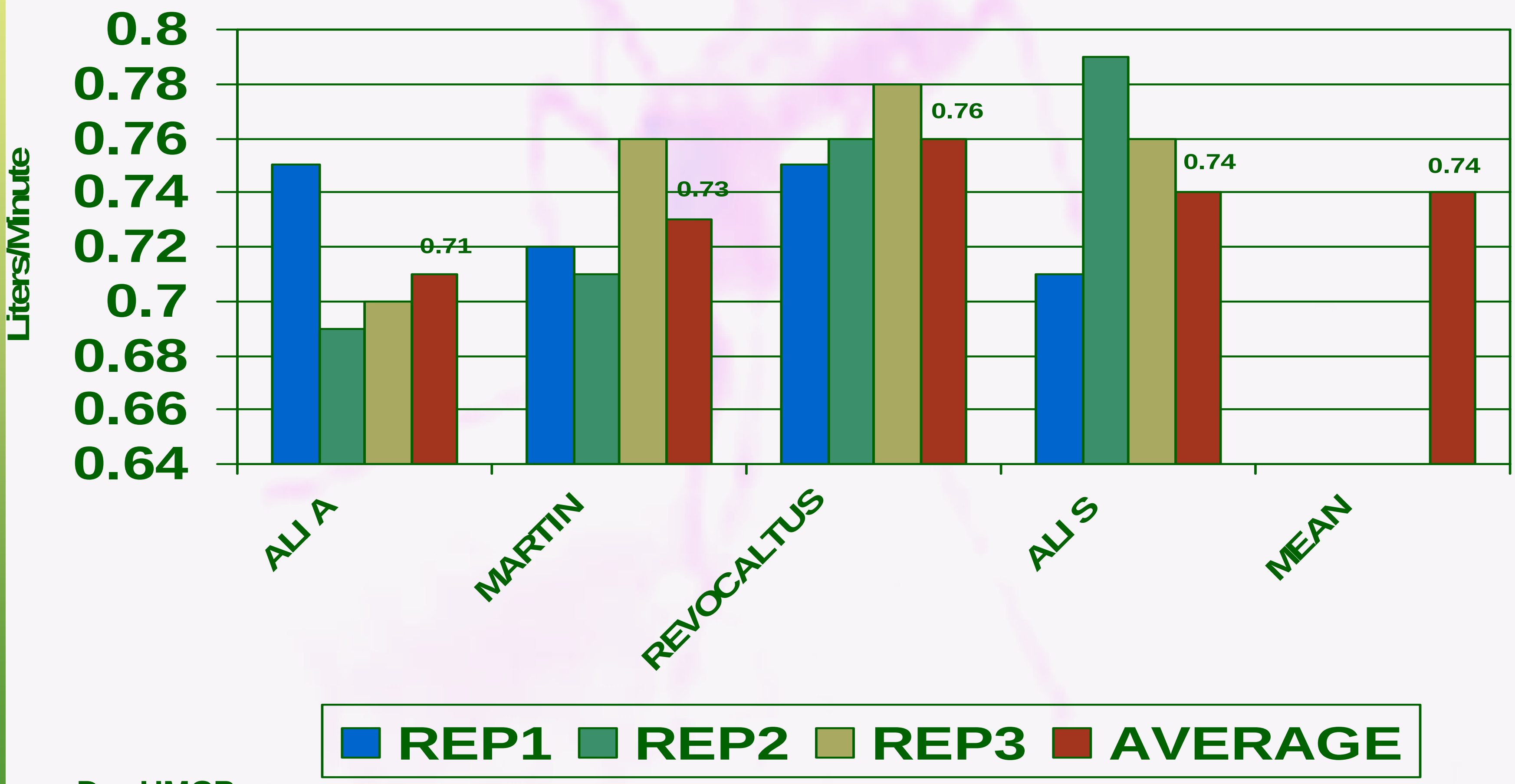

Dar UMCP
Dar es Salaam
January 2006

VALENT BIOSCIENCES CORPORATION

Measuring Your Working Speed

- Measure and mark 50 meters in typical habitat.
- Time how long it takes to walk 50 meters at a comfortable working pace while carrying equipment and pretending to spray.
- Repeat the measurement three times
- Make an average of your times
- 50 divided by average time = meters per minute

Results of Speed Tests – Meters per Minute

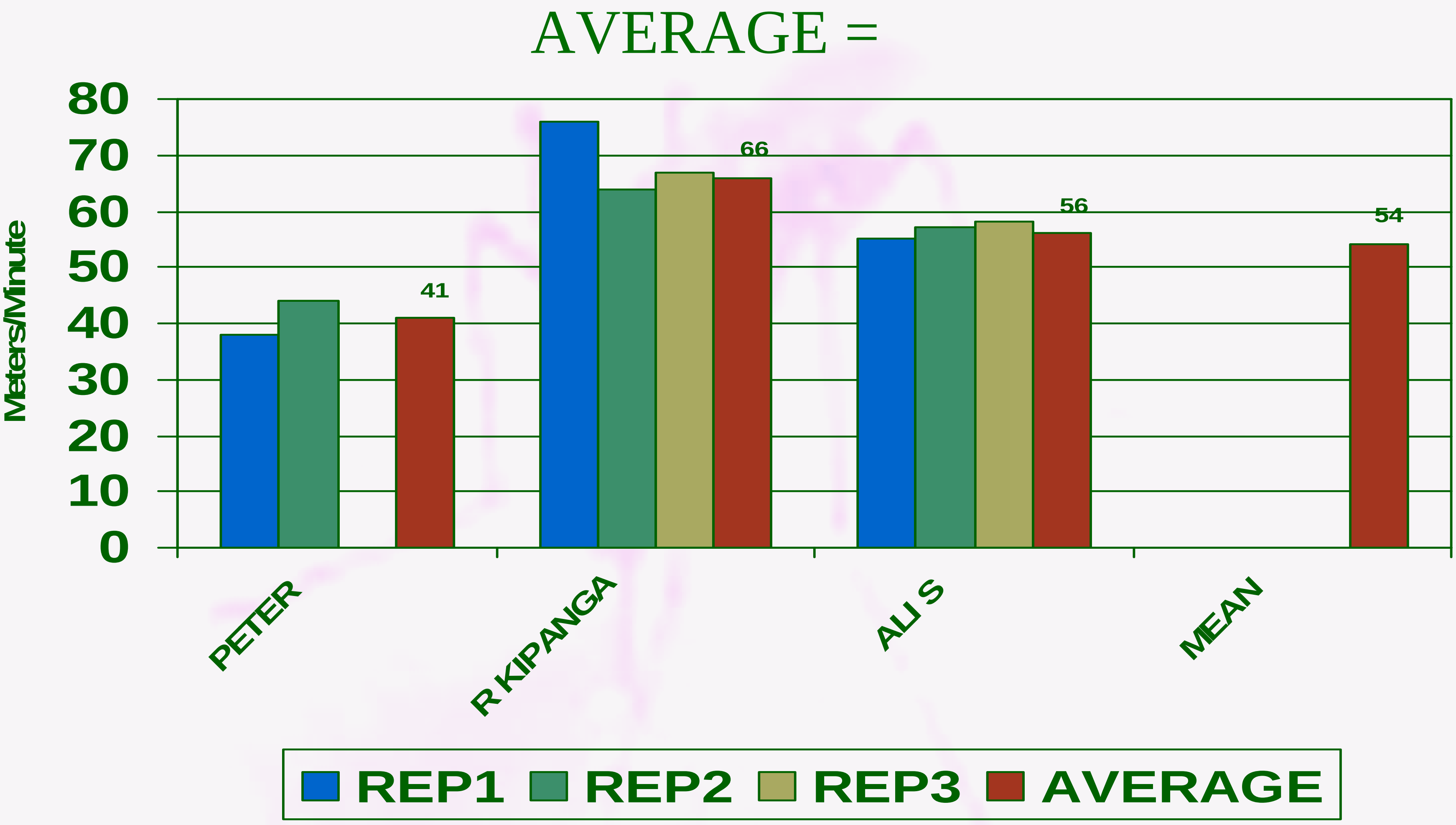

Dar UMCP
Dar es Salaam
January 2006

VALENT BIOSCIENCES CORPORATION

Calibration for VectoBac WDG

Solo Backpack with #2 disk (no core); On pressure setting #3 (approx 3 bar)

APPLICATION RATE = 400 GRAMS PER HECTARE
SPEED = 54 METERS PER MINUTE
SWATH = 10 METERS
FLOW = 0.74 LITERS PER MINUTE
SPRAY RATE = (0.74 x 10000) / 54 x 10 = 14 LITERS PER HECTARE
DILUTION = 400 GRAMS / 14 LITERS or 200 GRAMS / 7 LITERS

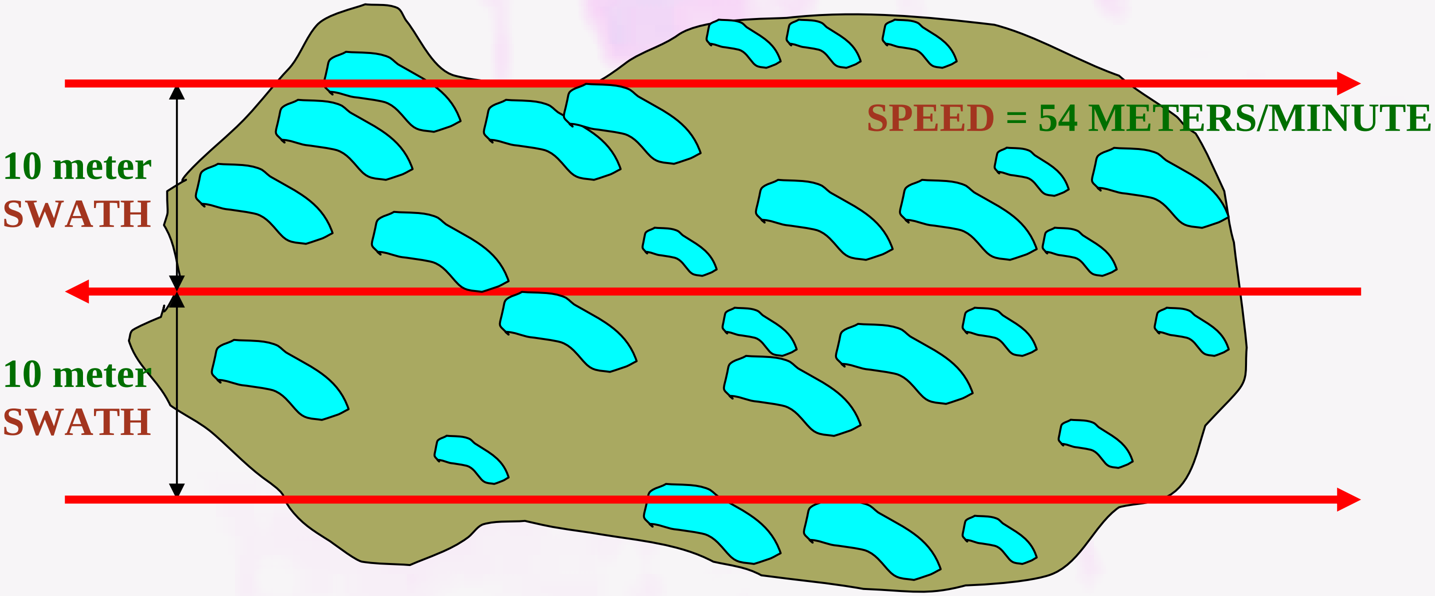

One Tank (14 l) = One Hectare

Dar UMCP
Dar es Salaam
January 2006

VALENT BIOSCIENCES CORPORATION

Mixing instructions

1) Add half of the water
2) Add pre-measured WDG slowly while shaking/stirring as you add
3) Add the rest of the water
4) Shake vigorously for 2 minutes

Standardizing Calibrations

- Calibrate each sprayer
- Repeat calibration during season.
- When sweeping the spray wand, make a full swing.
- Make a fast enough sweep for even coverage.
- Standardize against expected use rates.
- Practice, Practice, Practice…

Mix carefully and thoroughly: Add and mix small amounts at a time

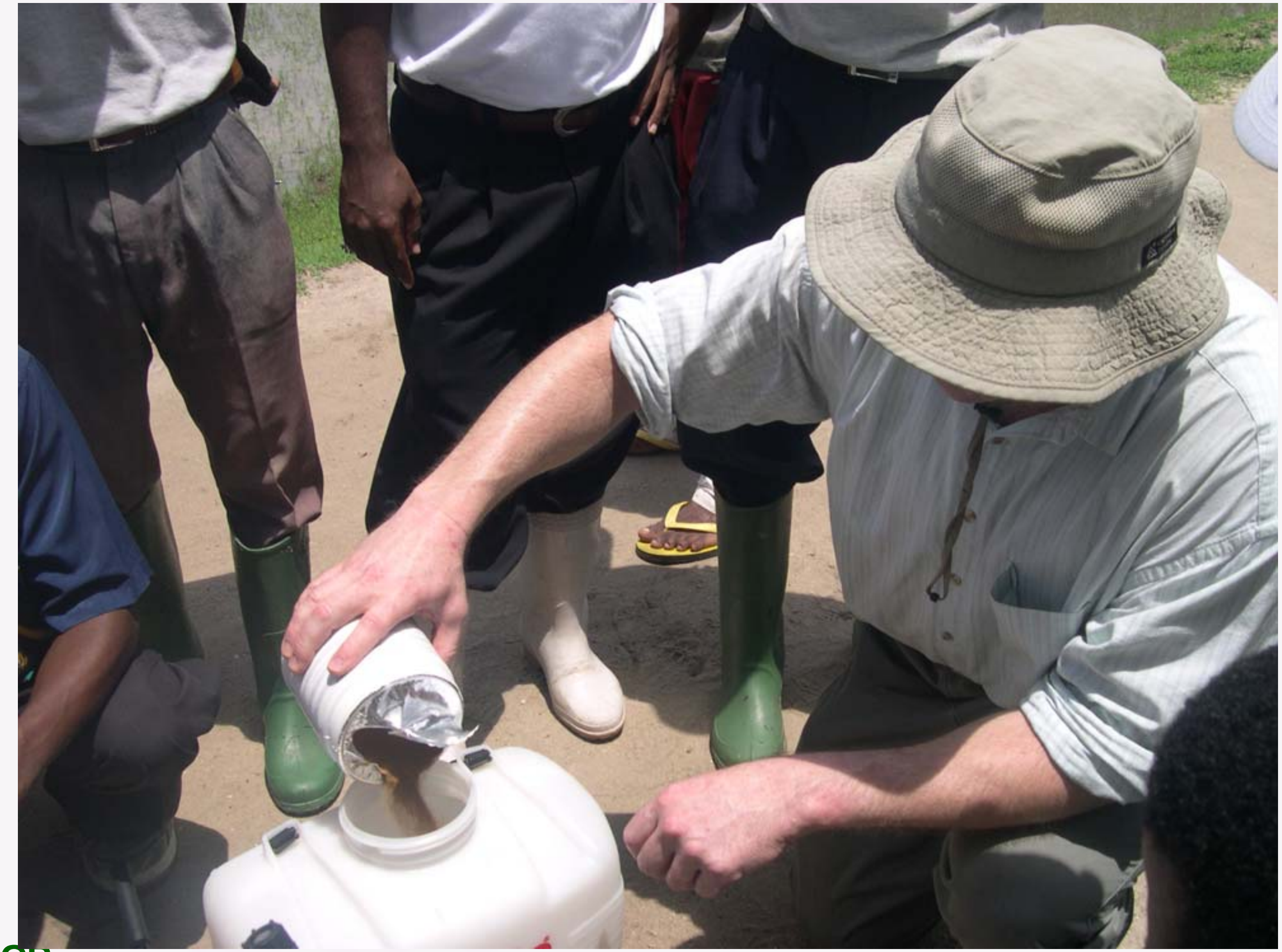

Dar UMCP
Dar es Salaam
January 2006

VALENT BIOSCIENCES CORPORATION

Apply evenly and consistently

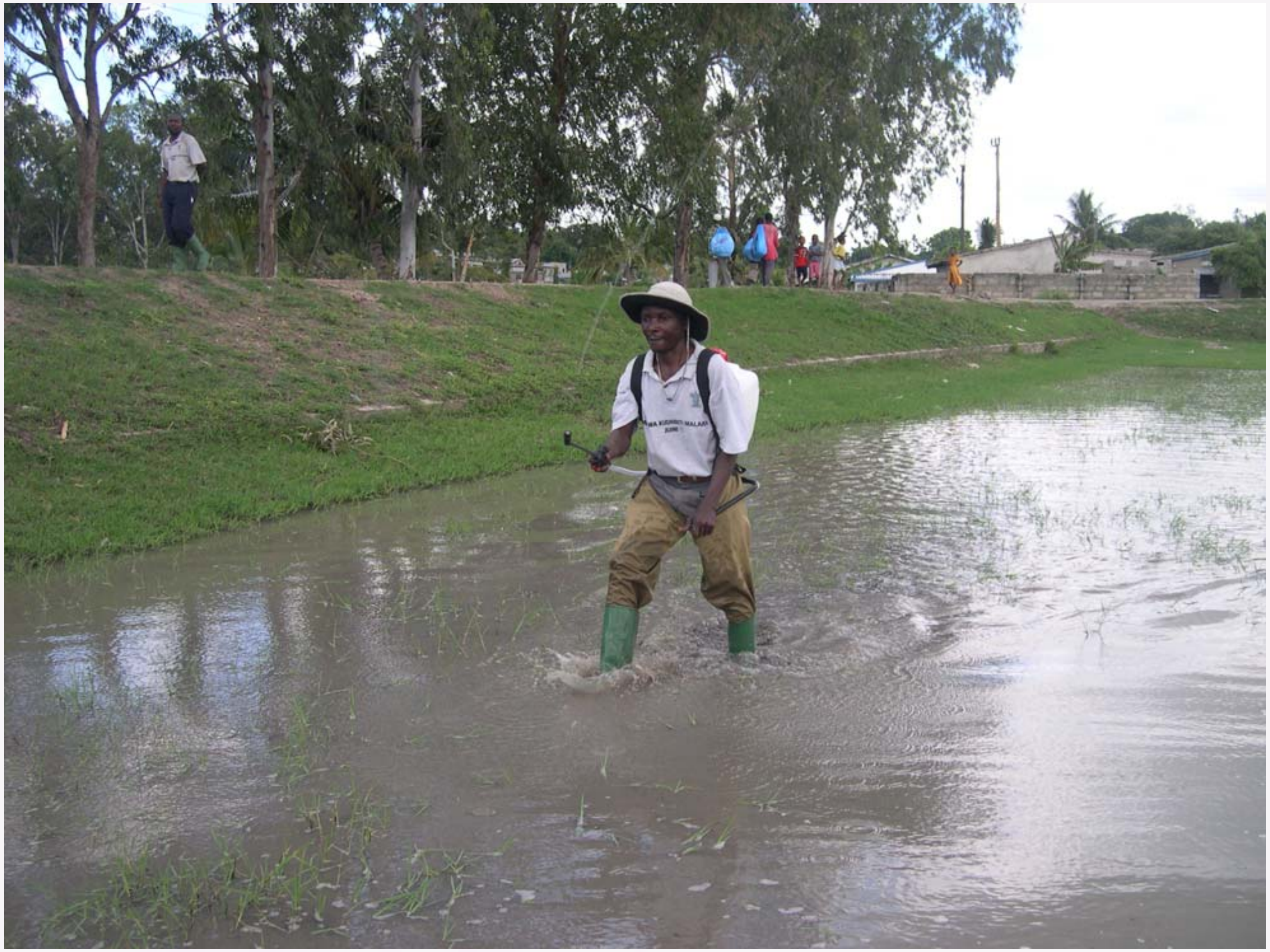

Dar UMCP
Dar es Salaam
January 2006

VALENT BIOSCIENCES CORPORATION

Treat the entire surface area with 10 meter-wide swaths

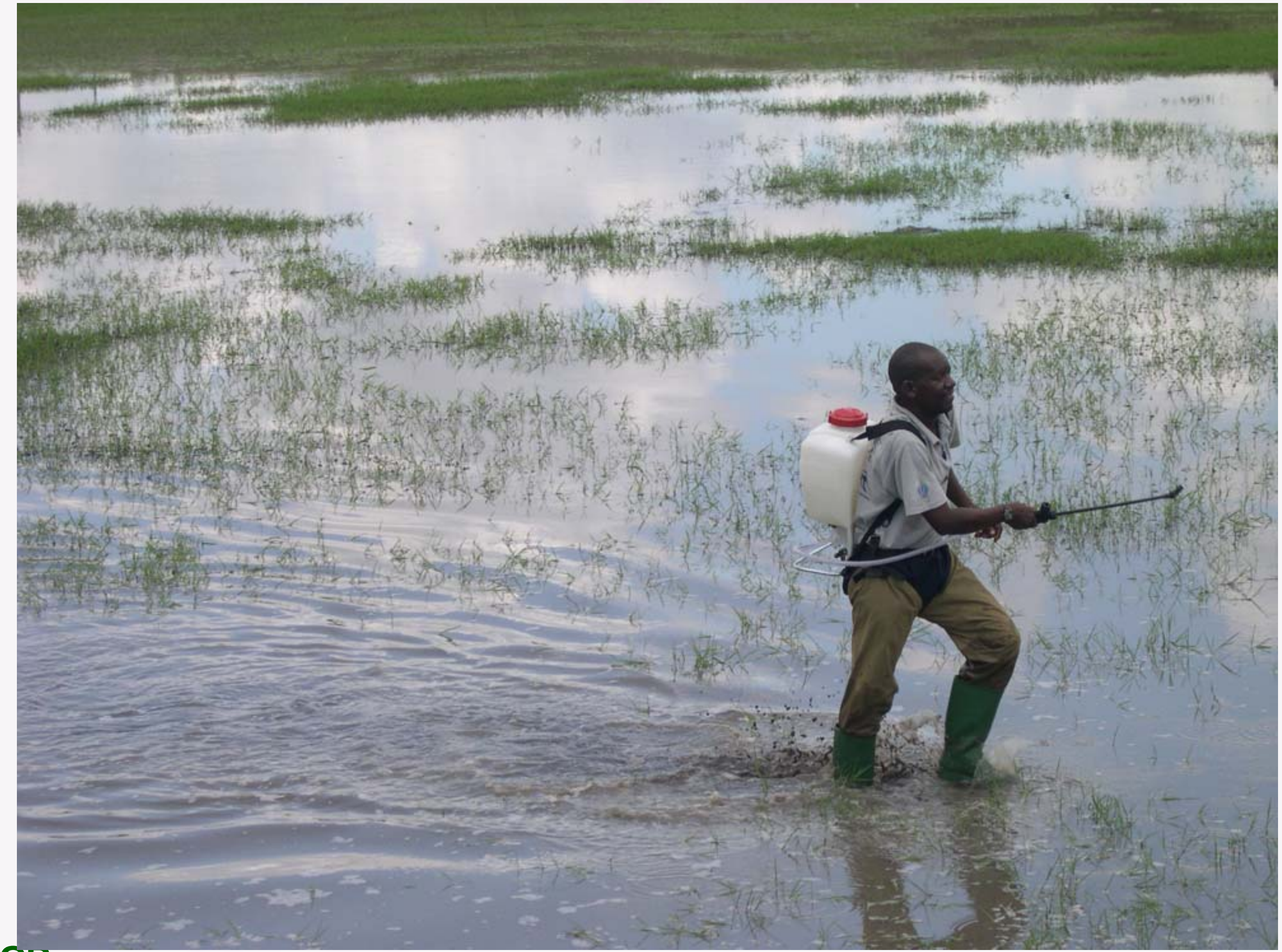

Dar UMCP
Dar es Salaam
January 2006

VALENT BIOSCIENCES CORPORATION

Verification of Application

- Does actual use match expected use.
  - Size of each area treated
  - Rates intended
  - Overall inventory vs use accounting
  - End of day match?

VALENT BIOSCIENCES CORPORATION
