# Supplementary material for: A tool box for operational mosquito larval control: preliminary results and early lessons from the Urban Malaria Control Programme in Dar es Salaam, Tanzania
Source: Malar J. 2008 Jan 25;7:20. doi: 10.1186/1475-2875-7-20 (PMC2259364; doi:10.1186/1475-2875-7-20)
Supplement: Additional file 9 — Ward-level weekly summary form for larval surveillance data and form checklist for collation in pre-labelled folders and evaluation by municipal management. The document shows the data collection form used to prepare a weekly summary of the number of aquatic habitats and their colonisation with mosquito larvae. [file 1475-2875-7-20-S9.pdf]

Weekly habitat summary data sheet

**Folder number:**		
		Code:
**Municipality:**		
**Ward:**		
**Mtaa:**		

Signature Supervisor ______________________ Date: _______/_________/________

Signature Inspector ______________________ Date: _______/_________/________

Signature Co-ordinator ______________________ Date: _______/_________/________

**Year**	**Month**	**Week**	10-cell unit	**No. of habitats**	No. of habitats with **water**	No. habitats with **Anopheles early**	No. of habitats with **Anopheles late**	No. of habitats with **Culex early**	No. of habitats with **Culex late**	No. of habitats with **pupae**	10-cell unit	**No. of habitats**	No. of habitats with **water**	No. habitats with **Anopheles early**	No. of habitats with **Anopheles late**	No. of habitats with **Culex early**	No. of habitats with **Culex late**	No. of habitats with **pupae**
2007																		

Signature:
